# Genetic diversity and population genetic structure of Cambodian indigenous chickens

**DOI:** 10.5713/ab.21.0351

**Published:** 2022-01-05

**Authors:** Theary Ren, Mitsuo Nunome, Takayuki Suzuki, Yoichi Matsuda

**Affiliations:** 1 General Directorate of Animal Health and Production, National Animal Health and Production Research Institute, Phnom Penh 12352, Cambodia; 2 Asian Satellite Campuses Institute, Nagoya University, Nagoya 464-8601, Japan; 3 Avian Bioscience Research Center, Graduate School of Bioagricultural Sciences, Nagoya University, Nagoya 464-8601, Japan; 4 Laboratory of Avian Bioscience, Department of Animal Sciences, Graduate School of Bioagricultural Sciences, Nagoya University, Nagoya 464-8601, Japan

**Keywords:** Clustering Analysis, Large-scale Population Genetic Study, Microsatellite, Mitochondrial DNA D-loop Sequence, Phylogenetic Tree

## Abstract

**Objective:**

Cambodia is located within the distribution range of the red junglefowl, the common ancestor of domestic chickens. Although a variety of indigenous chickens have been reared in Cambodia since ancient times, their genetic characteristics have yet to be sufficiently defined. Here, we conducted a large-scale population genetic study to investigate the genetic diversity and population genetic structure of Cambodian indigenous chickens and their phylogenetic relationships with other chicken breeds and native chickens worldwide.

**Methods:**

A Bayesian phylogenetic tree was constructed based on 625 mitochondrial DNA D-loop sequences, and Bayesian clustering analysis was performed for 666 individuals with 23 microsatellite markers, using samples collected from 28 indigenous chicken populations in 24 provinces and three commercial chicken breeds.

**Results:**

A total of 92 haplotypes of mitochondrial D-loop sequences belonging to haplogroups A to F and J were detected in Cambodian chickens; in the indigenous chickens, haplogroup D (44.4%) was the most common, and haplogroups A (21.0%) and B (13.2%) were also dominant. However, haplogroup J, which is rare in domestic chickens but abundant in Thai red junglefowl, was found at a high frequency (14.5%), whereas the frequency of haplogroup E was considerably lower (4.6%). Population genetic structure analysis based on microsatellite markers revealed the presence of three major genetic clusters in Cambodian indigenous chickens. Their genetic diversity was relatively high, which was similar to findings reported for indigenous chickens from other Southeast Asian countries.

**Conclusion:**

Cambodian indigenous chickens are characterized by mitochondrial D-loop haplotypes that are common to indigenous chickens throughout Southeast Asia, and may retain many of the haplotypes that originated from wild ancestral populations. These chickens exhibit high population genetic diversity, and the geographical distribution of three major clusters may be attributed to inter-regional trade and poultry transportation routes within Cambodia or international movement between Cambodia and other countries.

## INTRODUCTION

The ancestral relationships between junglefowl (genus *Gallus*) in the wild, and the original domestication event are of considerable interest with respect to establishing the evolutionary history of domestic chickens and their genetic diversity. In this regard, DNA-based analyses of mitochondrial DNA (mtDNA) D-loop sequences has revealed that the Indochinese subspecies of the red junglefowl (*Gallus gallus gallus*) is the primary maternal ancestor of domestic chickens [1,2]. That the red junglefowl is the main progenitor of domestic chickens is also supported by the findings of other studies that have used microsatellite markers [3]. On the basis of the examination of a large number of mtDNA D-loop sequences from domestic chickens worldwide and four red junglefowl subspecies (*Gallus gallus gallus*, *G. g. spadiceus*, *G. g. jabouillei*, and *G. g. bankiva*), Liu et al [4] indicated the possibility that domestic chicken breeds are derived from at least three subspecies of *G. gallus*, and also that there are at least two centers of domestication, namely, Southeast Asia and the Indian subcontinent. However, the monophyletic origin of domestic chickens from the red junglefowl remains contentious, given that there is a non-negligible genetic contribution from other junglefowl species such as the grey junglefowl (*Gallus sonneratii*) and Ceylon junglefowl (*Gallus lafayeti*) [5,6]. Liu et al [4] have suggested that there are nine highly divergent clades (named haplogroups A to I) related to geographical distribution in a wide range of domestic chickens and red junglefowl across Eurasian regions. Moreover, on the basis of an investigation of 4,938 mitochondrial DNA fragments, including 2,843 previously published and 2,095 de novo sequences from 2,044 domestic chickens and 51 red junglefowl, Miao et al [7] phylogenetically classified these into 15 haplogroups, A to K and W to Z. Among these, the common haplogroups A to G are found in both domestic chickens and red junglefowl, whereas the rare haplogroups H to I and W to Z are specific to domestic chickens and red junglefowl, respectively.

In Southeast Asia, Cambodia is located on the Indochina Peninsula, which lies within the distribution range of the red junglefowl, which is acknowledged to be the common ancestor of present-day domestic chickens [1,2,4]. Indigenous chickens are commonly bred in free range settings by smallholder farmers, and given the superior quality and flavor of their meat, the demand for indigenous chickens exceeds that for broilers or commercial hybrid chickens in Cambodia [8]. Accordingly, these birds command higher prices. Moreover, indigenous chickens typically have a higher tolerance to heat and stress than commercial chickens, as well as a better resistance to diseases. Consequently, indigenous chickens represent an important source of income for smallholder poultry producers in developing countries [9]. However, given certain disadvantages, such as small body size, low growth rate, low productivity caused by a low egg-laying rate, and limited supply from smallholder farmers, the genetic resources of Cambodian indigenous chickens are currently in decline [10,11]. Thus, at present, the import of chicken meat from other countries is necessary to meet domestic demand. Poultry production in Cambodia is, nevertheless, still dominated by smallholder farmers, who typically raise birds using the backyard system, which is more prone to outbreaks of highly pathogenic avian influenza (HPAI) than the confinement rearing system that generally has epidemic prevention measures [9]. However, advances in clean poultry breeding systems have led to a decline in traditional poultry farming, and consequently, indigenous chicken populations are threatened by both the modernization of poultry farming and outbreaks of HPAI. Furthermore, increasing expansion of the commercial chicken industry and the intermixture of commercial hybrids with indigenous chickens have contributed to an erosion of the genetic variation and uniqueness of indigenous chickens. Consequently, assessments of the genetic diversity and population genetic structure of Cambodian indigenous chickens are deemed essential, in terms of both determining their unique identity and conserving them as a high-quality genetic resource that represents both a heritage and reservoir of genetic variability, which can be utilized to improve commercial chicken breeds or establish novel breeds [12,13]. To date, however, although the phenotypic characteristics of four Cambodian indigenous chicken breeds have been reported [14], there has yet been no genetic characterization of indigenous chickens in Cambodia.

mtDNA D-loop sequences have been widely used for ge netic characterization of wild and domestic chickens [4,7,15], owing to their notable beneficial characteristics: i) the mtDNA D-loop region is readily amplified using the polymerase chain reaction (PCR); ii) given the high rates of polymorphism, the D-loop is suitable for examining intraspecific genetic variation; and iii) a large number of D-loop sequences are available in the public domain that enable analyses of maternal phylogenetic relationships among chickens worldwide. Similarly, owing to their high polymorphism, microsatellite markers are also among the most powerful molecular tools for estimating genetic diversity among chicken populations and their genetic structures [3,16], and microsatellite markers recommended by the Food and Agriculture Organization (FAO) are openly available [17]. Indeed, microsatellites have been successfully applied in analyses of the genetic diversity and population structure of chickens [3,18–20].

The aim of the present study was to assess the genetic diversity of Cambodian indigenous chickens and their phylogenetic relationships with indigenous chickens in other Asian countries and the red junglefowl, using mtDNA D-loop sequences and microsatellites as diagnostic molecular tools. We conducted a large-scale population genetic assessment of 28 populations of indigenous chickens, collected from across 24 provinces, as well as three commercial chicken breeds reared in Cambodia. To the best of our knowledge, this study represents the first attempt to determine the genetic diversity and population genetic structure of Cambodian indigenous chickens.

## MATERIALS AND METHODS

### Sample collection

**Figure 1:**
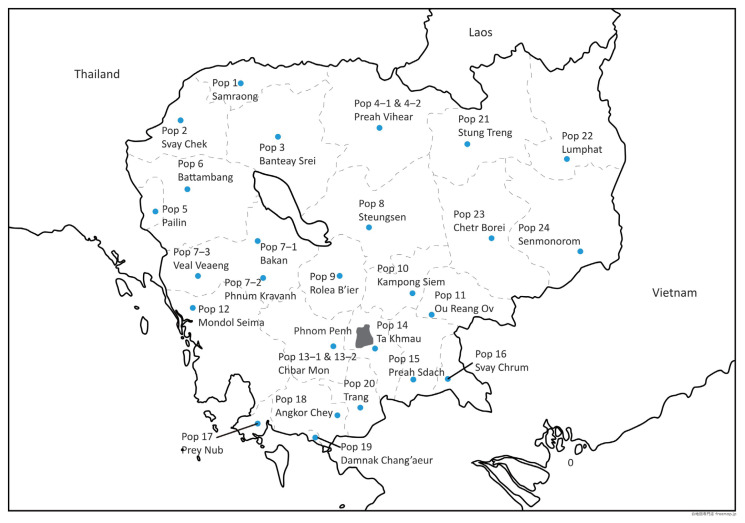
Map of localities where the blood samples of indigenous chickens were collected.

Between 2018 and 2020, blood samples were collected from 690 chickens at locations throughout Cambodia: 646 indigenous chickens from 28 villages in 26 districts of 24 provinces (blood samples were collected from one farm population in each village) and 44 commercial chickens of three breeds [nine three-way cross hybrids between indigenous chickens and commercial chickens (hereafter referred to as the “three-way hybrid chicken breed”) and 29 Isa Brown chickens from Phnom Penh, and six Rhode Island White chickens from Kandal Province (Table 1; Figure 1). Blood was collected using heparinized syringes and stored in vacutainer tubes containing ethylenediaminetetraacetic acid (Hong Thien My Medical Equipment Joint Stock Company, Ho Chi Minh, Vietnam) at 4°C until use. All procedures conducted in the study adhered to the guidelines for the care and use of experimental animals at Nagoya University, and the experimental protocols were approved by the Animal Experiment Committee of the Graduate School of Bioagricultural Sciences, Nagoya University (approval No. 2018031348).

### DNA extraction

Total genomic DNA was extracted from 20-μL blood samples using an ISOSPIN Blood and Plasma DNA Kit (Nippon Gene, Toyama, Japan) according to the manufacturer’s protocol.

### Polymerase chain reaction amplification and sequencing of the mtDNA D-loop region

The complete mtDNA D-loop region was PCR amplified using the primer set: Gg_Dloop_1F (5′-AGGACTACGGCTT GAAAAGC-3′) [5] and Gg_Dloop_5R (5′CTTCAGTGC CATGCTTTGTG-3′), which was designed in this study using Primer3web version 4.1.0 (https://primer3.ut.ee/). PCR amplification was carried out in 10-μL reaction mixtures containing 50 ng genomic DNA, 4 pmol of each primer, and 5.0 μL of SapphireAmp Fast PCR Master Mix (Takara Bio, Otsu, Japan). The cycling conditions were as follows: an initial denaturation at 94°C for 1 min; followed by 35 cycles of denaturation at 94°C for 20 s, annealing at 61°C for 5 s, and elongation at 72°C for 10 s; and a final extension at 72°C for 5 min. The PCR products were detected by electrophoresis on 1.5% agarose gels and then purified using the 20% polyethylene glycol/2.5 M NaCl precipitation method [21,22]. Cycle sequencing was performed using a BigDye Terminator v3.1 Cycle Sequencing Kit (Thermo Fisher Scientific, Waltham, MA, USA), and nucleotide sequences were determined using an ABI PRISM 3130 Genetic Analyzer (Thermo Fisher Scientific, USA).

### Polymerase chain reaction amplification of microsatellite DNA markers

Genotyping was also performed using 23 microsatellite markers, which were selected from among the 30 markers recommended by the FAO for studying the genetic diversity of chickens [17] (Supplementary Table S1). PCR amplification was performed via multiplex PCR based on two different microsatellite loci, using 10-μL reaction mixtures containing approximately 50 ng genomic DNA, 10 pmol of each primer, and 5.0 μL of AmpliTaq Gold 360 Master Mix (Thermo Fisher Scientific, USA). The cycling conditions were as follows: an initial denaturation at 95°C for 10 min; followed by 35 cycles of denaturation at 95°C for 30 s, annealing at 55°C for 30 s, and elongation at 72°C for 30 s; and a final extension at 72°C for 7 min. PCR products were electrophoresed with Hi-Di Formamide (Thermo Fisher Scientific, USA) and a GeneScan 600 LIZ Size Standard (Thermo Fisher Scientific, USA) using the ABI PRISM 3130 Genetic Analyzer. Allele sizes were determined using Geneious Prime v2020.2.2 (Biomatters, Auckland, New Zealand).

### Phylogenetic analysis of mtDNA D-loop sequences

DNA sequences were aligned against the chicken mitochondrial reference genome (accession No. X52392) [23] using Geneious Prime v2020.2.2. To determine the phylogenetic positions of Cambodian indigenous chickens among chicken populations worldwide, a Bayesian phylogenetic tree was constructed using BEAST v2.4.3 [24] based on 165 mtDNA D-loop sequences obtained from GenBank (Supplementary Table S2, S3), which were used as reference sequences for D-loop haplogroups A to K and W to Z. A D-loop sequence of Ceylon junglefowl (*Gallus lafayettei*) (NC_007239) [5] was used as an outgroup sequence. Phylogenetic analysis was performed with 20 million Markov Chain Monte Carlo (MCMC) generations, sampling one tree every 2,000 generations. The optimal nucleotide substitution model for the sequences was selected based on the Bayesian information criterion using jModelTest v2.1.10 [25,26], and the convergence of the runs was verified using Tracer v1.7.1 [27]. After discarding the initial 10% of 10,000 sampled trees as burn-in, a maximum clade credibility tree was constructed from the remaining trees using Tree Annotator v2.4.3 [24]. A diagrammatic representation of the summarized tree was generated using FigTree v1.4.2. The haplotypes of the sequences were determined using the Mito-ToolPy program [28].

### Genetic diversity analysis of mtDNA D-loop sequences

The nucleotide diversity (*π*) [29], number of haplotypes (*h*), and Watterson estimator per sequence (*Theta-w*) [30] were calculated using DnaSP v6 [31].

### Genetic diversity analysis using microsatellite markers

Genetic diversity indices, namely, the number of alleles (*A*), allelic richness (*AR*), the mean number of alleles per population (*Na*), null allele frequency (*NAF*), and F-statistics (*F_IS_*, *F_ST_*, and *F_IT_*), were calculated for each microsatellite DNA marker, using Microsatellite Analyzer v4.05 (*A* and *AR*) [32], GenAlEx v6.5 (*Na*, *F_IS_*, *F_ST_*, and *F_IT_*) [33], and Cervus v3.0.7 (*NAF*) [34,35] (Supplementary Table S1). The mean number of effective alleles (*Ne*), observed heterozygosity (*Ho*), and expected heterozygosity (*He*) were also calculated using GenAlEx 6.5.

### Population structure analysis using microsatellite markers

Bayesian clustering analysis was conducted to infer the number of genetic clusters using STRUCTURE v2.3 [36]. Log probability values from K = 1 to K = 29 were estimated for a sampling period of 100,000 MCMC generations after a burn-in period of 100,000 generations under the admixture model and the correlated allele frequency model [37]. Twenty independent MCMC runs were carried out for each K value, among which, runs with variances of log-likelihood values more than twice as large as those of the other MCMC runs were excluded from subsequent analyses. The clustering patterns of the remaining runs were analyzed to generate a major clustering pattern for each K using CLUMPAK [38]. The optimal K value was then determined based on the delta-K values from K = 2 to K = 28 using the Evanno method [39] using Structure Harvester v0.6.94 [40].

## RESULTS

### Genetic diversity of Cambodian indigenous chickens

The genetic diversity of 28 village populations of Cambodian indigenous chickens collected from 24 provinces and three commercial chicken breeds was assessed using mtDNA D-loop sequences and microsatellite markers (Table 1). The average values for the number of mtDNA D-loop haplotypes, nucleotide diversity (*π*), and *Theta-W* per population were 7.89, 0.006, and 6.14, respectively. For the genetic diversity indices based on the selected 23 microsatellite markers, the average values of *AR*, *Na*, *Ne*, *Ho*, and *He* per population were 3.92, 5.43, 3.12, 0.62, and 0.62, respectively. Values higher than the average values of each index are underlined in Table 1. The average *F* value was 0.003, ranging from −0.097 for Klengpor village in Banteay Meanchey to 0.156 for Pruok village in Ratanakiri, which indicates a low level of inbreeding within each population. Population in Klengpor village in Banteay Meanchey (Pop 2), Chamkar Ou village in Pursat (Pop 7-1), and Chamkadoung village in Kampong Speu (Pop 13-1) showed relatively low genetic diversity, as determined by both mtDNA D-loop sequences and microsatellite markers. Despite the small sample size, the three-way hybrid chicken breed showed high genetic diversity at the mtDNA level, compared with the other two commercial chicken breeds, Isa Brown and Rhode Island White.

### Haplotype diversity of mtDNA D-loop sequences in Cambodian indigenous and commercial chickens

**Figure 2:**
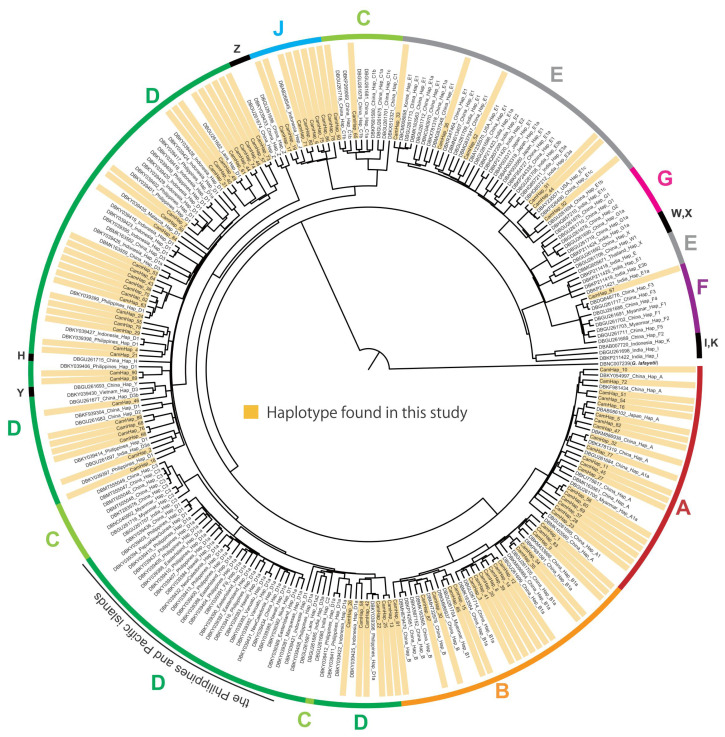
Bayesian phylogenetic tree constructed based on mtDNA D-loop haplotypes of 625 sequences from indigenous chickens and commercial chickens in Cambodia (highlighted with orange boxes) and 165 sequences obtained from the GenBank database. Phylogenetic positions of haplogroups A to F and J are specified on the different colored circumferential lines. The locations of rare haplogroups H, I, K, W, X, Y, and Z are shown with black circumferential lines.

A total of 92 haplotypes were detected among the 625 sampled Cambodian chickens, which consisted of 585 indigenous chickens from 28 populations in 24 provinces and 40 individuals from the three commercial chicken breeds (Supplementary Table S4, S5). Among the 92 haplotypes, 89 and 11 were detected in indigenous chickens and commercial chickens, respectively, with eight haplotypes being held in common, whereas the other three haplotypes (CamHap_90, 91, and 92) were specific to the commercial chicken breeds. The 92 haplotypes were classified into haplogroups A to F and J (Figure 2; Table 2), with haplogroup D being the predominant type in Cambodian indigenous chickens (44.4%), and the frequencies of haplogroups A and B being the second and fourth highest, respectively (A, 21.0%; B, 13.2%). Haplogroup J, which is one of the rare haplogroups detected in domestic chickens worldwide, was the third most frequent haplogroup identified in Cambodian indigenous chickens (14.5%), whereas in contrast, haplogroup E, which is the predominant haplogroup in domestic chickens worldwide, showed less than one-third the frequency of haplogroup J (4.6%) in the indigenous chickens. The 27 indigenous chickens with haplogroup E have the same haplotypes as commercial chickens (CamHap 33, 36, and 51) (Supplementary Table S4). Haplotypes of haplogroup D that are contained in the clade of The Philippines and Pacific island population were not detected in any of the Cambodian indigenous chickens (Figure 2), and haplogroups C (1.4%) and F (0.9%) were rare in these chickens.

### Genetic clusters of Cambodian indigenous chickens

**Figure 3:**
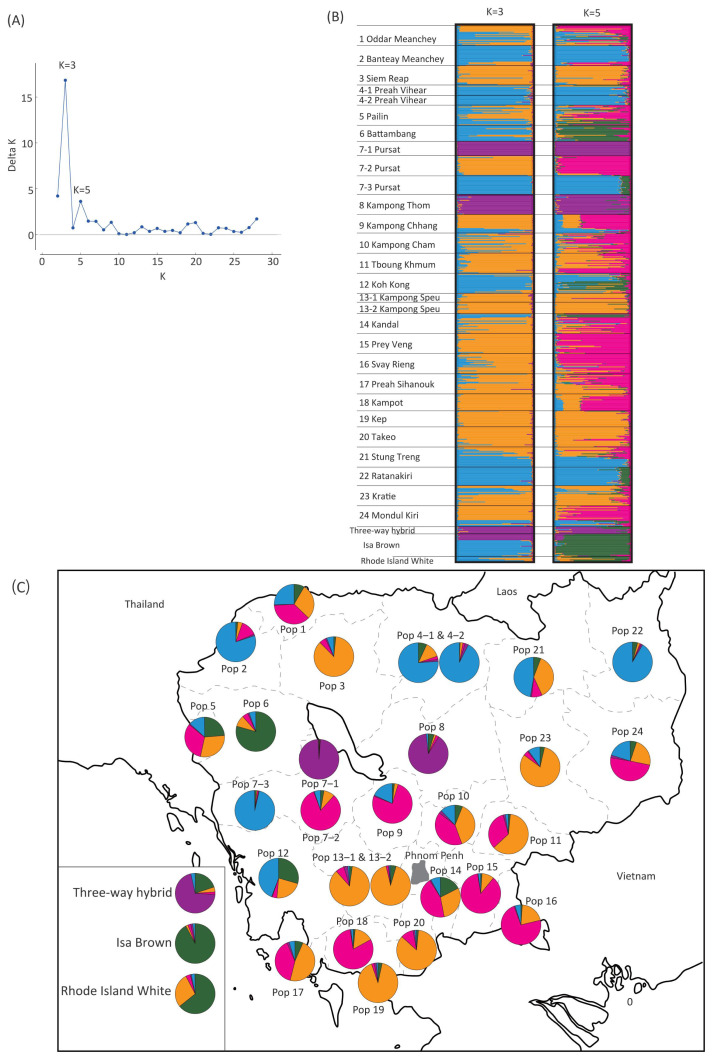
Genetic population structures of Cambodian indigenous and commercial chickens based on microsatellite markers. (A) Delta K values were calculated using Structure Harvester. The highest and second highest delta-K were exhibited at K = 3 and K = 5, respectively. (B) Structure plot for 28 village populations and three commercial breeds at K = 3 and K = 5. Each horizontal bar represents one individual. Assignment probabilities of each individual in the genetic clusters at K = 3 and K = 5 are indicated as proportions of three and five colors in each bar, respectively. (C) Geographic distributions of five genetic clusters. The proportion of five clusters in each population is shown with five different colors in the circle.

Genotyping of the 23 microsatellite markers was carried out for a total of 666 individuals, which consisted of 623 indigenous chickens from 28 populations in 24 provinces and 43 individuals from the three commercial chicken breeds. Structure Harvester analysis indicated that the highest and second highest delta K values were K = 3 and K =5, respectively (Figure 3A). At K = 3 and K = 5, indigenous chickens of Chamkar Ou village in Pursat (Pop 7-1), Preybanlek village in Kampong Thom (Pop 8), and the three-way hybrid chicken breed were assigned to the same cluster (shown in purple at K = 3 and K = 5 in Figure 3B). At K = 5, the Toul Ta Aek village population in Battambang (Pop 6) was grouped the same cluster as the two commercial chicken breeds, Isa Brown and Rhode Island White (shown in green). To determine the geographical distributions of the five genetic clusters at K = 5, we represented the percentage of each cluster in populations using pie charts (Figure 3C). The orange cluster was widely distributed in many village populations throughout Cambodia, whereas the blue and magenta clusters tended to be distributed predominantly in the northern part and the central and southern parts of Cambodia, respectively.

## DISCUSSION

Mitochondrial DNA D-loop sequences of the red junglefowl and domestic chickens have previously been phylogenetically classified into 15 haplogroups, namely, A to K and W to Z [7]. The 15 haplogroups can be subdivided into five major (A to E), three minor (F to H), and seven rare (I to K and W to Z) haplogroups [7], among which, we detected seven haplogroups (A to F and J) in indigenous Cambodian chickens. Haplogroups A and B are widely distributed in East Asia, and haplogroups C and D are frequently observed in Southeast Asia, with haplotype D being the most frequently detected in the red junglefowl [4,7]. In the present study, we identified haplogroups A, B, and D as being predominant types in Cambodian indigenous chickens, which is consistent with the genetic characteristics previously reported for indigenous chickens in Southeast Asia. Contrastingly, haplotype C was found to be rare in Cambodian indigenous chickens. Although haplogroup D has also been established to be predominant in The Philippines and some Pacific islands; however, the haplotypes of haplogroup D found in Cambodian indigenous chickens were found to differ from those of The Philippines and Pacific island clades. Notably, haplogroup E, which is the predominant type in domestic chickens, was found at low frequencies in Cambodian indigenous chickens. The frequency of haplogroup E is also low in indigenous chickens in Laos, Vietnam, and Myanmar [7,18,41,42], although it is frequently distributed among indigenous chickens in Thailand (10.9% and 23.2%) [43,44] and Bangladesh (33.3%) [45]. Haplogroup E is believed to have originated in India and is widely distributed in Western countries [7], which tends to indicate that it has not spread extensively in Southeast Asia. We found the minor haplogroup F to be rare in Cambodian indigenous chickens, being detected only in Kandal Province; however, this haplogroup is also found in indigenous chickens in China [4,7,46], India [7], Laos [7,42,47], Vietnam [7,48], Thailand [44], and Myanmar [4,7,49], and in the red junglefowl in China [4,7], Myanmar [4], Thailand [2], and Cambodia [47]. These findings would therefore appear to indicate that at the mtDNA level, Cambodian indigenous chickens may have retained genetic characteristics that are typical of indigenous chickens in continental Southeast Asia. However, a notable finding of this study is that haplogroup J occurs at a relatively high frequency in Cambodian indigenous chickens. This haplogroup is widely distributed in red junglefowl in Thailand [44], whereas it is comparatively rare in domestic chickens and has only been reported in a limited number of indigenous chickens in Indonesia [50] and Thailand [44]. These results thus indicate that indigenous Cambodian chickens may retain many of the haplotypes derived from ancestral wild populations.

In a number of previous studies, the genetic diversity of indigenous chickens in Southeast Asia has been investigated using microsatellite DNA markers [18,20,44,51]. For example, Cuc et al [18] examined the genetic diversity of nine indigenous chicken breeds in Vietnam and found that the mean number of alleles per locus (*Na*), observed heterozygosity (*Ho*), and expected heterozygosity (*He*) were 6.09, 0.60, and 0.63, respectively. *Ho* and *He* values have also been found to be higher than 0.60 in three breeds from Indonesia (0.63 to 0.66 and 0.65 to 0.72, respectively) and four breeds from Laos (0.62 to 0.69 and 0.64 to 0.68, respectively) [20] but were less than 0.60 in nine Thai indigenous chicken breeds (averaging 0.56 and 0.57, respectively) [44]. In 28 village populations of Cambodian indigenous chickens examined in the present study using 23 microsatellite markers, we obtained *Ho* and *He* values higher than 0.60 (both 0.62 on average), which is comparable to the values obtained for indigenous chickens in Vietnam, Indonesia, and Laos, although *Na* was lower than 6.00 (5.43). These findings accordingly indicate that in common with indigenous chickens in other Southeast Asian countries, indigenous chickens in Cambodia have high genetic diversity.

Population genetic analysis using 23 microsatellite markers revealed the presence of three major genetic clusters in Cambodian indigenous chickens, one of which is distributed in almost all regions of Cambodia surveyed, whereas the other two clusters are predominant in the northern part and the central and southern parts of Cambodia. The geographical distribution of these three clusters is conceivably attributable to inter-regional trade and transportation routes of poultry in Cambodia or between Cambodia and other countries [52]. An interesting finding in the present study is that one cluster is restricted to Chamkar Ou village in Pursat and Preybanlek village in Kampong Thom, which are located in the central part of Cambodia, and that the three-way hybrid chicken breed was grouped in the same genetic cluster. These findings tend to indicate that the hybrid chicken breed may have been established by crossing commercial chickens with indigenous chickens derived from these regions. To further establish the phylogenetic position of Cambodian indigenous chickens from the standpoint of chicken gene pools in Southeast Asia, it will be necessary to conduct additional comparative genome-wide analyses of indigenous chickens from Cambodia and neighboring countries, as well as population genetic studies using DNA markers.

## CONCLUSION

This study is the first population genetic study on the genetic diversity and population genetic structure of Cambodian indigenous chickens. Present data show that the high genetic diversity is conserved in Cambodian indigenous chickens and many of the mtDNA D-loop haplotypes that were derived from the ancestral wild populations are retained. Further large-scale genomic analyses of Cambodian indigenous chickens should be conducted to conserve Cambodian indigenous chickens as an important resource that has unique and beneficial genetic characteristics for developing novel chicken breeds with high quality and productivity.

## Figures and Tables

**Table 1:** Locations of sample collection and the genetic diversity of 28 populations of indigenous chickens and three commercial chicken breeds in Cambodia

Type of chickens	Population No.	Province	Village / Commune / District	N	mt DNA D-loop sequence^1)^				Microsatellite^2)^						
	
					n	*h*	*π*	*Theta-W*	n	*AR*	*Na*	*Ne*	*Ho*	*He*	*F*
Indigeous chicken	1	Oddar Meanchey	Doun Kaen village, Sangkat Samraong commune, Krong Samraong district	25	25	11	0.006	5.826	25	4.149	6.174	3.348	0.599	0.640	0.072
	2	Banteay Meanchey	Klengpor, Sla Kram, Svay Chek	25	22	6	0.005	4.938	25	3.478	4.957	2.676	0.632	0.573	−0.097
	3	Siem Reap	Preshdak, Preah Dak, Banteay Srei	25	23	6	0.007	5.961	24	4.033	5.522	3.112	0.635	0.622	−0.012
	4-1	Preah Vihear	Kandal, Sangkat Kampong Pranak, Krong Preah Vihear	13	11	5	0.005	7.170	13	4.129	5.217	3.082	0.635	0.618	−0.010
	4-2		Stapo, Sangkat Pal Hal, Krong Preah Vihear	12	11	6	0.007	8.194	12	4.118	4.870	3.206	0.635	0.620	−0.045
	5	Pailin	Toulslorlaov, Sangkat Toul Lvea, Krong Pailin	25	22	11	0.004	6.309	25	4.326	6.478	3.374	0.630	0.655	0.040
	6	Battambang	Toul Ta Aek, Sangkat Toul Ta Aek, Krong Battambang	25	25	8	0.006	5.562	20	3.789	4.783	3.096	0.583	0.614	0.052
	7-1	Pursat	Chamkar Ou, Trapeang Chorng, Bakan	19	18	3	0.006	4.361	18	3.006	3.478	2.320	0.548	0.511	−0.073
	7-2		Krobaochrum, Bak Chenhchien, Phnum Kravanh	25	19	7	0.006	6.581	25	3.680	4.826	3.053	0.667	0.622	−0.065
	7-3		Pramaoy, Pramaoy, Veal Veaeng	25	20	8	0.007	7.610	23	3.702	4.957	2.971	0.637	0.615	−0.022
	8	Kampong Thom	Preybanlek, Sangkat Achar Leak, Krong Steungsen	25	20	9	0.006	7.047	23	3.899	5.913	3.009	0.618	0.608	−0.016
	9	Kampong Chhnang	Troping Sbaov, Srae Thmei, Rolea B’ier	25	22	12	0.005	7.955	23	4.103	6.130	3.217	0.607	0.632	0.050
	10	Kampong Cham	Kaohdach, Kaoh Mitt, Kampong Siem	25	24	12	0.007	6.962	25	4.276	6.783	3.397	0.657	0.657	0.000
	11	Tboung Khmum	Chamkar Kor, Chak, Ou Reang Ov	25	25	7	0.004	4.502	25	4.056	5.565	3.316	0.645	0.641	−0.007
	12	Koh Kong	Toul Kokir Leu, Toul Kokir, Mondol Seima	25	21	6	0.007	6.115	25	4.129	6.087	3.309	0.648	0.656	0.023
	13-1	Kampong Speu	Chamkadoung, Sangkat Chbar Mon, Krong Chbar Mon	11	11	6	0.003	5.121	11	3.386	4.000	2.460	0.590	0.545	−0.061
	13-2		Pungro, Sangkat Kandaol Dom, Krong Chbar Mon	14	13	6	0.008	8.056	14	3.802	4.870	2.886	0.596	0.586	−0.023
	14	Kandal	Ta Kdol, Sangkat Ta Kdol, Krong Ta Khmau	25	25	14	0.006	7.415	25	4.156	6.348	3.421	0.621	0.658	0.060
	15	Prey Veng	Taket, Preah Sdach, Preah Sdach	25	22	3	0.004	3.014	25	3.711	4.870	3.021	0.652	0.624	−0.045
	16	Svay Rieng	Traok, Kampong Chamlang, Svay Chrum	25	25	14	0.006	7.415	25	3.609	4.870	2.879	0.612	0.596	−0.026
	17	Preah Sihanouk	Troping Sruy, Andoung Thma, Prey Nub	25	23	10	0.006	5.690	25	3.891	5.652	3.148	0.613	0.633	0.039
	18	Kampot	Breal, Tani, Angkor Chey	25	23	7	0.005	5.148	21	3.671	4.652	3.147	0.598	0.595	−0.015
	19	Kep	Chamkachek, Pong Tuek, Damnak Chang’aeur	25	24	10	0.007	7.766	20	3.978	5.478	3.090	0.633	0.620	−0.007
	20	Takeo	Troping Thom, Roneam, Trang	25	25	7	0.006	6.091	25	4.091	6.261	3.191	0.578	0.629	0.081
	21	Stung Treng	Reacheanukhul, Sangkat Stung Treng, Stung Treng Municipality	25	23	9	0.006	7.315	25	4.359	6.696	3.562	0.671	0.666	−0.010
	22	Ratanakiri	Pruok, Ba Tang, Lumphat	25	17	5	0.005	4.733	25	4.015	5.261	3.134	0.540	0.627	0.156
	23	Kratie	Dar, Dar, Chetr Borei	25	23	6	0.002	4.877	25	3.934	5.174	3.178	0.619	0.623	0.006
	24	Mondul Kiri	Polung, Sangkat Romonea, Senmonorom Municipality	27	23	7	0.003	4.064	26	4.298	6.130	3.638	0.636	0.652	0.026
	
		Mean				7.893	0.006	6.136		3.921	5.429	3.116	0.619	0.619	0.003

Commercial chicken	25	Phnom Penh	Three-way hybrid	9	9	6	0.007	6.991	9	3.790	4.043	2.717	0.634	0.586	−0.087
	26	Phnom Penh	Isa Brown	29	25	4	0.002	1.324	28	3.978	5.478	3.547	0.630	0.666	0.069
	27	Kandal	Rhode Island White (Thum, Kokir, Kien Svay)	6	6	2	0.000	0.438	6	3.304	3.174	2.533	0.612	0.543	−0.118

1) N, total number of individuals; *n*, number of individuals examined; *h*, number of observed haplotypes; *π*, nucleotide diversity; *Theta-W*, Watterson estimator (Theta-W per sequence in Dnasp).

2) *AR*, allelic richness; *Na*, mean number of alleles per locus; *Ne*, number of effective alleles frequencies = 1 / (Sum pi^2); *He*, expected heterozygosity; *Ho*, observed heterozygosity; *F*, fixation index = (*He* − *Ho*)/*He*.

1),2) The values of genetic diversity indices which are higher than the mean values are underlined.

**Table 2:** Frequencies of mitochondrial DNA D-loop haplogroups in Cambodian indigenous chickens and commercial chicken breeds

Type of chickens	Province	Population No.	No. of individuals	Haplogroup						

				A	B	C	D	E	F	J
Indigenous chicken	Oddar Meanchey	1	25	6	7		10			2
	Banteay Meanchey	2	22	1	16		5			
	Siem Reap	3	23	6	4		9			4
	Preah Vihear	4-1	11	1	1		7			2
		4-2	11	2	2		5			2
	Pailin	5	22	3	1		16			2
	Battambang	6	25	3	15		2	5		
	Pursat	7-1	18	5			4			9
		7-2	19	1	4		9			5
		7-3	20	5	2		7	4		2
	Kampong Thom	8	20	7	3		6	3		1
	Kampong Chhnang	9	22	3	1	1	13	1		3
	Kampong Cham	10	24	8	4		7			5
	Tboung Khmum	11	25	9	1		15			
	Koh Kong	12	21	1		4	9			7
	Kampong Speu	13-1	11	1			9	1		
		13-2	13	3			3		5	2
	Kandal	14	25	8	1		14			2
	Prey Veng	15	22	9			11	1		1
	Svay Rieng	16	25	1			14			10
	Preah Sihanouk	17	23	13	1		5			4
	Kampot	18	23	14	1		8			
	Kep	19	24	1	6		12	1		4
	Takeo	20	25	3	2		13			7
	Stung Treng	21	23		4	3	7	8		1
	Ratanakiri	22	17	5			2			10
	Kratie	23	23	1	1		18	3		
	Mondul Kiri	24	23	3			20			
	subtotal (average paercentage)			123 (21.0%)	77 (13.2%)	8 (1.4%)	260 (44.4%)	27 (4.6%)	5 (0.9%)	85 (14.5%)
Commercial chicken breed										
Three-way hybrid		25	9	2	3	0	1	3	0	0
Isa Brown		26	25	0	0	0	0	25	0	0
Road Island Red		27	6	0	0	0	0	6	0	0

## SUPPLEMENTARY MATERIAL

**Supplementary Table S1.** Primer sequences of microsatellite markers and the values of genetic diversity indices **Supplementary Table S2.** List of mitochondrial DNA D-loop sequences obtained from the GenBank database that were used for constructing the Bayesian phylogenetic tree **Supplementary Table S3.** Nucleotide sequences of mitochondrial DNA D-loop haplotypes obtained from the GenBank database that were used for constructing the Bayesian phylogenetic tree **Supplementary Table S4.** List of mitochondrial DNA D-loop halotypes found in Cambodian indigenous chickens and commercial chicken breeds **Supplementary Table S5.** Nucleotide sequences of mitochondrial DNA D-loop halotypes found in Cambodian indigenous chickens and commercial chicken breeds
