# Supplementary material for: Genetic diversity and population genetic structure of Cambodian indigenous chickens
Source: Anim Biosci. 2022 Jan 5;35(6):826–37. doi: 10.5713/ab.21.0351 (PMC9066038; doi:10.5713/ab.21.0351)
Supplement: Supplementary file 1 [file ab-21-0351-suppl.pdf]

Supplementary Table S1. Primer sequences of microsatellite markers and the values of genetic diversity indices

Marker	Chromosome	Primer sequence		Index [1)]						
		Forward 5′>3′	Reverse 3′>5′	*A*	*AR*	*Na*	*NAF*	F_{IS}	F_{ST}	F_{IT}
ADL0268	1	CTCCACCCCTCTCAGAACTA	CAACTTCCCATCTACCTACT	8	3.832	4.563	−0,007	−0.127	0.110	−0.003
MCW0111	1	GCTCCATGTGAAGTGGTTTA	GCTCCATGTGAAGTGGTTTA	15	5.085	5.375	0.026	−0.063	0.098	0.041
MCW0247	1	GTTGTTCAAAAGAAGATGCATG	TTGCATTAACTGGGCACTTTC	7	4.039	4.438	0.016	−0.047	0.123	0.081
MCW0034	2	ATCTTGAAACCTCACAAAGC	TCTTCCAACCTATTTTTAGT	21	6.498	6.719	0.127	−0.068	0.221	0.168
MCW0206	2	CTTGACAGTGATGCATTAAATG	ACATCTAGAATTGACTGTTCAC	20	4.877	5.594	0.052	0.022	0.137	0.155
LEI0166	3	CTCCTGCCCTTAGCTACGCA	TATCCCCTGGCTGGGAGTTT	14	3.917	4.469	0.013	−0.082	0.092	0.018
MCW0222	3	GCAGTTACATTGAAATGATTCC	TTCTCAAAACACCTAGAAGAC	4	2.877	3.125	0.108	0.048	0.091	0.135
MCW0016	3	ATGGCGCAGAAGGCAAAGCGATAT	TGGCTTCTGAAGCAGTTGCTATGG	23	6.421	7.063	0.025	−0.067	0.108	0.048
MCW0037	3	ACCGGTGCCATCAATTACCTATTA	GAAAGCTCACATGACACTGCGAAA	7	4.242	4.813	0.042	−0.002	0.102	0.101
MCW0103	3	AACTGCGTTGAGAGTGAATGC	TTTCCTAACTGGATGCTTCTG	4	2.062	2.063	0.054	0.036	0.105	0.137
MCW0295	4	ATCACTACAGAACACCCTCTC	TATGTATGCACGCAGATATCC	15	5.661	6.563	0.038	−0.037	0.094	0.061
LEI0094	4	GATCTCACCAGTATGAGCTGC	TCTCACACTGTAACACAGTGC	23	7.452	8.813	0.052	−0.006	0.109	0.103
MCW0078	5	CCACACGGAGAGGAGAAGGTCT	TAGCATATGAGTGTACTGAGCTTC	8	3.464	3.438	0.072	−0.015	0.151	0.139
MCW0098	4	GGCTGCTTTGTGCTCTTCTCG	CGATGGTCGTAATTCTCACGT	4	2.279	2.469	0.023	−0.023	0.084	0.063
MCW0081	5	GTTGCTGAGAGCCTGGTGCAG	CCTGTATGTGGAATTACTTCTC	18	4.068	4.031	0.155	0.031	0.268	0.290
MCW0183	7	ATCCCAGTGTCGAGTATCCGA	TGAGATTTACTGGAGCCTGCC	25	5.970	6.969	0.136	0.000	0.199	0.199
ADL0278	8	CCAGCAGTCTACCTTCCTAT	TGTCATCCAAGAACAGTGTG	18	4.758	5.844	0.004	−0.106	0.083	−0.014
MCW0067	10	GCACTACTGTGTGCTGCAGTTT	GAGATGTAGTTGCCACATTCCGAC	10	4.899	4.844	0.096	0.055	0.169	0.215
MCW0216	13	GGGTTTTACAGGATGGGACG	AGTTTCACTCCCAGGGCTCG	9	4.210	4.969	0.048	−0.020	0.088	0.070
MCW0104	13	TAGCACAACTCAAGCTGTGAG	AGACTTGCACAGCTGTGACC	32	7.173	8.688	0.085	−0.034	0.185	0.157
MCW0123	14	CCACTAGAAAAGAACATCCTC	GGCTGATGTAAGAAGGGATGA	13	5.476	6.156	0.040	−0.035	0.110	0.079
MCW0330	17	TGGACCTCATCAGTCTGACAG	AATGTTCTCATAGAGTTCCTGC	19	4.909	6.000	0.061	0.008	0.103	0.111
MCW0165	23	CAGACATGCATGCCCAGATGA	GATCCAGTCCTGCAGGCTGC	4	2.442	2.531	0.192	0.170	0.155	0.298

1) *A*: number of alleles, *AR*: allelic richness, *Na*: mean number of alleles per locus, *NAF*: null allele frequency, $F_{IS} = 1 – (H_I/H_S)$ frequency, $F_{ST} = 1 – (H_S/H_T)$ frequency and $F_{IT} = 1 – (H_I/H_T)$ frequency, H_I: averaged observed heterozygosity within populations, H_S:averaged expected heterozygosity within populations, H_T: expected heterozygosity for all populations

Supplementary Table S2. List of mitochondrial DNA D-loop sequences obtained from the GenBank database that were used for constructing the Bayesian phylogenetic tree

Accession No.	Haplotype name[1)]	Region	Country	Haplogroup
AB007720	DBAB007720_Indonesia_HapK	Southeast Asia	Indonesia	K
AB086102	DBAB086102_Japan_Hap_A	East Asia	Japan	A
AB268545	DBAB268545_Indonesia_HapJ	Southeast Asia	Indonesia	J
AP003317	DBAP003317_Japan_Hap_E1	East Asia	Japan	E
AP003318	DBAP003318_Japan_Hap_E1a	East Asia	Japan	E
AP003580	DBAP003580_Japan_Hap_E1	East Asia	Japan	E
AY235570	DBAY235570_USA_Hap_E1	North America	USA	E
AY235571	DBAY235571_USA_Hap_E1c	North America	USA	E
CM008858	DBCM008858_Korea_Hap_E1	East Asia	Korea	E
DQ648776	DBDQ648776_China_Hap_F3	East Asia	China	F
GU261674	DBGU261674_China_Hap_Z	East Asia	China	Z
GU261675	DBGU261675_China_Hap_C1a	East Asia	China	C
GU261676	DBGU261676_China_Hap_G1	East Asia	China	G
GU261677	DBGU261677_China_Hap_D3b	East Asia	China	D
GU261678	DBGU261678_China_Hap_G2	East Asia	China	G
GU261679	DBGU261679_China_Hap_C1b	East Asia	China	C
GU261680	DBGU261680_India_Hap_C2	South Asia	India	C
GU261681	DBGU261681_China_Hap_C1a	East Asia	China	C
GU261682	DBGU261682_Laos_Hap_D1	Southeast Asia	Laos	D
GU261683	DBGU261683_China_Hap_D2	East Asia	China	D
GU261684	DBGU261684_China_Hap_A1a	East Asia	China	A
GU261685	DBGU261685_India_Hap_D3a	South Asia	India	D
GU261686	DBGU261686_China_Hap_E1	East Asia	China	E
GU261687	DBGU261687_Laos_Hap_D1a2	Southeast Asia	Laos	D
GU261688	DBGU261688_China_Hap_F4	East Asia	China	F
GU261689	DBGU261689_China_Hap_F2	East Asia	China	F
GU261690	DBGU261690_China_Hap_G1a	East Asia	China	G
GU261691	DBGU261691_Myanmar_Hap_F1	Southeast Asia	Myanmar	F
GU261692	DBGU261692_China_Hap_X	East Asia	China	X
GU261693	DBGU261693_China_Hap_Y	East Asia	China	Y
GU261694	DBGU261694_China_Hap_E1b	East Asia	China	E
GU261695	DBGU261695_China_Hap_A1	East Asia	China	A
GU261696	DBGU261696_China_Hap_Z	East Asia	China	Z
GU261697	DBGU261697_India_Hap_D3a	South Asia	India	D
GU261698	DBGU261698_India_Hap_I	South Asia	India	I
GU261699	DBGU261699_China_Hap_B1a	East Asia	China	B
GU261700	DBGU261700_Myanmar_Hap_A1a	Southeast Asia	Myanmar	A
GU261701	DBGU261701_China_Hap_C1c	East Asia	China	C
GU261702	DBGU261702_China_Hap_F1	East Asia	China	F
GU261703	DBGU261703_Myanmar_Hap_F2	Southeast Asia	Myanmar	F
GU261704	DBGU261704_Myanmar_Hap_B1	Southeast Asia	Myanmar	B
GU261705	DBGU261705_China_Hap_B1a	East Asia	China	B
GU261706	DBGU261706_China_Hap_W1	East Asia	China	W
GU261707	DBGU261707_India_Hap_C3	South Asia	India	C
GU261708	DBGU261708_India_Hap_E3b	South Asia	India	E
GU261709	DBGU261709_India_Hap_E1	South Asia	India	E
GU261710	DBGU261710_China_Hap_G1	East Asia	China	G
GU261711	DBGU261711_China_Hap_F5	East Asia	China	F
GU261712	DBGU261712_China_Hap_E1a	East Asia	China	E
GU261713	DBGU261713_China_Hap_E1	East Asia	China	E
GU261714	DBGU261714_China_Hap_B1a	East Asia	China	B
GU261715	DBGU261715_China_Hap_H	East Asia	China	H
GU261716	DBGU261716_Myanmar_Hap_C3	Southeast Asia	Myanmar	C
GU261717	DBGU261717_China_Hap_F3	East Asia	China	F
GU261718	DBGU261718_China_Hap_C1b	East Asia	China	C

GU261719	DBGU261719_China_Hap_G1a	East Asia	China	G
HQ857209	DBHQ857209_India_Hap_E2	South Asia	India	E
HQ857210	DBHQ857210_India_Hap_E1c	South Asia	India	E
HQ857211	DBHQ857211_India_Hap_E3a	South Asia	India	E
HQ857212	DBHQ857212_India_Hap_E3a	South Asia	India	E
KF826490	DBKF826490_China_Hap_E1c	East Asia	China	E
KF939304	DBKF939304_China_Hap_D1	East Asia	China	D
KF954727	DBKF954727_China_Hap_E1a	East Asia	China	E
KF981434	DBKF981434_China_Hap_A	East Asia	China	A
KJ778617	DBKJ778617_China_Hap_A	East Asia	China	A
KM433666	DBKM433666_China_Hap_B1a	East Asia	China	B
KM886936	DBKM886936_China_Hap_A	East Asia	China	A
KM886937	DBKM886937_China_Hap_B	East Asia	China	B
KM096864	DBKM096864_China_Hap_B1a	East Asia	China	B
KP211418	DBKP211418_India_Hap_E	South Asia	India	E
KP211419	DBKP211419_India_Hap_E3b	South Asia	India	E
KP211420	DBKP211420_India_Hap_E2	South Asia	India	E
KP211421	DBKP211421_India_Hap_E1a	South Asia	India	E
KP211422	DBKP211422_India_Hap_I	South Asia	India	I
KP211423	DBKP211423_India_Hap_E1a	South Asia	India	E
KP211424	DBKP211424_India_Hap_G1a	South Asia	India	G
KP211425	DBKP211425_India_Hap_E1	South Asia	India	E
KP244335	DBKP244335_China_Hap_E1	East Asia	China	E
KP269069	DBKP269069_China_Hap_C1b	East Asia	China	C
KP681580	DBKP681580_China_Hap_C1b	East Asia	China	C
KP681581	DBKP681581_China_Hap_B1a	East Asia	China	B
KP742951	DBKP742951_China_Hap_B	East Asia	China	B
KR347464	DBKR347464_China_Hap_E1	East Asia	China	E
KT283576	DBKT283576_China_Hap_C3	East Asia	China	C
KX512321	DBKX512321_China_Hap_C1	East Asia	China	C
KX781318	DBKX781318_China_Hap_E1a	East Asia	China	E
KX781319	DBKX781319_China_Hap_A	East Asia	China	A
KX987152	DBKX987152_China_Hap_B	East Asia	China	B
KY039381	DBKY039381_Marquesas_Hap_D1a	Pacific area	Marquesas	D
KY039382	DBKY039382_Niue_Hap_D1	Pacific area	Niue	D
KY039383	DBKY039383_Hawaii_Hap_D1a	Pacific area	Hawaii	D
KY039384	DBKY039384_Hawaii_Hap_D1a	Pacific area	Hawaii	D
KY039385	DBKY039385_Hawaii_Hap_D1a	Pacific area	Hawaii	D
KY039386	DBKY039386_Easter Island_Hap_D1a	Pacific area	Easter Island	D
KY039387	DBKY039387_Easter Island_Hap_D1a	Pacific area	Easter Island	D
KY039388	DBKY039388_Easter Island_Hap_D1a	Pacific area	Easter Island	D
KY039389	DBKY039389_Easter Island_Hap_D1a	Pacific area	Easter Island	D
KY039390	DBKY039390_Easter Island_Hap_D1a	Pacific area	Easter Island	D
KY039391	DBKY039391_Fiji_Hap_D1a	Pacific area	Fiji	D
KY039392	DBKY039392_Vanuatu_Hap_D1a	Pacific area	Vanuatu	D
KY039393	DBKY039393_Vanuatu_Hap_D1a	Pacific area	Vanuatu	D
KY039394	DBKY039394_Papua New Guinea_Hap_D1	Pacific area	Papua New Guinea	D
KY039395	DBKY039395_Indonesia_Hap_D1	Southeast Asia	Indonesia	D
KY039396	DBKY039396_Philippines_Hap_D1a	Southeast Asia	Philippines	D
KY039397	DBKY039397_Philippines_Hap_D1	Southeast Asia	Philippines	D
KY039398	DBKY039398_Philippines_Hap_D1	Southeast Asia	Philippines	D
KY039399	DBKY039399_Philippines_Hap_D1	Southeast Asia	Philippines	D
KY039400	DBKY039400_Philippines_Hap_D1a	Southeast Asia	Philippines	D
KY039401	DBKY039401_Philippines_Hap_D1	Southeast Asia	Philippines	D
KY039402	DBKY039402_Philippines_Hap_D1	Southeast Asia	Philippines	D
KY039403	DBKY039403_Philippines_Hap_D1	Southeast Asia	Philippines	D
KY039404	DBKY039404_Philippines_Hap_D1a	Southeast Asia	Philippines	D
KY039405	DBKY039405_Philippines_Hap_D1a	Southeast Asia	Philippines	D

KY039406	DBKY039406_Philippines_Hap_D1	Southeast Asia	Philippines	D
KY039407	DBKY039407_Philippines_Hap_D1a	Southeast Asia	Philippines	D
KY039408	DBKY039408_Philippines_Hap_D1a	Southeast Asia	Philippines	D
KY039409	DBKY039409_Philippines_Hap_D1	Southeast Asia	Philippines	D
KY039410	DBKY039410_Philippines_Hap_D1a	Southeast Asia	Philippines	D
KY039411	DBKY039411_Philippines_Hap_D1a	Southeast Asia	Philippines	D
KY039412	DBKY039412_Philippines_Hap_D1a	Southeast Asia	Philippines	D
KY039413	DBKY039413_Philippines_Hap_D1a	Southeast Asia	Philippines	D
KY039414	DBKY039414_Philippines_Hap_D1	Southeast Asia	Philippines	D
KY039415	DBKY039415_Philippines_Hap_D1a	Southeast Asia	Philippines	D
KY039416	DBKY039416_Philippines_Hap_D1a	Southeast Asia	Philippines	D
KY039417	DBKY039417_Philippines_Hap_D1a	Southeast Asia	Philippines	D
KY039418	DBKY039418_Indonesia_Hap_D1	Southeast Asia	Indonesia	D
KY039419	DBKY039419_Indonesia_Hap_D1	Southeast Asia	Indonesia	D
KY039420	DBKY039420_Indonesia_Hap_D1	Southeast Asia	Indonesia	D
KY039421	DBKY039421_Indonesia_Hap_D1	Southeast Asia	Indonesia	D
KY039422	DBKY039422_Indonesia_Hap_D1a	Southeast Asia	Indonesia	D
KY039423	DBKY039423_Indonesia_Hap_D1	Southeast Asia	Indonesia	D
KY039424	DBKY039424_Indonesia_Hap_D1	Southeast Asia	Indonesia	D
KY039425	DBKY039425_Indonesia_Hap_D1a	Southeast Asia	Indonesia	D
KY039426	DBKY039426_Indonesia_Hap_D1a	Southeast Asia	Indonesia	D
KY039427	DBKY039427_Indonesia_Hap_D1	Southeast Asia	Indonesia	D
KY039428	DBKY039428_Indonesia_Hap_D1	Southeast Asia	Indonesia	D
KY039429	DBKY039429_Indonesia_Hap_D1	Southeast Asia	Indonesia	D
KY039430	DBKY039430_Vietnam_Hap_D3	Southeast Asia	Vietnam	D
KY039431	DBKY039431_New Caledonia_Hap_D1a	Pacific area	New Caledonia	D
KY039432	DBKY039432_New Caledonia_Hap_D1a	Pacific area	New Caledonia	D
KY039433	DBKY039433_China_Hap_Z	East Asia	China	Z
KY039434	DBKY039434_China_Hap_D1a	East Asia	China	D
KY039435	DBKY039435_Malacca_Hap_D1	Pacific area	Malacca	D
KY039436	DBKY039436_China_Hap_D1	East Asia	China	D
KY039437	DBKY039437_Philippines_Hap_D1a	Southeast Asia	Philippines	D
KY054997	DBKY054997_China_Hap_A	East Asia	China	A
MG605671	DBMG605671_Thailand_Hap_X	Southeast Asia	Thailand	X
MG837547	DBMG837547_China_Hap_E1	East Asia	China	E
MG837548	DBMG837548_China_Hap_E1	East Asia	China	E
MH732978	DBMH732978_China_Hap_B	East Asia	China	B
MH879470	DBMH879470_China_Hap_B	East Asia	China	B
MK163559	DBMK163559_China_Hap_D3	East Asia	China	D
MK163560	DBMK163560_China_Hap_A	East Asia	China	A
MK163561	DBMK163561_China_Hap_A	East Asia	China	A
MK163562	DBMK163562_China_Hap_D3	East Asia	China	D
MK163563	DBMK163563_China_Hap_E1	East Asia	China	E
MK163564	DBMK163564_China_Hap_B1a	East Asia	China	B
MK163565	DBMK163565_China_Hap_B	East Asia	China	B
MN013407	DBMN013407_China_Hap_E1	East Asia	China	E
MT555046	DBMT555046_China_Hap_C3	East Asia	China	C
MT555047	DBMT555047_China_Hap_C3	East Asia	China	C
MT555048	DBMT555048_China_Hap_C3	East Asia	China	C
MT555049	DBMT555049_China_Hap_C3	East Asia	China	C
NC040902	DBNC040902_Myanmar_Hap_C3	Southeast Asia	Myanmar	C
NC040970	DBNC040970_China_Hap_E1	East Asia	China	E
NC007239	DBNC007239 (G_lafayetii)	South Asia	Sri Lanka	

1) Haplotype names shown in the Bayesian phylogenetic tree in Figure 2.

Supplementary Table S3. Nucleotide sequences of mitochondrial DNA D-loop haplotypes obtained from the GenBank dartabase that were used for constructing the Bayesian phylogenetic tree

Accession No.	Haplotype name	Nucleotide sequence
AB007720	DBAB007720_Indonesia_HapK	ATTTTATTTTTTAACCTAACTCCCCTACTAAGTGTACCCCCCCTTTCCCCCCCAGGGGGGGTATACTATGCATAATCGTGCATACATTTATATACCACATATATTATGGT<br>ACCGGTAATATATACTATATATGTACTAAACCCATTATATGTATACGGGCATTAATCTATATTCCACATTTCTCCCAATGTCCATTCTATGCATGATCCAAGACATACTC<br>ATTCACCTTCCCCATAGATAGCTCCAAACCACTACCAAGACACCTAACTATGAATGGTTACAGGACATAAATCTCACTCTCATGCTCTTCCCCCAACAAGTCACCTAAC<br>TATGAATGGTTACAGGACATATACTTAACTACC--<br>ATGTTCTAACCCATTTGGTTATGCTCGTCGTATCAGATGGATTTATTGATCGTTCACCTCACGAGAGATCAGCAACCCCTGCTCGTAATGTACTTCATGACCAGTCTCA<br>GGCCCATTCTTTCCCCCTACACCCCTCGCCCTACTTGCCTTCCACCGTACCTCTGGTTCCTCGGTCAGGCACATCCCATGCATAACTCCTGAACTTTCTCACTTTTCAC<br>GAAGTCATCTGTGGATTATCTTCCCCTCTTTAGTCCGTGATCGCGGCATCTTCTCTCTTCTATTGCTGTTGGTTCCTTCTCTTTTTGGGGCTTCTTCACAGGTTGCCCTT<br>CACAGTGCGGGTGCGGAGTGCTATTCAAGTGAAGCCTGGACTACACCTGCGTTGCGTCCTATCCTAGTCCTCTCGTGTCCCTCGATGAGACGGTTTGCGTGTATGGGG<br>AATCATCTTGACACTGATGCACTTTGGATCGCATTTGGTTATGGTTCTTCCACCCCCCCC-<br>GGTAAATGGTGCTATTTAGTGAATGCTTGTCGGACATATTTTTATCAATTTTCACTTCCTCTATTTTCTTCACAAAACTAGGAAATTCACCACAATTTTTTCTTTGTTATT<br>TTTTAATTTTTTTTTTATTTTTTAAAAACATTTTTTAAAAAACTAAATTACATACAAACTACCGCATAAAATCCCTCAAACTATACAAACGTTTATCGTGTAATGTATATAC<br>ATTATTGTTTATTCTATCATTATTAGAGAAACTCCACTACCAAAACCATCATTAAAACAAAAATTTACATGCCACTTAACTCCCCTCACAAACAATCGTTATTTATATTGT<br>TAATTAGCAAACACAAAACCCGC
AB086102	DBAB086102_Japan_Hap_A	ATTTTATTTTTTAACCTAACTCCCCTACTAAGTGTACCCCCCCTTTCCCCCCCAGGGGGGGTATACTATGCATAATCGTGCATACATTTATATACCACATATATTATGGT<br>ACCGGTAATATATACTATATATGTACTAAACCCATTATATGTATACGGGCATTAACCTATATTCCACATTTCTCCCAATGTCCATTCTATGCATGATCCAGGACATACTC<br>ATTTACCCTCCCCATAGACAGTTCCAAACCACTATCAAGCCACCTAACTATGAATGGTTACAGGACATAAATCTCACTCTCATGTTCTCCCCCCAACAAGTCACCTAAC<br>TATGAATGGTTACAGGACATACATCTAACTACC--<br>ATGTTCTAACCCATTTGGTTATGCTCGCCGTATCAGATGGATTTATTGATCGTCCACCTCACGAGAGATCAGCAACCCCTGCCTGTAATGTACTTCATGACCAGTCTCA<br>GGCCCATTCTTTCCCCCTACACCCCTCGCCCTACTTGCCTTCCACCGTACCTCTGGTTCCTCGGTCAGGCACATCCCATGCATAACTCCTGAACTTTCTCACTTTTCAC<br>GAAGTCATCTGTGGATTATCTTCCCCTCTTTAGTCCGTGATCGCGGCATCTTCTCTCTTCTATTGCTGTTGGTTCCTTCTCTTTTTGGGGCTTCTTCACAGGTTACCCTT<br>CACAGTGCGGGTGCGGAGTGCTATTCAAGTGAAGCCTGGACTACACCTGCGTTGCGTCCTATCCTAGTCCTCTCGTGTCCCTCGATGAGACGGTTTGCGTGTATGGGG<br>AATCATCTTGACACTGATGCACTTTGGATCGCATTTGGTTATGGTTCTTCCACCCCCCC--<br>GGTAAATGGTGCTATTTAGTGAATGCTTGTCGGACATATTTTTATCAATTTTCACTTCCTCTATTTTCTTCACAAAACTAGGAAATTCACCACAATTTTTTCTTTGTTATT<br>TTTTAATTTTTTTTTTATTTTTTAAAAACATTTTTTAAAAAACTAAATTACATACAAACTACCGCATAAAATCCCTCAAACTATACAAACGTTTATCGTATAATATATATAC<br>ATTATTGTTTATTCTATCATTATTAGAGAAACTCCACTACCAAAACCATCATTAAAACAAAAATTTACATGCCACTTAACTCCCCTCACAAACAATCGTTATTTATATTGT<br>TAATTAGCAAACACAAAACCCGC
AB268545	DBAB268545_Indonesia_HapJ	ATTTTATTTTTTAACCTAACTCCCCTACTAAGTGTACCCCCCCTTTCCCCCCCAGGGGGGGTATACTATGCATAATCGTGCATACATTTATATACCACATATATTATGGT<br>ACCGGTAATATATACTATATATGTACTAAACCCATTATATGTATACGGGCATTAATCTATATTCCACATTTCTCCCAATGTCCATTCTATGCATGATCCAAGACATACTC<br>ATTCACTCTCCCCATGGACAGCTCTAAGCCACTACCAAGCCACCTAACTATGAATGGTTGCAGGACATAAATCTCACTCTCATGTTCTTCCCCCAACAAGTCACCTAAC<br>TATGAATGGTTACAGGACATACATCTAACTACT--<br>ATGTTCTAACCCATTTGGTTATGCTCGTCGTATCAGATGGATTTATTGATCGTCCACCTCACGAGAGATCAGCAACCCCTGCCTGTAATGTACTTCATGACCAGTCTCA<br>GGCCCATTCTTTCCCCCTACACCCCTCGCCCTACTTGCCTTCCACCGTACCTCTGGTTCCTCGGTCAGGCACATCCCATGCATAACTCCTGAACTTTCTCACTTTTCAC<br>GAAGTCATCTGTGGATTATCTTCCCCTCTTTAGTCCGTGATCGCGGCATCTTCTCTCTTCTATTGCTGTTGGTTCCTTCTCTTTTTGGGGCTTCTTCACAGGTTACCCTT<br>CACAGTGCGGGTGCGGAGTGCTATTCAAGTGAAGCCTGGACTACACCTGCGTTGCGTCCTATCCTAGTCCTCTCGTGTCCCTCGATGAGACGGTTTGCGTGTATGGGG<br>AATCATCTTGACACTGATGCACTTTGGATCGCATTTGGTTATGGTTCTTCCACCCCCCCC-<br>GGTAAATGGTGCTATTTAGTGAATGCTTGTCGGACATATTTTTATCAATTTTCACTTCCTCTATTTTCTTCACAAAACTAGGAAATTCACCACAATTTTTTCTTTGTTATT<br>TTTTAATTTTTTTTTTATTTTTTAAAAACATTTTTTAAAAAACTAAATTACATACAAACTACCGCATAAAATCCCTCAAACTATACAAACGTTTATCGTATAATATATATAC<br>ATTATTGTTTATTCTATCATTATTAGAGAAACTCCACTACCAAAACCATCATTAAAACAAAAATTTACATGCCACTTAACTCCCCTCACAAACAATCGTTATTTATATTGT<br>TAATTAGCAAACACAAAACCCGC

AP003317	DBAP003317_Japan_Hap_E1	ATTTTATTTTTTAACCTAACTCCCCTACTAAGTGTACCCCCCCTTTCCCCCCCAGGGGGGGTATACTATGCATAATCGTGCATACATTTATATACCACATATATTATGGT<br>ACCGGTAATATATACTATATATGTACTAAACCCATTATATGTATACGGGCATTAATCTATATTCCACATTTCTCCCAATGTCCATTCTATGCATGATCCAGGACACACTC<br>ATTCACCCTCCCCATAGACAGCTCCAAACCACTACCAAGTCACCTAACTATGAATGGTTACAGGACATAAATCTCACTCTCATGTTCTTCCCCCAACAAGTCACCTAAC<br>TATGAATGGTTACAGGACATACATTTAACTACC---<br>ATGTTCTAACCCATTTGGTTATGCTCGCCGTATCAGATGGATTTATTGATCGTCCACCTCACGAGAGATCAGCAACCCCTGCTTGTAATGTACTTCATGACCAGTCTCA<br>GGCCCATTCTTTCCCCCTACACCCCTCGCCCTACTTGCCTTCCACCGTACCTCTGGTTCCTCGGTCAGGCACATCCCATGCATAACTCCTGAACTTTCTCACTTTTCAC<br>GAAGTCATCTGTGGATTATCTTCCCCTCTTTAGTCCGTGATCGCGGCATCTTCTCTCTTCTATTGCTGTTGGTTCCTTCTCTTTTTGGGGCTTCTTCACAGGTTGCCCTT<br>CACAGTGCGGGTGCGGAGTGCTATTCAAGTGAAGCCTGGACTACACCTGCGTTGCGTCCTATCCTAGTCCTCTCGTGTCCCTCGATGAGACGGTTTGCGTGTATGGGG<br>AATCATCTTGACACTGATGCACTTTGGATCGCATTTGGTTATGGTTCTTCCACCCCCCC--<br>GGTAAATGGTGCTATTTAGTGAATGCTTGTCGGACATATTTTTATCAATTTTCACTTCCTCTATTTTCTTCACAAAACTAGGAAATTCACCACAATTTTTTCTTTGTTATT<br>TTTTAATTTTTTTTTTATTTTTTAAAAACATTTTTTAAAAAACTAAATTACATACAAACTACCGCATAAAATCCCTCAAACTATACAAACGTTTATCGTATAATATATATAC<br>ATTATTGTTTATTCTATCATTATTAGAGAAACTCCACTACCAAAACCATCATTAAAACAAAAATTTACATGCCACTTAACTCCCCTCACAAACAATCGTTATTTATATTGT<br>TAATTAGCAAACACAAAACCTGC
AP003318	DBAP003318_Japan_Hap_E1a	ATTTTATTTTTTAACCTAACTCCCCTACTAAGTGTACCCCCCCTTTCCCCCCCAGGGGGGGTATACTATGCATAATCGTGCATACATTTATATACCACATATATTATGGT<br>ACCGGTAATATATACTATATATGTACTAAACCCATTATATGTATACGGGCATTAATCTATATTCCACATTTCTCCCAATGTCCATTCTATGCATGATCCAGGACACACTC<br>ATTCACCCTCCCCATAGACAGCTCCAAACCACTACCAAGTCACCTAACTATGAATGGTTACAGGACATAAATCTCACTCTCATGTTCTTCCCCCAACAAGTCACCTAAC<br>TATGAATGGTTACAGGACATACATTTAACTACC---<br>ATGTTCTAACCCATTTGGTTATGCTCGCCGTATCAGATGGATTTATTGATCGTCCACCTCACGAGAGATCAGCAACCCCTGCTTGTAATGTACTTCATGACCAGTCTCA<br>GGCCCATTCTTTCCCCCTACACCCCTCGCCCTACTTGCCTTCCACCGTACCTCTGGTTCCTCGGTCAGGCACATCCCATGCATAACTCCTGAACTTTCTCACTTTTCAC<br>GAAGTCATCTGTGGATTATCTTCCCCTCTTTAGTCCGTGATCGCGGCATCTTCTCTCTTCTATTGCTGTTGGTTCCTTCTCTTTTTGGGGCTTCTTCACAGGTTGCCCTT<br>CACAGTGCGGGTGCGGAGTGCTATTCAAGTGAAGCCTGGACTACACCTGCGTTGCGTCCTATCCTAGTCCTCTCGTGTCCCTCGATGAGACGGTTTGCGTGTATGGGG<br>AATCATCTTGACACTGATGCACTTTGGATCGCATTTGGTTATGGTTCTTCCACCCCCCC--<br>GGTAAATGGTGCTATTTAGTGAATGCTTGTCGGACATATTTTTATCAATTTTCACTTCCTCTATTTTCTTCACAAAACTAGGAAATTCACCACAATTTTTTCTTTGTTATT<br>TTTTAATTTTTTTTTTATTTTTTAAAAACATTTTTTAAAAAACTAAATTACATACAAACTACCGCATAAAATCCCTCAAACTATACAAACGTTTATCGTATAATATATATAC<br>ATTATTGTTTATTCTATCATTATTAGAGAAACTCCACTACCAAAACCATCATTAAAACAAAAATTTACATGCCACTTAACTCCCCTCACAAACAATCGTTATTTATATTGT<br>TAATTAGCAAACACAAAACCTGC
AP003580	DBAP003580_Japan_Hap_E1	ATTTTATTTTTTAACCTAACTCCCCTACTAAGTGTACCCCCCCTTTCCCCCCCAGGGGGGGTATACTATGCATAATCGTGCATACATTTATATACCACATATATTATGGT<br>ACCGGTAATATATACTATATATGTACTAAACCCATTATATGTATACGGGCATTAATCTATATTCCACATTTCTCCCAATGTCCATTCTATGCATGATCCAGGACACACTC<br>ATTCACCCTCCCCATAGACAGCTCCAAACCACTACCAAGTCACCTAACTATGAATGGTTACAGGACATAAATCTCACTCTCATGTTCTTCCCCCAACAAGTCACCTAAC<br>TATGAATGGTTACAGGACATACATTTAACTACC---<br>ATGTTCTAACCCATTTGGTTATGCTCGCCGTATCAGATGGATTTATTGATCGTCCACCTCACGAGAGATCAGCAACCCCTGCTTGTAATGTACTTCATGACCAGTCTCA<br>GGCCCATTCTTTCCCCCTACACCCCTCGCCCTACTTGCCTTCCACCGTACCTCTGGTTCCTCGGTCAGGCACATCCCATGCATAACTCCTGAACTTTCTCACTTTTCAC<br>GAAGTCATCTGTGGATTATCTTCCCCTCTTTAGTCCGTGATCGCGGCATCTTCTCTCTTCTATTGCTGTTGGTTCCTTCTCTTTTTGGGGCTTCTTCACAGGTTGCCCTT<br>CACAGTGCGGGTGCGGAGTGCTATTCAAGTGAAGCCTGGACTACACCTGCGTTGCGTCCTATCCTAGTCCTCTCGTGTCCCTCGATGAGACGGTTTGCGTGTATGGGG<br>AATCATCTTGACACTGATGCACTTTGGATCGCATTTGGTTATGGTTCTTCCACCCCCCCC-<br>GGTAAATGGTGCTATTTAGTGAATGCTTGTCGGACATATTTTTATCAATTTTCACTTCCTCTATTTTCTTCACAAAACTAGGAAATTCACCACAATTTTTTCTTTGTTATT<br>TTTTAATTTTTTTTTTATTTTTTAAAAACATTTTTTAAAAAACTAAATTACATACAAACTACCGCATAAAATCCCTCAAACTATACAAACGTTTATCGTATAATATATATAC<br>ATTATTGTTTATTCTATCATTATTAGAGAAACTCCACTACCAAAACCATCATTAAAACAAAAATTTACATGCCACTTAACTCCCCTCACAAACAATCGTTATTTATATTGT<br>TAATTAGCAAACACAAAACCTGC

AY235570	DBAY235570_USA_Hap_E1	ATTTTATTTTTTAACCTAACTCCCCTACTAAGTGTACCCCCCCTTTCCCCCCCAGGGGGGGTATACTATGCATAATCGTGCATACATTTATATACCACATATATTATGGT<br>ACCGGTAATATATACTATATATGTACTAAACCCATTATATGTATACGGGCATTAATCTATATTCCACATTTCTCCCAATGTCCATTCTATGCATGATCCAGGACACACTC<br>ATTCACCCTCCCCATAGACAGCTCCAAACCACTACCAAGTCACCTAACTATGAATGGTTACAGGACATAAATCTCACTCTCATGTTCTTCCCCCAACAAGTCACCTAAC<br>TATGAATGGTTACAGGACATACATTTAACTACC--<br>ATGTTCTAACCCATTTGGTTATGCTCGCCGTATCAGATGGATTTATTGATCGTCCACCTCACGAGAGATCAGCAACCCCTGCTTGTAATGTACTTCATGACCAGTCTCA<br>GGCCCATTCTTTCCCCCTACACCCCTCGCCCTACTTGCCTTCCACCGTACCTCTGGTTCCTCGGTCAGGCACATCCCATGCATAACTCCTGAACTTTCTCACTTTTCAC<br>GAAGTCATCTGTGGATTATCTTCCCCTCTTTAGTCCGTGATCGCGGCATCTTCTCTCTTCTATTGCTGTTGGTTCCTTCTCTTTTTGGGGCTTCTTCACAGGTTGCCCTT<br>CACAGTGCGGGTGCGGAGTGCTATTCAAGTGAAGCCTGGACTACACCTGCGTTGCGTCCTATCCTAGTCCTCTCGTGTCCCTCGATGAGACGGTTTGCGTGTATGGGG<br>AATCATCTTGACACTGATGCACTTTGGATCGCATTTGGTTATGGTTCTTCCACCCCCCCC-<br>GGTAAATGGTGCTATTTAGTGAATGCTTGTCGGACATATTTTTATCAATTTTCACTTCCTCTATTTTCTTCACAAAACTAGGAAATTCACCACAATTTTTTCTTTGTTATT<br>TTTTAATTTTTTTTTTATTTTTTAAAAACATTTTTTAAAAAACTAAATTACATACAAACTACCGCATAAAATCCCTCAAACTATACAAACGTTTATCGTATAATATATATAC<br>ATTATTGTTTATTCTATCATTATTAGAGAAACTCCACTACCAAAACCATCATTAAAACAAAAATTTACATGCCACTTAACTCCCCTCACAAACAATCGTTATTTATATTGT<br>TAATTAGCAAACACAAAACCTGC
AY235571	DBAY235571_USA_Hap_E1c	ATTTTATTTTTTAACCTAACTCCCCTACTAAGTGTACCCCCCCTTTCCCCCCCAGGGGGGGTATACTATGCATAATCGTGCATACATTTATATACCACATATATTATGGT<br>ACCGGTAATATATACTATATATGTACTAAACCCATTATATGTATACGGGCATTAATCTATATTCCACATTTCTCCCAATGTCCATTCTATGCATGATCCAGGACACACTC<br>GTTCACCCTCCCCATAGACAGCTCCAAACCACTACCAAGTCACCTAACTATGAATGGTTACAGGACATAAATCTCACTCTCATGTTCTTCCCCCAACAAGTCACCTAAT<br>TATGAATGGTTACAGGACATACATTTAACTACC--<br>ATGTTCTAACCCATTTGGTTATGCTCGCCGTATCAGATGGATTTATTGATCGTCCACCTCACGAGAGATCAGCAACCCCTGCTTGTAATGTACTTCATGACCAGTCTCA<br>GGCCCATTCTTTCCCCCTACACCCCTCGCCCTACTTGCCTTCCACCGTACCTCTGGTTCCTCGGTCAGGCACATCCCATGCATAACTCCTGAACTTTCTCACTTTTCAC<br>GAAGTCATCTGTGGATTATCTTCCCCTCTTTAGTCCGTGATCGCGGCATCTTCTCTCTTCTATTGCTGTTGGTTCCTTCTCTTTTTGGGGCTTCTTCACAGGTTGCCCTT<br>CACAGTGCGGGTGCGGAGTGCTATTCAAGTGAAGCCTGGACTACACCTGCGTTGCGTCCTATCCTAGTCCTCTCGTGTCCCTCGATGAGACGGTTTGCGTGTATGGGG<br>AATCATCTTGACACTGATGCACTTTGGATCGCATTTGGTTATGGTTCTTCCACCCCCCC--<br>GGTAAATGGTGCTATTTAGTGAATGCTTGTCGGACATATTTTTATCAATTTTCACTTCCTCTATTTTCTTCACAAAACTAGGAAATTCACCACAATTTTTTCTTTGTTATT<br>TTTTAATTTTTTTTTTATTTTTTAAAAACATTTTTTAAAAAACTAAATTACATACAAACTACCGCATAAAATCCCTCAAACTATACAAACGTTTATCGTATAATATATATAC<br>ATTATTGTTTATTCTATCATTATTAGAGAAACTCCACTACCAAAACCATCATTAAAACAAAAATTTACATGCCACTTAACTCCCCTCACAAACAATCGTTATTTATATTGT<br>TAATTAGCAAACACAAAACCTGC
CM008858	DBCM008858_Korea_Hap_E1	ATTTTATTTTTTAACCTAACTCCCCTACTAAGTGTACCCCCCCTTTCCCCCCCAGGGGGGGTATACTATGCATAATCGTGCATACATTTATATACCACATATATTATGGT<br>ACCGGTAATATATACTATATATGTACTAAACCCATTATATGTATACGGGCATTAATCTATATTCCACATTTCTCCCAATGTCCATTCCATGCATGATCCAGGACACACTC<br>ATTCACCCTCCCCATAGACAGCTCCAAACCACTACCAAGTCACCTAACTATGAATGGTTACAGGACATAAATCTCACTCTCATGTTCTTCCCCCAACAAGTCACCTAAC<br>TATGAATGGTTACAGGACATACATTTAACTACC--<br>ATGTTCTAACCCATTTGGTTATGCTCGCCGTATCAGATGGATTTATTGATCGTCCACCTCACGAGAGATCAGCAACCCCTGCTTGTAATGTACTTCATGACCAGTCTCA<br>GGCCCATTCTTTCCCCCTACACCCCTCGCCCTACTTGCCTTCCACCGTACCTCTGGTTCCTCGGTCAGGCACATCCCATGCATAACTCCTGAACTTTCTCACTTTTCAC<br>GAAGTCATCTGTGGATTATCTTCCCCTCTTTAGTCCGTGATCGCGGCATCTTCTCTCTTCTATTGCTGTTGGTTCCTTCTCTTTTTGGGGCTTCTTCACAGGTTGCCCTT<br>CACAGTGCGGGTGCGGAGTGCTATTCAAGTGAAGCCTGGACTACACCTGCGTTGCGTCCTATCCTAGTCCTCTCGTGTCCCTCGATGAGACGGTTTGCGTGTATGGGG<br>AATCATCTTGACACTGATGCACTTTGGATCGCATTTGGTTATGGTTCTTCCACCCCCCC--<br>GGTAAATGGTGCTATTTAGTGAATGCTTGTCGGACATATTTTTATCAATTTTCACTTCCTCTATTTTCTTCACAAAACTAGGAAATTCACCACAATTTTTTCTTTGTTATT<br>TTTTAATTTTTTTTTTATTTTTTAAAAACATTTTTTAAAAAACTAAATTACATACAAACTACCGCATAAAATCCCTCAAACTATACAAACGTTTATCGTATAATATATATAC<br>ATTATTGTTTATTCTATCATTATTAGAGAAACTCCACTACCAAAACCATCATTAAAACAAAAATTTACATGCCACTTAACTCCCCTCACAAACAATCGTTATTTATATTGT<br>TAATTAGCAAACACAAAACCTGC

DQ648776	DBDQ648776_China_Hap_F3	ATTTTATTTTTTAACCTAACTCCCCTACTAAGTGTACCCCCCCTTTCCCCCCCAGGGGGGGTATACTATGCATAATCGTGCATACATTTATATACCACATATATTATGGT<br>ACCGGTAATATATACTATATATGTACTAAACCCATTATATGTATACGGGCATTAATCTATATTCCACATTTCTCCCAATGTCCATTCTATGCATGATCCAGGACATACTC<br>ATTCACCCTCCCTACAGACAGCTCTAAACCACCACCAAGTCACCTAACTATGAATGGTTACAGGACATAAATCTCACTCTCATGTTCTTCCCCTACCAAGTCACCTAAC<br>TATGAATGGTTACAGGACATACATTTAACTACC--<br>ATGTTCTAACCCATTTGGTTATGCTCGCCGTATCAGATGGATTTATTGATCGTCCACCTCACGAGAGATCAGCAACCCCTGCTTGTAATGTACTTCATGACCAGTCTCA<br>GGCCCATTCTTTCCCCCTACACCCCTCGCCCTACTTGCCTTCCACCGTACCTCTGGTTCCTCGGTCAGGCACATCCCATGCATAACTCCTGAACTTTCTCACTTTTCAC<br>GAAGTCATCTGTGGATTATCTTCCCCTCTTTAGTCCGTGATCGCGGCATCTTCTCTCTTCTATTGCTGTTGGTTCCTTCTCTTTTTGGGGCTTCTTCACAGGTTACCCTT<br>CACAGTGCGGGTGCGGAGTGCTATTCAAGTGAAGCCTGGACTACACCTGCGTTGCGTCCTATCCTAGTCCTCTCGTGTCCCTCGATGAGACGGTTTGCGTGTATGGGG<br>AATCATCTTGACACTGATGCACTTTGGATCGCATTTGGTTATGGTTCTTCCACCCCCCC--<br>GGTAAATGGTGCTATTTAGTGAATGCTTGTCGGACATATTTTTACCAATTTTCACTTCCTCTATTTTCTTCACAAAACTAGGAAATTCACCACAATTTTTTCTTTGTTATT<br>TTTTAATTTTTTTTTTATTTTTTAAAAACATTTTTTAAAAAACTAAATTACATACAAACTACCGCATAAAATCCCTCAAACTATACAAACGTTTATCGTATAATATATATAC<br>ATTATTGTTTATTCTATCATTATTAGAGAAACTCCACTACCAAAACCATCATTAAAACAAAAATTTACATGCCACTTAACTCCCCTCACAAACAATCGTTATTTATATTGT<br>TAATTAGCAAACACAAAACCTGC
GU261674	DBGU261674_China_Hap_Z	ATTTTATTTTTTAACCTAACTCCCCTACTAAGTGTACCCCCCCTTTCCCCCCCAGGGGGGGTATACTATGCATAATCGTGCATACATTTATATACCACATATATTATGGT<br>ACCGGTAATATATACTATATATGTACTAAACCCATTATATGTATACGGGCATTAATCTATATTCCACATTTCTCCCAATGTCCATTCTATGCATGATCCAGGACATACTC<br>ATTCACCCTCCCCATAGACAGTTCCAAACCACTACCAAGCCACCTAACTATGAATGGTTACAGGACATAAATCTCACTCTCATGCTCTTCCCCCAACAAGTCACCTAAC<br>TATGAATGGTTACAGGACATACATTTAACTACC--<br>ATGTTCTAACCCATTTGGTTATGCTCGACGTATCAGATGGATTTATTGATCGTCCACCTCACGAGAGATCAGCAACCCCTGCCTGTAATGTACTTCATGACCAGTCTCA<br>GGCCCATTCTTTCCCCCTACACCCCTCGCCCTACTTGCCTTCCACCGTACCTCTGGTTCCTCGGTCAGGCACATCCCATGCATAACTCCTGAACTTTCTCACTTTTCAC<br>GAAGTCATCTGTGGATTATCTTCCCCTCTTTAGTCCGTGATCGCGGCATCTTCTCTCTTCTATTGCTGTTGGTTCCTTCTCTTTTTGGGGCTTCTTCACAGGTTGCCCTT<br>CACAGTGCGGGTGCGGAGTGCTATTCAAGTGAAGCCTGGACTACACCTGCGTTGCGTCCTATCCTAGTCCTCTCGTGTCCCTCGATGAGACGGTTTGCGTGTATGGGG<br>AATCATCTTGACACTGATGCACTTTGGATCGCATTTGGTTATGGTTCTTCCACCCCCCC--<br>GGTAAATGGTGCTATTTAGTGAATGCTTGCCGGACATATTTTTATCAATTTTCACTTCCTCTATTTTCTTCACAAAACTAGGAAATTCACCACAATTTTTTCTTTGTTATT<br>TTTTAATTTTTTTTTTATTTTTTAAAAACATTTTTTAAAAAACTAAATTACATACAAACTACCGCATAAAATCCCTCAAACTATACAAACGTTTATCGTATAATATATATAC<br>ATTATTGTTTATTCTATCATTATTAGAGAAACTCCACTACCAAAACCATCATTAAAACAAAAATTTACATGCCACTTAACTCCCCTCACAAACAATCGTTATTTATATTGT<br>TAATTAGCAAACACAAAACCCGC
GU261675	DBGU261675_China_Hap_C1a	ATTTTATTTTTTAACCTAACTCCCCTACTAAGTGTACCCCCCCTTTCCCCCCCAGGGGGGGTATACTATGCATAATCGTGCATACATTTATATACCACATATATTATGGT<br>ACCGGTAATATATACTATATATGTACTAAACCCATTATATGTATACGGGCATTAATCTATATTCCACATTTCTCCCAATGTCCATTCTATGCATGATCCAGGACATACTC<br>ATTCACCCTCCCCATAGACAACTCCAAACCACTACCAAGCCACCTAACTATGAATGGTTGCAGGACATAAATCTCACTCTCATGTTCTTCCCCCAACAAGTCACCTAAC<br>TATGAATGGTTACAGGACATACATTTAACTACT--<br>ATGCTCTAACCCATTTGGTTATGCTCGCCGTATCAGATGGATTTATTGATCGTCCACCTCACGAGAGATCAGCAACCCCTGCCTGTAATGTACTTCATGACCAGTCTCA<br>GGCCCATTCTTTCCCCCTACACCCCTCGCCCTACTTGCCTTCCACCGTACCTCTGGTTCCTCGGTCAGGCACATCCCATGCATAACTCCTGAACTTTCTCACTTTTCAC<br>GAAGTCATCTGTGGATTATCTTCCCCTCTTTAGTCCGTGATCGCGGCATCTTCTCTCTTCTATTGCTGTTGGTTCCTTCTCTTTTTGGGGCTTCTTCACAGGTTGCCCTT<br>CACAGTGCGGGTGCGGAGTGCTATTCAAGTGAAGCCTGGACTACACCTGCGTTGCGTCCTATCCTAGTCCTCTCGTGTCCCTCGATGAGACGGTTTGCGTGTATGGGG<br>AATCATCTTGACACTGATGCACTTTGGATCGCATTTGGTTATGGTTCTTCCACCCCCCC--<br>GGTAAATGGTGCTATTTAGTGAATGCTTGTCGGACATATTTTTATCAATTTTCACTTCCTCTATTTTCTTCACAAAACTAGGAAATTCACCACAATTTTTTCTTTGTTATT<br>TTTTAATTTTTTTTTTATTTTTTAAAAACATTTTTTAAAAAACTAAATTACATACAAACTACCGCATAAAATCCCTCAAACTATACAAACGTTTATCGTATAATATATATAC<br>ATTATTGTTTATTCTATCATTATTAGAGAAACTCCACTACCAAAACCATCATTAAAACAAAAATTTACATGCCACTTAACTCCCCTCACAAACAATCGTTATTTATATTGT<br>TAATTAGCAAACACAAAACCCGC

GU261676	DBGU261676_China_Hap_G1	ATTTTATTTTTTAACCTAACTCCCCTACTAAGTGTACCCCCCCTTTCCCCCCCAGGGGGGGTATACTATGCATAATCGTGCATACATTTATATACCACATATATTATGGT<br>ACCGGTAATATATACTATATATGTACTAAACCCATTATATGTATACGGGCATTAATCTATATTCCACATTTCTCCCAATGTCCATTCTATGCATGATCCAAGACATACTC<br>ATTCACCCTCCCCATAGACAGCTCCAAACCACTACCAAGTCACCTAACTATGAATGGTTACAGGACATAAATCTAACTCTTATGTTCTTCCCCTAACAAGTCACCTAAC<br>TATGAATGGTTACAGGACATACATTTAACTACC--<br>ATGTTCTAACCCATTTGGTTATGCTCGCCGTATCAGATGGATTTATTGATCGTCCACCTCACGAGAGATCAGCAACCCCTGCTTGTAATGTACTTCATGACCAGTCTCA<br>GGCCCATTCTTTCCCCCTACACCCCTCGCCCTACTTGCCTTCCACCGTACCTCTGGTTCCTCGGTCAGGCACATCCCATGCATAACTCCTGAACTTTCTCACTTTTCAC<br>GAAGTCATCTGTGGATTATCTTCCCCTCTTTAGTCCGTGATCGCGGCATCTTCTCTCTTCTATTGCTGTTGGTTCCTTCTCTTTTTGGGGCTTCTTCACAGGTTGCCCTT<br>CACAGTGCGGGTGCGGAGTGCTATTCAAGTGAAGCCTGGACTACACCTGCGTTGCGTCCTATCCTAGTCCTCTCGTGTCCCTCGATGAGACGGTTTGCGTGTATGGGG<br>AATCATCTTGACACTGATGCACTTTGGATCGCATTTGGTTATGGTTCTTCCACCCCCCCC-<br>GGTAAATGGTGCTATTTAGTGAATGCTTGTCGGACATATTTTTATCAATTTTCACTTCCTCTATTTTCTTCACAAAACTAGGAAATTCACCACAATTTTTTCTTTGTTATT<br>TTTTAATTTTTTTTTTATTTTTTAAAAACATTTTTTAAAAAACTAAATTACATACAAACTACCGCATAAAATCCCTCAAACTATACAAACGTTTATCGTATAATATATATAC<br>ATTATTGTTTATTCTATCATTATTAGAGAAACTCCACTACCAAAACCATCATTAAAACAAAAATTTACATGCCACTTAACTCCCCTCACAAACAATCGTTATTTATATTGT<br>TAATTAGCAAACACAAAACCTGC
GU261677	DBGU261677_China_Hap_D3b	ATTTTATTTTTTAACCTAACTCCCCTACTAAGTGTACCCCCCCTTTCCCCCCCAGGGGGGGTATACTATGCATAATCGTGCATACATTTATATACCACATATATTATGGT<br>ACCGGTAATATATACTATATATGTACTAAACCCATTATATGTATACGGGCATTAATCTATATTCCACATTTCTCCCAATGTCCATTCTATGCATGATCCAGGACATACCC<br>ATTCACCCTCCCCATAGACAGCTCCAAACCACTACCAAGTCACCTAACTATGAATGGTTACAGGACATAAATCTCACTCTTATGCTCTTCCCCCAACAAGTCACCTAAC<br>TATGAATGGTTACAGGACATACATTTAACTACC--<br>ATGTTCTAACCCATTTGGTTATGCTCGACGTATCAGATGGATTTATTGATCGTCCACCTCACGAGAGATCAGCAACCCCTGCCTGTAATGTACTTCATGACCAGTCTCA<br>GGCCCATTCTTTCCCCCTACACCCCTCGCCCTACTTGCCTTCCACCGTACCTCTGGTTCCTCGGTCAGGCACATCCCATGCATAACTCCTGAACTTTCTCACTTTTCAC<br>GAAGTCATCTGTGGATTATCTTCCCCTCTTTAGTCCGTGATCGCGGCATCTTCTCTCTTCTATTGCTGTTGGTTCCTTCTCTTTTTGGGGCTTCTTCACAGGTTGCCCTT<br>CACAGTGCGGGTGCGGAGTGCTATTCAAGTGAAGCCTGGACTACACCTGCGTTGCGTCCTATCCTAGTCCTCTCGTGTCCCTCGATGAGACGGTTTGCGTGTATGGGG<br>AATCATCTTGACACTGATGCACTTTGGATCGCATTTGGTTATGGTTCTTCCACCCCCCC--<br>GGTAAATGGTGCTATTTAGTGAATGCTTGTCGGACATATTTTTATCAATTTTCACTTCCTCTATTTTCTTCACAAAACTAGGAAATTCACCACAATTTTTTCTTTGTTATT<br>TTTTAATTTTTTTTTTATTTTTTAAAAACATTTTTTAAAAAACTAAATTACATACAAACTACCGCATAAAATCCCTCAAACTATACAAACGTTTATCGTATAATATATATAC<br>ATTATTGTTTATTCTATCATTATTAGAGAAACTCCACTACCAAAACCATCATTAAAACAAAAATTTACATGCCACTTAACTCCCCTCACAAACAATCGTTATTTATATTGT<br>TAATTAGCAAACACAAAACCCGC
GU261678	DBGU261678_China_Hap_G2	ATTTTATTTTTTAACCTAACTCCCCTACTAAGTGTACCCCCCCTTTCCCCCCCAGGGGGGGTATACTATGCATAATCGTGCATACATTTATATACCACATATATTATGGT<br>ACCGGTAATATATACTATATATGTACTAAACCCATTATATGTATACGGGCATTAATCTATATTCCACATTTCTCCCAATGTCCATTCTATGCATGATCCAAGACATACTC<br>ATTCACCCTCCCCATAGACAGCTCCAAACCACTACCAAGTCACCTAACTATGAATGGTTACAGGACATAAATCTAACTCTTATGTTCTTCCCCTAACAAGCCACCTAAC<br>TATGAATGGTTACAGGGCATACATTTAACTACC--<br>ATGTTCTAACCCATTTGGTTATGCTCGCCGTATCAGATGGATTTATTGATCGTCCACCTCACGAGAGATCAGCAACCCCTGCTTGTAATGTACTTCATGACCAGTCTCA<br>GGCCCATTCTTTCCCCCTACACCCCTCGCCCTACTTGCCTTCCACCGTACCTCTGGTTCCTCGGTCAGGCACATCCCATGCATAACTCCTGAACTTTCTCACTTTTCAC<br>GAAGTCATCTGTGGATTATCTTCCCCTCTTTAGTCCGTGATCGCGGCATCTTCTCTCTTCTATTGCTGTTGGTTCCTTCTCTTTTTGGGGCTTCTTCACAGGTTGCCCTT<br>CACAGTGCGGGTGCGGAGTGCTATTCAAGTGAAGCCTGGACTACACCTGCGTTGCGTCCTATCCTAGTCCTCTCGTGTCCCTCGATGAGACGGTTTGCGTGTATGGGG<br>AATCATCTTGACACTGATGCACTTTGGATCGCATTTGGTTATGGTTCTTCCACCCCCCC--<br>GGTAAATGGTGCTATTTAGTGAATGCTTGTCGGACATATTTTTATCAATTTTCACTTCCTCTATTTTCTTCACAAAACTAGGAAATTCACCACAATTTTTTCTTTGTTATT<br>TTTTAATTTTTTTTTTATTTTTTAAAAACATTTTTTAAAAAACTAAATTACATACAAACTACCGCATAAAATCCCTCAAACTATACAAACGTTTATCGTATAATATATATAC<br>ATTATTGTTTATTCTATCATTATTAGAGAAACTCCACTACCAAAACCATCATTAAAACAAAAATTTACATGCCACTTAACTCCCCTCACAAACAATCGTTATTTATATTGT<br>TAATTAGCAAACACAAAACCTGC

GU261679	DBGU261679_China_Hap_C1b	ATTTTATTTTTTAACCTAACTCCCCTACTAAGTGTACCCCCCCTTTCCCCCCCAGGGGGGGTATACTATGCATAATCGTGCATACATTTATATACCACATATATTATGGT<br>ACCGGTAATATATACTATATATGTACTAAACCCATTATATGTATACGGGCATTAATCTATATTCCACATTTCTCCCAATGTCCATTCTATGCATGATCCAGGACATACTC<br>ATTCACCCTCCCCATAGACAACTCCAAACCACTACCAAGCCACCTAACTATGAATGGTTGCAGGACATAAATCTCACTCTCATGTTCTTCCCCCAACAAGTCACCTAAC<br>TATGAATGGTTGCAGGACATACATTTAACTACT--<br>ATGCTCTAACCCATTTGGTTATGCTCGTCGTATCAGATGGATTTATTGATCGTCCACCTCACGAGAGATCAGCAACCCCTGCCTGTAATGTACTTCATGACCAGTCTCA<br>GGCCCATTCTTTCCCCCTACACCCCTCGCCCTACTTGCCTTCCACCGTACCTCTGGTTCCTCGGTCAGGCACATCCCATGCATAACTCCTGAACTTTCTCACTTTTCAC<br>GAAGTCATCTGTGGATTATCTTCCCCTCTTTAGTCCGTGATCGCGGCATCTTCTCTCTTCTATTGCTGTTGGTTCCTTCTCTTTTTGGGGCTTCTTCACAGGTTGCCCTT<br>CACAGTGCGGGTGCGGAGTGCTATTCAAGTGAAGCCTGGACTACACCTGCGTTGCGTCCTATCCTAGTCCTCTCGTGTCCCTCGATGAGACGGTTTGCGTGTATGGGG<br>AATCATCTTGACACTGATGCACTTTGGATCGCATTTGGTTATGGTTCTTCCACCCCCCCC-<br>GGTAAATGGTGCTATTTAGTGAATGCTTGTCGGACATATTTTTATCAATTTTCACTTCCTCTATTTTCTTCACAAAACTAGGAAATTCACCACAATTTTTTCTTTGTTATT<br>TTTTAATTTTTTTTTTATTTTTTAAAAACATTTTTTAAAAAACTAAATTACATACAAACTACCGCATAAAATCCCTCAAACTATACAAACGTTTATCGTATAATATATATAC<br>ATTATTGTTTATTCTATCATTATTAGAGAAACTCCACTACCAAAACCATCATTAAAACAAAAATTTACATGCCACTTAACTCCCCTCACAAACAATCGTTATTTATATTGT<br>TAATTAGCAAACACAAAACCCGC
GU261680	DBGU261680_India_Hap_C2	ATTTTATTTTTTAACCTAACTCCCCTACTAAGTGTACCCCCCCTTTCCCCCCCAGGGGGGGTATACTATGCATAATCGTGCATACATTTATATACCACATATATTATGGT<br>ACCGGTAATATATACTATATATGTACTAAACCCATTATATGTATACGGGCATTAATCTATATTCCACATTTCTCCCAATGTCCATTCTATGCATGATCCAGGACATACTC<br>ATTCACCCTCCCCATAGACAGCTCCAAACCACTACCAAGTCACCTAACTATGAATGGTTACAGGACATAAATCTCACTCTCATGCTCTTCCCCCAACAAGTCACCTAAC<br>TATGAATGGTTGCAGGACATACATTTAACTATT--<br>ATGTTCTAACCCATTTGGTTATGCTCGCCGTATCAGATGGATTTATTGATCGTCCACCTCACGAGAGATCAGCAACCCCTGCCTGTAATGTACTTCATGACCAGTCTCA<br>GGCCCATTCTTTCCCCCTACACCCCTCGCCCTACTTGCCTTCCACCGTACCTCTGGTTCCTCGGTCAGGCACATCCCATGCATAACTCCTGAACTTTCTCACTTTTCAC<br>GAAGTCATCTGTGGATTATCTTCCCCTCTTTAGTCCGTGATCGCGGCATCTTCTCTCTTCTATTGCTGTTGGTTCCTTCTCTTTTTGGGGCTTCTTCACAGGTTGCCCTT<br>CACAGTGCGGGTGCGGAGTGCTATTCAAGTGAAGCCTGGACTACACCTGCGTTGCGTCCTATCCTAGTCCTCTCGTGTCCCTCGATGAGACGGTTTGCGTGTATGGGG<br>AATCATCTTGACACTGATGCACTTTGGATCGCATTTGGTTATGGTTCTTCCACCCCCCCC-<br>GGTAAATGGTGCTATTTAGTGAATGCTTGTCGGACATATTTTTATCAATTTTCACTTCCTCTATTTTCTTCACAAAACTAGGAAATTCACCACAATTTTTTCTTTGTTATT<br>TTTTAATTTTTTTTTTATTTTTTAAAAACATTTTTTAAAAAACTAAATTACATACAAACTACCGCATAAAATCCCTCAAACTATACAAACGTTTATCGTATAATATATATAC<br>ATTATTGTTTATTCTATCATTATTAGAGAAACTCCACTACCAAAACCATCATTAAAACAAAAATTTACATGCCACTTAACTCCCCTCACAAACAATCGTTATTTATATTGT<br>TAATTAGCAAACACAAAACCCGC
GU261681	DBGU261681_China_Hap_C1a	ATTTTATTTTTTAACCTAACTCCCCTACTAAGTGTACCCCCCCTTTCCCCCCCAGGGGGGGTATACTATGCATAATCGTGCATACATTTATATACCACATATATTATGGT<br>ACCGGTAATATATACTATATATGTACTAAACCCATTATATGTATACGGGCATTAATCTATATTCCACATTTCTCCCAATGTCCATTCTATGCATGATCCAGGACATACTC<br>ATTCACCCTCCCCATAGACAACTCCAGACCACTACCAAGCCACCTAACTATGAATGGTTACAGGACATAAATCTCACTCTCATGTTCTTCCCCCAACAAGTCACCTAAC<br>TATGAATGGTTGCAGGACATACATTTAACTACT--<br>ATGCTCTAACCCATTTGGTTATGCTCGCCGTATCAGATGGATTTATTGATCGTCCACCTCACGAGAGATCAGCAACCCCTGCCTGTAATGTACTTCATGACCAGTCTCA<br>GGCCCATTCTTTCCCCCTACACCCCTCGCCCTACTTGCCTTCCACCGTACCTCTGGTTCCTCGGTCAGGCACATCCCATGCATAACTCCTGAACTTTCTCACTTTTCAC<br>GAAGTCATCTGTGGATTATCTTCCCCTCTTTAGTCCGTGATCGCGGCATCTTCTCTCTTCTATTGCTGTTGGTTCCTTCTCTTTTTGGGGCTTCTTCACAGGTTGCCCTT<br>CACAGTGCGGGTGCGGAGTGCTATTCAAGTGAAGCCTGGACTACACCTGCGTTGCGTCCTATCCTAGTCCTCTCGTGTCCCTCGATGAGACGGTTTGCGTGTATGGGG<br>AATCATCTTGACACTGATGCACTTTGGATCGCATTTGGTTATGGTTCTTCCACCCCCCC--<br>GGTAAATGGTGCTATTTAGTGAATGCTTGTCGGACATATTTTTATCAATTTTCACTTCCTCTATTTTCTTCACAAAACTAGGAAATTCACCACAATTTTTTCTTTGTTATT<br>TTTTAATTTTTTTTTTATTTTTTAAAAACATTTTTTAAAAAACTAAATTACATACAAACTACCGCATAAAATCCCTCAAACTATACAAACGTTTATCGTATAATATATATAC<br>ATTATTGTTTATTCTATCATTATTAGAGAAACTCCACTACCAAAACCATCATTAAAACAAAAATTTACATGCCACTTAACTCCCCTCACAAACAATCGTTATTTATATTGT<br>TAATTAGCAAACACAAAACCCGC

GU261682	DBGU261682_Laos_Hap_D1	ATTTTATTTTTTAACCTAACTCCCCTACTAAGTGTACCCCCCCTTTCCCCCCCAGGGGGGGTATACTATGCATAATCGTGCATACATTTATATACCACATATATTATGGT<br>ACCGGTAATATATACTATATATGTACTAAACCCATTATATGTATACGGGCATTAATCTATATTCCACATTTCTCCCAATGTCCATTCTATGCATGATCCAGGACATACCC<br>ATTCACCCTCCCCATAGACAGCTCCAAACCACTACCAAGTCACCTAACTATGAATGGTTGCAGGACATAAATCTCACTCTCATGCTCTTCCCCCAACAAGTCACCTAAC<br>TATGAATGGTTACGGGACATACATTTAACTACC---<br>ATGTTCTAACCCATTTGGTTATGCTCGCCGTATCAGATGGATTTATTGATCGTCCACCTCACGAGAGATCAGCAACCCCTGCCTGTAATGTACTTCATGACCAGTCTCA<br>GGCCCATTCTTTCCCCCTACACCCCTCGCCCTACTTGCCTTCCACCGTACCTCTGGTTCCTCGGTCAGGCACATCCCATGCATAACTCCTGAACTTTCTCACTTTTCAC<br>GAAGTCATCTGTGGATTATCTTCCCCTCTTTAGTCCGTGATCGCGGCATCTTCTCTCTTCTATTGCTGTTGGTTCCTTCTCTTTTTGGGGCTTCTTCACAGGTTGCCCTT<br>CACAGTGCGGGTGCGGAGTGCTATTCAAGTGAAGCCTGGACTACACCTGCGTTGCGTCCTATCCTAGTCCTCTCGTGTCCCTCGATGAGACGGTTTGCGTGTATGGGG<br>AATCATCTTGACACTGATGCACTTTGGATCGCATTTGGTTATGGTTCTTCCACCCCCCC--<br>GGTAAATGGTGCTATTTAGTGAATGCTTGTCGGACATATTTTTATCAATTTTCACTTCCTCTATTTTCTTCACAAAACTAGGAAATTCACCACAATTTTTTCTTTGTTATT<br>TTTTAATTTTTTTTTTATTTTTTAAAAACATTTTTTAAAAAACTAAATTACATACAAACTACCGCATAAAATCCCTCAAACTATACAAACGTTTATCGTATAATATATATAC<br>ATTATTGTTTATTCTATCATTATTAGAGAAACTCCACTACCAAAACCATCATTAAAACAAAAATTTACATGCCACTTAACTCCCCTCACAAACAATCGTTATTTATATTGT<br>TAATTAGCAAACACAAAACCCGC
GU261683	DBGU261683_China_Hap_D2	ATTTTATTTTTTAACCTAACTCCCCTACTAAGTGTACCCCCCCTTTCCCCCCCAGGGGGGGTATACTATGCATAATCGTGCATACATTTATATACCACATATATTATGGT<br>ACCGGTAATATATACTATATATGTACTAAACCCATTATATGTATACGGGCATTAATCTATATTCCACATTTCTCCCAATGTCCATTCTATGCATGATCCAGGACATACTC<br>ATTCACCCTCCCCATAGACAGCTCCAAACCACTACCAAGTCACCTAACTATGAATGGTTGCAGGACATAAATCTCACTCTCATGCTCTTCCCCCAACAAGTCACCTAAC<br>TATGAATGGTTACAGGACATACATTTAACTACC---<br>ATGTTCTAACCCATTTGGTTATGCTCGCCGTATCAGATGGATTTATTGATCGTCCACCTCACGAGAGATCAGCAACCCCTGCCTGTAATGTACTTCATGACCAGTCTCA<br>GGCCCATTCTTTCCCCCTACACCCCTCGCCCTACTTGCCTTCCACCGTACCTCTGGTTCCTCGGTCAGGCACATCCCATGCATAACTCCTGAACTTTCTCACTTTTCAC<br>GAAGTCATCTGTGGATTATCTTCCCCTCTTTAGTCCGTGATCGCGGCATCTTCTCTCTTCTATTGCTGTTGGTTCCTTCTCTTTTTGGGGCTTCTTCACAGGTTGCCCTT<br>CACAGTGCGGGTGCGGAGTGCTATTCAAGTGAAGCCTGGACTACACCTGCGTTGCGTCCTATCCTAGTCCTCTCGTGTCCCTCGATGAGACGGTTTGCGTGTATGGGG<br>AATCATCTTGACACTGATGCACTTTGGATCGCATTTGGTTATGGTTCTTCCACCCCCCC--<br>GGTAAATGGTGCTATTTAGTGAATGCTTGTCGGACATATTTTTATCAATTTTCACTTCCTCTATTTTCTTCACAAAACTAGGAAATTCACCACAATTTTTTCTTTGTTATT<br>TTTTAATTTTTTTTTTATTTTTTAAAAACATTTTTTAAAAAACTAAATTACATACAAACTACCGCATAAAATCCCTCAAACTATACAAACGTTTATCGTATAATATATATAC<br>ATTATTGTTTATTCTATCATTATTAGAGAAACTCCACTACCAAAACCATCATTAAAACAAAAATTTACATGCCACTTAACTCCCCTCACAAACAATCGTTATTTATATTGT<br>TAATTAGCAAACACAAAACCCGC
GU261684	DBGU261684_China_Hap_A1a	ATTTTATTTTTTAACCTAACTCCCCTACTAAGTGTACCCCCCCTTTCCCCCCCAGGGGGGGTATACTATGCATAATCGTGCATACATTTATATACCACATATATTATGGT<br>ACCGGTAATATATACTATATATGTACTAAACCCATTATATGTATACGGGCATTAACCTATATTCCACATTTCTCCCAATGTCCATTCTATGCATGATCCAGGACATACTC<br>ATTTACCCTCCCCATAGACAGTTCCAAACCACTATCAAGCCACCTAACTATGAATGGTTACAGGACATAAATCTCACTCTCATGTTCTCCCCCCAACAAGTCACCTAAC<br>TATGAATGGTTACAGGACATACATTTAACTACC---<br>ATGTTCTAACCCATTTGGTTATGCTCGCCGTATCAGATGGATTTATTGATCGTCCACCTCACGAGAGATCAGCAACCCCTGCCTGTAATGTACTTCATGACCAGTCTCA<br>GGCCCATTCTTTCCCCCTACACCCCTCGCCCTACTTGCCTTCCACCGTACCTCTGGTTCCTCGGTCAGGCACATCCCATGCATAACTCCTGAACTTTCTCACTTTTCAC<br>GAAGTCATCTGTGGATTATCTTCCCCTCTTTAGTCCGTGATCGCGGCATCTTCTCTCTTCTATTGCTGTTGGTTCCTTCTCTTTTTGGGGCTTCTTCACAGGTTGCCCTT<br>CACAGTGCGGGTGCGGAGTGCTATTCAAGTGAAGCCTGGACTACACCTGCGTTGCGTCCTATCCTAGTCCTCTCGTGTCCCTCGATGAGACGGTTTGCGTGTATGGGG<br>AATCATCTTGACACTGATGCACTTTGGATCGCATTTGGTTATGGTTCTTCCACCCCCCC--<br>GGTAAATGGTGCTATTTAGTGAATGCTTGTCGGACATATTTTTATCAATTTTCACTTCCTCTATTTTCTTCACAAAACTAGGAAATTCACCACAATTTTTTCTTTGTTATT<br>TTTTAATTTTTTTTTTATTTTTTAAAAACATTTTTTAAAAAACTAAATTACATACAAACTACCGCATAAAATCCCTCAAACTATACAAACGTTTATCGTATAATATATATAC<br>ATTATTGTTTATTCTATCATTATTAGAGAAACTCCACTACCAAAACCATCATTAAAACAAAAATTTACATGCCACTTAACTCCCCTCACAAACAATCGTTATTTATATTGT<br>TAATTAGCAAACACAAAACCCGC

GU261685	DBGU261685_India_Hap_D3a	ATTTTATTTTTTAACCTAACTCCCCTACTAAGTGTACCCCCCCTTTCCCCCCCAGGGGGGGTATACTATGCATAATCGTGCATACATTTATATACCACATATATTATGGT<br>ACCGGTAATATATACTATATATGTACTAAACCCATTATATGTATACGGGCATTAATCTATATTCCACATTTCTCCCAATGTCCATTCTATGCATGATCTAGGACATACTT<br>ATTCACCCTCCCCATAGACAGCTCCAAACCACTACCAAGTCACCTAACTATGAATGGTTGCAGGACATAAATCTCACTCTCATGCTCTTCCCCCAACAAGTCACCTAAC<br>TATGAATGGTTGCAGGACATACATTTAACTACC---<br>ATGTTCTAACCCATTTGGTTATGCTCGCCGTATCAGATGGATTTATTGATCGTCCACCTCACGAGAGATCAGCAACCCCTGCCTGTAATGTACTTCATGACCAGTCTCA<br>GGCCCATTCTTTCCCCCTACACCCCTCGCCCTACTTGCCTTCCACCGTACCTCTGGTTCCTCGGTCAGGCACATCCCATGCATAACTCCTGAACTTTCTCACTTTTCAC<br>GAAGTCATCTGTGGATTATCTTCCCCTCTTTAGTCCGTGATCGCGGCATCTTCTCTCTTCTATTGCTGTTGGTTCCTTCTCTTTTTGGGGCTTCTTCACAGGTTGCCCTT<br>CACAGTGCGGGTGCGGAGTGCTATTCAAGTGAAGCCTGGACTACACCTGCGTTGCGTCCTATCCTAGTCCTCTCGTGTCCCTCGATGAGACGGTTTGCGTGTATGGGG<br>AATCATCTTGACACTGATGCACTTTGGATCGCATTTGGTTATGGTTCTTCCACCCCCCC--<br>GGTAAATGGTGCTATTTAGTGAATGCTTGTCGGACATATTTTTATCAATTTTCACTTCCTCTATTTTCTTCACAAAACTAGGAAATTCACCACAATTTTTTCTTTGTTATT<br>TTTTAATTTTTTTTTTATTTTTTAAAAACATTTTTTAAAAAACTAAATTACATACAAACTACCGCATAAAATCCCTCAAACTATACAAACGTTTATCGTATAATATATATAC<br>ATTATTGTTTATTCTATCATTATTAGAGAAACTCCACTACCAAAACCATCATTAAAACAAAAATTTACATGCCACTTAACTCCCCTCACAAACAATCGTTATTTATATTGT<br>TAATTAGCAAACACAAAACCCGC
GU261686	DBGU261686_China_Hap_E1	ATTTTATTTTTTAACCTAACTCCCCTACTAAGTGTACCCCCCCTTTCCCCCCCAGGGGGGGTATACTATGCATAATCGTGCATACATTTATATACCACATATATTATGGT<br>ACCGGTAATATATACTATATATGTACTAAACCCATTATATGTATACGGGCATTAATCTATATTCCACATTTCTCCCAATGTCCATTCTATGCATGATCCAGGACACACTC<br>ATTCACCCTCCCCATAGACAGCTCTAAACCACTACCAAGTCACCTAACTATGAATGGTTACAGGACATAAATCTCACTCTCATGTTCTTCCCCCAACAAGTCACCTAAC<br>TATGAATGGTTACAGGACATACATTTAACTACC---<br>ATGTTCTAACCCATTTGGTTATGCTCGCCGTATCAGATGGATTTATTGATCGTCCACCTCACGAGAGATCAGCAACCCCTGCTTGTAATGTACTTCATGACCAGTCTCA<br>GGCCCATTCTTTCCCCCTACACCCCTCGCCCTACTTGCCTTCCACCGTACCTCTGGTTCCTCGGTCAGGCACATCCCATGCATAACTCCTGAACTTTCTCACTTTTCAC<br>GAAGTCATCTGTGGATTATCTTCCCCTCTTTAGTCCGTGATCGCGGCATCTTCTCTCTTCTATTGCTGTTGGTTCCTTCTCTTTTTGGGGCTTCTTCACAGGTTGCCCTT<br>CACAGTGCGGGTGCGGAGTGCTATTCAAGTGAAGCCTGGACTACACCTGCGTTGCGTCCTATCCTAGTCCTCTCGTGTCCCTCGATGAGACGGTTTGCGTGTATGGGG<br>AATCATCTTGACACTGATGCACTTTGGATCGCATTTGGTTATGGTTCTTCCACCCCCCC--<br>GGTAAATGGTGCTATTTAGTGAATGCTTGTCGGACATATTTTTATCAATTTTCACTTCCTCTATTTTCTTCACAAAACTAGGAAATTCACCACAATTTTTTCTTTGTTATT<br>TTTTAATTTTTTTTTTATTTTTTAAAAACATTTTTTAAAAAACTAAATTACATACAAACTACCGCATAAAATCCCTCAAACTATACAAACGTTTATCGTATAATATATATAC<br>ATTATTGTTTATTCTATCATTATTAGAGAAACTCCACTACCAAAACCATCATTAAAACAAAAATTTACATGCCACTTAACTCCCCTCACAAACAATCGTTATTTATATTGT<br>TAATTAGCAAACACAAAACCTGC
GU261687	DBGU261687_Laos_Hap_D1a2	ATTTTATTTTTTAACCTAACTCCCCTACTAAGTGTACCCCCCCTTTCCCCCCCAGGGGGGGTATACTATGCATAATCGTGCATACATTTATATACCACATATATTATGGT<br>ACCGGTAATATATACTATATATGTACTAAACCCATTATATGTATACGGGCATTAATCTATATTCCACATTTCTCCCAATGTCCATTCTATGCATGATCCAGGACATACTC<br>ATTCACCCTCCCCATAGACAGCTCCAAACCACTACCAAGTCACCTAACTATGAATGGTTGCAGGACATAAATCTCACTCTCATGCTCTTCCCCCAACAAGTCACCTAAC<br>TATGAATGGTTGCAGGACATACATTTAACTACC---<br>ATGTTCTAACCCATTTGGTTATGCTCGCCGTATCAGATGGATTTATTGATCGTCCACCTCACGAGAGATCAGCAACCCCTGCCTGTAATGTACTTCATGACCAGTCTCA<br>GGCCCATTCTTTCCCCCTACACCCCTCGCCCTACTTGCCTTCCACCGTACCTCTGGTTCCTCGGTCAGGCACATCCCATGCATAACTCCTGAACTTTCTCACTTTTCAC<br>GAAGTCATCTGTGGATTATCTTCCCCTCTTTAGTCCGTGATCGCGGCATCTTCTCTCTTCTATTGCTGTTGGTTCCTTCTCTTTTTGGGGCTTCTTCACAGGTTGCCCTT<br>CACAGTGCGGGTGCGGAGTGCTATTCAAGTGAAGCCTGGACTACACCTGCGTTGCGTCCTATCCTAGTCCTCTCGTGTCCCTCGATGAGACGGTTTGCGTGTATGGGG<br>AATCATCTTGACACTGATGCACTTTGGATCGCATTTGGTTATGGTTCTTCCACCCCCCC--<br>GGTAAATGGTGCTATTTAGTGAATGCTTGTCGGACATATTTTTATCAATTTTCACTTCCTCTATTTTCTTCACAAAACTAGGAAATTCACCACAATTTTTTCTTTGTTATT<br>TTTTAATTTTTTTTTTATTTTTTAAAAACATTTTTTAAAAAACTAAATTACATACAAACTACCGCATAAAATCCCTCAAACTATACAAACGTTTATCGTATAATATATATAC<br>ATTATTGTTTATTCTATCATTATTAGAGAAACTCCACTACCAAAACCATCATTAAAACAAAAATTTACATGCCACTTAACTCCCCTCACAAACAATCGTTATTTATATTGT<br>TAATTAGCAAACACAAAACCCGC

GU261688	DBGU261688_China_Hap_F4	ATTTTATTTTTTAACCTAACTCCCCTACTAAGTGTACCCCCCCTTTCCCCCCCAGGGGGGGTATACTATGCATAATCGTGCATACATTTATATACCACATATATTATGGT<br>ACCGGTAATATATACTATATATGTACTAAACCCATTATATGTATACGGGCATTAATCTATATTCCACATTTCTCCCAATGTCCATTCTATGCATGATCCAGGACATACTC<br>ATTCACCCTCCCTACAGACAGCTCCAAACCACCACCAAGTCACTTAACTATGAATGGTTACAGGACATAAATCTCACTCTCATGTTCTTCCCCTACCAAGTCACCTAAC<br>TATGAATGGTTACAGGACATACATTTAACTACC---<br>ATGTTCTAACCCATTTGGTTATGCTCGCCGTATCAGATGGATTTATTGATCGTCCACCTCACGAGAGATCAGCAACCCCTGCTTGTAATGTACTTCATGACCAGTCTCA<br>GGCCCATTCTTTCCCCCTACACCCCTCGCCCTACTTGCCTTCCACCGTACCTCTGGTTCCTCGGTCAGGCACATCCCATGCATAACTCCTGAACTTTCTCACTTTTCAC<br>GAAGTCATCTGTGGATTATCTTCCCCTCTTTAGTCCGTGATCGCGGCATCTTCTCTCTTCTATTGCTGTTGGTTCCTTCTCTTTTTGGGGCTTCTTCACAGGTTGCCCTT<br>CACAGTGCGGGTGCGGAGTGCTATTCAAGTGAAGCCTGGACTACACCTGCGTTGCGTCCTATCCTAGTCCTCTCGTGTCCCTCGATGAGACGGTTTGCGTGTATGGGG<br>AATCATCTTGACACTGATGCACTTTGGATCGCATTTGGTTATGGTTCTTCCACCCCCCC--<br>GGTAAATGGTGCTATTTAGTGAATGCTTGTCGGACATATTTTTACCAATTTTCACTTCCTCTATTTTCTTCACAAAACTAGGAAATTCACCACAATTTTTTCTTTGTTATT<br>TTTTAATTTTTTTTTTATTTTTTAAAAACATTTTTTAAAAAACTAAATTACATACAAACTACCGCATAAAATCCCTCAAACTATACAAACGTTTATCGTATAATATATATAC<br>ATTATTGTTTATTCTATCATTATTAGAGAAACTCCACTACCAAAACCATCATTAAAACAAAAATTTACATGCCACTTAACTCCCCTCACAAACAATCGTTATTTATATTGT<br>TAATTAGCAAACACAAAACCTGC
GU261689	DBGU261689_China_Hap_F2	ATTTTATTTTTTAACCTAACTCCCCTACTAAGTGTACCCCCCCTTTCCCCCCCAGGGGGGGTATACTATGCATAATCGTGCATACATTTATATACCACATATATTATGGT<br>ACCGGTAATATATACTATATATGTACTAAACCCATTATATGTATACGGGCATTAATCTATATTCCACATTTCTCCCAATGTCCATTCTATGCATGATCCAGGACATACTC<br>ATTCACCCTCCCTACAGACAGCTCCAAACCACCACCAAGTCACCTAACTATGAATGGTTACAGGACATAAATCTCACTCTCATGTTCTTCCCCTACCAAGTCACCTAAC<br>TATGAATGGTTGCAGGACATACATTTAACTACC---<br>ATGTTCTAACCCATTTGGTTATGCTCGCCGTATCAGATGGATTTATTGATCGTCCACCTCACGAGAGATCAGCAACCCCTGCTTGTAATGTACTTCATGACCAGTCTCA<br>GGCCCATTCTTTCCCCCTACACCCCTCGCCCTACTTGCCTTCCACCGTACCTCTGGTTCCTCGGTCAGGCACATCCCATGCATAACTCCTGAACTTTCTCACTTTTCAC<br>GAAGTCATCTGTGGATTATCTTCCCCTCTTTAGTCCGTGATCGCGGCATCTTCTCTCTTCTATTGCTGTTGGTTCCTTCTCTTTTTGGGGCTTCTTCACAGGTTGCCCTT<br>CACAGTGCGGGTGCGGAGTGCTATTCAAGTGAAGCCTGGACTACACCTGCGTTGCGTCCTATCCTAGTCCTCTCGTGTCCCTCGATGAGACGGTTTGCGTGTATGGGG<br>AATCATCTTGACACTGATGCACTTTGGATCGCATTTGGTTATGGTTCTTCCACCCCCCC--<br>GGTAAATGGTGCTATTTAGTGAATGCTTGTCGGACATATTTTTACCAATTTTCACTTCCTCTATTTTCTTCACAAAACTAGGAAATTCACCACAATTTTTTCTTTGTTATT<br>TTTTAATTTTTTTTTTATTTTTTAAAAACATTTTTTAAAAAACTAAATTACATACAAACTACCGCATAAAATCCCTCAAACTATACAAACGTTTATCGTATAATATATATAC<br>ATTATTGTTTATTCTATCATTATTAGAGAAACTCCACTACCAAAACCATCATTAAAACAAAAATTTACATGCCACTTAACTCCCCTCACAAACAATCGTTATTTATATTGT<br>TAATTAGCAAACACAAAACCTGC
GU261690	DBGU261690_China_Hap_G1a	ATTTTATTTTTTAACCTAACTCCCCTACTAAGTGTACCCCCCCTTTCCCCCCCAGGGGGGGTATACTATGCATAATCGTGCATACATTTATATACCACATATATTATGGT<br>ACCGGTAATATATACTATATATGTACTAAACCCATTATATGTATACGGGCATTAATCTATATTCCACATTTCTCCCAATGTCCATTCTATGCATGATCCAAGACATACTC<br>ATTCACCCTCCCCATAGACAGCTCCAAACCACTACCAAGTCACCTAACTATGAATGGTTACAGGACATAAATCTAACTCTTATGTTCTTCCCCTAACAAGCCACCTAAC<br>TATGAATGGTTACAGGACATACATTTAACTATC---<br>ATGTTCTAACCCATTTGGTTATGCTCGCCGTATCAGATGGATTTATTGATCGTCCACCTCACGAGAGATCAGCAACCCCTGCTTGTAATGTACTTCATGACCAGTCTCA<br>GGCCCATTCTTTCCCCCTACACCCCTCGCCCTACTTGCCTTCCACCGTACCTCTGGTTCCTCGGTCAGGCACATCCCATGCATAACTCCTGAACTTTCTCACTTTTCAC<br>GAAGTCATCTGTGGATTATCTTCCCCTCTTTAGTCCGTGATCGCGGCATCTTCTCTCTTCTATTGCTGTTGGTTCCTTCTCTTTTTGGGGCTTCTTCACAGGTTGCCCTT<br>CACAGTGCGGGTGCGGAGTGCTATTCAAGTGAAGCCTGGACTACACCTGCGTTGCGTCCTATCCTAGTCCTCTCGTGTCCCTCGATGAGACGGTTTGCGTGTATGGGG<br>AATCATCTTGACACTGATGCACTTTGGATCGCATTTGGTTATGGTTCTTCCACCCCCCCC-<br>GGTAAATGGTGCTATTTAGTGAATGCTTGTCGGACATATTTTTATCAATTTTCACTTCCTCTATTTTCTTCACAAAACTAGGAAATTCACCACAATTTTTTCTTTGTTATT<br>TTTTAATTTTTTTTTTATTTTTTAAAAACATTTTTTAAAAAACTAAATTACATACAAACTACCGCATAAAATCCCTCAAACTATACAAACGTTTATCGTATAATATATATAC<br>ATTATTGTTTATTCTATCATTATTAGAGAAACTCCACTACCAAAACCATCATTAAAACAAAAATTTACATGCCACTTAACTCCCCTCACAAACAATCGTTATTTATATTGT<br>TAATTAGCAAACACAAAACCTGC

GU261691	DBGU261691_Myanmar_Hap_F1	ATTTTATTTTTTAACCTAACTCCCCTACTAAGTGTACCCCCCCTTTCCCCCCCAGGGGGGGTATACTATGCATAATCGTGCATACATTTATATACCACATATATTATGGT<br>ACCGGTAATATATACTATATATGTACTAAACCCATTATATGTATACGGGCATTAATCTATATTCCACATTTCTCCCAATGTCCATTCTATGCATGATCCAGGACATACTC<br>ATTCACCCTCCCTACAGACAGCTCCAAACCACCACCAAGTCACCTAACTATGAATGGTTACAGGACATAAATCTCACTCTCATGTTCTTCCCCTACCAAGTCACCTAAC<br>TATGAATGGTTACAGGACATACATTTAACTACC--<br>ATGTTCTAACCCATTTGGTTATGCTCGCCGTACCAGATGGATTTATTGATCGTCCACCTCACGAGAGATCAGCAACCCCTGCTTGTAATGTACTTCATGACCAGTCTCA<br>GGCCCATTCTTTCCCCCTACACCCCTCGCCCTACTTGCCTTCCACCGTACCTCTGGTTCCTCGGTCAGGCACATCCCATGCATAACTCCTGAACTTTCTCACTTTTCAC<br>GAAGTCATCTGTGGATTATCTTCCCCTCTTTAGTCCGTGATCGCGGCATCTTCTCTCTTCTATTGCTGTTGGTTCCTTCTCTTTTTGGGGCTTCTTCACAGGTTGCCCTT<br>CACAGTGCGGGTGCGGAGTGCTATTCAAGTGAAGCCTGGACTACACCTGCGTTGCGTCCTATCCTAGTCCTCTCGTGTCCCTCGATGAGACGGTTTGCGTGTATGGGG<br>AATCATCTTGACACTGATGCACTTTGGATCGCATTTGGTTATGGTTCTTCCACCCCCCC--<br>GGTAAATGGTGCTATTTAGTGAATGCTTGTCGGACATATTTTTACCAATTTTCACTTCCTCTATTTTCTTCACAAAACTAGGAAATTCACCACAATTTTTTCTTTGTTATT<br>TTTTAATTTTTTTTTTATTTTTTAAAAACATTTTTTAAAAAACTAAATTACATACAAACTACCGCATAAAATCCCTCAAACTATACAAACGTTTATCGTATAATATATATAC<br>ATTATTGTTTATTCTATCATTATTAGAGAAACTCCACTACCAAAACCATCATTAAAACAAAAATTTACATGCCACTTAACTCCCCTCACAAACAATCGTTATTTATATTGT<br>TAATTAGCAAACACAAAACCTGC
GU261692	DBGU261692_China_Hap_X	ATTTTATTTTTTAACCTAACTCCCCTACTAAGTGTACCCCCCCTTTCCCCCCCAGGGGGGGTATACTATGCATAATCGTGCATACATTTATATACCACATATATTATGGT<br>ACCGGTAATATATACTATATATGTACTAAACCCATTATATGTATACGGGCATTAATCTATATTCCACATTTCTCCCAATGTCCATTCTATGCATGATCCAGGACATACTC<br>ATTCACCCTCCCCATAGACAACTCCAAACCACTACCAAGTCACCTAACTATGAATGGTTACAGGACATAAATCTCACTCTCATGTTCTTCCCCCAACAAGTCACTTAAC<br>TATGAATGGTTACAGGACATACATTTAACTACT--<br>ATGTTCTAACCCATTTGGTTATGCTCGCCGTATCAGATGGATTTATTGATCGTTCACCTCACGAGAGATCAGCAACCCCTGCTTGTAATGTACTTCATGACCAGTCTCA<br>GGCCCATTCTTTCCCCCTACACCCCTCGCCCTACTTGCCTTCCACCGTACCTCTGGTTCCTCGGTCAGGCACATCCCATGCATAACTCCTGAACTTTCTCACTTTTCAC<br>GAAGTCATCTGTGGATTATCTTCCCCTCTTTAGTCCGTGATCGCGGCATCTTCTCTCTTCTATTGCTGTTGGTTCCTTCTCTTTTTGGGGCTTCTTCACAGGTTGCCCTT<br>CACAGTGCGGGTGCGGAGTGCTATTCAAGTGAAGCCTGGACTACACCTGCGTTGCGTCCTATCCTAGTCCTCTCGTGTCCCTCGATGAGACGGTTTGCGTGTATGGGG<br>AATCATCTTGACACTGATGCACTTTGGATCGCATTTGGTTATGGTTCTTCCACCCCCCC--<br>GGTAAATGGTGCTATTTAGTGAATGCTTGTCGGACATATTTTTATCAATTTTCACTTCCTCTATTTTCTTCACAAAACTAGGAAATTCACCACAATTTTTTCTTTGTTATT<br>TTTTAATTTTTTTTTTATTTTTTAAAAACATTTTTTAAAAAACTAAATTACATACAAACTACCGCATAAAATCCCTCAAACTATACAAACGTTTATCGTATAATATATATAC<br>ATTATTGTTTATTCTATCATTATTAGAGAAACTCCACTACCAAAACCATCATTAAAACAAAAATTTACATGCCACTTAACTCCCCTCACAAACAATCGTTATTTATATTGT<br>TAATTAGCAAACACAAAACCTGC
GU261693	DBGU261693_China_Hap_Y	ATTTTATTTTTTAACCTAACTCCCCTACTAAGTGTACCCCCCCTTTCCCCCCCAGGGGGGGTATACTATGCATAATCGTGCATACATTTATATACCACATATATTATGGT<br>ACCGGTAATATATACTATATATGTACTAAACCCATTATATGTATACGGGCATTAATCTATATTCCACATTTCTCCCAATGTCCATCCTATGCATGATCCAGGACATACTC<br>ATTCACCCTCCCCATAGACAGCTCCAAACCACTACCAAGTCACCTAACCATGAATGGTTGCAGGACATAAATCTCACTCTCATGTTCTTCCCCCAACAAGTCACCTAAC<br>TATGAATGGTTACAGGACATACATTTAACTACC--<br>ATGTTCTAACCCATTTGGTTATGCTCGACGTATCAGATGGATTTATTGATCGTCCACCTCACGAGAGATCAGCAACCCCTGCCTGTAATGTACTTCATGACCAGTCTCA<br>GGCCCATTCTTTCCCCCTACACCCCTCGCCCTACTTGCCTTCCACCGTACCTCTGGTTCCTCGGTCAGGCACATCCCATGCATAACTCCTGAACTTTCTCACTTTTCAC<br>GAAGTCATCTGTGGATTATCTTCCCCTCTTTAGTCCGTGATCGCGGCATCTTCTCTCTTCTATTGCTGTTGGTTCCTTCTCTTTTTGGGGCTTCTTCACAGGTTGCCCTT<br>CACAGTGCGGGTGCGGAGTGCTATTCAAGTGAAGCCTGGACTACACCTGCGTTGCGTCCTATCCTAGTCCTCTCGTGTCCCTCGATGAGACGGTTTGCGTGTATGGGG<br>AATCATCTTGACACTGATGCACTTTGGATCGCATTTGGTTATGGTTCTTCCACCCCCCCC-<br>GGTAAATGGTGCTATTTAGTGAATGCTTGTCGGACATATTTTTATCAATTTTCACTTCCTCTATTTTCTTCACAAAACTAGGAAATTCACCACAATTTTTTCTTTGTTATT<br>TTTTAATTTTTTTTTTATTTTTTAAAAACATTTTTTAAAAAACTAAATTACATACAAACTACCGCATAAAATCCCTCAAACTATACAAACGTTTATCGTATAATATATATAC<br>ATTATTGTTTATTCTATCATTATTAGAGAAACTCCACTACCAAAACCATCATTAAAACAAAAATTTACATGCCACTTAACTCCCCTCACAAACAATCGTTATTTATATTGT<br>TAATTAGCAAACACAAAACCCGC

GU261694	DBGU261694_China_Hap_E1b	ATTTTATTTTTTAACCTAACTCCCCTACTAAGTGTACCCCCCCTTTCCCCCCCAGGGGGGGTATACTATGCATAATCGTGCATACATTTATATACCACATATATTATGGT<br>ACCGGTAATATATACTATATATGTACTAAACCCATTATATGTATACGGGCATTAATCTATATTCCACATTTCTCCCAATGTCCATTCTATGCATGATCCAGGACACACTC<br>ATTCACCCTCCCCATAGACAGTTCCAAACCACTACCAAGTCACCTAACTATGAATGGTTACAGGACATAAATCTCACTCTCATGTTCTTCCCCCAACAAGTCACCTAAC<br>TATGAATGGTTACAGGACATACATTTAACTACC---<br>ATGTTCTAACCCATTTGGTTATGCTCGCCGTATCAGATGGATTTATTGATCGTCCACCTCACGAGAGATCAGCAACCCCTGCTTGTAATGTACTTCATGACCAGTCTCA<br>GGCCCATTCTTTCCCCCTACACCCCTCGCCCTACTTGCCTTCCACCGTACCTCTGGTTCCTCGGTCAGGCACATCCCATGCATAACTCCTGAACTTTCTCACTTTTCAC<br>GAAGTCATCTGTGGATTATCTTCCCCTCTTTAGTCCGTGATCGCGGCATCTTCTCTCTTCTATTGCTGTTGGTTCCTTCTCTTTTTGGGGCTTCTTCACAGGTTACCCTT<br>CACAGTGCGGGTGCGGAGTGCTATTCAAGTGAAGCCTGGACTACACCTGCGTTGCGTCCTATCCTAGTCCTCTCGTGTCCCTCGATGAGACGGTTTGCGTGTATGGGG<br>AATCATCTTGACACTGATGCACTTTGGATCGCATTTGGTTATGGTTCTTCCACCCCCCCC-<br>GGTAAATGGTGCTATTTAGTGAATGCTTGTCGGACATATTTTTATCAATTTTCACTTCCTCTATTTTCTTCACAAAACTAGGAAATTCACCACAATTTTTTCTTTGTTATT<br>TTTTAATTTTTTTTTTATTTTTTAAAAACATTTTTTAAAAAACTAAATTACATACAAACTACCGCATAAAATCCCTCAAACTATACAAACGTTTATCGTATAATATATATAC<br>ATTATTGTTTATTCTATCATTATTAGAGAAACTCCACTACCAAAACCATCATTAAAACAAAAATTTACATGCCACTTAACTCCCCTCACAAACAATCGTTATTTATATTGT<br>TAATTAGCAAACACAAAACCTGC
GU261695	DBGU261695_China_Hap_A1	ATTTTATTTTTTAACCTAACTCCCCTACTAAGTGTACCCCCCCTTTCCCCCCCAGGGGGGGTATACTATGCATAATCGTGCATACATTTATATACCACATATATTATGGT<br>ACCGGTAATATATACTATATATGTACTAAACCCATTATATGTATACGGGCATTAACCTATATTCCACATTTCTCCCAATGTCCATTCTATGCATGATCCAGGACATACTC<br>ATTTACCCTCCCCATAGACAGTTCCAAACCACTATCAAGCCACCTAACTATGAATGGTTACAGGACATAAATCTCACTCTCATGTTCTCCCCCCAACAAGTCACCTAAC<br>TATGAATGGTTACAGGACATACATTTAACTACC---<br>ATGTTCTAACCCATTTGGTTATGCTCGCCGTACCAGATGGATTTATTGATCGTCCACCTCACGAGAGATCAGCAACCCCTGCCTGTAATGTACTTCATGACCAGTCTCA<br>GGCCCATTCTTTCCCCCTACACCCCTCGCCCTACTTGCCTTCCACCGTACCTCTGGTTCCTCGGTCAGGCACATCCCATGCATAACTCCTGAACTTTCTCACTTTTCAC<br>GAAGTCATCTGTGGATTATCTTCCCCTCTTTAGTCCGTGATCGCGGCATCTTCTCTCTTCTATTGCTGTTGGTTCCTTCTCTTTTTGGGGCTTCTTCACAGGTTGCCCTT<br>CACAGTGCGGGTGCGGAGTGCTATTCAAGTGAAGCCTGGACTACACCTGCGTTGCGTCCTATCCTAGTCCTCTCGTGTCCCTCGATGAGACGGTTTGCGTGTATGGGG<br>AATCATCTTGACACTGATGCACTTTGGATCGCATTTGGTTATGGTTCTTCCACCCCCCC--<br>GGTAAATGGTGCTATTTAGTGAATGCTTGTCGGACATATTTTTATCAATTTTCACTTCCTCTATTTTCTTCACAAAACTAGGAAATTCACCACAATTTTTTCTTTGTTATT<br>TTTTAATTTTTTTTTTATTTTTTAAAAACATTTTTTAAAAAACTAAATTACATACAAACTACCGCATAAAATCCCTCAAACTATACAAACGTTTATCGTATAATATATATAC<br>ATTATTGTTTATTCTATCATTATTAGAGAAACTCCACTACCAAAACCATCATTAAAACAAAAATTTACATGCCACTTAACTCCCCTCACAAACAATCGTTATTTATATTGT<br>TAATTAGCAAACACAAAACCCGC
GU261696	DBGU261696_China_Hap_Z	ATTTTATTTTTTAACCTAACTCCCCTACTAAGTGTACCCCCCCTTTCCCCCCCAGGGGGGGTATACTATGCATAATCGTGCATACATTTATATACCACATATATTATGGT<br>ACCGGTAATATATACTATATATGTACTAAACCCATTATATGTATACGGGCATTAATCTATATTCCACATTTCTCCCAATGTCCATTCCATGCATGATCCAGGACATACTC<br>ATTCACCCTCCCCATAGACAGTTCCAAACCACTACCAAGCCACCTAACTATGAATGGTTACAGGACATAGATCTCACTCTCATGCTCTTCCCCCAACAAGTCACCTAAC<br>TATGAATGGTTACAGGACATACATTTAACTACC---<br>ATGTTCTAACCCATTTGGTTATGCTCGCCGTATCAGATGGATTTATTGATCGTCCACCTCACGAGAGATCAGCAACCCCTGCCTGTAATGTACTTCATGACCAGTCTCA<br>GGCCCATTCTTTCCCCCTACACCCCTCGCCCTACTTGCCTTCCACCGTACCTCTGGTTCCTCGGTCAGGCACATCCCATGCATAACTCCTGAACTTTCTCACTTTTCAC<br>GAAGTCATCTGTGGATTATCTTCCCCTCTTTAGTCCGTGATCGCGGCATCTTCTCTCTTCTATTGCTGTTGGTTCCTTCTCTTTTTGGGGCTTCTTCACAGGTTGCCCTT<br>CACAGTGCGGGTGCGGAGTGCTATTCAAGTGAAGCCTGGACTACACCTGCGTTGCGTCCTATCCTAGTCCTCTCGTGTCCCTCGATGAGACGGTTTGCGTGTATGGGG<br>AATCATCTTGACACTGATGCACTTTGGATCGCATTTGGTTATGGTTCTTCCACCCCCCCC-<br>GGTAAATGGTGCTATTTAGTGAATGCTTGCCGGACATATTTTTATCAATTTTCACTTCCTCTATTTTCTTCACAAAACTAGGAAATTCACCACAATTTTTTCTTTGTTATT<br>TTTTAATTTTTTTTTTATTTTTTAAAAACATTTTTTAAAAAACTAAATTACATACAAACTACCGCATAAAATCCCTCAAACTATACAAACGTTTATCGTATAATATATATAC<br>ATTATTGTTTATTCTATCATTATTAGAGAAACTCCACTACCAAAACCATCATTAAAACAAAAATTTACATGCCACTTAACTCCCCTCACAAACAATCGTTATTTATATTGT<br>TAATTAGCAAACACAAAACCCGC

GU261697	DBGU261697_India_Hap_D3a	ATTTTATTTTTTAACCTAACTCCCCTACTAAGTGTACCCCCCCTTTCCCCCCCAGGGGGGGTATACTATGCATAATCGTGCATACATTTATATACCACATATATTATGGT<br>ACCGGTAATATATACTATATATGTACTAAACCCATTATATGTATACGGGCATTAATCTATATTCCACATTTCTCCCAATGTCCATTCTATGCATGATCCAGGACATACTC<br>ATTCACCCTCCCCATAGACAGCTCCAAACCACTACCAAGTCACCTAACTATGAATGGTTACAGGACATAAATCTCACTCTCATGCTCTTCCCCCAACAAGTCACCTAAC<br>TATGAATGGTTACAGGACATACATTTAACTACC---<br>ATGTTCTAACCCATTTGGTTATGCTCGCCGTATCAGATGGATTTATTGATCGTCCACCTCACGAGAGATCAGCAACCCCTGCCTGTAATGTACTTCATGACCAGTCTCA<br>GGCCCATTCTTTCCCCCTACACCCCTCGCCCTACTTGCCTTCCACCGTACCTCTGGTTCCTCGGTCAGGCACATCCCATGCATAACTCCTGAACTTTCTCACTTTTCAC<br>GAAGTCATCTGTGGATTATCTTCCCCTCTTTAGTCCGTGATCGCGGCATCTTCTCTCTTCTATTGCTGTTGGTTCCTTCTCTTTTTGGGGCTTCTTCACAGGTTGCCCTT<br>CACAGTGCGGGTGCGGAGTGCTATTCAAGTGAAGCCTGGACTACACCTGCGTTGCGTCCTATCCTAGTCCTCTCGTGTCCCTCGATGAGACGGTTTGCGTGTATGGGG<br>AATCATCTTGACACTGATGCACTTTGGATCGCATTTGGTTATGGTTCTTCCACCCCCCCC-<br>GGTAAATGGTGCTATTTAGTGAATGCTTGTCGGACATATTTTTATCAATTTTCACTTCCTCTATTTTCTTCACAAAACTAGGAAATTCACCACAATTTTTTCTTTGTTATT<br>TTTTAATTTTTTTTTTATTTTTTAAAAACATTTTTTAAAAAACTAAATTACATACAAACTACCGCATAAAATCCCTCAAACTATACAAACGTTTATCGTATAATATATATAC<br>ATTATTGTTTATTCTATCATTATTAGAGAAACTCCACTACCAAAACCATCATTAAAACAAAAATTTACATGCCACTTAACTCCCCTCACAAACAATCGTTATTTATATTGT<br>TAATTAGCAAACACAAAACCCGC
GU261698	DBGU261698_India_Hap_I	ATTTTATTTTTTAACCTAACTCCCCTACTAAGTGTACCCCCCCTTTCCCCCCCAGGGGGGGTATACTATGCATAATCGTGCATACATTTATATACCACATATATTATGGT<br>ACCGGTAATATATACTATATATGTACTAAACCCATTATATGTATACGGGCATTAATCTACATTCCACATTTCTCCCAATGTCCATTCTATGCATGGTCCAAGACATACTT<br>ATTCACCTTCCCCATAGACAGCTCTAAACCACTACCAAGTCACCTAACTATGAATGGTTACAGGACATAAATCTCACTCTCATGTTCTTCCCCCAACAAGACACCTAAC<br>TATGAATGGTTACAGGACATACACTTAACTATT---<br>ATGTTCTAACCCATTTGGTTATGCTCGTCGTATCAGATGGATTTATTGATCGTCCACCTCACGAGAGATCAGCAACCCCTGCTTGTAATGTACTTCATGACCAGTCTCA<br>GGCCCATTCTTTCCCCCTACACCCCTCGCCCTACTTGCCTTCCACCGTGCCTCTGGTTCCTCGGTCAGGCACATCCCATGCATAACTCCTGAACTTTCTCACTTTTCAC<br>GAAGTCATCTGTGGATTATCTTCCCCTCTTTAGTCCGTGATCGCGGCATCTTCTCTCTTCTATTGCTGTTGGTTCCTTCTCTTTTTGGGGCTTCTTCACAGGTTACCCTT<br>CACAGTGCGGGTGCGGAGTGCTATTCAAGTGAAGCCTGGACTACACCTGCGTTGCGTCCTATCCTAGTCCTCTCGTGTCCCTCGATGAGACGGTTTGCGTGTATGGGG<br>AATCATCTTGACACTGATGCACTTTGGATCGCATTTGGTTATGGTTCTTCCACCCCCCC--<br>GGTAAATGGTGCTATTTAGTGAATGCTTGTCGGACATATTTTTATCAATTTTCACTTCCTCTATTTTCTTCACAAAACTAGGAAATTCACCACAATTTTTTCTTTGTTATT<br>TTTTAATTTTTTTTTTATTTTTTAAAAACATTTTTTAAAAAACTAAATTACATACAAACTACCGCATAAAATCCCTCAAACTATACAAACGTTTATCGTATAATATATATAC<br>ATTATTGTTTATTCTATCATTATTAGAGAAACTCCACTACCAAAACCATCATTAAAACAAAAATTTACATGCCACTTAACTCCCCTCACAAACAATCGTTATTTATATTGT<br>TAATTAGCAAACACAAAACCCGC
GU261699	DBGU261699_China_Hap_B1a	ATTTTATTTTTTAACCTAACTCCCCTACTAAGTGTACCCCCCCTTTCCCCCCCAGGGGGGGTATACTATGCATAATCGTGCATACATTTATATACCACATATATTATGGT<br>ACCGGTAATATATACTATATATGTACTAAACCCATTATATGTATACGGGCATTAATCTATATTCCACATTTCTCCCAATGTCCATTCTATGCATGATCCAAGACATACTC<br>ATTCACCCTCCCCATAGACAGTTCTAAACCACTACCAAGCCACCTAACTATGAATGGTTACAGGACATAAATCTCACTCTCATGTTCTCCCCCTAACAAGTCACCTAAC<br>TATGAATGGTTACAGGACATACATTTAACTACC---<br>ATGTTCTAACCCATTTGGTTATGCTCGCCGTATCAGATGGATTTATTGATCGTCCACCTCACGAGAGATCAGCAACCCCTGCCTGTAATGTACTTCATGACCAGTCTCA<br>GGCCCATTCTTTCCCCCTACACCCCTCGCCCTACTTGCCTTCCACCGTACCTCTGGTTCCTCGGTCAGGCACATCCCATGCATAACTCCTGAACTTTCTCACTTTTCAC<br>GAAGTCATCTGTGGATTATCTTCCCCTCTTTAGTCCGTGATCGCGGCATCTTCTCTCTTCTATTGCTGTTGGTTCCTTCTCTTTTTGGGGCTTCTTCACAGGTTGCCCTT<br>CACAGTGCGGGTGCGGAGTGCTATTCAAGTGAAGCCTGGACTACACCTGCGTTGCGTCCTATCCTAGTCCTCTCGTGTCCCTCGATGAGACGGTTTGCGTATATGGGG<br>AATCATCTTGACACTGATGCACTTTGGATCGCATTTGGTTATGGTTCTTCCACCCCCCCC-<br>GGTAAATGGTGCTATTTAGTGAATGCTTGTCGGACATATTTTTATCAATTTTCACTTCCTCTATTTTCTTCACAAAACTAGGAAATTCACCACAATTTTTTCTTTGTTATT<br>TTTTAATTTTTTTTTTATTTTTTAAAAACATTTTTTAAAAAACTAAATTACATACAAACTACCGCATAAAATCCCTCAAACTATACAAACGTTTATCGTATAATATATATAC<br>ATTATTGTTTATTCTATCATTATTAGAGAAACTCCACTACCAAAACCATCATTAAAACAAAAATTTACATGCCACTTAACTCCCCTCACAAACAATCGTTATTTATATTGT<br>TAATTAGCAAACACAAAACCCAC

GU261700	DBGU261700_Myanmar_Hap_A1a	ATTTTATTTTTTAACCTAACTCCCCTACTAAGTGTACCCCCCCTTTCCCCCCCAGGGGGGGTATACTATGCATAATCGTGCATACATTTATATACCACATATATTATGGT<br>ACCGGTAATATATACTATATATGTACTAAACCCATTATATGTATACGGGCATTAACCTATATTCCACATTTCTCCCAATGTCCATTCTATGCATGATCCAGGACATACTC<br>ATTTACCCTCCCCATAGACAGTTCCAAACCACTACCAAGCCACCTAACTATGAATGGTTACAGGACATAAATCTCACTCTCATGTTCTCCCCCCAACAAGTCACCTAAC<br>TATGAATGGTTACAGGACATACATTTAACTACC--<br>ATGTTCTAACCCATTTGGTTATGCTCGACGTATCAGATGGATTTATTGATCGTCCACCTCACGAGAGATCAGCAACCCCTGCCTGTAATGTACTTCATGACCAGTCTCA<br>GGCCCATTCTTTCCCCCTACACCCCTCGCCCTACTTGCCTTCCACCGTACCTCTGGTTCCTCGGTCAGGCACATCCCATGCATAACTCCTGAACTTTCTCACTTTTCAC<br>GAAGTCATCTGTGGATTATCTTCCCCTCTTTAGTCCGTGATCGCGGCATCTTCTCTCTTCTATTGCTGTTGGTTCCTTCTCTTTTTGGGGCTTCTTCACAGGTTGCCCTT<br>CACAGTGCGGGTGCGGAGTGCTATTCAAGTGAAGCCTGGACTACACCTGCGTTGCGTCCTATCCTAGTCCTCTCGTGTCCCTCGATGAGACGGTTTGCGTGTATGGGG<br>AATCATCTTGACACTGATGCACTTTGGATCGCATTTGGTTATGGTTCTTCCACCCCCCC--<br>GGTAAATGGTGCTATTTAGTGAATGCTTGTCGGACATATTTTTATCAATTTTCACTTCCTCTATTTTCTTCACAAAACTAGGAAATTCACCACAATTTTTTCTTTGTTATT<br>TTTTAATTTTTTTTTTATTTTTTAAAAACATTTTTTAAAAAACTAAATTACATACAAACTACCGCATAAAATCCCTCAAACTATACAAACGTTTATCGTATAATATATATAC<br>ATTATTGTTTATTCTATCATTATTAGAGAAACTCCACTACCAAAACCATCATTAAAACAAAAATTTACATGCCACTTAACTCCCCTCACAAACAATCGTTATTTATATTGT<br>TAATTAGCAAACACAAAACCCGC
GU261701	DBGU261701_China_Hap_C1c	ATTTTATTTTTTAACCTAACTCCCCTACTAAGTGTACCCCCCCTTTCCCCCCCAGGGGGGGTATACTATGCATAATCGTGCATACATTTATATACCACATATATTATGGT<br>ACCGGTAATATATACTATATATGTACTAAACCCATTATATGTATACGGGCATTAATCTATATTCCACATTTCTCCCAATGTCCATTCTATGCATGATCCAAGACATACTC<br>ATTCACCCTCCCCATAGACAACTCCAAACCACTACCAAGCCACCTAACTATGAATGGTTGCAGGACATAAATCTCACTCTCATGTTCTTCCCCCAACAAGTCACCTAAC<br>TATGAATGGTTACAGGACATACATTTAACTACT--<br>ATGCTCTAACCCATTTGGTTATGCTCGCCGTATCAGATGGATTTATTGATCGTCCACCTCACGAGAGATCAGCAACCCCTGCCTGTAATGTACTTCATGACCAGTCTCA<br>GGCCCATTCTTTCCCCCTACACCCCTCGCCCTACTTGCCTTCCACCGTACCTCTGGTTCCTCGGTCAGGCACATCCCATGCATAACTCCTGAACTTTCTCACTTTTCAC<br>GAAGTCATCTGTGGATTATCTTCCCCTCTTTAGTCCGTGATCGCGGCATCTTCTCTCTTCTATTGCTGTTGGTTCCTTCTCTTTTTGGGGCTTCTTCACAGGTTGCCCTT<br>CACAGTGCGGGTGCGGAGTGCTATTCAAGTGAAGCCTGGACTACACCTGCGTTGCGTCCTATCCTAGTCCTCTCGTGTCCCTCGATGAGACGGTTTGCGTGTATGGGG<br>AATCATCTTGACACTGATGCACTTTGGATCGCATTTGGTTATGGTTCTTCCACCCCCCC--<br>GGTAAATGGTGCTATTTAGTGAATGCTTGTCGGACATATTTTTATCAATTTTCACTTCCTCTATTTTCTTCACAAAACTAGGAAATTCACCACAATTTTTTCTTTGTTATT<br>TTTTAATTTTTTTTTTATTTTTTAAAAACATTTTTTAAAAAACTAAATTACATACAAACTACCGCATAAAATCCCTCAAACTATACAAACGTTTATCGTATAATATATATAC<br>ATTATTGTTTATTCTATCATTATTAGAGAAACTCCACTACCAAAACCATCATTAAAACAAAAATTTACATGCCACTTAACTCCCCTCACAAACAATCGTTATTTATATTGT<br>TAATTAGCAAACACAAAACCCGC
GU261702	DBGU261702_China_Hap_F1	ATTTTATTTTTTAACCTAACTCCCCTACTAAGTGTACCCCCCCTTTCCCCCCCAGGGGGGGTATACTATGCATAATCGTGCATACATTTATATACCACATATATTATGGT<br>ACCGGTAATATATACTATATATGTACTAAACCCATTATATGTATACGGGCATTAATCTATATTCCACATTTCTCCCAATGTCCATTCTATGCATGATCCAGGACATACTC<br>ATTCACCCTCCCTACAGACAGCTCCAAACCACCACCAAGTCACCTAACTATGAATGGTTACAGGACATAAATCTCACTCTCATGTTCTTCCCCTACCAAGTCACCTAAC<br>TATGAATGGTTACAGGGCATACATTTAACTACC--<br>ATGTTCTAACCCATTTGGTTATGCTCGCCGTACCAGATGGATTTATTGATCGTCCACCTCACGAGAGATCAGCAACCCCTGCTTGTAATGTACTTCATGACCAGTCTCA<br>GGCCCATTCTTTCCCCCTACACCCCTCGCCCTACTTGCCTTCCACCGTACCTCTGGTTCCTCGGTCAGGCACATCCCATGCATAACTCCTGAACTTTCTCACTTTTCAC<br>GAAGTCATCTGTGGATTATCTTCCCCTCTTTAGTCCGTGATCGCGGCATCTTCTCTCTTCTATTGCTGTTGGTTCCTTCTCTTTTTGGGGCTTCTTCACAGGTTGCCCTT<br>CACAGTGCGGGTGCGGAGTGCTATTCAAGTGAAGCCTGGACTACACCTGCGTTGCGTCCTATCCTAGTCCTCTCGTGTCCCTCGATGAGACGGTTTGCGTGTATGGGG<br>AATCATCTTGACACTGATGCACTTTGGATCGCATTTGGTTATGGTTCTTCCACCCCCCCC-<br>GGTAAATGGTGCTATTTAGTGAATGCTTGTCGGACATATTTTTACCAATTTTCACTTCCTCTATTTTCTTCACAAAACTAGGAAATTCACCACAATTTTTTCTTTGTTATT<br>TTTTAATTTTTTTTTTATTTTTTAAAAACATTTTTTAAAAAACTAAATTACATACAAACTACCGCATAAAATCCCTCAAACTATACAAACGTTTATCGTATAATATATATAC<br>ATTATTGTTTATTCTATCATTATTAGAGAAACTCCACTACCAAAACCATCATTAAAACAAAAATTTACATGCCACTTAACTCCCCTCACAAACAATCGTTATTTATATTGT<br>TAATTAGCAAACACAAAACCTGC

GU261703	DBGU261703_Myanmar_Hap_F2	ATTTTATTTTTTAACCTAACTCCCCTACTAAGTGTACCCCCCCTTTCCCCCCCAGGGGGGGTATACTATGCATAATCGTGCATACATTTATATACCACATATATTATGGT ACCGGTAATATATACTATATATGTACTAAACCCATTATATGTATACGGGCATTAATCTATATTCCACATTTCTCCCAATGTCCATTCTATGCATGATCCAGGACATACTC ATTCACCCTCCCTACAGACAGCTCCAAACCACCACCAAGTCACCTAACTATGAATGGTTACAGGACATAAATCTCACTCTCATGTTCTTCCCCTACCAAGTCACCTAAC TATGAATGGTTACAGGACATACATTTAACTACC-- ATGTTCTAACCCATTTGGTTATGCTCGCCGTATCAGATGGATTTATTGATCGTCCACCTCACGAGAGATCAGCAACCCCTGCTTGTAATGTACTTCATGACCAGTCTCA GGCCCATTCTTTCCCCCTACACCCCTCGCCCTACTTGCCTTCCACCGTACCTCTGGTTCCTCGGTCAGGCACATCCCATGCATAACTCCTGAACTTTCTCACTTTTCAC GAAGTCATCTGTGGATTATCTTCCCCTCTTTAGTCCGTGATCGCGGCATCTTCTCTCTTCTATTGCTGTTGGTTCCTTCTCTTTTTGGGGCTTCTTCACAGGTTGCCCTT CACAGTGCGGGTGCGGAGTGCTATTCAAGTGAAGCCTGGACTACACCTGCGTTGCGTCCTATCCTAGTCCTCTCGTGTCCCTCGATGAGACGGTTTGCGTGTATGGGG AATCATCTTGACACTGATGCACTTTGGATCGCATTTGGTTATGGTTCTTCCACCCCCCC-- GGTAAATGGTGCTATTTAGTGAATGCTTGTCGGACATATTTTTACCAATTTTCACTTCCTCTATTTTCTTCACAAAACTAGGAAATTCACCACAATTTTTTCTTTGTTATT TTTTAATTTTTTTTTTATTTTTTAAAAACATTTTTTAAAAAACTAAATTACATACAAACTACCGCATAAAATCCCTCAAACTATACAAACGTTTATCGTATAATATATATAC ATTATTGTTTATTCTATCATTATTAGAGAAACTCCACTACCAAAACCATCATTAAAACAAAAATTTACATGCCACTTAACTCCCCTCACAAACAATCGTTATTTATATTGT TAATTAGCAAACACAAAACCTGC
GU261704	DBGU261704_Myanmar_Hap_B1	ATTTTATTTTTTAACCTAACTCCCCTACTAAGTGTACCCCCCCTTTCCCCCCCAGGGGGGGTATACTATGCATAATCGTGCATACATTTATATACCACATATATTATGGT ACCGGTAATATATACTATATATGTACTAAACCCATTATATGTATACGGGCATTAATCTATATTCCACATTTCTCCCAATGTCCATTCTATGCATGATCCAAGACATACTC ACTCACCCTCCCCATAGACAGTTCTAAACCACTATCAAGCCACCTAACTATGAATGGTTACAGGACATAAATCTTACTCTCATGTTCTCCCCCTAACAAGTCACCTAAC TATGAATGGTTACAGGACATACATTTAACTACC-- ATGTTCTAACCCATTTGGTTATGCTCGCCGTATCAGATGGATTTATTGATCGTCCACCTCACGAGAGATCAGCAACCCCTGCCTGTAATGTACTTCATGACCAGTCTCA GGCCCATTCTTTCCCCCTACACCCCTCGCCCTACTTGCCTTCCACCGTACCTCTGGTTCCTCGGTCAGGCACATCCCATGCATAACTCCTGAACTTTCTCACTTTTCAC GAAGTCATCTGTGGATTATCTTCCCCTCTTTAGTCCGTGATCGCGGCATCTTCTCTCTTCTATTGCTGTTGGTTCCTTCTCTTTTTGGGGCTTCTTCACAGGTTGCCCTT CACAGTGCGGGTGCGGAGTGCTATTCAAGTGAAGCCTGGACTACACCTGCGTTGCGTCCTATCCTAGTCCTCTCGTGTCCCTCGATGAGACGGTTTGCGTGTATGGGG AATCATCTTGACACTGATGCACTTTGGATCGCATTTGGTTATGGTTCTTCCACCCCCCC-- GGTAAATGGTGCTATTTAGTGAATGCTTGTCGGACATATTTTTATCAATTTTCACTTCCTCTATTTTCTTCACAAAACTAGGAAATTCACCACAATTTTTTCTTTGTTATT TTTTAATTTTTTTTTTATTTTTTAAAAACATTTTTTAAAAAACTAAATTACATACAAACTACCGCATAAAATCCCTCAAACTATACAAACGTTTATCGTATAATATATATAC ATTATTGTTTATTCTATCATTATTAGAGAAACTCCACTACCAAAACCATCATTAAAACAAAAATTTACATGCCACTTAACTCCCCTCACAAACAATCGTTATTTATATTGT TAATTAGCAAACACAAAACCCAC
GU261705	DBGU261705_China_Hap_B1a	ATTTTATTTTTTAACCTAACTCCCCTACTAAGTGTACCCCCCCTTTCCCCCCCAGGGGGGGTATACTATGCATAATCGTGCATACATTTATATACCACATATATTATGGT ACCGGTAATATATACTATATATGTACTAAACCCATTATATGTATACGGGCATTAATCTATATTCCACATTTCTCCCAATGTCCATTCTATGCATGATCCAAGACATACTC ATTCACCCTCCCCATAGACAGTTCTAAACCACTATCAAGCCACCTAACTATGAATGGTTACAGGACATAAATCTCACTCTCATGTTCTCCCCCTAACAAGTCACCTAAC TATGAATGGTTACAGGACATACATTTAACTACC-- ATGTTCTAACCCATTTGGTTATGCTCGCCGTATCAGATGGATTTATTGATCGTCCACCTCACGAGAGATCAGCAACCCCTGCCTGTAATGTACTTCATGACCAGTCTCA GGCCCATTCTTTCCCCCTACACCCCTCGCCCTACTTGCCTTCCACCGTACCTCTGGTTCCTCGGTCAGGCACATCCCATGCATAACTCCTGAACTTTCTCACTTTTCAC GAAGTCATCTGTGGATTATCTTCCCCTCTTTAGTCCGTGATCGCGGCATCTTCTCTCTTCTATTGCTGTTGGTTCCTTCTCTTTTTGGGGCTTCTTCACAGGTTACCCTT CACAGTGCGGGTGCGGAGTGCTATTCAAGTGAAGCCTGGACTACACCTGCGTTGCGTCCTATCCTAGTCCTCTCGTGTCCCTCGATGAGACGGTTTGCGTATATGGGG AATCATCTTGACACTGATGCACTTTGGATCGCATTTGGTTATGGTTCTTCCACCCCCCCC- GGTAAATGGTGCTATTTAGTGAATGCTTGTCGGACATATTTTTATCAATTTTCACTTCCTCTATTTTCTTCACAAAACTAGGAAATTCACCACAATTTTTTCTTTGTTATT TTTTAATTTTTTTTTTATTTTTTAAAAACATTTTTTAAAAAACTAAATTACATACAAACTACCGCATAAAATCCCTCAAACTATACAAACGTTTATCGTATAATATATATAC ATTATTGTTTATTCTATCATTATTAGAGAAACTCCACTACCAAAACCATCATTAAAACAAAAATTTACATGCCACTTAACTCCCCTCACAAACAATCGTTATTTATATTGT TAATTAGCAAACACAAAACCCAC

GU261706	DBGU261706_China_Hap_W1	ATTTTATTTTTTAACCTAACTCCCCTACTAAGTGTACCCCCCCTTTCCCCCCCAGGGGGGGTATACTATGCATAATCGTGCATACATTTATATACCACATATATTATGGT<br>ACCGGTAATATATACTATATACGTACTAAACCCATTATATGTATACGGGCATTAACCTATATTCCACATTTCTCCCAATGTCCATTCTATGCATGATCCAGGACATACTC<br>ATTCACCCTCCCCATAGACAGCTCCAAACCACTACCAAGTCACCTAACTATGAATGGTTACAGGACATAAATCTCACTCTCATGTTCTCCCCCCAACAAGTCACCTAAC<br>TATGAATGGTTACAGGACATACATTTAACTACT---<br>ATGTTCTAACCCATTTGGTTATGCTCGTCGTATCAGATGGATTTATTGATCGTTCACCTCACGAGAGATCAGCAACCCCTGCTTGTAATGTACTTCATGACCAGTCTCA<br>GGCCCATTCTTTCCCCCTACACCCCTCGCCCTACTTGCCTTCCACCGTGCCTCTGGTTCCTCGGTCAGGCACATCCCATGCATAACTCCTGAACTTTCTCACTTTTCAC<br>GAAGTCATCTGTGGATTATCTTCCCCTCTTTAGTCCGTGATCGCGGCATCTTCTCTCTTCTATTGCTGTTGGTTCCTTCTCTTTTTGGGGCTTCTTCACAGGTTGCCCTT<br>CACAGTGCGGGTGCGGAGTGCTATTCAAGTGAAGCCTGGACTACACCTGCGTTGCGTCCTATCCTAGTCCTCTCGTGTCCCTCGATGAGACGGTTTGCGTGTATGGGG<br>AATCATCTTGACACTGATGCACTTTGGATCGCATTTGGTTATGGTTCTTCCACCCCCCC--<br>GGTAAATGGTGCTATTTAGTGAATGCTTGTCGGACATATTTTTATCAATTTTCACTTCCTCTATTTTCTTCACAAAACTAGGAAATTCACCACAATTTTTTCTTTGTTATT<br>TTTTAATTTTTTTTTTATTTTTTAAAAACATTTTTTAAAAAACTAAATTACATACAAACTACCGCATAAAATCCCTCAAACTATACAAACGTTTATCGTATAATATATATAC<br>ATTATTGTTTATTCTATCATTATTAGAGAAACTCCACTACCAAAACCATCATTAAAACAAAAATTTACATGCCACTTAACTCCCCTCACAAACAATCGTTATTTATATTGT<br>TAATTAGCAAACACAAAACCTGC
GU261707	DBGU261707_India_Hap_C3	ATTTTATTTTTTAACCTAACTCCCCTACTAAGTGTACCCCCCCTTTCCCCCCCAGGGGGGGTATACTATGCATAATCGTGCATACATTTATATACCACATATATTATGGT<br>ACCGGTAATATATACTATATATGTACTAAACCCATTATATGTATACGGGCATTAATCTATATTCCTCATTTCTCCCAATGTCCATTCTATGCATGATCCAGGACATACTC<br>ATTCACCCTCCCCATAGACAGCTCCAAACCACTACCAAGTCACTTAACTATGAATGGTTGCAGGACATAAATCTCACTCTCATGCTCTTCCCCCAACAAGTCACCTAAC<br>TATGAATGGTTACAGGACATACATTTAACTACT---<br>ATGTTCTAACCCATTTGGTTATGCTCGCCGTATCAGATGGATTTATTGATCGTCCACCTCACGAGAGATCAGCAACCCCTGCCTGTAATGTACTTCATGACCAGTCTCA<br>GGCCCATTCTTTCCCCCTACACCCCTCGCCCTACTTGCCTTCCACCGTACCTCTGGTTCCTCGGTCAGGCACATCCCATGCATAACTCCTGAACTTTCTCACTTTTCAC<br>GAAGTCATCTGTGGATTATCTTCCCCTCTTTAGTCCGTGATCGCGGCATCTTCTCTCTTCTATTGCTGTTGGTTCCTTCTCTTTTTGGGGCTTCTTCACAGGTTGCCCTT<br>CACAGTGCGGGTGCGGAGTGCTATTCAAGTGAAGCCTGGACTACACCTGCGTTGCGTCCTATCCTAGTCCTCTCGTGTCCCTCGATGAGACGGTTTGCGTGTATGGGG<br>AATCATCTTGACACTGATGCACTTTGGATCGCATTTGGTTATGGTTCTTCCACCCCCCC--<br>GGTAAATGGTGCTATTTAGTGAATGCTTGTCGGACATATTTTTATCAATTTTCACTTCCTCTATTTTCTTCACAAAACTAGGAAATTCACCACAATTTTTTCTTTGTTATT<br>TTTTAATTTTTTTTTTATTTTTTAAAAACATTTTTTAAAAAACTAAATTACATACAAACTACCGCATAAAATCCCTCAAACTATACAAACGTTTATCGTATAATATATATAC<br>ATTATTGTTTATTCTATCATTATTAGAGAAACTCCACTACCAAAACCATCATTAAAACAAAAATTTACATGCCACTTAACTCCCCTCACAAACAATCGTTATTTATATTGT<br>TAATTAGCAAACACAAAACCCGC
GU261708	DBGU261708_India_Hap_E3b	ATTTTATTTTTTAACCTAACTCCCCTACTAAGTGTACCCCCCCTTTCCCCCCCAGGGGGGGTATACTATGCATAATCGTGCATACATTTATATACCACATATATTATGGT<br>ACCGGTAATATATACTATATATGTACTAAACCCATTATATGTATACGGGCATTAATCTATATTCCACATTTCTCCCAATGTCCATTCTATGCATGATCCAGGACACACTC<br>ATTCACCCTCCCCATAGACAGCTCTAAGCCACTACCAAGTCACCTAACTATGAATGGTTACAGGACATAAATCTCACTCTCATGTTCTTCCCCCAACAAGTCACCTAAC<br>TATGAATGGTTACGGGACATACATCTAACTACC---<br>ATGTTCTAACCCATTTGGTTATGCTCGCCGTATCAGATGGATTTATTGATCGTCCACCTCACGAGAGATCAGCAACCCCTGCTTGTAATGTACTTCATGACCAGTCTCA<br>GGCCCATTCTTTCCCCCTACACCCCTCGCCCTACTTGCCTTCCACCGTACCTCTGGTTCCTCGGTCAGGCACATCCCATGCATAACTCCTGAACTTTCTCACTTTTCAC<br>GAAGTCATCTGTGGATTATCTTCCCCTCTTTAGTCCGTGATCGCGGCATCTTCTCTCTTCTATTGCTGTTGGTTCCTTCTCTTTTTGGGGCTTCTTCACAGGTTGCCCTT<br>CACAGTGCGGGTGCGGAGTGCTATTCAAGTGAAGCCTGGACTACACCTGCGTTGCGTCCTATCCTAGTCCTCTCGTGTCCCTCGATGAGACGGTTTGCGTGTATGGGG<br>AATCATCTTGACACTGATGCACTTTGGATCGCATTTGGTTATGGTTCTTCCACCCCCCC--<br>GGTAAATGGTGCTATTTAGTGAATGCTTGTCGGACATATTTTTATCAATTTTCACTTCCTCTATTTTCTTCACAAAACTAGGAAATTCACCACAATTTTTTCTTTGTTATT<br>TTTTAATTTTTTTTTTATTTTTTAAAAACATTTTTTAAAAAACTAAATTACATACAAACTACCGCATAAAATCCCTCAAACTATACAAACGTTTATCGTATAATATATATAC<br>ATTATTGTTTATTCTATCATTATTAGAGAAACTCCACTACCAAAACCATCATTAAAACAAAAATTTACATGCCACTTAACTCCCCTCACAAACAATCGTTATTTATATTGT<br>TAATTAGCAAACACAAAACCTGC

GU261709	DBGU261709_India_Hap_E1	ATTTTATTTTTTAACCTAACTCCCCTACTAAGTGTACCCCCCCTTTCCCCCCCAGGGGGGGTATACTATGCATAATCGTGCATACATTTATATACCACATATATTATGGT<br>ACCGGTAATATATACTATATATGTACTAAACCCATTATATGTATACGGGCATTAATCTATATTCCACATTTCTCCCAATGTCCATTCTATGCATGATCCAGGACACACTC<br>ATTCACCCTCCCCATAGACAGCTCCAAACCACTACCAAGTCACCTAACTATGAATGGTTACAGGACATAAATCTCACTCTCATGTTCTTCCCCCAACAAGTCACCTAAC<br>TATGAATGGTTACAGGACATACATTTAACTACC--<br>ATGTTCTAACCCATTTGGTTATGCTCGCCGTATCAGATGGATTTATTGATCGTCCACCTCACGAGAGATCAGCAACCCCTGCTTGTAATGTACTTCATGACCAGTCTCA<br>GGCCCATTCTTTCCCCCTACACCCCTCGCCCTACTTGCCTTCCACCGTACCTCTGGTTCCTCGGTCAGGCACATCCCATGCATAACTCCTGAACTTTCTCACTTTTCAC<br>GAAGTCATCTGTGGATTATCTTCCCCTCTTTAGTCCGTGATCGCGGCATCTTCTCTCTTCTATTGCTGTTGGTTCCTTCTCTTTTTGGGGCTTCTTCACAGGTTGCCCTT<br>CACAGTGCGGGTGCGGAGTGCTATTCAAGTGAAGCCTGGACTACACCTGCGTTGCGTCCTATCCTAGTCCTCTCGTGTCCCTCGATGAGACGGTTTGCGTGTATGGGG<br>AATCATCTTGACACTGATGCACTTTGGATCGCATTTGGTTATGGTTCTTCCACCCCCCC--<br>GGTAAATGGTGCTATTTAGTGAATGCTTGTCGGACATATTTTTATCAATTTTCACTTCCTCTATTTTCTTCACAAAACTAGGAAATTCACCACAATTTTTTCTTTGTTATT<br>TTTTAATTTTTTTTTTTATTTTTTAAAAACATTTTTTAAAAAACTAAATTACATACAAACTACCGCATAAAATCCCTCAAACTATACAAACGTTTATCGTATAATATATATAC<br>ATTATTGTTTATTCTATCATTATTAGAGAAACTCCACTACCAAAACCATCATTAAAACAAAAATTTACATGCCACTTAACTCCCCTCACAAACAATCGTTATTTATATTGT<br>TAATTAGCAAACACAAAACCTGC
GU261710	DBGU261710_China_Hap_G1	ATTTTATTTTTTAACCTAACTCCCCTACTAAGTGTACCCCCCCTTTCCCCCCCAGGGGGGGTATACTATGCATAATCGTGCATACATTTATATACCACATATATTATGGT<br>ACCGGTAATATATACTATATATGTACTAAACCCATTATATGTATACGGGCATTAATCTATATTCCACATTTCTCCCAATGTCCATTCTATGCATGATCCAAGACATACTC<br>ATTCACCCTCCCCATAGACAGCTCCAAACCACTACCAAGTCACCTAACTATGAATGGTTGCAGGACATAAATCTAACTCTTATGTTCTTCCCCTACCAAGTCACCTAAC<br>TATGAATGGTTACAGGACATACATTTAACTACC--<br>ATGTTCTAACCCATTTGGTTATGCTCGACGTATCAGATGGATTTATTGATCGTCCACCTCACGAGAGATCAGCAACCCCTGCTTGTAATGTACTTCATGACCAGTCTCA<br>GGCCCATTCTTTCCCCCTACACCCCTCGCCCTACTTGCCTTCCACCGTACCTCTGGTTCCTCGGTCAGGCACATCCCATGCATAACTCCTGAACTTTCTCACTTTTCAC<br>GAAGTCATCTGTGGATTATCTTCCCCTCTTTAGTCCGTGATCGCGGCATCTTCTCTCTTCTATTGCTGTTGGTTCCTTCTCTTTTTGGGGCTTCTTCACAGGTTACCCTT<br>CACAGTGCGGGTGCGGAGTGCTATTCAAGTGAAGCCTGGACTACACCTGCGTTGCGTCCTATCCTAGTCCTCTCGTGTCCCTCGATGAGACGGTTTGCGTGTATGGGG<br>AATCATCTTGACACTGATGCACTTTGGATCGCATTTGGTTATGGTTCTTCCACCCCCCCC-<br>GGTAAATGGTGCTATTTAGTGAATGCTTGTCGGACATATTTTTATCAATTTTCACTTCCTCTATTTTCTTCACAAAACTAGGAAATTCACCACAATTTTTTCTTTGTTATT<br>TTTTAATTTTTTTTTTTATTTTTTAAAAACATTTTTTAAAAAACTAAATTACATACAAACTACCGCATAAAATCCCTCAAACTATACAAACGTTTATCGTATAATATATATAC<br>ATTATTGTTTATTCTATCATTATTAGAGAAACTCCACTACCAAAACCATCATTAAAACAAAAATTTACATGCCACTTAACTCCCCTCACAAACAATCGTTATTTATATTGT<br>TAATTAGCAAACACAAAACCTGC
GU261711	DBGU261711_China_Hap_F5	ATTTTATTTTTTAACCTAACTCCCCTACTAAGTGTACCCCCCCTTTCCCCCCCAGGGGGGGTATACTATGCATAATCGTGCATACATTTATATACCACATATATTATGGT<br>ACCGGTAATATATACTATATATGTACTAAACCCATTATATGTATACGGGCATTAATCTATATTCCACATTTCTCCCAATGTCCATTCTATGCATGATCCAGGACATACTC<br>ATTCACCCTCCCTACAGACAGCTCCAAACCACCACCAAGTCACCTAACTATGAATGGTTACAGGACATAAATCTCACTCTCATGTTCTTCCCCTACCAAGTCACCTAAC<br>TATGAATGGTTACAGGACATACATTTAACTACC--<br>ATGTTCTAACCCATTTGGTTATGCTCGCCGTATCAGATGGATTTATTGATCGTCCACCTCACGAGAGATCAGCAACCCCTGCTTGTAATGTACTTCATGACCAGTCTCA<br>GGCCCATTCTTTCCCCCTACACCCCTCGCCCTACTTGCCTTCCACCGTACCTCTGGTTCCTCGGTCAGGCACATCCCATGCATAACTCCTGAACTTTCTCACTTTTCAC<br>GAAGTCATCTGTGGATTATCTTCCCCTCTTTAGTCCGTGATCGCGGCATCTTCTCTCTTCTATTGCTGTTGGTTCCTTCTCTTTTTGGGGCTTCTTCACAGGTTGCCCTT<br>CACAGTGCGGGTGCGGAGTGCTATTCAAGTGAAGCCTGGACTACACCTGCGTTGCGTCCTATCCTAGTCCTCTCGTGTCCCTCGATGAGACGGTTTGCGTGTATGGGG<br>AATCATCTTGACACTGATGCACTTTGGATCGCATTTGGTTATGGTTCTTCCACCCCCCC--<br>GGTAAATGGTGCTATTTAGTGAATGCTTGTCGGACATATTTTTACCAATTTTCACTTCCTCTATTTTCTTCACAAAACTAGGAAATTCACCACAATTTTTTCTTTGTTATT<br>TTTTAATTTTTTTTTTTATTTTTTAAAAACATTTTTTAAAAAACTAAATTACATACAAACTACCGCATAAAATCCCTCAAACTATACAAACGTTTATCGTATAATATATATAC<br>ATTATTGTTTATTCTATCATTATTAGAGAAACTCCACTACCAAAACCATCATTAAAACAAAAATTTACATGCCACTTAACTCCCCTCACAAACAATCGTTATTTATATTGT<br>TAATTAGCAAACACAAAACCTGC

GU261712	DBGU261712_China_Hap_E1a	ATTTTATTTTTTAACCTAACTCCCCTACTAAGTGTACCCCCCCTTTCCCCCCCAGGGGGGGTATACTATGCATAATCGTGCATACATTTATATACCACATATATTATGGT<br>ACCGGTAATATATACTATATATGTACTAAACCCATTATATGTATACGGGCATTAATCTATATTCCACATTTCTCCCAATGTCCATTCTATGCATGATCCAGGACACACTC<br>ATTCACCCTCCCCATAGACAGCTCCAAACCACTACCAAGTCACCTAACTATGAATGGTTACAGGACATAAATCTCACTCTCATGTTCTTCCCCCAACAAGTCACCTAAC<br>TATGAATGGTTACAGGACATACATTTAACTACC--<br>ATGTTCTAACCCATTTGGTTATGCTCGCCGTATCAGATGGATTTATTGATCGTCCACCTCACGAGAGATCAGCAACCCCTGCTTGTAATGTACTTCATGACCAGTCTCA<br>GGCCCATTCTTTCCCCCTACACCCCTCGCCCTACTTGCCTTCCACCGTACCTCTGGTTCCTCGGTCAGGCACATCCCATGCATAACTCCTGAACTTTCTCACTTTTCAC<br>GAAGTCATCTGTGGATTATCTTCCCCTCTTTAGTCCGTGATCGCGGCATCTTCTCTCTTCTATTGCTGTTGGTTCCTTCTCTTTTTGGGGCTTCTTCACAGGTTGCCCTT<br>CACAGTGCGGGTGCGGAGTGCTATTCAAGTGAAGCCTGGACTACACCTGCGTTGCGTCCTATCCTAGTCCTCTCGTGTCCCTCGATGAGACGGTTTGCGTGTATGGGG<br>AATCATCTTGACACTGATGCACTTTGGATCGCATTTGGTTATGGTTCTTCCACCCCCCC--<br>GGTAAATGGTGCTATTTAGTGAATGCTTGTCGGACATATTTTTATCAATTTTCACTTCCTCTATTTTCTTCACAAAACTAGGAAATTCACCACAATTTTTTCTTTGTTATT<br>TTTTAATTTTTTTTTTATTTTTTAAAAACATTTTTTAAAAAACTAAATTACATACAAACTACCGCATAAAATCCCTCAAACTATACAAACGTTTATCGTATAATATATATAC<br>ATTATTGTTTATTCTATCATTATTAGAGAAACTCCACTACCAAAACCATCATTAAAACAAAAATTTACATGCCACTTAACTCCCCTCACAAACAATCGTTATTTATATTGT<br>TAATTAGCAAACACAAAACCTGC
GU261713	DBGU261713_China_Hap_E1	ATTTTATTTTTTAACCTAACTCCCCTACTAAGTGTACCCCCCCTTTCCCCCCCAGGGGGGGTATACTATGCATAATCGTGCATACATTTATATACCACATATATTATGGT<br>ACCGGTAATATATACTATATATGTACTAAACCCATTATATGTATACGGGCATTAATCTATATTCCACATTTCTCCCAATGTCCATTCCATGCATGATCCAGGACACACTC<br>ATTCACCCTCCCCATAGACAGCTCCAAACCACTACCAAGTCACCTAACTATGAATGGTTACAGGACATAAATCTCACTCTCATGTTCTTCCCCCAACAAGTCACCTAAC<br>TATGAATGGTTACAGGACATACATTTAACTACC--<br>ATGTTCTAACCCATTTGGTTATGCTCGCCGTATCAGATGGATTTATTGATCGTCCACCTCACGAGAGATCAGCAACCCCTGCTTGTAATGTACTTCATGACCAGTCTCA<br>GGCCCATTCTTTCCCCCTACACCCCTCGCCCTACTTGCCTTCCACCGTACCTCTGGTTCCTCGGTCAGGCACATCCCATGCATAACTCCTGAACTTTCTCACTTTTCAC<br>GAAGTCATCTGTGGATTATCTTCCCCTCTTTAGTCCGTGATCGCGGCATCTTCTCTCTTCTATTGCTGTTGGTTCCTTCTCTTTTTGGGGCTTCTTCACAGGTTGCCCTT<br>CACAGTGCGGGTGCGGAGTGCTATTCAAGTGAAGCCTGGACTACACCTGCGTTGCGTCCTATCCTAGTCCTCTCGTGTCCCTCGATGAGACGGTTTGCGTGTATGGGG<br>AATCATCTTGACACTGATGCACTTTGGATCGCATTTGGTTATGGTTCTTCCACCCCCCC--<br>GGTAAATGGTGCTATTTAGTGAATGCTTGTCGGACATATTTTTATCAATTTTCACTTCCTCTATTTTCTTCACAAAACTAGGAAATTCACCACAATTTTTTCTTTGTTATT<br>TTTTAATTTTTTTTTTATTTTTTAAAAACATTTTTTAAAAAACTAAATTACATACAAACTACCGCATAAAATCCCTCAAACTATACAAACGTTTATCGTATAATATATATAC<br>ATTATTGTTTATTCTATCATTATTAGAGAAACTCCACTACCAAAACCATCATTAAAACAAAAATTTACATGCCACTTAACTCCCCTCACAAACAATCGTTATTTATATTGT<br>TAATTAGCAAACACAAAACCTGC
GU261714	DBGU261714_China_Hap_B1a	ATTTTATTTTTTAACCTAACTCCCCTACTAAGTGTACCCCCCCTTTCCCCCCCAGGGGGGGTATACTATGCATAATCGTGCATACATTTATATACCACATATATTATGGT<br>ACCGGTAATATATACTATATATGTACTAAACCCATTATATGTATACGGGCATTAATCTATATTCCACATTTCTCCCAATGTCCATTCTATGCATGATCCAAGACATACTC<br>ATTCACCCTCCCCATAGACAGTTCTAAACCACTATCAAGCCACCTAACTATGAATGGTTACAGGACATAAATCTTACTCTCATGTTCTCCCCCTAACAAGTCACCTAAC<br>TATGAATGGTTACAGGACATACATTTAACTACC--<br>ATGTTCTAACCCATTTGGTTATGCTCGCCGTATCAGATGGATTTATTGATCGTCCACCTCACGAGAGATCAGCAACCCCTGCCTGTAATGTACTTCATGACCAGTCTCA<br>GGCCCATTCTTTCCCCCTACACCCCTCGCCCTACTTGCCTTCCACCGTACCTCTGGTTCCTCGGTCAGGCACATCCCATGCATAACTCCTGAACTTTCTCACTTTTCAC<br>GAAGTCATCTGTGGATTATCTTCCCCTCTTTAGTCCGTGATCGCGGCATCTTCTCTCTTCTATTGCTGTTGGTTCCTTCTCTTTTTGGGGCTTCTTCACAGGTTGCCCTT<br>CACAGTGCGGGTGCGGAGTGCTATTCAAGTGAAGCCTGGACTACACCTGCGTTGCGTCCTATCCTAGTCCTCTCGTGTCCCTCGATGAGACGGTTTGCGTATATGGGG<br>AATCATCTTGACACTGATGCACTTTGGATCGCATTTGGTTATGGTTCTTCCACCCCCCCC-<br>GGTAAATGGTGCTATTTAGTGAATGCTTGTCGGACATATTTTTATCAATTTTCACTTCCTCTATTTTCTTCACAAAACTAGGAAATTCACCACAATTTTTTCTTTGTTATT<br>TTTTAATTTTTTTTTTATTTTTTAAAAACATTTTTTAAAAAACTAAATTACATACAAACTACCGCATAAAATCCCTCAAACTATACAAACGTTTATCGTATAATATATATAC<br>ATTATTGTTTATTCTATCATTATTAGAGAAACTCCACTACCAAAACCATCATTAAAACAAAAATTTACATGCCACTTAACTCCCCTCACAAACAATCGTTATTTATATTGT<br>TAATTAGCAAACACAAAACCCAC

GU261715	DBGU261715_China_Hap_H	ATTTTATTTTTTAACCTAACTCCCCTACTAAGTGTACCCCCCCTTTCCCCCCCAGGGGGGGTATACTATGCATAATCGTGCATACATTTATATACCACATATATTATGGT<br>ACCGGTAATATATACTATATATGTACTAAACCCATTATATGTATACGGGCATTAATCTATATTCCACATTTCTCCCAATGTCCATTTCATGCATGATCCAGGACATACTC<br>ATTCACCCTCCCCATAGACAGCTCCAAACCACTACCAAGTCACCTAACTATGAATGGTTGCAGGACATAAATCTCACTCTCATGCTCTTCCCCCAACAAGTCACCTAAC<br>TATGAATGGTTACAGGACATACATTTAACTACT--<br>ATGTTCTAACCCATTTGGTTATGCTCGTCGTATCAGATGGATTTATTGATCGTCCACCTCACGAGAGATCAGCAACCCCTGCTTGTAATGTACTTCATGACCAGTCTCA<br>GGCCCATTCTTTCCCCCTACACCCCTCGCCCTACTTGCCTTCCACCGTACCTCTGGTTCCTCGGTCAGGCACATCCCATGCATAACTCCTGAACTTTCTCACTTTTCAC<br>GAAGTCATCTGTGGATTATCTTCCCCTCTTTAGTCCGTGATCGCGGCATCTTCTCTCTTCTATTGCTGTTGGTTCCTTCTCTTTTTGGGGCTTCTTCACAGGTTGCCCTT<br>CACAGTGCGGGTGCGGAGTGCTATTCAAGTGAAGCCTGGACTACACCTGCGTTGCGTCCTATCCTAGTCCTCTCGTGTCCCTCGATGAGACGGTTTGCGTGTATGGGG<br>AATCATCTTGACACTGATGCACTTTGGATCGCATTTGGTTATGGTTCTTCCACCCCCCCC-<br>GGTAAATGGTGCTATTTAGTGAATGCTTGTCGGACATATTTTTATCAATTTTCACTTCCTCTATTTTCTTCACAAAACTAGGAAATTCACCACAATTTTTTCTTTGTTATT<br>TTTTAATTTTTTTTTTATTTTTTAAAAACATTTTTTAAAAAACTAAATTACATACAAACTACCGCATAAAATCCCTCAAACTATACAAACGTTTATCGTATAATATATATAC<br>ATTATTGTTTATTCTATCATTATTAGAGAAACTCCACTACCAAAACCATCATTAAAACAAAAATTTACATGCCACTTAACTCCCCTCACAAACAATCGTTATTTATATTGT<br>TAATTAGCAAACACAAAACCCGC
GU261716	DBGU261716_Myanmar_Hap_C3	ATTTTATTTTTTAACCTAACTCCCCTACTAAGTGTACCCCCCCTTTCCCCCCCAGGGGGGGTATACTATGCATAATCGTGCATACATTTATATACCACATATATTATGGT<br>ACCGGTAATATATACTATATATGTACTAAACCCATTATATGTATACGGGCATTAATCTATATTCCTCATTTCTCCCAATGTCCATTCTATGCATGATCCAGGACATACTC<br>ATTCACCCTCCCCATAGACAGCTCCAAACCACTACCAAGTCACTTAACTATGAATGGTTGCAGGACATAAATCTCACTCTCATGCTCTTCCCCCAACAAGTCACCTAAC<br>TATGAATGGTTGCAGGACATACATTTAACTACT--<br>ATGTTCTAACCCATTTGGTTATGCTCGCCGTATCAGATGGATTTATTGATCGTCCACCTCACGAGAGATCAGCAACCCCTGCCTGTAATGTACTTCATGACCAGTCTCA<br>GGCCCATTCTTTCCCCCTACACCCCTCGCCCTACTTGCCTTCCACCGTACCTCTGGTTCCTCGGTCAGGCACATCCCATGCATAACTCCTGAACTTTCTCACTTTTCAC<br>GAAGTCATCTGTGGATTATCTTCCCCTCTTTAGTCCGTGATCGCGGCATCTTCTCTCTTCTATTGCTGTTGGTTCCTTCTCTTTTTGGGGCTTCTTCACAGGTTACCCTT<br>CACAGTGCGGGTGCGGAGTGCTATTCAAGTGAAGCCTGGACTACACCTGCGTTGCGTCCTATCCTAGTCCTCTCGTGTCCCTCGATGAGACGGTTTGCGTGTATGGGG<br>AATCATCTTGACACTGATGCACTTTGGATCGCATTTGGTTATGGTTCTTCCACCCCCCC--<br>GGTAAATGGTGCTATTTAGTGAATGCTTGTCGGACATATTTTTATCAATTTTCACTTCCTCTATTTTCTTCACAAAACTAGGAAATTCACCACAATTTTTTCTTTGTTATT<br>TTTTAATTTTTTTTTTATTTTTTAAAAACATTTTTTAAAAAACTAAATTACATACAAACTACCGCATAAAATCCCTCAAACTATACAAACGTTTATCGTATAATATATATAC<br>ATTATTGTTTATTCTATCATTATTAGAGAAACTCCACTACCAAAACCATCATTAAAACAAAAATTTACATGCCACTTAACTCCCCTCACAAACAATCGTTATTTATATTGT<br>TAATTAGCAAACACAAAACCCGC
GU261717	DBGU261717_China_Hap_F3	ATTTTATTTTTTAACCTAACTCCCCTACTAAGTGTACCCCCCCTTTCCCCCCCAGGGGGGGTATACTATGCATAATCGTGCATACATTTATATACCACATATATTATGGT<br>ACCGGTAATATATACTATATATGTACTAAACCCATTATATGTATACGGGCATTAATCTATATTCCACATTTCTCCCAATGTCCATTCTATGCATGATCCAGGACATACTC<br>ATTCACCCTCCCTACAGACAGCTCTAAACCACCACCAAGTCACCTAACTATGAATGGTTACAGGACATAAATCTCACTCTCATGTTCTTCCCCTACCAAGTCACCTAAC<br>TATGAATGGTTACAGGACATACATTTAACTACC--<br>ATGTTCTAACCCATTTGGTTATGCTCGCCGTATCAGATGGATTTATTGATCGTCCACCTCACGAGAGATCAGCAACCCCTGCTTGTAATGTACTTCATGACCAGTCTCA<br>GGCCCATTCTTTCCCCCTACACCCCTCGCCCTACTTGCCTTCCACCGTACCTCTGGTTCCTCGGTCAGGCACATCCCATGCATAACTCCTGAACTTTCTCACTTTTCAC<br>GAAGTCATCTGTGGATTATCTTCCCCTCTTTAGTCCGTGATCGCGGCATCTTCTCTCTTCTATTGCTGTTGGTTCCTTCTCTTTTTGGGGCTTCTTCACAGGTTACCCTT<br>CACAGTGCGGGTGCGGAGTGCTATTCAAGTGAAGCCTGGACTACACCTGCGTTGCGTCCTATCCTAGTCCTCTCGTGTCCCTCGATGAGACGGTTTGCGTGTATGGGG<br>AATCATCTTGACACTGATGCACTTTGGATCGCATTTGGTTATGGTTCTTCCACCCCCCC--<br>GGTAAATGGTGCTATTTAGTGAATGCTTGTCGGACATATTTTTACCAATTTTCACTTCCTCTATTTTCTTCACAAAACTAGGAAATTCACCACAATTTTTTCTTTGTTATT<br>TTTTAATTTTTTTTTTATTTTTTAAAAACATTTTTTAAAAAACTAAATTACATACAAACTACCGCATAAAATCCCTCAAACTATACAAACGTTTATCGTATAATATATATAC<br>ATTATTGTTTATTCTATCATTATTAGAGAAACTCCACTACCAAAACCATCATTAAAACAAAAATTTACATGCCACTTAACTCCCCTCACAAACAATCGTTATTTATATTGT<br>TAATTAGCAAACACAAAACCTGC

GU261718	DBGU261718_China_Hap_C1b	ATTTTATTTTTTAACCTAACTCCCCTACTAAGTGTACCCCCCCCTTTCCCCCCCAGGGGGGGTATACTATGCATAATCGTGCATACATTTATATACCACATATATTATGGT<br>ACCGGTAATATATACTATATATGTACTAAACCCATTATATGTATACGGGCATTAATCTATATTCCACATTTCTCCCAATGTCCATTCTATGCATGATCCAGGACATACTC<br>ATTCACCCTCCCCATAGACAACTCCAAACCACTACCAAGCCACCTAACTATGAATGGTTGCAGGACATAAATCTCACTCTCATGTTCTTCCCCCAACAAGTCACCTAAC<br>TATGAATGGTTGCAGGACATACATTTAACTACT---<br>ATGCTCTAACCCATTTGGTTATGCTCGCCGTATCAGATGGATTTATTGATCGTCCACCTCACGAGAGATCAGCAACCCCTGCCTGTAATGTACTTCATGACCAGTCTCA<br>GGCCCATTCTTTCCCCCTACACCCCTCGCCCTACTTGCCTTCCACCGTACCTCTGGTTCCTCGGTCAGGCACATCCCATGCATAACTCCTGAACTTTCTCACTTTTCAC<br>GAAGTCATCTGTGGATTATCTTCCCCTCTTTAGTCCGTGATCGCGGCATCTTCTCTCTTCTATTGCTGTTGGTTCCTTCTCTTTTTGGGGCTTCTTCACAGGTTGCCCTT<br>CACAGTGCGGGTGCGGAGTGCTATTCAAGTGAAGCCTGGACTACACCTGCGTTGCGTCCTATCCTAGTCCTCTCGTGTCCCTCGATGAGACGGTTTGCGTGTATGGGG<br>AATCATCTTGACACTGATGCACTTTGGATCGCATTTGGTTATGGTTCTTCCACCCCCCCC-<br>GGTAAATGGTGCTATTTAGTGAATGCTTGTCGGACATATTTTTATCAATTTTCACTTCCTCTATTTTCTTCACAAAACTAGGAAATTCACCACAATTTTTTCTTTGTTATT<br>TTTTAATTTTTTTTTTATTTTTTAAAAACATTTTTTAAAAAACTAAATTACATACAAACTACCGCATAAAATCCCTCAAACTATACAAACGTTTATCGTATAATATATATAC<br>ATTATTGTTTATTCTATCATTATTAGAGAAACTCCACTACCAAAACCATCATTAAAACAAAAATTTACATGCCACTTAACTCCCCTCACAAACAATCGTTATTTATATTGT<br>TAATTAGCAAACACAAAACCCGC
GU261719	DBGU261719_China_Hap_G1a	ATTTTATTTTTTAACCTAACTCCCCTACTAAGTGTACCCCCCCCTTTCCCCCCCAGGGGGGGTATACTATGCATAATCGTGCATACATTTATATACCACATATATTATGGT<br>ACCGGTAATATATACTATATATGTACTAAACCCATTATATGTATACGGGCATTAATCTATATTCCACATTTCTCCCAATGTCCATTCTATGCATGATCCAAGACATACTC<br>ATTCACCCTCCCCATAGACAGCTCCAAACCACTACCAAGTCACCTAACTATGAATGGTTACAGGACATAAATCTAACTCTTATGTTCTTCCCCTAACAAGCCACCTAAC<br>TATGAATGGTTACAGGACATACATTTAACTACC---<br>ATGTTCTAACCCATTTGGTTATGCTCGCCGTATCAGATGGATTTATTGATCGTCCACCTCACGAGAGATCAGCAACCCCTGCTTGTAATGTACTTCATGACCAGTCTCA<br>GGCCCATTCTTTCCCCCTACACCCCTCGCCCTACTTGCCTTCCACCGTACCTCTGGTTCCTCGGTCAGGCACATCCCATGCATAACTCCTGAACTTTCTCACTTTTCAC<br>GAAGTCATCTGTGGATTATCTTCCCCTCTTTAGTCCGTGATCGCGGCATCTTCTCTCTTCTATTGCTGTTGGTTCCTTCTCTTTTTGGGGCTTCTTCACAGGTTGCCCTT<br>CACAGTGCGGGTGCGGAGTGCTATTCAAGTGAAGCCTGGACTACACCTGCGTTGCGTCCTATCCTAGTCCTCTCGTGTCCCTCGATGAGACGGTTTGCGTGTATGGGG<br>AATCATCTTGACACTGATGCACTTTGGATCGCATTTGGTTATGGTTCTTCCACCCCCCC--<br>GGTAAATGGTGCTATTTAGTGAATGCTTGTCGGACATATTTTTATCAATTTTCACTTCCTCTATTTTCTTCACAAAACTAGGAAATTCACCACAATTTTTTCTTTGTTATT<br>TTTTAATTTTTTTTTTATTTTTTAAAAACATTTTTTAAAAAACTAAATTACATACAAACTACCGCATAAAATCCCTCAAACTATACAAACGTTTATCGTATAATATATATAC<br>ATTATTGTTTATTCTATCATTATTAGAGAAACTCCACTACCAAAACCATCATTAAAACAAAAATTTACATGCCACTTAACTCCCCTCACAAACAATCGTTATTTATATTGT<br>TAATTAGCAAACACAAAACCTGC
HQ857209	DBHQ857209_India_Hap_E2	ATTTTATTTTTTAACCTAACTCCCCTACTAAGTGTACCCCCCCCTTTCCCCCCCAGGGGGGGTATACTATGCATAATCGTGCATACATTTATATACCACATATATTATGGT<br>ACCGGTAATATATACTATATATGTACTAAACCCATTATATGTATACGGGCATTAATCTATATTCCACATTTCTCCCAATGTCCATTCTATGCATGATCCAGGACACACTC<br>ATCCACCCTCCCCATAGACAGCTCTAAACCACTACCAAGTCACCTAACTATGAATGGTTACAGGACATAAATCTCACTCTCATGTTCTTCCCCCAACAAGTCACCTAAC<br>TATGAATGGTTACAGGACATACATTTAACTACC---<br>ATGTTCTAACCCATTTGGTTATGCTCGCCGTATCAGATGGATTTATTGATCGTCCACCTCACGAGAGATCAGCAACCCCTGCTTGTAATGTACTTCATGACCAGTCTCA<br>GGCCCATTCTTTCCCCCTACACCCCTCGCCCTACTTGCCTTCCACCGTACCTCTGGTTCCTCGGTCAGGCACATCCCATGCATAACTCCTGAACTTTCTCACTTTTCAC<br>GAAGTCATCTGTGGATTATCTTCCCCTCTTTAGTCCGTGATCGCGGCATCTTCTCTCTTCTATTGCTGTTGGTTCCTTCTCTTTTTGGGGCTTCTTCACAGGTTGCCCTT<br>CACAGTGCGGGTGCGGAGTGCTATTCAAGTGAAGCCTGGACTACACCTGCGTTGCGTCCTATCCTAGTCCTCTCGTGTCCCTCGATGAGACGGTTTGCGTGTATGGGG<br>AATCATCTTGACACTGATGCACTTTGGATCGCATTTGGTTATGGTTCTTCCACCCCCCC--<br>GGTAAATGGTGCTATTTAGTGAATGCTTGTCGGACATATTTTTATCAATTTTCACTTCCTCTATTTTCTTCACAAAACTAGGAAATTCACCACAATTTTTTCTTTGTTATT<br>TTTTAATTTTTTTTTTATTTTTTAAAAACATTTTTTAAAAAACTAAATTACATACAAACTACCGCATAAAATCCCTCAAACTATACAAACGTTTATCGTATAATATATATAC<br>ATTATTGTTTATTCTATCATTATTAGAGAAACTCCACTACCAAAACCATCATTAAAACAAAAATTTACATGCCACTTAACTCCCCTCACAAGCAATCGTTATTTATATTGT<br>TAATTAGCAAACACAAAACCTGC

HQ857210	DBHQ857210_India_Hap_E1c	ATTTTATTTTTTAACCTAACTCCCCTACTAAGTGTACCCCCCCTTTCCCCCCCAGGGGGGGTATACTATGCATAATCGTGCATACATTTATATACCACATATATTATGGT<br>ACCGGTAATATATACTATATATGTACTAAACCCATTATATGTATACGGGCATTAATCTATATTCCACATTTCTCCCAATGTCCATTCTATGCATGGTCCAGGACACACTC<br>ATTCACCCTCCCCATAAACAGCTCTAAACCACTACCAAGTCACCTAACTATGAATGGTTACAGGACATAAATCTCACTCTCATGTTCTTCCCCCAACAAGTCACCTAAT<br>TATGAATGGTTACAGGACATACATTTAACTACC--<br>ATGTTCTAACCCATTTGGTTATGCTCGCCGTATCAGATGGATTTATTGATCGTCCACCTCACGAGAGATCAGCAACCCCTGCTTGTAATGTACTTCATGACCAGTCTCA<br>GGCCCATTCTTTCCCCCTACACCCCTCGCCCTACTTGCCTTCCACCGTACCTCTGGTTCCTCGGTCAGGCACATCCCATGCATAACTCCTGAACTTTCTCACTTTTCAC<br>GAAGTCATCTGTGGATTATCTTCCCCTCTTTAGTCCGTGATCGCGGCATCTTCTCTCTTCTATTGCTGTTGGTTCCTTCTCTTTTTGGGGCTTCTTCACAGGTTGCCCTT<br>CACAGTGCGGGTGCGGAGTGCTATTCAAGTGAAGCCTGGACTACACCTGCGTTGCGTCCTATCCTAGTCCTCTCGTGTCCCTCGATGAGACGGTTTGCGTGTATGGGG<br>AATCATCTTGACACTGATGCACTTTGGATCGCATTTGGTTATGGTTCTTCCACCCCCCC--<br>GGTAAATGGTGCTATTTAGTGAATGCTTGTCGGACATATTTTTATCAATTTTCACTTCCTCTATTTTCTTCACAAAACTAGGAAATTCACCACAATTTTTTCTTTGTTATT<br>TTTTAATTTTTTTTTTATTTTTTAAAAACATTTTTTAAAAAACTAAATTACATACAAACTACCGCATAAAATCCCTCAAACTATACAAACGTTTATCGTATAATATATATAC<br>ATTATTGTTTATTCTATCATTATTAGAGAAACTCCACTACCAAAACCATCATTAAAACAAAAATTTACATGCCACTTAACTCCCCTCACAAACAATCGTTATTTATATTGT<br>TAATTAGCAAACACAAAACCTGC
HQ857211	DBHQ857211_India_Hap_E3a	ATTTTATTTTTTAACCTAACTCCCCTACTAAGTGTACCCCCCCTTTCCCCCCCAGGGGGGGTATACTATGCATAATCGTGCATACATTTATATACCACATATATTATGGT<br>ACCGGTAATATATACTATATATGTACTAAACCCATTATATGTATACGGGCATTAATCTATATTCCACATTTCTCCCAATGTCCATTCTATGCATGATCCAGGACACACTC<br>GTTCACCCTCCCCATAGACAGCTCCAAGCCACTACCAAGTCACCTAACTATGAATGGTTGCAGGACATAAATCTCACTCTCATGTTCTTCCCCCAACAAGTCACCTAAC<br>TATGAATGGTTACAGGACATACATCTAACTACC--<br>ATGTTCTAACCCATTTGGTTATGCTCGCCGTATCAGATGGATTTATTGATCGTCCACCTCACGAGAGATCAGCAACCCCTGCTTGTAATGTACTTCATGACCAGTCTCA<br>GGCCCATTCTTTCCCCCTACACCCCTCGCCCTACTTGCCTTCCACCGTACCTCTGGTTCCTCGGTCAGGCACATCCCATGCATAACTCCTGAACTTTCTCACTTTTCAC<br>GAAGTCATCTGTGGATTATCTTCCCCTCTTTAGTCCGTGATCGCGGCATCTTCTCTCTTCTATTGCTGTTGGTTCCTTCTCTTTTTGGGGCTTCTTCACAGGTTACCCTT<br>CACAGTGCGGGTGCGGAGTGCTATTCAAGTGAAGCCTGGACTACACCTGCGTTGCGTCCTATCCTAGTCCTCTCGTGTCCCTCGATGAGACGGTTTGCGTGTATGGGG<br>AATCATCTTGACACTGATGCACTTTGGATCGCATTTGGTTATGGTTCTTCCACCCCCCCC-<br>GGTAAATGGTGCTATTTAGTGAATGCTTGTCGGACATATTTTTATCAATTTTCACTTCCTCTATTTTCTTCACAAAACTAGGAAATTCACCACAATTTTTTCTTTGTTATT<br>TTTTAATTTTTTTTTTATTTTTTAAAAACATTTTTTAAAAAACTAAATTACATACAAACTACCGCATAAAATCCCTCAAACTATACAAACGTTTATCGTATAATATATATAC<br>ATTATTGTTTATTCTATCATTATTAGAGAAACTCCACTACCAAAACCATCATTAAAACAAAAATTTACATGCCACTTAACTCCCCTCACAAACAATCGTTATTTATATTGT<br>TAATTAGCAAACACAAAACCTGC
HQ857212	DBHQ857212_India_Hap_E3a	ATTTTATTTTTTAACCTAACTCCCCTACTAAGTGTACCCCCCCTTTCCCCCCCAGGGGGGGTATACTATGCATAATCGTGCATACATTTATATACCACATATATTATGGT<br>ACCGGTAATATATACTATATATGTACTAAACCCATTATATGTATACGGGCATTAATCTATATTCCACATTTCTCCCAATGTCCATTCTATGCATGATCCAGGACACACTC<br>GTTCACCCTCCCCATAGACAGCTCCAAGCCACTACCAAGTCACCTAACTATGAATGGTTACAGGACATAAATCTCACTCTCATGTTCTTCCCCCAACAAGTCACCTAAC<br>TATGAATGGTTACAGGACATACATCTAACTACC--<br>ATGTTCTAACCCATTTGGTTATGCTCGCCGTATCAGATGGATTTATTGATCGTCCACCTCACGAGAGATCAGCAACCCCTGCTTGTAATGTACTTCATGACCAGTCTCA<br>GGCCCATTCTTTCCCCCTACACCCCTCGCCCTACTTGCCTTCCACCGTACCTCTGGTTCCTCGGTCAGGCACATCCCATGCATAACTCCTGAACTTTCTCACTTTTCAC<br>GAAGTCATCTGTGGATTATCTTCCCCTCTTTAGTCCGTGATCGCGGCATCTTCTCTCTTCTATTGCTGTTGGTTCCTTCTCTTTTTGGGGCTTCTTCACAGGTTGCCCTT<br>CACAGTGCGGGTGCGGAGTGCTATTCAAGTGAAGCCTGGACTACACCTGCGTTGCGTCCTATCCTAGTCCTCTCGTGTCCCTCGATGAGACGGTTTGCGTGTATGGGG<br>AATCATCTTGACACTGATGCACTTTGGATCGCATTTGGTTATGGTTCTTCCACCCCCCCC-<br>GGTAAATGGTGCTATTTAGTGAATGCTTGTCGGACATATTTTTATCAATTTTCACTTCCTCTATTTTCTTCACAAAACTAGGAAATTCACCACAATTTTTTCTTTGTTATT<br>TTTTAATTTTTTTTTTATTTTTTAAAAACATTTTTTAAAAAACTAAATTACATACAAACTACCGCATAAAATCCCTCAAACTATACAAACGTTTATCGTATAATATATATAC<br>ATTATTGTTTATTCTATCATTATTAGAGAAACTCCACTACCAAAACCATCATTAAAACAAAAATTTACATGCCACTTAACTCCCCTCACAAACAATCGTTATTTATATTGT<br>TAATTAGCAAACACAAAACCTGC

KF826490	DBKF826490_China_Hap_E1c	ATTTTATTTTTTAACCTAACTCCCCTACTAAGTGTACCCCCCCTTTCCCCCCCAGGGGGGGTATACTATGCATAATCGTGCATACATTTATATACCACATATATTATGGT<br>ACCGGTAATATATACTATATATGTACTAAACCCATTATATGTATACGGGCATTAATCTATATTCCACATTTCTCCCAATGTCCATTCTATGCATGATCCAGGACACACTC<br>GTTCACCCTCCCCATAGACAGCTCCAAACCACTACCAAGTCACCTAACTATGAATGGTTACAGGACATAAATCTCACTCTCATGTTCTTCCCCCAACAAGTCACCTAAT<br>TATGAATGGTTACAGGACATACATTTAACTACC--<br>ATGTTCTAACCCATTTGGTTATGCTCGCCGTATCAGATGGATTTATTGATCGTCCACCTCACGAGAGATCAGCAACCCCTGCTTGTAATGTACTTCATGACCAGTCTCA<br>GGCCCATTCTTTCCCCCTACACCCCTCGCCCTACTTGCCTTCCACCGTACCTCTGGTTCCTCGGTCAGGCACATCCCATGCATAACTCCTGAACTTTCTCACTTTTCAC<br>GAAGTCATCTGTGGATTATCTTCCCCTCTTTAGTCCGTGATCGCGGCATCTTCTCTCTTCTATTGCTGTTGGTTCCTTCTCTTTTTGGGGCTTCTTCACAGGTTGCCCTT<br>CACAGTGCGGGTGCGGAGTGCTATTCAAGTGAAGCCTGGACTACACCTGCGTTGCGTCCTATCCTAGTCCTCTCGTGTCCCTCGATGAGACGGTTTGCGTGTATGGGG<br>AATCATCTTGACACTGATGCACTTTGGATCGCATTTGGTTATGGTTCTTCCACCCCCCC--<br>GGTAAATGGTGCTATTTAGTGAATGCTTGTCGGACATATTTTTATCAATTTTCACTTCCTCTATTTTCTTCACAAAACTAGGAAATTCACCACAATTTTTTCTTTGTTATT<br>TTTTAATTTTTTTTTTATTTTTTAAAAACATTTTTTAAAAAACTAAATTACATACAAACTACCGCATAAAATCCCTCAAACTATACAAACGTTTATCGTATAATATATATAC<br>ATTATTGTTTATTCTATCATTATTAGAGAAACTCCACTACCAAAACCATCATTAAAACAAAAATTTACATGCCACTTAACTCCCCTCACAAACAATCGTTATTTATATTGT<br>TAATTAGCAAACACAAAACCTGC
KF939304	DBKF939304_China_Hap_D1	ATTTTATTTTTTAACCTAACTCCCCTACTAAGTGTACCCCCCCTTTCCCCCCCAGGGGGGGTATACTATGCATAATCGTGCATACATTTATATACCACATATATTATGGT<br>ACCGGTAATATATACTATATATGTACTAAACCCATTATATGTATACGGGCATTAATCTATATTCCACATTTCTCCCAATGTCCATTCTATGCATGATCCAGGACATACTC<br>ATTCACCCTCCCCATAGACAGCTCCAAACCACTACCAAGTCACCTAACTATGAATGGTTGCAGGACATAAATCTCACTCTCATGCTCTTCCCCCAACAAGTCACCTAAC<br>TATGAATGGTTACAGGACATACATTTAACTACC--<br>ATGTTCTAACCCATTTGGTTATGCTCGCCGTATCAGATGGATTTATTGATCGTCCACCTCACGAGAGATCAGCAACCCCTGCCTGTAATGTACTTCATGACCAGTCTCA<br>GGCCCATTCTTTCCCCCTACACCCCTCGCCCTACTTGCCTTCCACCGTACCTCTGGTTCCTCGGTCAGGCACATCCCATGCATAACTCCTGAACTTTCTCACTTTTCAC<br>GAAGTCATCTGTGGATTATCTTCCCCTCTTTAGTCCGTGATCGCGGCATCTTCTCTCTTCTATTGCTGTTGGTTCCTTCTCTTTTTGGGGCTTCTTCACAGGTTGCCCTT<br>CACAGTGCGGGTGCGGAGTGCTATTCAAGTGAAGCCTGGACTACACCTGCGTTGCGTCCTATCCTAGTCCTCTCGTGTCCCTCGATGAGACGGTTTGCGTGTATGGGG<br>AATCATCTTGACACTGATGCACTTTGGATCGCATTTGGTTATGGTTCTTCCACCCCCCCC-<br>GGTAAATGGTGCTATTTAGTGAATGCTTGTCGGACATATTTTTATCAATTTTCACTTCCTCTATTTTCTTCACAAAACTAGGAAATTCACCACAATTTTTTCTTTGTTATT<br>TTTTAATTTTTTTTTTATTTTTTAAAAACATTTTTTAAAAAACTAAATTACATACAAACTACCGCATAAAATCCCTCAAACTATACAAACGTTTATCGTATAATATATATAC<br>ATTATTGTTTATTCTATCATTATTAGAGAAACTCCACTACCAAAACCATCATTAAAACAAAAATTTACATGCCACTTAACTCCCCTCACAAACAATCGTTATTTATATTGT<br>TAATTAGCAAACACAAAACCCGC
KF954727	DBKF954727_China_Hap_E1a	ATTTTATTTTTTAACCTAACTCCCCTACTAAGTGTACCCCCCCTTTCCCCCCCAGGGGGGGTATACTATGCATAATCGTGCATACATTTATATACCACATATATTATGGT<br>ACCGGTAATATATACTATATATGTACTAAACCCATTATATGTATACGGGCATTAATCTATATTCCACATTTCTCCCAATGTCCATTCTATGCATGATCCAGGACACACTC<br>ATTCACCCTCCCCATAGACAGCTCCAAACCACTACCAAGTCACCTAACTATGAATGGTTACAGGACATAAATCTCACTCTCATGTTCTTCCCCCAACAAGTCACCTAAC<br>TATGAATGGTTACAGGACATACATTTAACTACC--<br>ATGTTCTAACCCATTTGGTTATGCTCGCCGTATCAGATGGATTTATTGATCGTCCACCTCACGAGAGATCAGCAACCCCTGCTTGTAATGTACTTCATGACCAGTCTCA<br>GGCCCATTCTTTCCCCCTACACCCCTCGCCCTACTTGCCTTCCACCGTACCTCTGGTTCCTCGGTCAGGCACATCCCATGCATAACTCCTGAACTTTCTCACTTTTCAC<br>GAAGTCATCTGTGGATTATCTTCCCCTCTTTAGTCCGTGATCGCGGCATCTTCTCTCTTCTATTGCTGTTGGTTCCTTCTCTTTTTGGGGCTTCTTCACAGGTTGCCCTT<br>CACAGTGCGGGTGCGGAGTGCTATTCAAGTGAAGCCTGGACTACACCTGCGTTGCGTCCTATCCTAGTCCTCTCGTGTCCCTCGATGAGACGGTTTGCGTGTATGGGG<br>AATCATCTTGACACTGATGCACTTTGGATCGCATTTGGTTATGGTTCTTCCACCCCCCC--<br>GGTAAATGGTGCTATTTAGTGAATGCTTGTCGGACATATTTTTATCAATTTTCACTTCCTCTATTTTCTTCACAAAACTAGGAAATTCACCACAATTTTTTCTTTGTTATT<br>TTTTAATTTTTTTTTTATTTTTTAAAAACATTTTTTAAAAAACTAAATTACATACAAACTACCGCATAAAATCCCTCAAACTATACAAACGTTTATCGTATAATATATATAC<br>ATTATTGTTTATTCTATCATTATTAGAGAAACTCCACTACCAAAACCATCATTAAAACAAAAATTTACATGCCACTTAACTCCCCTCACAAACAATCGTTATTTATATTGT<br>TAATTAGCAAACACAAAACCTGC

KF981434	DBKF981434_China_Hap_A	ATTTTATTTTTTAACCTAACTCCCCTACTAAGTGTACCCCCCCTTTCCCCCCCAGGGGGGGTATACTATGCATAATCGTGCATACATTTATATACCACATATATTATGGT<br>ACCGGTAATATATACTATATATGTACTAAACCCATTATATGTATACGGGCATTAACCTATATTCCACATTTCTCCCAATGTCCATTCTATGCATGATCCAGGACATACTC<br>ATTTACCCTCCCCATAGATAGTTCCAAACCACTATCAAGCCACCTAACTATGAATGGTTACAGGACATAAATCTCACTCTCATGTTCTCCCCCCAACAAGTCACCTAAC<br>TATGAATGGTTACAGGACATACATTTAACTACC--<br>ATGTTCTAACCCATTTGGTTATGCTCGCCGTATCAGATGGATTTATTGATCGTCCACCTCACGAGAGATCAGCAACCCCTGCCTGTAATGTACTTCATGACCAGTCTCA<br>GGCCCATTCTTTCCCCCTACACCCCTCGCCCTACTTGCCTTCCACCGTACCTCTGGTTCCTCGGTCAGGCACATCCCATGCATAACTCCTGAACTTTCTCACTTTTCAC<br>GAAGTCATCTGTGGATTATCTTCCCCTCTTTAGTCCGTGATCGCGGCATCTTCTCTCTTCTATTGCTGTTGGTTCCTTCTCTTTTTGGGGCTTCTTCACAGGTTGCCCTT<br>CACAGTGCGGGTGCGGAGTGCTATTCAAGTGAAGCCTGGACTACACCTGCGTTGCGTCCTATCCTAGTCCTCTCGTGTCCCTCGATGAGACGGTTTGCGTGTATGGGG<br>AATCATCTTGACACTGATGCACTTTGGATCGCATTTGGTTATGGTTCTTCCACCCCCCC--<br>GGTAAATGGTGCTATTTAGTGAATGCTTGTCGGACATATTTTTATCAATTTTCACTTCCTCTATTTTCTTCACAAAACTAGGAAATTCACCACAATTTTTTCTTTGTTATT<br>TTTTAATTTTTTTTTTATTTTTTAAAAACATTTTTTAAAAAACTAAATTACATACAAACTACCGCATAAAATCCCTCAAACTATACAAACGTTTATCGTATAATATATATAC<br>ATTATTGTTTATTCTATCATTATTAGAGAAACTCCACTACCAAAACCATCATTAAAACAAAAATTTACATGCCACTTAACTCCCCTCACAAACAATCGTTATTTATATTGT<br>TAATTAGCAAACACAAAACCCGC
KJ778617	DBKJ778617_China_Hap_A	ATTTTATTTTTTAACCTAACTCCCCTACTAAGTGTACCCCCCCTTTCCCCCCCAGGGGGGGTATACTATGCATAATCGTGCATACATTTATATACCACATATATTATGGT<br>ACCGGTAATATATACTATATATGTACTAAACCCATTATATGTATACGGGCATTAACCTATATTCCACATTTCTCCCAATGTCCATTCTATGCATGATCCAGGACATACTC<br>ATTTACCCTCCCCATAGACAGTTCCAAACCACTATCAAGCCACCTAACTATGAATGGTTACAGGACATAAATCTCACTCTCATGTTCTCCCCCCAACAAGTCACCTAAC<br>TATGAATGGTTACAGGACATACATTTAACTACC--<br>ATGTTCTAACCCATTTGGTTATGCTCGCCGTATCAGATGGATTTATTGATCGTCCACCTCACGAGAGATCAGCAACCCCTGCCTGTAATGTACTTCATGACCAGTCTCA<br>GGCCCATTCTTTCCCCCTACACCCCTCGCCCTACTTGCCTTCCACCGTACCTCTGGTTCCTCGGTCAGGCACATCCCATGCATAACTCCTGAACTTTCTCACTTTTCAC<br>GAAGTCATCTGTGGATTATCTTCCCCTCTTTAGTCCGTGATCGCGGCATCTTCTCTCTTCTATTGCTGTTGGTTCCTTCTCTTTTTGGGGCTTCTTCACAGGTTGCCCTT<br>CACAGTGCGGGTGCGGAGTGCTATTCAAGTGAAGCCTGGACTACACCTGCGTTGCGTCCTATCCTAGTCCTCTCGTGTCCCTCGATGAGACGGTTTGCGTGTATGGGG<br>AATCATCTTGACACTGATGCACTTTGGATCGCATTTGGTTATGGTTCTTCCACCCCCCC--<br>GGTAAATGGTGCTATTTAGTGAATGCTTGTCGGACATATTTTTATCAATTTTCACTTCCTCTATTTTCTTCACAAAACTAGGAAATTCACCACAATTTTTTCTTTGTTATT<br>TTTTAATTTTTTTTTTATTTTTTAAAAACATTTTTTAAAAAACTAAATTACATACAAACTACCGCATAAAATCCCTCAAACTATACAAACGTTTATCGTATAATATATATAC<br>ATTATTGTTTATTCTATCATTATTAGAGAAACTCCACTACCAAAACCATCATTAAAACAAAAATTTACATGCCACTTAACTCCCCTCACAAACAATCGTTATTTATATTGT<br>TAATTAGCAAACACAAAACCCGC
KM433666	DBKM433666_China_Hap_B1a	ATTTTATTTTTTAACCTAACTCCCCTACTAAGTGTACCCCCCCTTTCCCCCCCAGGGGGGGTATACTATGCATAATCGTGCATACATTTATATACCACATATATTATGGT<br>ACCGGTAATATATACTATATATGTACTAAACCCATTATATGTATACGGGCATTAATCTATATTCCACATTTCTCCCAATGTCCATTCTATGCATGATCCAAGACATACTC<br>ATTCACCCTCCCCATAGACAGTTCTAAACCACTATCAAGCCACCTAACTATGAATGGTTACAGGACATAAATCTCACTCTCATGTTCTCCCCCTAACAAGTCACCTAAC<br>TATGAATGGTTACAGGACATACATTTAACTACC--<br>ATGTTCTAACCCATTTGGTTATGCTCGCCGTATCAGATGGATTTATTGATCGTCCACCTCACGAGAGATCAGCAACCCCTGCCTGTAATGTACTTCATGACCAGTCTCA<br>GGCCCATTCTTTCCCCCTACACCCCTCGCCCTACTTGCCTTCCACCGTACCTCTGGTTCCTCGGTCAGGCACATCCCATGCATAACTCCTGAACTTTCTCACTTTTCAC<br>GAAGTCATCTGTGGATTATCTTCCCCTCTTTAGTCCGTGATCGCGGCATCTTCTCTCTTCTATTGCTGTTGGTTCCTTCTCTTTTTGGGGCTTCTTCACAGGTTGCCCTT<br>CACAGTGCGGGTGCGGAGTGCTATTCAAGTGAAGCCTGGACTACACCTGCGTTGCGTCCTATCCTAGTCCTCTCGTGTCCCTCGATGAGACGGTTTGCGTATATGGGG<br>AATCATCTTGACACTGATGCACTTTGGATCGCATTTGGTTATGGTTCTTCCACCCCCCCC-<br>GGTAAATGGTGCTATTTAGTGAATGCTTGTCGGACATATTTTTATCAATTTTCACTTCCTCTATTTTCTTCACAAAACTAGGAAATTCACCACAATTTTTTCTTTGTTATT<br>TTTTAATTTTTTTTTTATTTTTTAAAAACATTTTTTAAAAAACTAAATTACATACAAACTACCGCATAAAATCCCTCAAACTATACAAACGTTTATCGTATAATATATATAC<br>ATTATTGTTTATTCTATCATTATTAGAGAAACTCCACTACCAAAACCATCATTAAAACAAAAATTTACATGCCACTTAACTCCCCTCACAAACAATCGTTATTTATATTGT<br>TAATTAGCAAACACAAAACCCAC

KM886936	DBKM886936_China_Hap_A	ATTTTATTTTTTAACCTAACTCCCCTACTAAGTGTACCCCCCCTTTCCCCCCCAGGGGGGGTATACTATGCATAATCGTGCATACATTTATATACCACATATATTATGGT<br>ACCGGTAATATATACTATATATGTACTAAACCCATTATATGTATACGGGCATTAACCTATATTCCACATTTCTCCCAATGTCCATTCTATGCATGATCCAGGACATACTC<br>ATTTACCCTCCCCATAGACAGTTCCAAACCACTATCAAGCCACCTAACTATGAATGGTTACAGGACATAAATCTCACTCTCATGTTCTCCCCCCAACAAGTCACCTAAC<br>TATGAATGGTTACAGGACATACATTTAACTACC--<br>ATGTTCTAACCCATTTGGTTATGCTCGCCGTATCAAATGGATTTATTGATCGTCCACCTCACGAGAGATCAGCAACCCCTGCCTGTAATGTACTTCATGACCAGTCTCA<br>GGCCCATTCTTTCCCCCTACACCCCTCGCCCTACTTGCCTTCCACCGTACCTCTGGTTCCTCGGTCAGGCACATCCCATGCATAACTCCTGAACTTTCTCACTTTTCAC<br>GAAGTCATCTGTGGATTATCTTCCCCTCTTTAGTCCGTGATCGCGGCATCTTCTCTCTTCTATTGCTGTTGGTTCCTTCTCTTTTTGGGGCTTCTTCACAGGTTACCCTT<br>CACAGTGCGGGTGCGGAGTGCTATTCAAGTGAAGCCTGGACTACACCTGCGTTGCGTCCTATCCTAGTCCTCTCGTGTCCCTCGATGAGACGGTTTGCGTGTATGGGG<br>AATCATCTTGACACTGATGCACTTTGGATCGCATTTGGTTATGGTTCTTCCACCCCCCC--<br>GGTAAATGGTGCTATTTAGTGAATGCTTGTCGGACATATTTTTATCAATTTTCACTTCCTCTATTTTCTTCACAAAACTAGGAAATTCACCACAATTTTTTCTTTGTTATT<br>TTTTAATTTTTTTTTTATTTTTTAAAAACATTTTTTAAAAAACTAAATTACATACAAACTACCGCATAAAATCCCTCAAACTATACAGACGTTTATCGTATAATATATATAC<br>ATTATTGTTTATTCTATCATTATTAAAGAAACTCCACTACCAAAACCATCATTAAAACAAAAATTTACATGCCACTTAACTCCCCTCACAAACAATCGTTATTTATATTGT<br>TAATTAGCAAACACAAAACCCGC
KM886937	DBKM886937_China_Hap_B	ATTTTATTTTTTAACCTAACTCCCCTACTAAGTGTACCCCCCCTTTCCCCCCCAGGGGGGGTATACTATGCATAATCGTGCATACATTTATATACCACATATATTATGGT<br>ACCGGTAATATATACTATATATGTACTAAACCCATTATATGTATACGGGCATTAATCTATATTCCACATTTCTCCCAATGTCCATTCTATGCATGATCCAAGACATACTC<br>ATTCACCCTCCCCATAGACAGTTCTAAACCACTATCAAGCCACCTAACTATGAATGGTTACAGGACATAAATCTCACTCTCATGTTCTCCCCCTAACAAGTCACCTAAC<br>TATGAATGGTTACAGGACATACATTTAACTACC--<br>ATGTTCTAACCCATTTGGTTATGCTCGCCGTATCAAATGGATTTATTGATCGTCCACCTCACGAGAGATCAGCAACCCCTGCCTGTAATGTACTTCATGACCAGTCTCA<br>GGCCCATTCTTTCCCCCTACACCCCTCGCCCTACTTGCCTTCCACCGTACCTCTGGTTCCTCGGTCAGGCACATCCCATGCATAACTCCTGAACTTTCTCACTTTTCAC<br>GAAGTCATCTGTGGATTATCTTCCCCTCTTTAGTCCGTGATCGCGGCATCTTCTCTCTTCTATTGCTGTTGGTTCCTTCTCTTTTTGGGGCTTCTTCACAGGTTGCCCTT<br>CACAGTGCGGGTGCGGAGTGCTATTCAAGTGAAGCCTGGACTACACCTGCGTTGCGTCCTATCCTAGTCCTCTCGTGTCCCTCGATGAGACGGTTTGCGTGTATGGGG<br>AATCATCTTGACACTGATGCACTTTGGATCGCATTTGGTTATGGTTCTTCCACCCCCCCC-<br>GGTAAATGGTGCTATTTAGTGAATGCTTGTCGGACATATTTTTATCAATTTTCACTTCCTCTATTTTCTTCACAAAACTAGGAAATTCACCACAATTTTTTCTTTGTTATT<br>TTTTAATTTTTTTTTTATTTTTTAAAAACATTTTTTAAAAAACTAAATTACATACAAACTACCGCATAAAATCCCTCAAACTATACAAACGTTTATCGTATAATATATATAC<br>ATTATTGTTTATTCTATCATTATTAGAGAAACTCCACTACCAAAACCATCATTAAAACAAAAATTTACATGCCACTTAACTCCCCTCACAAACAATCGTTATTTATATTGT<br>TAATTAGCAAACACAAAACCCAC
KM096864	DBKM096864_China_Hap_B1a	ATTTTATTTTTTAACCTAACTCCCCTACTAAGTGTACCCCCCCTTTCCCCCCCAGGGGGGGTATACTATGCATAATCGTGCATACATTTATATACCACATATATTATGGT<br>ACCGGTAATATATACTATATATGTACTAAACCCATTATATGTATACGGGCATTAATCTATATTCCACATTTCTCCCAATGTCCATTCTATGCATGATCCAAGACATACTC<br>ATTCACCCTCCCCATAGACAGTTCTAAACCACTATCAAGCCACCTAACTATGAATGGTTACAGGACATAAATCTCACTCTCATGCTCTCCCCCTAACAAGTCACCTAAC<br>TATGAATGGTTACAGGACATACATTTAACTACC--<br>ATGTTCTAACCCATTTGGTTATGCTCGCCGTATCAGATGGATTTATTGATCGTCCACCTCACGAGAGATCAGCAACCCCTGCCTGTAATGTACTTCATGACCAGTCTCA<br>GGCCCATTCTTTCCCCCTACACCCCTCGCCCTACTTGCCTTCCACCGTACCTCTGGTTCCTCGGTCAGGCACATCCCATGCATAACTCCTGAACTTTCTCACTTTTCAC<br>GAAGTCATCTGTGGATTATCTTCCCCTCTTTAGTCCGTGATCGCGGCATCTTCTCTCTTCTATTGCTGTTGGTTCCTTCTCTTTTTGGGGCTTCTTCACAGGTTGCCCTT<br>CACAGTGCGGGTGCGGAGTGCTATTCAAGTGAAGCCTGGACTACACCTGCGTTGCGTCCTATCCTAGTCCTCTCGTGTCCCTCGATGAGACGGTTTGCGTATATGGGG<br>AATCATCTTGACACTGATGCACTTTGGATCGCATTTGGTTATGGTTCTTCCACCCCCCC--<br>GGTAAATGGTGCTATTTAGTGAATGCTTGTCGGACATATTTTTATCAATTTTCACTTCCTCTATTTTCTTCACAAAACTAGGAAATTCACCACAATTTTTTCTTTGTTATT<br>TTTTAATTTTTTTTTTATTTTTTAAAAACATTTTTTAAAAAACTAAATTACATACAAACTACCGCATAAAATCCCTCAAACTATACAAACGTTTATCGTATAATATATATAC<br>ATTATTGTTTATTCTATCATTATTAGAGAAACTCCACTACCAAAACCATCATTAAAACAAAAATTTACATGCCACTTAACTCCCCTCACAAACAATCGTTATTTATATTGT<br>TAATTAGCAAACACAAAACCCAC

KP211418	DBKP211418_India_Hap_E	ATTTTATTTTTTAACCTAACTCCCCTACTAAGTGTACCCCCCCTTTCCCC---<br>AGGGGGGGTATACTATGCATAATCGTGCATACATTTATATACCACATATATTATGGTACCGGTAATATATACTATATATGTACTAAACCCATTATATGTATACGGGCATT<br>AATCTATATTCCACATTTCTCCCAATGTCCATTCTATGCATGATCCAGGACATACTCATTTACCCTCCCCATAGACAGTTCCAAACCACTACCAAGCCACCTAACTATG<br>AATGGTTACAGGACATAAATCTCACTCTCATGTTCTCCCCCCAACAAGTCACCTAACTATGAATGGTTACAGGACATACATTTAACTACC--------------------------<br>---------------------------------------<br>ATGTTCTAACCCATTTGGTTATGCTCGCCGTATCAGATGGATTTATTGATCGTCCACCTCACGAGAGATCAGCAACCCCTGCTTGTAATGTACTTCATGACCAGTCTCA<br>GGCCCATTCTTTCCCCCTACACCCCTCGCCCTACTTGCCTTCCACCGTACCTCTGGTTCCTCGGTCAGGCACATCCCATGCATAACTCCTGAACTTTCTCACTTTTCAC<br>GAAGTCATCTGTGGATTATCTTCCCCTCTTTAGTCCGTGATCGCGGCATCTTCTCTCTTCTATTGCTGTTGGTTCCTTCTCTTTTTGGGGCTTCTTCACAGGTTGCCCTT<br>CACAGTGCGGGTGCG-<br>AGTGCTATTCAAGTGAAGCCTGGACTACACCTGCGTTGCGTCCTATCCTAGTCCTCTCGTGTCCCTCGATGAGACGGTTTGCGTGTATGGGGAATCATCTTGACACTGA<br>TGCACTTTGGATCGCATTTGGTTATGGTTCTTCCACCCCCCC--<br>GGTAAATGGTGCTATTTAGTGAATGCTTGTCGGACATATTTTTATCAATTTTCACTTCCTCTATTTTCTTCACAAAACTAGGAAATTCACCACAATTTTTTCTTTGTTATT<br>TTTTAATTTTTTTTTTATTTTTTAAAAACATTTTTTAAAAAACTAAATTACATACAAACTACCGCATAAAATCCCTCAAACTATACAAACGTTTATCGTATAATATATATAC<br>ATTATTGTTTATTCTATCATTATTAGAGAAACTCCACTACCAAAACCATCATTAAAACAAAAATTTACATGCCACTTAACTCCCCTCACAAACAATCGTTATTTATATTGT<br>TAATTAGCAAACACAAAACCTGC
KP211419	DBKP211419_India_Hap_E3b	ATTTTATTTTTTAACCTAACTCCCCTACTAAGTGTACCCCCCCTTTCCCC---<br>AGGGGGGGTATACTATGCATAATCGTGCATACATTTATATACCACATATATTATGGTACCGGTAATATATACTATATATGTACTAAACCCATTATATGTATACGGGCATT<br>AATCTATATTCCACATTTCTCCCAATGTCCATTCTATGCATGATCCAGGACATACTCATTTACCCTCCCCATAGACAGCTCTAAGCCACTACCAAGTCACCTAACTATG<br>AATGGTTACAGGACATAAATCTCACTCTCATGTTCTCCCCCCAACAAGTCACCTAACTATGAATGGTTACAGGACATACATTTAACTACC--------------------------<br>---------------------------------------<br>ATGTTCTAACCCATTTGGTTATGCTCGCCGTATCAGATGGATTTATTGATCGTCCACCTCACGAGAGATCAGCAACCCCTGCTTGTAATGTACTTCATGACCAGTCTCA<br>GGCCCATTCTTTCCCCCTACACCCCTCGCCCTACTTGCCTTCCACCGTACCTCTGGTTCCTCGGTCAGGCACATCCCATGCATAACTCCTGAACTTTCTCACTTTTCAC<br>GAAGTCATCTGTGGATTATCTTCCCCTCTTTAGTCCGTGATCGCGGCATCTTCTCTCTTCTATTGCTGTTGGTTCCTTCTCTTTTTGGGGCTTCTTCACAGGTTGCCCTT<br>CACAGTGCGGGTGCG-<br>AGTGCTATTCAAGTGAAGCCTGGACTACACCTGCGTTGCGTCCTATCCTAGTCCTCTCGTGTCCCTCGATGAGACGGTTTGCGTGTATGGGGAATCATCTTGACACTGA<br>TGCACTTTGGATCGCATTTGGTTATGGTTCTTCCACCCCCCC--<br>GGTAAATGGTGCTATTTAGTGAATGCTTGTCGGACATATTTTTATCAATTTTCACTTCCTCTATTTTCTTCACAAAACTAGGAAATTCACCACAATTTTTTCTTTGTTATT<br>TTTTAATTTTTTTTTTATTTTTTAAAAACATTTTTTAAAAAACTAAATTACATACAAACTACCGCATAAAATCCCTCAAACTATACAAACGTTTATCGTATAATATATATAC<br>ATTATTGTTTATTCTATCATTATTAGAGAAACTCCACTACCAAAACCATCATTAAAACAAAAATTTACATGCCACTTAACTCCCCTCACAAACAATCGTTATTTATATTGT<br>TAATTAGCAAACACAAAACCTGC
KP211420	DBKP211420_India_Hap_E2	ATTTTATTTTTTAACCTAACTCCCCTACTAAGTGTACCCCCCCTTTCCCC---<br>AGGGGGGGTATACTATGCATAATCGTGCATACATTTATATACCACATATATTATGGTACCGGTAATATATACTATATATGTACTAAACCCATTATATGTATACGGGCATT<br>AATCTATATTCCACATTTCTCCCAATGTCCATTCTATGCATGATCCAGGACACACTCATCCACCCTCCCCATAGACAGCTCCAAACCACTACCAAGTCACCTAACTATG<br>AATGGTTACAGGACATAAATCTCACTCTCATGTTCTCCCCCCAACAAGTCACCTAACTATGAATGGTTACAGGACATACATTTAACTACC--------------------------<br>---------------------------------------<br>ATGTTCTAACCCATTTGGTTATGCTCGCCGTATCAGATGGATTTATTGATCGTCCACCTCACGAGAGATCAGCAACCCCTGCTTGTAATGTACTTCATGACCAGTCTCA<br>GGCCCATTCTTTCCCCCTACACCCCTCGCCCTACTTGCCTTCCACCGTACCTCTGGTTCCTCGGTCAGGCACATCCCATGCATAACTCCTGAACTTTCTCACTTTTCAC<br>GAAGTCATCTGTGGATTATCTTCCCCTCTTTAGTCCGTGATCGCGGCATCTTCTCTCTTCTATTGCTGTTGGTTCCTTCTCTTTTTGGGGCTTCTTCACAGGTTGCCCTT<br>CACAGTGCGGGTGCG-<br>AGTGCTATTCAAGTGAAGCCTGGACTACACCTGCGTTGCGTCCTATCCTAGTCCTCTCGTGTCCCTCGATGAGACGGTTTGCGTGTATGGGGAATCATCTTGACACTGA<br>TGCACTTTGGATCGCATTTGGTTATGGTTCTTCCACCCCCCC--<br>GGTAAATGGTGCTATTTAGTGAATGCTTGTCGGACATATTTTTATCAATTTTCACTTCCTCTATTTTCTTCACAAAACTAGGAAATTCACCACAATTTTTTCTTTGTTATT<br>TTTTAATTTTTTTTTTATTTTTTAAAAACATTTTTTAAAAAACTAAATTACATACAAACTACCGCATAAAATCCCTCAAACTATACAAACGTTTATCGTATAATATATATAC<br>ATTATTGTTTATTCTATCATTATTAGAGAAACTCCACTACCAAAACCATCATTAAAACAAAAATTTACATGCCACTTAACTCCCCTCACAAGCAATCGTTATTTATATTGT<br>TAATTAGCAAACACAAAACCTGC

KP211421	DBKP211421_India_Hap_E1a	ATTTTATTTTTTAACCTAACTCCCCTACTAAGTGTACCCCCCCTTTCCCC---<br>AGGGGGGGTATACTATGCATAATCGTGCATACATTTATATACCACATATATTATGGTACCGGTAATATATACTATATATGTACTAAACCCATTATATGTATACGGGCATT<br>AATCTATATTCCACATTTCTCCCAATGTCCATTCTATGCATGATCCAGGACATACTCATTTACCCTCCCCATAGACAGCTCCAAACCACTATCAAGTCACCTAACTATG<br>AATGGTTACAGGACATAAATCTCACTCTCATGTTCTCCCCCCAACAAGTCACCTAACTATGAATGGTTACAGGACATACATTTAACTACC------------------------<br>------------------------------------<br>ATGTTCTAACCCATTTGGTTATGCTCGCCGTATCAGATGGATTTATTGATCGTCCACCTCACGAGAGATCAGCAACCCCTGCTTGTAATGTACTTCATGACCAGTCTCA<br>GGCCCATTCTTTCCCCCTACACCCCTCGCCCTACTTGCCTTCCACCGTACCTCTGGTTCCTCGGTCAGGCACATCCCATGCATAACTCCTGAACTTTCTCACTTTTCAC<br>GAAGTCATCTGTGGATTATCTTCCCCTCTTTAGTCCGTGATCGCGGCATCTTCTCTCTTCTATTGCTGTTGGTTCCTTCTCTTTTTGGGGCTTCTTCACAGGTTGCCCTT<br>CACAGTGCGGGTGCG-<br>AGTGCTATTCAAGTGAAGCCTGGACTACACCTGCGTTGCGTCCTATCCTAGTCCTCTCGTGTCCCTCGATGAGACGGTTTGCGTGTATGGGGAATCATCTTGACACTGA<br>TGCACTTTGGATCGCATTTGGTTATGGTTCTTCCACCCCCCC--<br>GGTAAATGGTGCTATTTAGTGAATGCTTGTCGGACATATTTTTATCAATTTTCACTTCCTCTATTTTCTTCACAAAACTAGGAAATTCACCACAATTTTTTCTTTGTTATT<br>TTTTAATTTTTTTTTTATTTTTTAAAAACATTTTTTAAAAAACTAAATTACATACAAACTACCGCATAAAATCCCTCAAACTATACAAACGTTTATCGTATAATATATATAC<br>ATTATTGTTTATTCTATCATTATTAGAGAAACTCCACTACCAAAACCATCATTAAAACAAAAATTTACATGCCACTTAACTCCCCTCACAAACAATCGTTATTTATATTGT<br>TAATTAGCAAACACAAAACCTGC
KP211422	DBKP211422_India_Hap_I	ATTTTATTTTTTAACCTAACTCCCCTACTAAGTGTACCCCCCCTTTCCCC---<br>AGGGGGGGTATACTATGCATAATCGTGCATACATTTATATACCACATATATTATGGTACCGGTAATATATACTATATATGTACTAAACCCATTATATGTATACGGGCATT<br>AATCTATATTCCACATTTCTCCCAATGTCCATTCCATGCATGATCCAAGACATACTCATTTACCCTCCTCATAGACAACTCTAAACCACTACCAAGCCACCTAACTATGA<br>ATGGTTACCGGACATAAACCTCACTCTCATGTTCTCCCCCCAACAAGTCACCTAACTATGAATGGTTACAGGACATATATTTAACTACT-------------------------<br>-----------------------------------<br>ATGTTCTAACCCATTTGGTTATGCTCGCCGTATCAGATGGATTTATTGATCGTCCACCTCACGAGAGATCAGCAACCCCTGCTCGTAATGTACTTCATGACCAGTCTCA<br>GGCCCATTCTTTCCCCCTACACCCCTCGCCCCACTTGCCTTCCACCGTGCCTCTGGTTCCTCGGTCAGGCACATCCCATGCATAACTCCTGAACTTTCTCACTTTTCAC<br>GAAGTCATCTGTGGATTATCTTCCCCTCTTTAGTCCGTGATCGCGGCATCTTCTCTCTTCTATTGCTGTTGGTTCCTTCTCTTTTTGGGGCTTCTTCACAGGTTGCCCTT<br>CACAGTGCGGGTGCG-<br>AGTGCTATTCAAGTGAAGCCTGGACTACACCTGCGTTGCGTCCTATCCTAGTCCTCTCGTGTCCCTCGATGAGACGGTTTGCGTGTATGGGGAATCATCTTGACACTGA<br>TGCACTTTGGATCGCATTTGGTTATGGTTCTTCCACCCCCCC--<br>GGTAAATGGTGCTATTTAGTGAATGCTTGTCGGACATATTTTTATCAATTTTCACTTCCTCTATTTTCTTCACAAAACTAGGAAATTCACCACAATTTTTTCTTTGTTATT<br>TTTTAATTTTTTTTTTATTTTTTAAAAACATTTTTTAAAAAACTAAATTACATACAAACTACCGCATAAAATCCCTCAAACTATACAAACGTTTATCGTATAATATATATAC<br>ATTATTGTTTATTCTATCATTATTAGAGAAACTCCACTACCAAAACCATCATTAAAACAAAAATTTACATGCCACTTAACTCCCCTCACAAACAATCGTTATTTATATTGT<br>TAATTAGCAAACACAAAACCCGC
KP211423	DBKP211423_India_Hap_E1a	ATTTTATTTTTTAACCTAACTCCCCTACTAAGTGTACCCCCCCTTTCCCC---<br>AGGGGGGGTATACTATGCATAATCGTGCATACATTTATATACCACATATATTATGGTACCGGTAATATATACTATATATGTACTAAACCCATTATATGTATACGGGCATT<br>AATCTATATTCCACATTTCTCCCAATGTCCATTCTATGCATGATCCAGGACACACTCATTCACCCTCCCCATAGACAGCTCCAAACCACTACCAAGTCACCTAACTATG<br>AATGGTTACAGGACATAAATCTCACTCTCATGTTCTCCCCCCAACAAGTCACCTAACTATGAATGGTTACAGGACATACATTTAACTACC------------------------<br>------------------------------------<br>ATGTTCTAACCCATTTGGTTATGCTCGCCGTATCAGATGGATTTATTGATCGTCCACCTCACGAGAGATCAGCAACCCCTGCTTGTAATGTACTTCATGACCAGTCTCA<br>GGCCCATTCTTTCCCCCTACACCCCTCGCCCTACTTGCCTTCCACCGTACCTCTGGTTCCTCGGTCAGGCACATCCCATGCATAACTCCTGAACTTTCTCACTTTTCAC<br>GAAGTCATCTGTGGATTATCTTCCCCTCTTTAGTCCGTGATCGCGGCATCTTCTCTCTTCTATTGCTGTTGGTTCCTTCTCTTTTTGGGGCTTCTTCACAGGTTGCCCTT<br>CACAGTGCGGGTGCG-<br>AGTGCTATTCAAGTGAAGCCTGGACTACACCTGCGTTGCGTCCTATCCTAGTCCTCTCGTGTCCCTCGATGAGACGGTTTGCGTGTATGGGGAATCATCTTGACACTGA<br>TGCACTTTGGATCGCATTTGGTTATGGTTCTTCCACCCCCCC--<br>GGTAAATGGTGCTATTTAGTGAATGCTTGTCGGACATATTTTTATCAATTTTCACTTCCTCTATTTTCTTCACAAAACTAGGAAATTCACCACAATTTTTTCTTTGTTATT<br>TTTTAATTTTTTTTTTATTTTTTAAAAACATTTTTTAAAAAACTAAATTACATACAAACTACCGCATAAAATCCCTCAAACTATACAAACGTTTATCGTATAATATATATAC<br>ATTATTGTTTATTCTATCATTATTAGAGAAACTCCACTACCAAAACCATCATTAAAACAAAAATTTACATGCCACTTAACTCCCCTCACAAACAATCGTTATTTATATTGT<br>TAATTAGCAAACACAAAACCTGC

KP211424	DBKP211424_India_Hap_G1a	ATTTTATTTTTTAACCTAACTCCCCTACTAAGTGTACCCCCCCTTTCCCC---AGGGGGGGTATACTATGCATAATCGTGCATACATTTATATACCACATATATTATGGTACCGGTAATATATACTATATATGTACTAAACCCATTATATGTATACGGGCATTAATCTATATTCCACATTTCTCCCAATGTCCATTCTATGCATGATCCAAGACATACTCATTAACCCTCCCCATAGACAGCTCTAAACCACTACCAAGTCACCTAACTATGAATGGTTACAGGACATAAATCTAACTCTTATGTTCTCCCCCCAACAAGCCACCTAACTATGAATGGTTACAGGACATACATTTAACTACC--ATGTTCTAACCCATTTGGTTATGCTCGCCGTATCAGATGGATTTATTGATCGTCCACCTCACGAGAGATCAGCAACCCCTGCTTGTAATGTACTTCATGACCAGTCTCAGGCCCATTCTTTCCCCCTACACCCCTCGCCCTACTTGCCTTCCACCGTACCTCTGGTTCCTCGGTCAGGCACATCCCATGCATAACTCCTGAACTTTCTCACTTTTCACGAAGTCATCTGTGGATTATCTTCCCCTCTTTAGTCCGTGATCGCGGCATCTTCTCTCTTCTATTGCTGTTGGTTCCTTCTCTTTTTGGGGCTTCTTCACAGGTTACCCTTCACAGTGCGGGTGCG-AGTGCTATTCAAGTGAAGCCTGGACTACACCTGCGTTGCGTCCTATCCTAGTCCTCTCGTGTCCCTCGATGAGACGGTTTGCGTGTATGGGGAATCATCTTGACACTGATGCACTTTGGATCGCATTTGGTTATGGTTCTTCCACCCCCCC--GGTAAATGGTGCTATTTAGTGAATGCTTGTCGGACATATTTTTATCAATTTTCACTTCCTCTATTTTCTTCACAAAACTAGGAAATTCACCACAATTTTTTCTTTGTTATTTTTTAATTTTTTTTTTATTTTTTAAAAACATTTTTTAAAAAACTAAATTACATACAAACTACCGCATAAAATCCCTCAAACTATACAAACGTTTATCGTATAATATATATACATTATTGTTTATTCTATCATTATTAGAGAAACTCCACTACCAAAACCATCATTAAAACAAAAATTTACATGCCACTTAACTCCCCTCACAAACAATCGTTATTTATATTGTTAATTAGCAAACACAAAACCTGC
KP211425	DBKP211425_India_Hap_E1	ATTTTATTTTTTAACCTAACTCCCCTACTAAGTGTACCCCCCCTTTCCCC---AGGGGGGGTATACTATGCATAATCGTGCATACATTTATATACCACATATATTATGGTACCGGTAATATATACTATATATGTACTAAACCCATTATATGTATACGGGCATTAATCTATATTCCACATTTCTCCCAATGTCCATTCTATGCATGATCCAGGACATACTCATTTACCCTCCCCATAAACAGCTCCAAACCACTACCAAGTCACCTAACTATGAATGGTTACAGGACATAAATCTCACTCTCATGTTCTCCCCCCAACAAGTCACCTAACTATGAATGGTTACAGGACATACATTTAACTACC--ATGTTCTAACCCATTTGGTTATGCTCGCCGTATCAGATGGATTTATTGATCGTCCACCTCACGAGAGATCAGCAACCCCTGCTTGTAATGTACTTCATGACCAGTCTCAGGCCCATTCTTTCCCCCTACACCCCTCGCCCTACTTGCCTTCCACCGTACCTCTGGTTCCTCGGTCAGGCACATCCCATGCATAACTCCTGAACTTTCTCACTTTTCACGAAGTCATCTGTGGATTATCTTCCCCTCTTTAGTCCGTGATCGCGGCATCTTCTCTCTTCTATTGCTGTTGGTTCCTTCTCTTTTTGGGGCTTCTTCACAGGTTGCCCTTCACAGTGCGGGTGCG-AGTGCTATTCAAGTGAAGCCTGGACTACACCTGCGTTGCGTCCTATCCTAGTCCTCTCGTGTCCCTCGATGAGACGGTTTGCGTGTATGGGGAATCATCTTGACACTGATGCACTTTGGATCGCATTTGGTTATGGTTCTTCCACCCCCCC--GGTAAATGGTGCTATTTAGTGAATGCTTGTCGGACATATTTTTATCAATTTTCACTTCCTCTATTTTCTTCACAAAACTAGGAAATTCACCACAATTTTTTCTTTGTTATTTTTTAATTTTTTTTTTATTTTTTAAAAACATTTTTTAAAAAACTAAATTACATACAAACTACCGCATAAAATCCCTCAAACTATACAAACGTTTATCGTATAATATATATACATTATTGTTTATTCTATCATTATTAGAGAAACTCCACTACCAAAACCATCATTAAAACAAAAATTTACATGCCACTTAACTCCCCTCACAAACAATCGTTATTTATATTGTTAATTAGCAAACACAAAACCTGC
KP244335	DBKP244335_China_Hap_E1	ATTTTATTTTTTAACCTAACTCCCCTACTAAGTGTACCCCCCCTTTCCCCCCCAGGGGGGGTATACTATGCATAATCGTGCATACATTTATATACCACATATATTATGGTACCGGTAATATATACTATATATGTACTAAACCCATTATATGTATACGGGCATTAATCTATATTCCACATTTCTCCCAATGTCCATTCTATGCATGATCCAGGACACACTCATTCACCCTCCCCATAGACAGCTCCAAACCACTACCAAGTCACCTAACTATGAATGGTTACAGGACATAAATCTCACTCTCATGTTCTTCCCCCAACAAGTCACCTAACTATGAATGGTTACAGGACATACATTTAACTACC--ATGTTCTAACCCATTTGGTTATGCTCGCCGTATCAGATGGATTTATTGATCGTCCACCTCACGAGAGATCAGCAACCCCTGCTTGTAATGTACTTCATGACCAGTCTCAGGCCCATTCTTTCCCCCTACACCCCTCGCCCTACTTGCCTTCCACCGTACCTCTGGTTCCTCGGTCAGGCACATCCCATGCATAACTCCTGAACTTTCTCACTTTTCACGAAGTCATCTGTGGATTATCTTCCCCTCTTTAGTCCGTGATCGCGGCATCTTCTCTCTTCTATTGCTGTTGGTTCCTTCTCTTTTTGGGGCTTCTTCACAGGTTGCCCTTCACAGTGCGGGTGCGGAGTGCTATTCAAGTGAAGCCTGGACTACACCTGCGTTGCGTCCTATCCTAGTCCTCTCGTGTCCCTCGATGAGACGGTTTGCGTGTATGGGGAATCATCTTGACACTGATGCACTTTGGATCGCATTTGGTTATGGTTCTTCCACCCCCCCC-GGTAAATGGTGCTATTTAGTGAATGCTTGTCGGACATATTTTTATCAATTTTCACTTCCTCTATTTTCTTCACAAAACTAGGAAATTCACCACAATTTTTTCTTTGTTATTTTTTAATTTTTTTTTTATTTTTTAAAAACATTTTTTAAAAAACTAAATTACATACAAACTACCGCATAAAATCCCTCAAACTATACAAACGTTTATCGTATAATATATATACATTATTGTTTATTCTATCATTATTAGAGAAACTCCACTACCAAAACCATCATTAAAACAAAAATTTACATGCCACTTAACTCCCCTCACAAACAATCGTTATTTATATTGTTAATTAGCAAACACAAAACCTGC

KP269069	DBKP269069_China_Hap_C1b	ATTTTATTTTTTAACCTAACTCCCCTACTAAGTGTACCCCCCCTTTCCCCCCCAGGGGGGGTATACTATGCATAATCGTGCATACATTTATATACCACATATATTATGGT<br>ACCGGTAATATATACTATATATGTACTAAACCCATTATATGTATACGGGCATTAATCTATATTCCACATTTCTCCCAATGTCCATTCTATGCATGATCCAGGACATACTC<br>ATTCACCCTCCCCATAGACAACTCCAAACCACTACCAAGCCACCTAACTATGAATGGTTGCAGGACATAAATCTCACTCTCATGTTCTTCCCCCAACAAGTCACCTAAC<br>TATGAATGGTTGCAGGACATACATTTAACTACT--<br>ATGCTCTAACCCATTTGGTTATGCTCGCCGTATCAGATGGATTTATTGATCGTCCACCTCACGAGAGATCAGCAACCCCTGCCTGTAATGTACTTCATGACCAGTCTCA<br>GGCCCATTCTTTCCCCCTACACCCCTCGCCCTACTTGCCTTCCACCGTACCTCTGGTTCCTCGGTCAGGCACATCCCATGCATAACTCCTGAACTTTCTCACTTTTCAC<br>GAAGTCATCTGTGGATTATCTTCCCCTCTTTAGTCCGTGATCGCGGCATCTTCTCTCTTCTATTGCTCTTGGTTCCTTCTCTTTTTGGGGCTTCTTCACAGGTTGCCCTT<br>CACAGTGCGGGTGCGGAGTGCTATTCAAGTGAAGCCTGGACTACACCTGCGTTGCGTCCTATCCTAGTCCTCTCGTGTCCCTCGATGAGACGGTTTGCGTGTATGGGG<br>AATCATCTTGACACTGATGCACTTTGGATCGCATTTGGTTATGGTTCTTCCACCCCCCC-<br>GGTAAATGGTGCTATTTAGTGAATGCTTGTCGGACATATTTTTATCAATTTTCACTTCCTCTATTTTCTTCACAAAACTAGGAAATTCACCACAATTTTTTCTTTGTTATT<br>TTTTAATTTTTTTTTTATTTTTTAAAAACATTTTTTAAAAAACTAAATTACATACAAACTACCGCATAAAATCCCTCAAACTATACAAACGTTTATCGTATAATATATATAC<br>ATTATTGTTTATTCTATCATTATTAGAGAAACTCCACTACCAAAACCATCATTAAAACAAAAATTTACATGCCACTTAACTCCCCTCACAAACAATCGTTATTTATATTGT<br>TAATTAGCAAACACAAAACCCGC
KP681580	DBKP681580_China_Hap_C1b	ATTTTATTTTTTAACCTAACTCCCCTACTAAGTGTACCCCCCCTTTCCCCCCCAGGGGGGGTATACTATGCATAATCGTGCATACATTTATATACCACATATATTATGGT<br>ACCGGTAATATATACTATATATGTACTAAACCCATTATATGTATACGGGCATTAATCTATATTCCACATTTCTCCCAATGTCCATTCTATGCATGATCCAGGACATACTC<br>ATTCACCCTCCCCATAGACAACTCCAAACCACTACCAAGCCACCTAACTATGAATGGTTGCAGGACATAAATCTCACTCTCATGTTCTTCCCCCAACAAGTCACCTAAC<br>TATGAATGGTTGCAGGACATACATTTAACTACT--<br>ATGCTCTAACCCATTTGGTTATGCTCGCCGTATCAGATGGATTTATTGATCGTCCACCTCACGAGAGATCAGCAACCCCTGCCTGTAATGTACTTCATGACCAGTCTCA<br>GGCCCATTCTTTCCCCCTACACCCCTCGCCCTACTTGCCTTCCACCGTACCTCTGGTTCCTCGGTCAGGCACATCCCATGCATAACTCCTGAACTTTCTCACTTTTCAC<br>GAAGTCATCTGTGGATTATCTTCCCCTCTTTAGTCCGTGATCGCGGCATCTTCTCTCTTCTATTGCTGTTGGTTCCTTCTCTTTTTGGGGCTTCTTCACAGGTTGCCCTT<br>CACAGTGCGGGTGCGGAGTGCTATTCAAGTGAAGCCTGGACTACACCTGCGTTGCGTCCTATCCTAGTCCTCTCGTGTCCCTCGATGAGACGGTTTGCGTGTATGGGG<br>AATCATCTTGACACTGATGCACTTTGGATCGCATTTGGTTATGGTTCTTCCACCCCCC--<br>GGTAAATGGTGCTATTTAGTGAATGCTTGTCGGACATATTTTTATCAATTTTCACTTCCTCTATTTTCTTCACAAAACTAGGAAATTCACCACAATTTTTTCTTTGTTATT<br>TTTTAATTTTTTTTTTATTTTTTAAAAACATTTTTTAAAAAACTAAATTACATACAAACTACCGCATAAAATCCCTCAAACTATACAAACGTTTATCGTATAATATATATAC<br>ATTATTGTTTATTCTATCATTATTAGAGAAACTCCACTACCAAAACCATCATTAAAACAAAAATTTACATGCCACTTAACTCCCCTCACAAACAATCGTTATTTATATTGT<br>TAATTAGCAAACACAAAACCCGC
KP681581	DBKP681581_China_Hap_B1a	ATTTTATTTTTTAACCTAACTCCCCTACTAAGTGTACCCCCCCTTTCCCCCCCAGGGGGGGTATACTATGCATAATCGTGCATACATTTATATACCACATATATTATGGT<br>ACCGGTAATATATACTATATATGTACTAAACCCATTATATGTATACGGGCATTAATCTATATTCCACATTTCTCCCAATGTCCATTCTATGCATGATCCAAGACATACTC<br>ATTCACCCTCCCCATAGACAGTTCTAAACCACTATCAAGCCACCTAACTATGAATGGTTACAGGACATAAATCTCACTCTCATGTTCTCCCCCTAACAAGTCACCTAAC<br>TATGAATGGTTACAGGACATACATTTAACTACC--<br>ATGTTCTAACCCATTTGGTTATGCTCGCCGTATCAGATGGATTTATTGATCGTCCACCTCACGAGAGATCAGCAACCCCTGCCTGTAATGTACTTCATGACCAGTCTCA<br>GGCCCATTCTTTCCCCCTACACCCCTCGCCCTACTTGCCTTCCACCGTACCTCTGGTTCCTCGGTCAGGCACATCCCATGCATAACTCCTGAACTTTCTCACTTTTCAC<br>GAAGTCATCTGTGGATTATCTTCCCCTCTTTAGTCCGTGATCGCGGCATCTTCTCTCTTCTATTGCTGTTGGTTCCTTCTCTTTTTGGGGCTTCTTCACAGGTTGCCCTT<br>CACAGTGCGGGTGCGGAGTGCTATTCAAGTGAAGCCTGGACTACACCTGCGTTGCGTCCTATCCTAGTCCTCTCGTGTCCCTCGATGAGACGGTTTGCGTATATGGGG<br>AATCATCTTGACACTGATGCACTTTGGATCGCATTTGGTTATGGTTCTTCCACCCCCCC-<br>GGTAAATGGTGCTATTTAGTGAATGCTTGTCGGACATATTTTTATCAATTTTCACTTCCTCTATTTTCTTCACAAAACTAGGAAATTCACCACAATTTTTTCTTTGTTATT<br>TTTTAATTTTTTTTTTATTTTTTAAAAACATTTTTTAAAAAACTAAATTACATACAAACTACCGCATAAAATCCCTCAAACTATACAAACGTTTATCGTATAATATATATAC<br>ATTATTGTTTATTCTATCATTATTAGAGAAACTCCACTACCAAAACCATCATTAAAACAAAAATTTACATGCCACTTAACTCCCCTCACAAACAATCGTTATTTATATTGT<br>TAATTAGCAAACACAAAACCCAC

KP742951	DBKP742951_China_Hap_B	ATTTTATTTTTTAACCTAACTCCCCTACTAAGTGTACCCCCCCTTTCCCCCCCAGGGGGGGTATACTATGCATAATCGTGCATACATTTATATACCACATATATTATGGT<br>ACCGGTAATATATACTATATATGTACTAAACCCATTATATGTATACGGGCATTAATCTATATTCCACATTTCTCCCAATGTCCATTCTATGCATGATCCAAGACATATTC<br>ATTCACCCTCCCCATAGACAGTTCTAAACCACTATCAAGTCACCTAACTATGAATGGTTACAGGACATAAATCTCACTCTCATGTTCTCCCCCTAACAAGTCACCTAAC<br>TATGAATGGTTACAGGACATACATTTAACTACC--<br>ATGTTCTAACCCATTTGGTTATGCTCGCCGTATCAGATGGATTTATTGATCGTCCACCTCACGAGAGATCAGCAACCCCTGCCTGTAATGTACTTCATGACCAGTCTCA<br>GGCCCATTCTTTCCCCCTACACCCCTCGCCCTACTTGCCTTCCACCGTACCTCTGGTTCCTCGGTCAGGCACATCCCATGCATAACTCCTGAACTTTCTCACTTTTCAC<br>GAAGTCATCTGTGGATTATCTTCCCCTCTTTAGTCCGTGATCGCGGCATCTTCTCTCTTCTATTGCTGTTGGTTCCTTCTCTTTTTGGGGCTTCTTCACAGGTTGCCCTT<br>CACAGTGCGGGTGCGGAGTGCTATTCAAGTGAAGCCTGGACTACACCTGCGTTGCGTCCTATCCTAGTCCTCTCGTGTCCCTCGATGAGACGGTTTGCGTGTATGGGG<br>AATCATCTTGACACTGATGCACTTTGGATCGCATTTGGTTATGGTTCTTCCACCCCCCCCCGGTAAATGGTGCTATTTAGTGAATGCTTGTCGGACATATTTTTATCAAT<br>TTTCACTTCCTCTATTTTCTTCACAAAACTAGGAAATTCACCACAATTTTTTCTTTGTTATTTTTTAATTTTTTTTTTATTTTTTAAAAACATTTTTTAAAAAACTAAATTAC<br>ATACAAACTACCGCATAAAATCCCTCAAACTATACAAACGTTTATCGTATAATATATATACATTATTGTTTATTCTATCATTATTAGAGAAACTCCACTACCAAAACCAT<br>CATTAAAACAAAAATTTACATGCCACTTAACTCCCCTCACAAACAATCGTTATTTATATTGTTAATTAGCAAACACAAAACCCAC
KR347464	DBKR347464_China_Hap_E1	ATTTTATTTTTTAACCTAACTCCCCTACTAAGTGTACCCCCCCTTTCCCCCCCAGGGGGGGTATACTATGCATAATCGTGCATACATTTATATACCACATATATTATGGT<br>ACCGGTAATATATACTATATATGTACTAAACCCATTATATGTATACGGGCATTAATCTATATTCCACATTTCTCCCAATGTCCATTCTATGCATGATCCAGGACACACTC<br>ATTCACCCTCCCCATAGACAGCTCCAAACCACTACCAAGTCACCTAACTATGAATGGTTACAGGACATAAATCTCACTCTCATGTTCTTCCCCCAACAAGTCACCTAAC<br>TATGAATGGTTACAGGACATACATTTAACTACC--<br>ATGTTCTAACCCATTTGGTTATGCTCGCCGTATCAGATGGATTTATTGATCGTCCACCTCACGAGAGATCAGCAACCCCTGCTTGTAATGTACTTCATGACCAGTCTCA<br>GGCCCATTCTTTCCCCCTACACCCCTCGCCCTACTTGCCTTCCACCGTACCTCTGGTTCCTCGGTCAGGCACATCCCATGCATAACTCCTGAACTTTCTCACTTTTCAC<br>GAAGTCATCTGTGGATTATCTTCCCCTCTTTAGTCCGTGATCGCGGCATCTTCTCTCTTCTATTGCTGTTGGTTCCTTCTCTTTTTGGGGCTTCTTCACAGGTTGCCCTT<br>CACAGTGCGGGTGCGGAGTGCTATTCAAGTGAAGCCTGGACTACACCTGCGTTGCGTCCTATCCTAGTCCTCTCGTGTCCCTCGATGAGACGGTTTGCGTGTATGGGG<br>AATCATCTTGACACTGATGCACTTTGGATCGCATTTGGTTATGGTTCTTCCACCCCCCCC-<br>GGTAAATGGTGCTATTTAGTGAATGCTTGTCGGACATATTTTTATCAATTTTCACTTCCTCTATTTTCTTCACAAAACTAGGAAATTCACCACAATTTTTTCTTTGTTATT<br>TTTTAATTTTTTTTTTATTTTTTAAAAACATTTTTTAAAAAACTAAATTACATACAAACTACCGCATAAAATCCCTCAAACTATACAAACGTTTATCGTATAATATATATAC<br>ATTATTGTTTATTCTATCATTATTAGAGAAACTCCACTACCAAAACCATCATTAAAACAAAAATTTACATGCCACTTAACTCCCCTCACAAACAATCGTTATTTATATTGT<br>TAATTAGCAAACACAAAACCTGC
KT283576	DBKT283576_China_Hap_C3	ATTTTATTTTTTAACCTAACTCCCCTACTAAGTGTACCCCCCCTTTCCCCCCCAGGGGGGGTATACTATGCATAATCGTGCATACATTTATATACCACATATATTATGGT<br>ACCGGTAATATATACTATATATGTACTAAACCCATTATATGTATACGGGCATTAATCTATATTCCTAATTTCTCCCAATGTCCATTCTATGCATGATCCAGGACATCCTC<br>ATTCACCCTCCCCATAGACAGCTCCAAACCACTACCAAGTCACTCAACTATGAATGGTTGCAGGACATAAATCTCACTCTCATGCTCTTCCCCCAACAAGTCACCTAAC<br>TATGAATGGTTGCAGGACATACATTTAACTACT--<br>ATGTTCTAACCCATTTGGTTATGCTCGCCGTATCAGATGGATTTATTGATCGTCCACCTCACGAGAGATCAGCAACCCCTGCCTGTAATGTACTTCATGACCAGTCTCA<br>GGCCCATTCTTTCCCCCTACACCCCTCGCCCTACTTGCCTTCCACCGTACCTCTGGTTCCTCGGTCAGGCACATCCCATGCATAACTCCTGAACTTTCTCACTTTTCAC<br>GAAGTCATCTGTGGATTATCTTCCCCTCTTTAGTCCGTGATCGCGGCATCTTCTCTCTTCTATTGCTGTTGGTTCCTTCTCTTTTTGGGGCTTCTTCACAGGTTAGCCTT<br>CACAGTGCGGGTGCGGAGTGCTATTCAAGTGAAGCCTGGACTACACCTGCGTTGCGTCCTATCCTAGTCCTCTCGTGTCCCTCGATGAGACGGTTTGCGTGTATGGGG<br>AATCATCTTGACACTGATGCACTTTGGATCGCATTTGGTTATGGTTCTTCCACCCCCCC--<br>GGTAAATGGTGCTATTTAGTGAATGCTTGTCGGACATATTTTTATCAATTTTCACTTCCTCTATTTTCTTCACAAAACTAGGAAATTCACCACAATTTTTTCTTTGTTATT<br>TTTTAATTTTTTTTTTATTTTTTAAAAACATTTTTTAAAAAACTAAATTACATACAAACTACCGCATAAAATCCCTCAAACTATACAAACGTTTATCGTATAATATATATAC<br>ATTATTGTTTATTCTATCATTATTAGAGAAACTCCACTACCAAAACCATCATTAAAACAAAAATTTACATGCCACTTAACTCCCCTCACAAACAATCGTTATTTATATTGT<br>TAATTAGCAAACACAAAACCCGC

KX512321	DBKX512321_China_Hap_C1	ATTTTATTTTTTAACCTAACTCCCCTACTAAGTGTACCCCCCCTTTCCCCCCCAGGGGGGGTATACTATGCATAATCGTGCATACATTTATATACCACATATATTATGGT<br>ACCGGTAATATATACTATATATGTACTAAACCCATTATATGTATACGGGCATTAATCTATATTCCACATTTCTCCCAATGTCCATTCTATGCATGATCCAGGACATACTC<br>ATTCACCCTCCCCATAGACAACTCCAAACCACTACCAAGCCACCTAACTATGAATGGTTGCAGGACATAAATCTCACTCTCATGTTCTTCCCCCAACAAGTCACCTAAC<br>TATGAATGGTTACAGGACATACATTTAACTACT---<br>ATGCTCTAACCCATTTGGTTATGCTCGCCGTATCAGATGGATTTATTGATCGTCCACCTCACGAGAGATCAGCAACCCCTGCCTGTAATGTACTTCATGACCAGTCTCA<br>GGCCCATTCTTTCCCCCTACACCCCTCGCCCTACTTGCCTTCCACCGTACCTCTGGTTCCTCGGTCAGGCACATCCCATGCATAACTCCTGAACTTTCTCACTTTTCAC<br>GAAGTCATCTGTGGATTATCTTCCCCTCTTTAGTCCGTGATCGCGGCATCTTCTCTCTTCTATTGCTGTTGGTTCCTTCTCTTTTTGGGGCTTCTTCACAGGTTGCCCTT<br>CACAGTGCGGGTGCGGAGTGCTATTCAAGTGAAGCCTGGACTACACCTGCGTTGCGTCCTATCCTAGTCCTCTCGCGTCCATCGATGAGACGGTTTGCGTGTATGGGG<br>AATCATCTTGACACTGATGCACTTTGGATCGGATTTGGTTATGGTTCTTCCACCCCCCC--<br>GGTAAATGGTGCTATTTAGTGAATGCTTGTCGGACATATTTTTATCAATTTTCACTTCCTCTATTTTCTTCACAAAACTAGGAAATTCACCACAATTTTTTCTTTGTTATT<br>TTTTAATTTTTTTTTTATTTTTTAAAAACATTTTTTAAAAAACTAAATTACATACAAACTACCGCATAAAATCCCTCAAACTATACAGACGTTTATGCTATAATATATATAC<br>ATTATTGTTTATTCTATCATTATTAAAGAAACTCCACTACCAAAACCGCCTCTAAAACAAAAATTTACATGCCACTTAACTCCCCTCACAAACAATCGTTATTTATATTGT<br>TAATTAGCAAACACAAAACCCGC
KX781318	DBKX781318_China_Hap_E1a	ATTTTATTTTTTAACCTAACTCCCCTACTAAGTGTACCCCCCCTTTCCCCCCCAGGGGGGGTATACTATGCATAATCGTGCATACATTTATATACCACATATATTATGGT<br>ACCGGTAATATATACTATATATGTACTAAACCCATTATATGTATACGGGCATTAATCTATATTCCACATTTCTCCCAATGTCCATTCTATGCATGATCCAGGACACACTC<br>ATTCACCCTCCCCATAGACAGCTCCAAACCACTACCAAGTCACCTAACTATGAATGGTTACAGGACATAAATCTCACTCTCATGTTCTTCCCCCAACAAGTCACCTAAC<br>TATGAATGGTTACAGGACATACATTTAACTACC---<br>ATGTTCTAACCCATTTGGTTATGCTCGCCGTATCAGATGGATTTATTGATCGTCCACCTCACGAGAGATCAGCAACCCCTGCTTGTAATGTACTTCATGACCAGTCTCA<br>GGCCCATTCTTTCCCCCTACACCCCTCGCCCTACTTGCCTTCCACCGTACCTCTGGTTCCTCGGTCAGGCACATCCCATGCATAACTCCTGAACTTTCTCACTTTTCAC<br>GAAGTCATCTGTGGATTATCTTCCCCTCTTTAGTCCGTGATCGCGGCATCTTCTCTCTTCTATTGCTGTTGGTTCCTTCTCTTTTTGGGGCTTCTTCACAGGTTGCCCTT<br>CACAGTGCGGGTGCGGAGTGCTATTCAAGTGAAGCCTGGACTACACCTGCGTTGCGTCCTATCCTAGTCCTCTCGTGTCCCTCGATGAGACGGTTTGCGTGTATGGGG<br>AATCATCTTGACACTGATGCACTTTGGATCGCATTTGGTTATGGTTCTTCCACCCCCCC--<br>GGTAAATGGTGCTATTTAGTGAATGCTTGTCGGACATATTTTTATCAATTTTCACTTCCTCTATTTTCTTCACAAAACTAGGAAATTCACCACAATTTTTTCTTTGTTATT<br>TTTTAATTTTTTTTTTATTTTTTAAAAACATTTTTTAAAAAACTAAATTACATACAAACTACCGCATAAAATCCCTCAAACTATACAAACGTTTATCGTATAATATATATAC<br>ATTATTGTTTATTCTATCATTATTAGAGAAACTCCACTACCAAAACCATCATTAAAACAAAAATTTACATGCCACTTAACTCCCCTCACAAACAATCGTTATTTATATTGT<br>TAATTAGCAAACACAAAACCTGC
KX781319	DBKX781319_China_Hap_A	ATTTTATTTTTTAACCTAACTCCCCTACTAAGTGTACCCCCCCTTTCCCCCCCAGGGGGGGTATACTATGCATAATCGTGCATACATTTATATACCACATATATTATGGT<br>ACCGGTAATATATACTATATATGTACTAAACCCATTATATGTATACGGGCATTAACCTATATTCCACATTTCTCCCAATGTCCATTCTATGCATGATCCAGGACATACTC<br>ATTTACCCTCCCCATAGGCAGTTCCAAACCACTATCAAGCCACCTAACTATGAATGGTTACAGGACATAAATCTCACTCTCATGTTCTCCCCCCAACAAGTCACCTAAC<br>TATGAATGGTTACAGGACATACATTTAACTACC---<br>ATGTTCTAACCCATTTGGTTATGCTCGCCGTATCAGATGGATTTATTGATCGTCCACCTCACGAGAGATCAGCAACCCCTGCCTGTAATGTACTTCATGACCAGTCTCA<br>GGCCCATTCTTTCCCCCTACACCCCTCGCCCTACTTGCCTTCCACCGTACCTCTGGTTCCTCGGTCAGGCACATCCCATGCATAACTCCTGAACTTTCTCACTTTTCAC<br>GAAGTCATCTGTGGATTATCTTCCCCTCTTTAGTCCGTGATCGCGGCATCTTCTCTCTTCTATTGCTGTTGGTTCCTTCTCTTTTTGGGGCTTCTTCACAGGTTGCCCTT<br>CACAGTGCGGGTGCGGAGTGCTATTCAAGTGAAGCCTGGACTACACCTGCGTTGCGTCCTATCCTAGTCCTCTCGTGTCCCTCGATGAGACGGTTTGCGTGTATGGGG<br>AATCATCTTGACACTGATGCACTTTGGATCGCATTTGGTTATGGTTCTTCCACCCCCCC--<br>GGTAAATGGTGCTATTTAGTGAATGCTTGTCGGACATATTTTTATCAATTTTCACTTCCTCTATTTTCTTCACAAAACTAGGAAATTCACCACAATTTTTTCTTTGTTATT<br>TTTTAATTTTTTTTTTATTTTTTAAAAACATTTTTTAAAAAACTAAATTACATACAAACTACCGCATAAAATCCCTCAAACTATACAAACGTTTATCGTATAATATATATAC<br>ATTATTGTTTATTCTATCATTATTAGAGAAACTCCACTACCAAAACCATCATTAAAACAAAAATTTACATGCCACTTAACTCCCCTCACAAACAATCGTTATTTATATTGT<br>TAATTAGCAAACACAAAACCCGC

KX987152	DBKX987152_China_Hap_B	ATTTTATTTTTTAACCTAACTCCCCTACTAAGTGTACCCCCCCTTTCCCCCCCAGGGGGGGTATACTATGCATAATCGTGCATACATTTATATACCACATATATTATGGT<br>ACCGGTAATATATACTATATATGTACTAAACCCATTATATGTATACGGGCATTAATCTATATTCCACATTTCTCCCAATGTCCATTCTATGCATGATCCAAGACATACTC<br>ATTCACCCTCCCCATAGACAGTTCTAAACCACTATCAAGCCACCTAACTATGAATGGTTACAGGACATAAATCTCACTCTCATGTTCTCCCCCTAACAAGTCACCTAAC<br>TATGAATGGTTACAGGACATACATTTAACTACC---<br>ATGTTCTAACCCATTTGGTTATGCTCGCCGTATCAGATGGATTTATTGATCGTCCACCTCACGAGAGATCAGCAACCCCTGCCTGTAATGTACTTCATGACCAGTCTCA<br>GGCCCATTCTTTCCCCCTACACCCCTCGCCCTACTTGCCTTCCACCGTACCTCTGGTTCCTCGGTCAGGCACATCCCATGCATAACTCCTGAACTTTCTCACTTTTCAC<br>GAAGTCATCTGTGGATTATCTTCCCCTCTTTAGTCCGTGATCGCGGCATCTTCTCTCTTCTATTGCTGTTGGTTCCTTCTCTTTTTGGGGCTTCTTCACAGGTTGCCCTT<br>CACAGTGCGGGTGCGGAGTGCTATTCAAGTGAAGCCTGGACTACACCTGCGTTGCGTCCTATCCTAGTCCTCTCGTGTCCCTCGATGAGACGGTTTGCGTGTATGGGG<br>AATCATCTTGACACTGATGCACTTTGGATCGCATTTGGTTATGGTTCTTCCACCCCCCCC-<br>GGTAAATGGTGCTATTTAGTGAATGCTTGTCGGACATATTTTTATCAATTTTCACTTCCTCTATTTTCTTCACAAAACTAGGAAATTCACCACAATTTTTTCTTTGTTATT<br>TTTTAATTTTTTTTTTATTTTTTAAAAACATTTTTTAAAAAACTAAATTACATACAAACTACCGCATAAAATCCCTCAAACTATACAAACGTTTATCGTATAATATATATAC<br>ATTATTGTTTATTCTATCATTATTAGAGAAACTCCACTACCAAAACCATCATTAAAACAAAAATTTACATGCCACTTAACTCCCCTCACAAACAATCGTTATTTATATTGT<br>TAATTAGCAAACACAAAACCCAC
KY039381	DBKY039381_Marquesas_Hap_D1a	ATTTTATTTTTTAACCTAACTCCCCTACTAAGTGTACCCCCCCTTTCCCCCCCAGGGGGGGTATACTATGCATAATCGTGCATACATTTATATACCACATATATTATGGT<br>ACCGGTAATATATACTATATATGTACTAAACCCATTATATGTATACGGGCATTAATCTATATTCCACATTTCTCCCAATGTCCATTCTATGCATGATCCAGGACATACTC<br>ATTCACCCTCCCCATAGACAGCTCCAAACCACTACCAAGTCACCTAACTATGAATGGTTGCAGGACATAAATCTTACTCTCATGCTCTTCCCCCAACAAGTCACCTAAC<br>TATGAATGGTTGCAGGACATACATTTAACTACC---<br>ATGTTCTAACCCATTTGGTTATGCTCGCCGTATCAGATGGATTTATTGATCGTCCACCTCACGAGAGATCAGCAACCCCTGCCTGTAATGTACTTCATGACCAGTCTCA<br>GGCCCATTCTTTCCCCCTACACCCCTCGCCCTACTTGCCTTCCACCGTACCTCTGGTTCCTCGGTCAGGCACATCCCATGCATAACTCCTGAACTTTCTCACTTTTCAC<br>GAAGTCATCTGTGGATTATCTTCCCCTCTTTAGTCCGTGATCGCGGCATCTTCTCTCTTCTATTGCTGTTGGTTCCTTCTCTTTTTGGGGCTTCTTCACAGGTTACCCTT<br>CACAGTGCGGGTGCGGAGTGCTATTCAAGTGAAGCCTGGACTACACCTGCGTTGCGTCCTATCCTAGTCCTCTCGTGTCCCTCGATGAGACGGTTTGCGTGTATGGGG<br>AATCATCTTGACACTGATGCACTTTGGATCGCATTTGGTTATGGTTCTTCCACCCNCCCC-<br>GGTAAATGGTGCTATTTAGTGAATGCTTGTCGGACATATTTTTATCAATTTTCACTTCCTCTATTTTCTTCACAAAACTAGGAAATTCACCACAATTTTTTCTTTGTTATT<br>TTTTAATTTTTTTTTTATTTTTTAAAAACATTTTTTAAAAAACTAAATTACATACAAACTACCGCATAAAATCCCTCAAACTATACAAACGTTTATCGTATAATATATATAC<br>ATTATTGTTTATTCTATCATTATTAGAGAAACTCCACTACCAAAACCATCATTAAAACAAAAATTTACATGCCACTTAACTCCCCTCACAAACAATCGTTATTTATATTGT<br>TAATTAGCAAACACAAAACCCGC
KY039382	DBKY039382_Niue_Hap_D1	ATTTTATTTTTTAACCTAACTCCCCTACTAAGTGTACCCCCCCTTTCCCCCCCAGGGGGGGTATACTATGCATAATCGTGCATACATTTATATACCACATATATTATGGT<br>ACCGGTAATATATACTATATATGTACTAAACCCATTATATGTATACGGGCATTAATCTATATTCCACATTTCTCCCAATGTCCATTCTATGCATGATCCAGGACATACTC<br>ATTCACCCTCCCCATAGACAGCTCCAAACCACTACCAAGTCACCTAACTATGAATGGTTGCAGGACATAAATCTTACTCTCATGCTCTTCCTCCAACAAGTCACCTAAC<br>TATGAATGGTTGCAGGACATACATTTAACTACC---<br>ATGTTCTAACCCATTTGGTTATGCTCGCCGTATCAGATGGATTTATTGATCGTCCACCTCACGAGAGATCAGCAACCCCTGCCTGTAATGTACTTCATGACCAGTCTCA<br>GGCCCATTCTTTCCCCCTACACCCCTCGCCCTACTTGCCTTCCACCGTACCTCTGGTTCCTCGGTCAGGCACATCCCATGCATAACTCCTGAACTTTCTCACTTTTCAC<br>GAAGTCATCTGTGGATTATCTTCCCCTCTTTAGTCCGTGATCGCGGCATCTTCTCTCTTCTATTGCTGTTGGTTCCTTCTCTTTTTGGGGCTTCTTCACAGGTTACCCTT<br>CACAGTGCGGGTGCGGAGTGCTATTCAAGTGAAGCCTGGACTACACCTGCGTTGCGTCCTATCCTAGTCCTCTCGTGTCCCTCGATGAGACGGTTTGCGTGTATGGGG<br>AATCATCTTGACACTGATGCACTTTGGATCGCATTTGGTTATGGTTCTTCCACCCCCCCC-<br>GGTAAATGGTGCTATTTAGTGAATGCTTGTCGGACATATTTTTATCAATTTTCACTTCCTCTATTTTCTTCACAAAACTAGGAAATTCACCACAATTTTTTCTTTGTTATT<br>TTTTAATTTTTTTTTTATTTTTTAAAAACATTTTTTAAAAAACTAAATTACATACAAACTACCGCATAAAATCCCTCAAACTATACAAACGTTTATCGTATAATATATATAC<br>ATTATTGTTTATTCTATCATTATTAGAGAAACTCCACTACCAAAACCATCATTAAAACAAAAATTTACATGCCACTTAACTCCCCTCACAAACAATCGTTATTTATATTGT<br>TAATTAGCAAACACAAAACCCGC

KY039383	DBKY039383_Hawaii_Hap_D1a	ATTTTATTTTTTAACCTAACTCCCCTACTAAGTGTACCCCCCCTTTCCCCCCCAGGGGGGGTATACTATGCATAATCGTGCATACATTTATATACCACATATATTATGGT<br>ACCGGTAATATATACTATATATGTACTAAACCCATTATATGTATACGGGCATTAATCTATATTCCACATTTCTCCCAATGTCCATTCTATGCATGATCCAGGACATACTC<br>ATTCACCCTCCCCATAGACAGCTCCAAACCACTACCAAGTCACCTAACTATGAATGGTTGCAGGACATAAATCTTACTCTCATGCTCTTCCCCCAACAAGTCACCTAAC<br>TATGAATGGTTGCAGGACATACATTTAACTACC--<br>ATGTTCTAACCCATTTGGTTATGCTCGCCGTATCAGATGGATTTATTGATCGTCCACCTCACGAGAGATCAGCAACCCCTGCCTGTAATGTACTTCATGACCAGTCTCA<br>GGCCCATTCTTTCCCCCTACACCCCTCGCCCTACTTGCCTTCCACCGTACCTCTGGTTCCTCGGTCAGGCACATCCCATGCATAACTCCTGAACTTTCTCACTTTTCAC<br>GAAGTCATCTGTGGATTATCTTCCCCTCTTTAGTCCGTGATCGCGGCATCTTCTCTCTTCTATTGCTGTTGGTTCCTTCTCTTTTTGGGGCTTCTTCACAGGTTACCCTT<br>CACAGTGCGGGTGCGGAGTGCTATTCAAGTGAAGCCTGGACTACACCTGCGTTGCGTCCTATCCTAGTCCTCTCGTGTCCCTCGATGAGACGGTTTGCGTGTATGGGG<br>AATCATCTTGACACTGATGCACTTTGGATCGCATTTGGTTATGGTTCTTCCACCCCCCCC-<br>GGTAAATGGTGCTATTTAGTGAATGCTTGTCGGACATATTTTTATCAATTTTCACTTCCTCTATTTTCTTCACAAAACTAGGAAATTCACCACAATTTTTCTTTGTTATT<br>TTTTAATTTTTTTTTTATTTTTTAAAAACATTTTTTAAAAAACTAAATTACATACAAACTACCGCATAAAATCCCTCAAACTATACAAACGTTTATCGTATAATATATATAC<br>ATTATTGTTTATTCTATCATTATTAGAGAAACTCCACTACCAAAACCATCATTAAAACAAAAATTTACATGCCACTTAACTCCCCTCACAAACAATCGTTATTTATATTGT<br>TAATTAGCAAACACAAAACCCGC
KY039384	DBKY039384_Hawaii_Hap_D1a	ATTTTATTTTTTAACCTAACTCCCCTACTAAGTGTACCCCCCCTTTCCCCCCCAGGGGGGGTATACTATGCATAATCGTGTATACATTTATATACCATATATATTATGGT<br>ACCGGTAATATATACTATATATGTACTAAACCCATTATATGTATACGGGCATTAATCTATATTCCACATTTCTCCCAATGTCCATTCTATGCATGATCCAGGACATACTC<br>ATTCACCCTCCCCATAGACAGCTCCAAACCACTACCAAGTCACCTAACTATGAATGGTTGCAGGACATAAATCTTACTCTCATGCTCTTCCCCCAACAAGTCACCTAAC<br>TATGAATGGTTGCAGGACATACATTTAACTACC--<br>ATGTTCTAACCCATTTGGTTATGCTCGCCGTATCAGATGGATTTATTGATCGTCCACCTCACGAGAGATCAGCAACCCCTGCCTGTAATGTACTTCATGACCAGTCTCA<br>GGCCCATTCTTTCCCCCTACACCCCTCGCCCTACTTGCCTTCCACCGTACCTCTGGTTCCTCGGTCAGGCACATCCCATGCATAACTCCTGAACTTTCTCACTTTTCAC<br>GAAGTCATCTGTGGATTATCTTCCCCTCTTTAGTCCGTGATCGCGGCATCTTCTCTCTTCTATTGCTGTTGGTTCCTTCTCTTTTTGGGGCTTCTTCACAGGTTACCCTT<br>CACAGTGCGGGTGCGGAGTGCTATTCAAGTGAAGCCTGGACTACACCTGCGTTGCGTCCTATCCTAGTCCTCTCGTGTCCCTCGATGAGACGGTTTGCGTGTATGGGG<br>AATCATCTTGACACTGATGCACTTTGGATCGCATTTGGTTATGGTTCTTCCANCCCCCC-<br>GGTAAATGGTGCTATTTAGTGAATGCTTGTCGGACATATTTTTATCAATTTTCACTTCCTCTATTTTCTTCACAAAACTAGGAAATTCACCACAATTTTTCTTTGTTATT<br>TTTTAATTTTTTTTTTATTTTTTAAAAACATTTTTTAAAAAACTAAATTACATACAAACTACCGCATAAAATCCCTCAAACTATACAAACGTTTATCGTATAATATATATAC<br>ATTATTGTTTATTCTATCATTATTAGAGAAACTCCACTACCAAAACCATCATTAAAACAAAAATTTACATGCCACTTAACTCCCCTCACAAACAATCGTTATTTATATTGT<br>TAATTAGCAAACACAAAACCCGC
KY039385	DBKY039385_Hawaii_Hap_D1a	ATTTTATTTTTTAACCTAACTCCCCTACTAAGTGTACCCCCCCTTTCCCCCCCAGGGGGGGTATACTATGCATAATCGTGCATACATTTATATACCACATATATTATGGT<br>ACCGGTAATATATACTATATATGTACTAAACCCATTATATGTATACGGGCATTAATCTATATTCCACATTTCTCCCAATGTCCATTCTATGCATGATCCAGGACATACTC<br>ATTCACCCTCCCCATAGACAGCTCCAAACCACTACCAAGTCACCTAACTATGAATGGTTGCAGGACATAAATCTTACTCTCATGCTCTTCCCCCAACAAGTCACCTAAC<br>TATGAATGGTTGCAGGACATACATTTAACTACC--<br>ATGTTCTAACCCATTTGGTTATGCTCGCCGTATCAGATGGATTTATTGATCGTCCACCTCACGAGAGATCAGCAACCCCTGCCTGTAATGTACTTCATGACCAGTCTCA<br>GGCCCATTCTTTCCCCCTACACCCCTCGCCCTACTTGCCTTCCACCGTACCTCTGGTTCCTCGGTCAGGCACATCCCATGCATAACTCCTGAACTTTCTCACTTTTCAC<br>GAAGTCATCTGTGGATTATCTTCCCCTCTTTAGTCCGTGATCGCGGCATCTTCTCTCTTCTATTGCTGTTGGTTCCTTCTCTTTTTGGGGCTTCTTCACAGGTTACCCTT<br>CACAGTGCGGGTGCGGAGTGCTATTCAAGTGAAGCCTGGACTACACCTGCGTTGCGTCCTATCCTAGTCCTCTCGTGTCCCTCGATGAGACGGTTTGCGTGTATGGGG<br>AATCATCTTGACACTGATGCACTTTGGATCGCATTTGGTTATGGTTCTTCCACCCCCCCC-<br>GGTAAATGGTGCTATTTAGTGAATGCTTGTCGGACATATTTTTATCAATTTTCACTTCCTCTATTTTCTTCACAAAACTAGGAAATTCACCACAATTTTTCTTTGTTATT<br>TTTTAATTTTTTTTTTATTTTTTAAAAACATTTTTTAAAAAACTAAATTACATACAAACTACCGCATAAAATCCCTCAAACTATACAAACGTTTATCGTATAATATATATAC<br>ATTATTGTTTATTCTATCATTATTAGAGAAACTCCACTACCAAAACCATCATTAAAACAAAAATTTACATGCCACTTAACTCCCCTCACAAACAATCGTTATTTATATTGT<br>TAATTAGCAAACACAAAACCCGC

KY039386	DBKY039386_Easter Island_Hap_D1a	ATTTTATTTTTTAACNTAACTCCCCTACTAAGTGTACCCCCCCTTTCCCCCCCAGGGGGGGTATACTATGCATAATCGTACATACATTTATATACCACATATATTATGGT<br>ACCGGTAATATATACTATATATGTANTAAANCCATTATATGTATACGGGCATTAATCTATATTCCACATTTCTCCCAATGTCCATTCTATGCATGATCCAGGACATACTC<br>ATTCACCCTCCCCATAGACAGCTCCAAACCACTACCAAGTCACCTAACTATGAATGGTTGCAGGACATAAATCTTACTCTCATGCTCTTCCCCCAACAAGTCACCTAAC<br>TATGAATGGTTGCAGGACATACATTTAACTACC---<br>ATGTTCTAACCCATTTGGTTATGCTCGCCGTATCAGATGGATTTATTGATCGTCCACCTCACGAGAGATCAGCAACCCCTGCCTGTAATGTACTTCATGACCAGTCTCA<br>GGCCCATTCTTTCCCCCTACACCCCTCGCCCTACTTGCCTTCCACCGTACCTCTGGTTCCTCGGTCAGGCACATCCCATGCATAACTCCTGAACTTTCTCACTTTTCAC<br>GAAGTCATCTGTGGATTATCTTCCCCTCTTTAGTCCGTGATCGCGGCATCTTCTCTCTTCTATTGCTGTTGGTTCCTTCTCTTTTTGGGGCTTCTTCACAGGTTACCCTT<br>CACAGTGCGGGTGCGGAGTGCTATTCAAGTGAAGCCTGGACTACACCTGCGTTGCGTCCTATCCTAGTCCTCTCGTGTCCCTCGATGAGACGGTTTGCGTGTATGGGG<br>AATCATCTTGACACTGATGCACTTTGGATCGCATTTGGTTATGGTTCTTCCACCCNCCCC-<br>GGTAAATGGTGCTATTTAGTGAATGCTTGTCGGACATATTTTTATCAATTTTCACTTCCTCTATTTTCTTCACAAAACTAGGAAATTCACCACAATTTTTTCTTTGTTATT<br>TTTTAATTTTTTTTTTATTTTTTAAAAACATTTTTTAAAAAACTAAATTACATACAAACTACCGCATAAAATCCCTCAAACTATACAAACGTTTATCGTATAATATATATAC<br>ATTATTGTTTATTCTATCATTATTAGAGAAACTCCACTACCAAAACCATCATTAAAACAAAAATTTACATGCCACTTAACTCCCCTCACAAACAATCGTTATTTATATTGT<br>TAATTAGCAAACACAAAACCCGC
KY039387	DBKY039387_Easter Island_Hap_D1a	ATTTTATTTTTTAATCTAACTCCCCTACTAAGTGTACCCCCCCTTTCCCNNCNANGGGNGGTATACTATGCATAATCGTGCATACATTTATATACCACATATATTATGGT<br>ANCGGTAATATATACTATATATGTACTAAACCCATTATATGTATACGGGCATTAATCTATATTCCACATTTCTCCCAATGTCCATTCTATGCATGATCCAGGACATACTC<br>ATTCACCCTCCCCATAGACAGCTCCAAACCACTACCAAGTCACCTAACTATGAATGGTTGCAGGACATAAATCTTACTCTCATGCTCTTCCCCCAACAAGTCACCTAAC<br>TATGAATGGTTGCAGGACATACATTTAACTACC---<br>ATGTTCTAACCCATTTGGTTATGCTCGCCGTATCAGATGGATTTATTGATCGTCCACCTCACGAGAGATCAGCAACCCCTGCCTGTAATGTACTTCATGACCAGTCTCA<br>GGCCCATTCTTTCCCCCTACACCCCTCGCCCTACTTGCCTTCCACCGTACCTCTGGTTCCTCGGTCAGGCACATCCCATGCATAACTCCTGAACTTTCTCACTTTTCAC<br>GAAGTCATCTGTGGATTATCTTCCCCTCTTTAGTCCGTGATCGCGGCATCTTCTCTCTTCTATTGCTGTTGNTTCCTTCTCTTTTTGGGGCTTCTTCACAGGTTACCCTT<br>CACAGTGCGGGTGCGGAGTGCTATTCAAGTGAAGCCTGGACTACACCTGCGTTGCGTCCTATCCTAGTCCTCTCGTGTCCCTCGATGAGACGGTTTGCGTGTATGGGG<br>AATCATCTTGACACTGATGCACTTTGGATCGCATTTGGTTATGGTTCTTCCANCCCCCC-<br>GGTAAATGGTGCTATTTAGTGAATGCTTNTCNGACATATTTTTATCAATTTTCACTTCCTCTATTTTCTTCACAAAACTAGGAAATTCACCACAATTTTTTCTTTGTTATT<br>TTTTAATTTTTTTTTTATTTTTTAAAAATATTTTTTAAAAAACTAAATTACATACAAACTACCGCATAAAATCCCTCAAACTATACAAACNTTTATCGTATAATATATATAC<br>ATTATTGTTTATTNTATCATTATTAGAGAAACTNCACTACCAAAACCATCATTAAAACAAAAATTTACATGCCACTTAACTCCCCTCACAAACAATCGTTATTTATATTGT<br>TAATTAGCAAACACAAAACCCGC
KY039388	DBKY039388_Easter Island_Hap_D1a	ATTTTATTTTTTAACCTAACTCCCCTATTAAGTGTACCCCCCCTTTCCCCCCCAGGGGGGGTATACTATGCATAATCGTGCATACATTTATATACCACATATATTATGGT<br>ACCGGTAATATATACTATATATGTACTAAACCCATTATATGTATACGGGCATTAATCTATATTCCACATTTCTCCCAATGTCCATTCTATGCATGATCCAGGACATACTC<br>ATTCACCCTCCCCATAGACAGCTCCAAACCACTACCAAGTCACCTAACTATGAATGGTTGCAGGACATAAATCTTACTCTCATGCTCTTCCCCCAACAAGTCACCTAAC<br>TATGAATGGTTGCAGGACATACATTTAACTACC---<br>ATGTTCTAACCCATTTGGTTATGCTCGCCGTATCAGATGGATTTATTGATCGTCCACCTCACGAGAGATCAGCAACCCCTGCCTGTAATGTACTTCATGACCAGTCTCA<br>GGCCCATTCTTTCCCCCTACACCCCTCGCCCTACTTGCCTTCCACCGTACCTCTGGTTCCTCGGTCAGGCACATCCCATGCATAACTCCTGAACTTTCTCACTTTTCAC<br>GAAGTCATCTGTGGATTATCTTCCCCTCTTTAGTCCGTGATCGCGGCATCTTCTCTCTTCTATTGCTGTTGGTTCCTTCTCTTTTTGGGGCTTCTTCACAGGTTACCCTT<br>CACAGTGCGGGTGCGGAGTGCTATTCAAGTGAAGCCTGGACTACACCTGCGTTGCGTCCTATCCTAGTCCTCTCGTGTCCCTCGATGAGACGGTTTGCGTGTATGGGG<br>AATCATCTTGACACTGATGCACTTTGGATCGCATTTGGTTATGGTTCTTCCANCCCCCC-<br>GGTAAATGGTGCTATTTAGTGAATGCTTGTCGGACATATTTTTATCAATTTTCACTTCCTCTATTTTCTTCACAAAACTAGGAAATTCACCACAATTTTTTCTTTGTTATT<br>TTTTAATTTTTTTTTTATTTTTTAAAAACATTTTTTAAAAAACTAAATTACATACAAANTACCGCATAAAATCCCTCAAACTATACAAACGTTTATCGTATAATATATATAC<br>ATTATTGTTTATTCTATCATTATTAGAGAAACTCCACTACCAAAACCATCATTAAAACAAAAATTTACATGCCACTTAACTCCCCTCACAAACAATCGTTATTTATATTGT<br>TAATTAGCAAACACAAAACCCGC

KY039389	DBKY039389_Easter Island_Hap_D1a	ATTTTATTTTTTAACCTAACTCCCCTACTAAGTGTACCCCCCCTTTCCCCCCCAGGGGGGGTATACTATGCATAATCGTGCATACATTTATATACCACATATATTATGGT<br>ACCGGTAATATATACTATATATGTACTAAACCCATTATATGTATACGGGCATTAATCTATATTCCACATTTCTCCCAATGTCCATTCTATGCATGATCCAGGACATACTC<br>ATTCACCCTCCCCATAGACAGCTCCAAACCACTACCAAGTCACCTAACTATGAATGGTTGCAGGACATAAATCTTACTCTCATGCTCTTCCCCCAACAAGTCACCTAAC<br>TATGAATGGTTGCAGGACATACATTTAACTACC--<br>ATGTTCTAACCCATTTGGTTATGCTCGCCGTATCAGATGGATTTATTGATCGTCCACCTCACGAGAGATCAGCAACCCCTGCCTGTAATGTACTTCATGACCAGTCTCA<br>GGCCCATTCTTTCCCCCTACACCCCTCGCCCTACTTGCCTTCCACCGTACCTCTGGTTCCTCGGTCAGGCACATCCCATGCATAACTCCTGAACTTTCTCACTTTTCAC<br>GAAGTCATCTGTGGATTATCTTCCCCTCTTTAGTCCGTGATCGCGGCATCTTCTCTCTTCTATTGCTGTTGGTTCCTTCTCTTTTTGGGGCTTCTTCACAGGTTACCCTT<br>CACAGTGCGGGTGCGGAGTGCTATTCAAGTGAAGCCTGGACTACACCTGCGTTGCGTCCTATCCTAGTCCTCTCGTGTCCCTCGATGAGACGGTTTGCGTGTATGGGG<br>AATCATCTTGACACTGATGCACTTTGGATCGCATTTGGTTATGGTTCTTCCACCCNCCCC-<br>GGTAAATGGTGCTATTTAGTGAATGCTTGTCGGACATATTTTTATCAATTTTCACTTCCTCTATTTTCTTCACAAAACTAGGAAATTCACCACAATTTTTTCTTTGTTATT<br>TTTTAATTTTTTTTTTATTTTTTAAAAACATTTTTTAAAAAACTAAATTACATACAAACTACCGCATAAAATCCCTCAAACTATACAAACGTTTATCGTATAATATATATAC<br>ATTATTGTTTATTCTATCATTATTAGAGAAACTCCACTACCAAAACCATCATTAAAACAAAAATTTACATGCCACTTAACTCCCCTCACAAACAATCGTTATTTATATTGT<br>TAATTAGCAAACACAAAACCCGC
KY039390	DBKY039390_Easter Island_Hap_D1a	ATTTTATTTTTTAACCTAACTCCCCTACTAAGTGTACCCCCCCTTTNCCCCCCAGGGGGGGTATACTATGCATAATCGTGCATACATTTATATACCACATATATTATGGT<br>ACCGGTAATATATACTATATATGTACTAAACCCATTATATGTATACGGGCATTAATCTATATTCCACATTTCTCCCAATGTCCATTCTATGCATGATCCAGGACATACTC<br>ATTCACCCTCCCCATAGACAGCTCCAAACCACTACCAAGTCACCTAACTATGAATGGTTGCAGGACATAAATCTTACTCTCATGCTCTTCCCCCAACAAGTCACCTAAC<br>TATGAATGGTTGCAGGACATACATTTAACTACC--<br>ATGTTCTAACCCATTTGGTTATGCTCGCCGTATCAGATGGATTTATTGATCGTCCACCTCACGAGAGATCAGCAACCCCTGCCTGTAATGTACTTCATGACCAGTCTCA<br>GGCCCATTCTTTCCCCCTACACCCCTCGCCCTACTTGCCTTCCACCGTACCTCTGGTTCCTCGGTCAGGCACATCCCATGCATAACTCCTGAACTTTCTCACTTTTCAC<br>GAAGTCATCTGTGGATTATCTTCCCCTCTTTAGTCCGTGATCGCGGCATCTTCTCTCTTCTATTGCTGTTGGTTCCTTCTCTTTTTGGGGCTTCTTCACAGGTTACCCTT<br>CACAGTGCGGGTGCGGAGTGCTATTCAAGTGAAGCCTGGACTACACCTGCGTTGCGTCCTATCCTAGTCCTCTCGTGTCCCTCGATGAGACGGTTTGCGTGTATGGGG<br>AATCATCTTGACACTGATGCACTTTGGATCGCATTTGGTTATGGTTCTTCCACCCCCCNN-<br>GGTAAATGGTGCTATTTAGTGAATGCTTGTCGGACATATTTTTATCAATTTTCACTTCCTCTATTTTCTTCACAAAACTAGGAAATTCACCACAATTTTTTCTTTGTTATT<br>TTTTAATTTTTTTTTTATTTTTTAAAAACATTTTTTAAAAAACTAAATTACATACAAACTACCGCATAAAATCCCTCAAACTATACAAACGTTTATCGTATAATATATATAC<br>ATTATTGTTTATTCTATCATTATTAGAGAAACTCCACTACCAAAACCATCATTAAAACAAAAATTTACATGCCACTTAACTCCCCTCACAAACAATCGTTATTTATATTGT<br>TAATTAGCAAACACAAAACCCGC
KY039391	DBKY039391_Fiji_Hap_D1a	ATTTTATTTTTTAACCTAACTCCCCTACTAAGTGTACCCCCCCTTTCCCCCCCAGGGGGGGTATACTATGCATAATCGTGCATACATTTATATACCACATATATTATGGT<br>ACCGGTAATATATACTATATATGTACTAAACCCATTATATGTATACGGGCATTAATCTATATTCCACATTTCTCCCAATGTCCATTCTATGCATGATCCAGGACATACTC<br>ATTCACCCTCCCCATAGACAGCTCCAAACCACTACCAAGTCACCTAACTATGAATGGTTGCAGGACATAAATCTTACTCTCATGCTCTTCCCCCAACAAGTCACCTAAC<br>TATGAATGGTTGCAGGACATACATTTAACTACC--<br>ATGTTCTAACCCATTTGGTTATGCTCGCCGTATCAGATGGATTTATTGATCGTCCACCTCACGAGAGATCAGCAACCCCTGCCTGTAATGTACTTCATGACCAGTCTCA<br>GGCCCATTCTTTCCCCCTACACCCCTCGCCCTACTTGCCTTCCACCGTACCTCTGGTTCCTCGGTCAGGCACATCCCATGCATAACTCCTGAACTTTCTCACTTTTCAC<br>GAAGTCATCTGTGGATTATCTTCCCCTCTTTAGTCCGTGATCGCGGCATCTTCTCTCTTCTATTGCTGTTGGTTCCTTCTCTTTTTGGGGCTTCTTCACAGGTTACCCTT<br>CACAGTGCGGGTGCGGAGTGCTATTCAAGTGAAGCCTGGACTACACCTGCGTTGCGTCCTATCCTAGTCCTCTCGTGTCCCTCGATGAGACGGTTTGCGTGTATGGGG<br>AATCATCTTGACACTGATGCACTTTGGATCGCATTTGGTTATGGTTCTTCCANCCCCCCC-<br>GGTAAATGGTGCTATTTAGTGAATGCTTGTCGGACATATTTTTATCAATTTTCACTTCCTCTATTTTCTTCACAAAACTAGGAAATTCACCACAATTTTTTCTTTGTTATT<br>TTTTAATTTTTTTTTTATTTTTTAAAAACATTTTTTAAAAAACTAAATTACATACAAACTACCGCATAAAATCCCTCAAACTATACAAACGTTTATCGTATAATATATATAC<br>ATTATTGTTTATTCTATCATTATTAGAGAAACTCCACTACCAAAACCATCATTAAAACAAAAATTTACATGCCACTTAACTCCCCTCACAAACAATCGTTATTTATATTGT<br>TAATTAGCAAACACAAAACCCGC

KY039392	DBKY039392_Vanuatu_Hap_D1a	ATTTTATTTTTTAACCTAACTCCCCTACTAAGTGTACCCCCCCTTTCCCCCCCAGGGGGGGTATACTATGCATAATCGTGCATACATTTATATACCACATATATTATGGT<br>ACCGGTAATATATACTATATATGTACTAAACCCATTATATGTATACGGGCATTAATCTATATTCCACATTTCTCCCAATGTCCATTCTATGCATGATCCAGGACATACTC<br>ATTCACCCTCCCCATAGACAGCTCCAAACCACTACCAAGCCACCTAACTATGAATGGTTGCAGGACATAAATCTTACTCTCATGCTCTTCCCCCAACAAGTCACCTAAC<br>TATGAATGGTTGCAGGACATACATTTAACTACC--<br>ATGTTCTAACCCATTTGGTTATGCTCGCCGTATCAGATGGATTTATTGATCGTCCACCTCACGAGAGATCAGCAACCCCTGCCTGTAATGTACTTCATGACCAGTCTCA<br>GGCCCATTCTTTCCCCCTACACCCCTCGCCCTACTTGCCTTCCACCGTACCTCTGGTTCCTCGGTCAGGCACATCCCATGCATAACTCCTGAACTTTCTCACTTTTCAC<br>GAAGTCATCTGTGGATTATCTTCCCCTCTTTAGTCCGTGATCGCGGCATCTTCTCTCTTCTATTGCTGTTGGTTCCTTCTCTTTTTGGGGCTTCTTCACAGGTTACCCTT<br>CACAGTGCGGGTGCGGAGTGCTATTCAAGTGAAGCCTGGACTACACCTGCGTTGCGTCCTATCCTAGTCCTCTCGTGTCCCTCGATGAGACGGTTTGCGTGTATGGGG<br>AATCATCTTGACACTGATGCACTTTGGATCGCATTTGGTTATGGTTCTTCCANCCCCCCC-<br>GGTAAATGGTGCTATTTAGTGAATGCTTGTCGGACATATTTTTATCAATTTTCACTTCCTCTATTTTCTTCACAAAACTAGGAAATTCACCACAATTTTTTCTTTGTTATT<br>TTTTAATTTTTTTTTTATTTTTTAAAAACATTTTTTAAAAAACTAAATTACATACAAACTACCGCATAAAATCCCTCAAACTATACAAACGTTTATCGTATAATATATATAC<br>ATTATTGTTTATTCTATCATTATTAGAGAAACTCCACTACCAAAACCATCATTAAAACAAAAATTTACATGCCACTTAACTCCCCTCACAAACAATCGTTATTTATATTGT<br>TAATTAGCAAACACAAAACCCGC
KY039393	DBKY039393_Vanuatu_Hap_D1a	ATTTTATTTTTTAACCTAACTCCCCTACTAAGTGTACCCCCCCTTTCCCCCCCAGGGGGGGTATACTATGCATAATCGTGCATACATTTATATACCACATATATTATGGT<br>ACCGGTAATATATACTATATATGTACTAAACCCATTATATGTATACGGGCATTAATCTATATTCCACATTTCTCCCAATGTCCATTCTATGCATGATCCAGGACATACTC<br>ATTCACCCTCCCCATAGACAGCTCCAAACCACTACCAAGCCACCTAACTATGAATGGTTGCAGGACATAAATCTTACTCTCATGCTCTTCCCCCAACAAGTCACCTAAC<br>TATGAATGGTTGCAGGACATACATTTAACTACC--<br>ATGTTCTAACCCATTTGGTTATGCTCGCCGTATCAGATGGATTTATTGATCGTCCACCTCACGAGAGATCAGCAACCCCTGCCTGTAATGTACTTCATGACCAGTCTCA<br>GGCCCATTCTTTCCCCCTACACCCCTCGCCCTACTTGCCTTCCACCGTACCTCTGGTTCCTCGGTCAGGCACATCCCATGCATAACTCCTGAACTTTCTCACTTTTCAC<br>GAAGTCATCTGTGGATTATCTTCCCCTCTTTAGTCCGTGATCGCGGCATCTTCTCTCTTCTATTGCTGTTGGTTCCTTCTCTTTTTGGGGCTTCTTCACAGGTTACCCTT<br>CACAGTGCGGGTGCGGAGTGCTATTCAAGTGAAGCCTGGACTACACCTGCGTTGCGTCCTATCCTAGTCCTCTCGTGTCCCTCGATGAGACGGTTTGCGTGTATGGGG<br>AATCATCTTGACACTGATGCACTTTGGATCGCATTTGGTTATGGTTCTTCCACCCNCCCC-<br>GGTAAATGGTGCTATTTAGTGAATGCTTGTCGGACATATTTTTATCAATTTTCACTTCCTCTATTTTCTTCACAAAACTAGGAAATTCACCACAATTTTTTCTTTGTTATT<br>TTTTAATTTTTTTTTTATTTTTTAAAAACATTTTTTAAAAAACTAAATTACATACAAACTACCGCATAAAATCCCTCAAACTATACAAACGTTTATCGTATAATATATATAC<br>ATTATTGTTTATTCTATCATTATTAGAGAAACTCCACTACCAAAACCATCATTAAAACAAAAATTTACATGCCACTTAACTCCCCTCACAAACAATCGTTATTTATATTGT<br>TAATTAGCAAACACAAAACCCGC
KY039394	[illegible]BKY039394_Papua New Guinea_Hap_[illegible]	ATTTTATTTTTTAACCTAACTCCCCTACTAAGTGTACCCCCCCTTTCCCCCCCAGGGGGGGTATACTATGCATAATCGTGCATACATTTATATACCACATATATTATGGT<br>ACCGGTAATATATACTATATATGTACTAAACCCATTATATGTATACGGGCATTAATCTATATTCCACATTTCTCCCAATGTCCATTCTATGCATGATCCAGGACATACTC<br>ATTCACCCTCCCCATAGACAGCTCCAAACCACTACCAAGTCACCTAACTATGAATGGTTGCAGGACATAAATCTTACTCTCATGCTCTTCCCCCAACAAGTCACCTAAC<br>TATGAATGGTTACAGGACATACATTTAACTACC--<br>ATGTTCTAACCCATTTGGTTATGCTCGCCGTATCAGATGGATTTATTGATCGTCCACCTCACGAGAGATCAGCAACCCCTGCCTGTAATGTACTTCATGACCAGTCTCA<br>GGCCCATTCTTTCCCCCTACACCCCTCGCCCTACTTGCCTTCCACCGTACCTCTGGTTCCTCGGTCAGGCACATCCCATGCATAACTCCTGAACTTTCTCACTTTTCAC<br>GAAGTCATCTGTGGATTATCTTCCCCTCTTTAGTCCGTGATCGCGGCATCTTCTCTCTTCTATTGCTGTTGGTTCCTTCTCTTTTTGGGGCTTCTTCACAGGTTACCCTT<br>CACAGTGCGGGTGCGGAGTGCTATTCAAGTGAAGCCTGGACTACACCTGCGTTGCGTCCTATCCTAGTCCTCTCGTGTCCCTCGATGAGACGGTTTGCGTGTATGGGG<br>AATCATCTTGACACTGATGCACTTTGGATCGCATTTGGTTATGGTTCTTCCANCCCCCCC-<br>GGTAAATGGTGCTATTTAGTGAATGCTTGTCGGACATATTTTTATCAATTTTCACTTCCTCTATTTTCTTCACAAAACTAGGAAATTCACCACAATTTTTTCTTTGTTATT<br>TTTTAATTTTTTTTTTATTTTTTAAAAACATTTTTTAAAAAACTAAATTACATACAAACTACCGCATAAAATCCCTCAAACTATACAAACGTTTATCGTATAATATATATAC<br>ATTATTGTTTATTCTATCATTATTAGAGAAACTCCACTACCAAAACCATCATTAAAACAAAAATTTACATGCCACTTAACTCCCCTCACAAACAATCGTTATTTATATTGT<br>TAATTAGCAAACACAAAACCCGC

KY039395	DBKY039395_Indonesia_Hap_D1	ATTTTATTTTTTAACCTAACTCCCCTACTAAGTGTACCCCCCCTTTCCCCCCCAGGGGGGGTATACTATGCATAATCGTGCATACATTTATATACCACATATATTATGGT<br>ACCGGTAATATATACTATATATGTACTAAACCCATTATATGTATACGGGCATTAATCTATATTCCACATTTCTCCCAATGTCCATTCTATGCATGATCCAGGACATACTC<br>ATTCACCCTCCCCATAGACAGCTCCAAACCACTACCAAGTCACCTAACTATGAATGGTTGCAGGACATAAATCTTACTCTCATGCTCTTCCCCCAACAAGTCACCTAAC<br>TATGAATGGTTACAGGACATACATTTAACTACC---<br>ATGTTCTAACCCATTTGGTTATGCTCGTCGTATCAGATGGATTTATTGATCGTCCACCTCACGAGAGATCAGCAACCCCTGCCTGTAATGTACTTCATGACCAGTCTCA<br>GGCCCATTCTTTCCCCCTACACCCCTCGCCCTACTTGCCTTCCACCGTACCTCTGGTTCCTCGGTCAGGCACATCCCATGCATAACTCCTGAACTTTCTCACTTTTCAC<br>GAAGTCATCTGTGGATTATCTTCCCCTCTTTAGTCCGTGATCGCGGCATCTTCTCTCTTCTATTGCTGTTGGTTCCTTCTCTTTTTGGGGCTTCTTCACAGGTTGCCCTT<br>CACAGTGCGGGTGCGGAGTGCTATTCAAGTGAAGCCTGGACTACACCTGCGTTGCGTCCTATCCTAGTCCTCTCGTGTCCCTCGATGAGACGGTTTGCGTGTATGGGG<br>AATCATCTTGACACTGATGCACTTTGGATCGCATTTGGTTATGGTTCTTCCACCCCCCCC-<br>GGTAAATGGTGCTATTTAGTGAATGCTTGTCGGACATATTTTTATCAATTTTCACTTCCTCTATTTTCTTCACAAAACTAGGAAATTCACCACAATTTTTTCTTTGTTATT<br>TTTTAATTTTTTTTTTATTTTTTAAAAACATTTTTTAAAAAACTAAATTACATACAAACTACCGCATAAAATCCCTCAAACTATACAAACGTTTATCGTATAATATATATAC<br>ATTATTGTTTATTCTATCATTATTAGAGAAACTCCACTACCAAAACCATCATTAAAACAAAAATTTACATGCCACTTAACTCCCCTCACAAACAATCGTTATTTATATTGT<br>TAATTAGCAAACACAAAACCCGC
KY039396	DBKY039396_Philippines_Hap_D1a	ATTTTATTTTTTAACCTAACTCCCCTACTAAGTGTACCCCCCCTTTCCCCCCCAGGGGGGGTATACTATGCATAATCGTGCATACATTTATATACCACATATATTATGGT<br>ACCGGTAATATATACTATATATGTACTAAACCCATTATATGTATACGGGCATTAATCTATATTCCACATTTCTCCCAATGTCCATTCTATGCATGATCCAGGACATACTC<br>ATTCACCCTCCCCATAGACAGCTCCAAACCACTACCAAGTCACCTAACTATGAATGGTTGCAGGACATAAATCTCACTCTCATGCTCTTCCCCCAACAAGTCACCTAAC<br>TATGAATGGTTGCAGGACATACATCTAACTACC---<br>ATGTTCTAACCCATTTGGTTATGCTCGCCGTATCAGATGGATTTATTGATCGTCCACCTCACGAGAGATCAGCAACCCCTGCCTGTAATGTACTTCATGACCAGTCTCA<br>GGCCCATTCTTTCCCCCTACACCCCTCGCCCTACTTGCCTTCCACCGTACCTCTGGTTCCTCGGTCAGGCACATCCCATGCATAACTCCTGAACTTTCTCACTTTTCAC<br>GAAGTCATCTGTGGATTATCTTCCCCTCTTTAGTCCGTGATCGCGGCATCTTCTCTCTTCTATTGCTGTTGGTTCCTTCTCTTTTTGGGGCTTCTTCACAGGTTGCCCTT<br>CACAGTGCGGGTGCGGAGTGCTATTCAAGTGAAGCCTGGACTACACCTGCGTTGCGTCCTATCCTAGTCCTCTCGTGTCCCTCGATGAGACGGTTTGCGTGTATGGGG<br>AATCATCTTGACACTGATGCACTTTGGATCGCATTTGGTTATGGTTCTTCCACCCCCCCC-<br>GGTAAATGGTGCTATTTAGTGAATGCTTGTCGGACATATTTTTATCAATTTTCACTTCCTCTATTTTCTTCACAAAACTAGGAAATTCACCACAATTTTTTCTTTGTTATT<br>TTTTAATTTTTTTTTTATTTTTTAAAAACATTTTTTAAAAAACTAAATTACATACAAACTACCGCATAAAATCCCTCAAACTATACAAACGTTTATCGTATAATATATATAC<br>ATTATTGTTTATTCTATCATTATTAGAGAAACTCCACTACCAAAACCATCATTAAAACAAAAATTTACATGCCACTTAACTCCCCTCACAAACAATCGTTATTTATATTGT<br>TAATTAGCAAACACAAAACCCGC
KY039397	DBKY039397_Philippines_Hap_D1	ATTTTATTTTTTAACCTAACTCCCCTACTAAGTGTACCCCCCCTTTCCCCCCCAGGGGGGGTATACTATGCATAATCGTGCATACATTTATATACCACATATATTATGGT<br>ACCGGTAATATATACTATATATGTACTAAACCCATTATATGTATACGGGCATTAATCTATATTCCACATTTCTCCCAATGTCCATTCTATGCATGATCCAGGACATACTC<br>ATTCACCCTCCCCATAGACAGCTCCAAACCACTACCAAGTCACCTAACTATGAATGGTTGCAGGACATAAATCTCACTCTCATGCTCTTCCCCCAACAAGTCACCTAAC<br>TATGAATGGTTACAGGACATACATTTAACTACC---<br>ATGTTCTAACCCATTTGGTTATGCTCGCCGTATCAGATGGATTTATTGATCGTCCACCTCACGAGAGATCAGCAACCCCTGCCTGTAATGTACTTCATGACCAGTCTCA<br>GGCCCATTCTTTCCCCCTACACCCCTCGCCCTACTTGCCTTCCACCGTACCTCTGGTTCCTCGGTCAGGCACATCCCATGCATAACTCCTGAACTTTCTCACTTTTCAC<br>GAAGTCATCTGTGGATTATCTTCCCCTCTTTAGTCCGTGATCGCGGCATCTTCTCTCTTCTATTGCTGTTGGTTCCTTCTCTTTTTGGGGCTTCTTCACAGGTTGCCCTT<br>CACAGTGCGGGTGCGGAGTGCTATTCAAGTGAAGCCTGGACTACACCTGCGTTGCGTCCTATCCTAGTCCTCTCGTGTCCCTCGATGAGACGGTTTGCGTGTATGGGG<br>AATCATCTTGACACTGATGCACTTTGGATCGCATTTGGTTATGGTTCTTCCACCCNCCCC-<br>GGTAAATGGTGCTATTTAGTGAATGCTTGTCGGACATATTTTTATCAATTTTCACTTCCTCTATTTTCTTCACAAAACTAGGAAATTCACCACAATTTTTTCTTTGTTATT<br>TTTTAATTTTTTTTTTATTTTTTAAAAACATTTTTTAAAAAACTAAATTACATACAAACTACCGCATAAAATCCCTCAAACTATACAAACGTTTATCGTATAATATATATAC<br>ATTATTGTTTATTCTATCATTATTAGAGAAACTCCACTACCAAAACCATCATTAAAACAAAAATTTACATGCCACTTAACTCCCCTCACAAACAATCGTTATTTATATTGT<br>TAATTAGCAAACACAAAACCCGC

KY039398	DBKY039398_Philippines_Hap_D1	ATTTTATTTTTTAACCTAACTCCCCTACTAAGTGTACCCCCCCTTTCCCCCCCAGGGGGGGTATACTATGCATAATCGTGCATACATTTATATACCACATATATTATGGT<br>ACCGGTAATATATACTATATATGTACTAAACCCATTATATGTATACGGGCATTAATCTATATTCCACATTTCTCCCAATGTCCATTCTATGCATGATCCAGGACATACTC<br>ATTCACCCTCCCCATAGACAGCTCCAAACCACTACCAAGTCACCTAACTATGAATGGTTGCAGGACATAAATCTCACTCTCATGCTCTTCCTCCAACAAGTCACCTAAC<br>TATGAATGGTTACAGGACATACATTTAACTACC--<br>ATGTTCTAACCCATTTGGTTATGCTCGCCGTATCAGATGGATTTATTGATCGTCCACCTCACGAGAGATCAGCAACCCCTGCCTGTAATGTACTTCATGACCAGTCTCA<br>GGCCCATTCTTTCCCCCTACACCCCTCGCCCTACTTGCCTTCCACCGTACCTCTGGTTCCTCGGTCAGGCACATCCCATGCATAACTCCTGAACTTTCTCACTTTTCAC<br>GAAGTCATCTGTGGATTATCTTCCCCTCTTTAGTCCGTGATCGCGGCATCTTCTCTCTTCTATTGCTGTTGGTTCCTTCTCTTTTTGGGGCTTCTTCACAGGTTGCCCTT<br>CACAGTGCGGGTGCGGAGTGCTATTCAAGTGAAGCCTGGACTACACCTGCGTTGCGTCCTATCCTAGTCCTCTCGTGTCCCTCGATGAGACGGTTTGCGTGTATGGGG<br>AATCATCTTGACACTGATGCACTTTGGATCGCATTTGGTTATGGTTCTTCCACCCNCCCC-<br>GGTAAATGGTGCTATTTAGTGAATGCTTGTCGGACATATTTTTATCAATTTTCACTTCCTCTATTTTCTTCACAAAACTAGGAAATTCACCACAATTTTTTCTTTGTTATT<br>TTTTAATTTTTTTTTTATTTTTTAAAAACATTTTTTAAAAAACTAAATTACATACAAACTACCGCATAAAATCCCTCAAACTATACAAACGTTTATCGTATAATATATATAC<br>ATTATTGTTTATTCTATCATTATTAGAGAAACTCCACTACCAAAACCATCATTAAAACAAAAATTTACATGCCACTTAACTCCCCTCACAAACAATCGTTATTTATATTGT<br>TAATTAGCAAACACAAAACCCGC
KY039399	DBKY039399_Philippines_Hap_D1	ATTTTATTTTTTAACCTAACTCCCCTACTAAGTGTACCCCCCCTTTCCCCCCCAGGGGGGGTATACTATGCATAATCGTGCATACATTTATATACCACATATATTATGGT<br>ACCGGTAATATATACTATATATGTACTAAACCCATTATATGTATACGGGCATTAATCTATATTCCACATTTCTCCCAATGTCCATTCTATGCATGATCCAGGACATACTC<br>ATTCACCCTCCCCATAGACAGCCCCAAACCACTACCAAGTCACCTAACTATGAATGGTTGCAGGACATAAATCTCACTCTCATGCTCTTCCCCCAACAAGTCACCTAAC<br>TATGAATGGTTACAGGACATACATTTAACTACC--<br>ATGTTCTAACCCATTTGGTTATGCTCGCCGTATCAGATGGATTTATTGATCGTCCACCTCACGAGAGATCAGCAACCCCTGCCTGTAATGTACTTCATGACCAGTCTCA<br>GGCCCATTCTTTCCCCCTACACCCCTCGCCCTACTTGCCTTCCACCGTACCTCTGGTTCCTCGGTCAGGCACATCCCATGCATAACTCCTGAACTTTCTCACTTTTCAC<br>GAAGTCATCTGTGGATTATCTTCCCCTCTTTAGTCCGTGATCGCGGCATCTTCTCTCTTCTATTGCTGTTGGTTCCTTCTCTTTTTGGGGCTTCTTCACAGGTTGCCCTT<br>CACAGTGCGGGTGCGGAGTGCTATTCAAGTGAAGCCTGGACTACACCTGCGTTGCGTCCTATCCTAGTCCTCTCGTGTCCCTCGATGAGACGGTTTGCGTGTATGGGG<br>AATCATCTTGACACTGATGCACTTTGGATCGCATTTGGTTATGGTTCTTCCACCCNCCCC-<br>GGTAAATGGTGCTATTTAGTGAATGCTTGTCGGACATATTTTTATCAATTTTCACTTCCTCTATTTTCTTCACAAAACTAGGAAATTCACCACAATTTTTTCTTTGTTATT<br>TTTTAATTTTTTTTTTATTTTTTAAAAACATTTTTTAAAAAACTAAATTACATACAAACTACCGCATAAAATCCCTCAAACTATACAAACGTTTATCGTATAATATATATAC<br>ATTATTGTTTATTCTATCATTATTAGAGAAACTCCACTACCAAAACCATCATTAAAACAAAAATTTACATGCCACTTAACTCCCCTCACAAACAATCGTTATTTATATTGT<br>TAATTAACAAACACAAAACCCGC
KY039400	DBKY039400_Philippines_Hap_D1a	ATTTTATTTTTTAACCTAACTCCCCTACTAAGTGTACCCCCCCTTTCCCCCCCAGGGGGGGTATACTATGCATAATCGTGCATACATTTATATACCACATATATTATGGT<br>ACCGGTAATATATACTATATATGTACTAAACCCATTATATGTATACGGGCATTAATCTATATTCCACATTTCTCCCAATGTCCATTCTATGCATGATCCAGGACATACTC<br>ATTCACCCTCCCCATAGACAGCTCTAAACCACTACCAAGTCACCTAACTATGAATGGTTGCAGGACATAAATCTTACTCTCATGCTCTTCCCCCAACAAGTCACCTAAC<br>TATGAATGGTTGCAGGACATACATTTAACTACC--<br>ATGTTCTAACCCATTTGGTTATGCTCGCCGTATCAGATGGATTTATTGATCGTCCACCTCACGAGAGATCAGCAACCCCTGCCTGTAATGTACTTCATGACCAGTCTCA<br>GGCCCATTCTTTCCCCCTACACCCCTCGCCCTACTTGCCTTCCACCGTACCTCTGGTTCCTCGGTCAGGCACATCCCATGCATAACTCCTGAACTTTCTCACTTTTCAC<br>GAAGTCATCTGTGGATTATCTTCCCCTCTTTAGTCCGTGATCGCGGCATCTTCTCTCTTCTATTGCTGTTGGTTCCTTCTCTTTTTGGGGCTTCTTCACAGGTTACCCTT<br>CACAGTGCGGGTGCGGAGTGCTATTCAAGTGAAGCCTGGACTACACCTGCGTTGCGTCCTATCCTAGTCCTCTCGTGTCCCTCGATGAGACGGTTTGCGTGTATGGGG<br>AATCATCTTGACACTGATGCACTTTGGATCGCATTTGGTTATGGTTCTTCCACCCCCCCC-<br>GGTAAATGGTGCTATTTAGTGAATGCTTGTCGGACATATTTTTATCAATTTTCACTTCCTCTATTTTCTTCACAAAACTAGGAAATTCACCACAATTTTTTCTTTGTTATT<br>TTTTAATTTTTTTTTTATTTTTTAAAAACATTTTTTAAAAAACTAAATTACATACAAACTACCGCATAAAATCCCTCAAACTATACAAACGTTTATCGTATAATATATATAC<br>ATTATTGTTTATTCTATCATTATTAGAGAAACTCCACTACCAAAACCATCATTAAAACAAAAATTTACATGCCACTTAACTCCCCTCACAAACAATCGTTATTTATATTGT<br>TAATTAGCAAACACAAAACCCGC

KY039401	DBKY039401_Philippines_Hap_D1	ATTTTATTTTTTAACCTAACTCCCCTACTAAGTGTACCCCCCCTTTCCCCCCCAGGGGGGGTATACTATGCATAATCGTGCATACATTTATATACCACATATATTATGGT<br>ACCGGTAATATATACTATATATGTACTAAACCCATTATATGTATACGGGCATTAATCTATATTCCACATTTCTCCCAATGTCCATTCTATGCATGATCCAGGACATACTC<br>ATTCACCCTCCCCATAGACAGCTCCAAACCACTACCAAGTCACCTAACTATGAATGGTTGCAGGACATAAATCTCACTCTCATGCTCCTCCCCCAACAAGCCACCTAA<br>CTATGAATGGTTACAGGACATACATTTAACTACC---<br>ATGTTCTAACCCATTTGGTTATGCTCGCCGTATCAGATGGATTTATTGATCGTCCACCTCACGAGAGATCAGCAACCCCTGCCTGTAATGTACTTCATGACCAGTCTCA<br>GGCCCATTCTTTCCCCCTACACCCCTCGCCCTACTTGCCTTCCACCGTACCTCTGGTTCCTCGGTCAGGCACATCCCATGCATAACTCCTGAACTTTCTCACTTTTCAC<br>GAAGTCATCTGTGGATTATCTTCCCCTCTTTAGTCCGTGATCGCGGCATCTTCTCTCTTCTATTGCTGTTGGTTCCTTCTCTTTTTGGGGCTTCTTCACAGGTTGCCCTT<br>CACAGTGCGGGTGCGGAGTGCTATTCAAGTGAAGCCTGGACTACACCTGCGTTGCGTCCTATCCTAGTCCTCTCGTGTCCCTCGATGAGACGGTTTGCGTGTATGGGG<br>AATCATCTTGACACTGATGCACTTTGGATCGCATTTGGTTATGGTTCTTCCACCCNCCCC-<br>GGTAAATGGTGCTATTTAGTGAATGCTTGTCGGACATATTTTTATCAATTTTCACTTCCTCTATTTTCTTCACAAAACTAGGAAATTCACCACAATTTTTTCTTTGTTATT<br>TTTTAATTTTTTTTTTATTTTTTAAAAACATTTTTTAAAAAACTAAATTACATACAAACTACCGCATAAAATCCCTCAAACTATACAAACGTTTATCGTATAATATATATAC<br>ATTATTGTTTATTCTATCATTATTAGAGAAACTCCACTACCAAAACCATCATTAAAACAAAAATTTACATGCCACTTAACTCCCCTCACAAACAATCGTTATTTATATTGT<br>TAATTAGCAAACACAAAACCCGC
KY039402	DBKY039402_Philippines_Hap_D1	ATTTTATTTTTTAACCTAACTCCCCTACTAAGTGTACCCCCCCTTTCCCCCCCAGGGGGGGTATACTATGCATAATCGTGCATACATTTATATACCACATATATTATGGT<br>ACCGGTAATATATACTATATATGTACTAAACCCATTATATGTATACGGGCATTAATCTATATTCCACATTTCTCCCAATGTCCATTCTATGCATGATCCAGGACATACTC<br>ATTCACCCTCCCCATAAACAGCTCCAAACCACTACCAAGTCACCTAACTATGAATGGTTGCAGGACATAAATCTCACTCTCATGCTCCTCCCCCAACAAGCCACCTAA<br>CTATGAATGGTTACAGGACATACATTTAACTACC---<br>ATGTTCTAACCCATTTGGTTATGCTCGCCGTATCAGATGGATTTATTGATCGTCCACCTCACGAGAGATCAGCAACCCCTGCCTGTAATGTACTTCATGACCAGTCTCA<br>GGCCCATTCTTTCCCCCTACACCCCTCGCCCTACTTGCCTTCCACCGTACCTCTGGTTCCTCGGTCAGGCACATCCCATGCATAACTCCTGAACTTTCTCACTTTTCAC<br>GAAGTCATCTGTGGATTATCTTCCCCTCTTTAGTCCGTGATCGCGGCATCTTCTCTCTTCTATTGCTGTTGGTTCCTTCTCTTTTTGGGGCTTCTTCACAGGTTGCCCTT<br>CACAGTGCGGGTGCGGAGTGCTATTCAAGTGAAGCCTGGACTACACCTGCGTTGCGTCCTATCCTAGTCCTCTCGTGTCCCTCGATGAGACGGTTTGCGTGTATGGGG<br>AATCATCTTGACACTGATGCACTTTGGATCGCATTTGGTTATGGTTCTTCCACCCNCCCC-<br>GGTAAATGGTGCTATTTAGTGAATGCTTGTCGGACATATTTTTATCAATTTTCACTTCCTCTATTTTCTTCACAAAACTAGGAAATTCACCACAATTTTTTCTTTGTTATT<br>TTTTAATTTTTTTTTTATTTTTTAAAAACATTTTTTAAAAAACTAAATTACATACAAACTACCGCATAAAATCCCTCAAACTATACAAACGTTTATCGTATAATATATATAC<br>ATTATTGTTTATTCTATCATTATTAGAGAAACTCCACTACCAAAACCATCATTAAAACAAAAATTTACATGCCACTTAACTCCCCTCACAAACAATCGTTATTTATATTGT<br>TAATTAGCAAACACAAAACCCGC
KY039403	DBKY039403_Philippines_Hap_D1	ATTTTATTTTTTAACCTAACTCCCCTACTAAGTGTACCCCCCCTTTCCCCCCCAGGGGGGGTATACTATGCATAATCGTGCATACATTTATATACCACATATATTATGGT<br>ACCGGTAATATATACTATATATGTACTAAACCCATTATATGTATACGGGCATTAATCTATATTCCACATTTCTCCCAATGTCCATTCTATGCATGATCCAGGACATACTC<br>ATTCACCCTCCCCATAGACAGCTCCAAACCACTACCAAGTCACCTAACTATGAATGGTTGCAGGACATAAATCTTACTCTCATGCTCTTCCCCCAACAAGTCACCTAAC<br>TATGAATGGTTACAGGACATACATTTAACTACC---<br>ATGTTCTAACCCATTTGGTTATGCTCGCCGTATCAGATGGATTTATTGATCGTCCACCTCACGAGAGATCAGCAACCCCTGCCTGTAATGTACTTCATGACCAGTCTCA<br>GGCCCATTCTTTCCCCCTACACCCCTCGCCCTACTTGCCTTCCACCGTACCTCTGGTTCCTCGGTCAGGCACATCCCATGCATAACTCCTGAACTTTCTCACTTTTCAC<br>GAAGTCATCTGTGGATTATCTTCCCCTCTTTAGTCCGTGATCGCGGCATCTTCTCTCTTCTATTGCTGTTGGTTCCTTCTCTTTTTGGGGCTTCTTCACAGGTTACCCTT<br>CACAGTGCGGGTGCGGAGTGCTATTCAAGTGAAGCCTGGACTACACCTGCGTTGCGTCCTATCCTAGTCCTCTCGTGTCCCTCGATGAGACGGTTTGCGTGTATGGGG<br>AATCATCTTGACACTGATGCACTTTGGATCGCATTTGGTTATGGTTCTTCCACCCNCCCC-<br>GGTAAATGGTGCTATTTAGTGAATGCTTGTCGGACATATTTTTATCAATTTTCACTTCCTCTATTTTCTTCACAAAACTAGGAAATTCACCACAATTTTTTCTTTGTTATT<br>TTTTAATTTTTTTTTTATTTTTTAAAAACATTTTTTAAAAAACTAAATTACATACAAACTACCGCATAAAATCCCTCAAACTATACAAACGTTTATCGTATAATATATATAC<br>ATTATTGTTTATTCTATCATTATTAGAGAAACTCCACTACCAAAACCATCATTAAAACAAAAATTTACATGCCACTTAACTCCCCTCACAAACAATCGTTATTTATATTGT<br>TAATTAGCAAACACAAAACCCGC

KY039404	DBKY039404_Philippines_Hap_D1a	ATTTTATTTTTTAACCTAACTCCCCTACTAAGTGTACCCCCCCTTTCCCCCCCAGGGGGGGTATACTATGCATAATCGTGCATACATTTATATACCACATATATTATGGT<br>ACCGGTAATATATACTATATATGTACTAAACCCATTATATGTATACGGGCATTAATCTATATTCCACATTTCTCCCAATGTCCATTCTATGCATGATCCAGGACATACTC<br>ATTCACCCTCCCCATAGACAGCTCCAAACCACTACCAAGTCACCTAACTATGAATGGTTGCAGGACATAAATCTTACTCTCATGCTCTTCCCCCAACAAGTCACCTAAC<br>TATGAATGGTTGCAGGACATACATTTAACTACC--<br>ATGTTCTAACCCATTTGGTTATGCTCGCCGTATCAGATGGATTTATTGATCGTCCACCTCACGAGAGATCAGCAACCCCTGCCTGTAATGTACTTCATGACCAGTCTCA<br>GGCCCATTCTTTCCCCCTACACCCCTCGCCCTACTTGCCTTCCACCGTACCTCTGGTTCCTCGGTCAGGCACATCCCATGCATAACTCCTGAACTTTCTCACTTTTCAC<br>GAAGTCATCTGTGGATTATCTTCCCCTCTTTAGTCCGTGATCGCGGCATCTTCTCTCTTCTATTGCTGTTGGTTCCTTCTCTTTTTGGGGCTTCTTCACAGGTTACCCTT<br>CACAGTGCGGGTGCGGAGTGCTATTCAAGTGAAGCCTGGACTACACCTGCGTTGCGTCCTATCCTAGTCCTCTCGTGTCCCTCGATGAGACGGTTTGCGTGTATGGGG<br>AATCATCTTGACACTGATGCACTTTGGATCGCATTTGGTTATGGTTCTTCCANCCCCCCC-<br>GGTAAATGGTGCTATTTAGTGAATGCTTGTCGGACATATTTTTATCAATTTTCACTTCCTCTATTTTCTTCACAAAACTAGGAAATTCACCACAATTTTTTCTTTGTTATT<br>TTTTAATTTTTTTTTTATTTTTTAAAAACATTTTTTAAAAAACTAAATTACATACAAACTACCGCATAAAATCCCTCAAACTATACAAACGTTTATCGTATAATATATATAC<br>ATTATTGTTTATTCTATCATTATTAGAGAAACTCCACTACCAAAACCATCATTAAAACAAAAATTTACATGCCACTTAACTCCCCTCACAAACAATCGTTATTTATATTGT<br>TAATTAGCAAACACAAAACCCGC
KY039405	DBKY039405_Philippines_Hap_D1a	ATTTTATTTTTTAACCTAACTCCCCTACTAAGTGTACCCCCCCTTTCCCCCCCAGGGGGGGTATACTATGCATAATCGTGCATACATTTATATACCACATATATTATGGT<br>ACCGGTAATATATACTATATATGTACTAAACCCATTATATGTATACGGGCATTAATCTATATTCCACATTTCTCCCAATGTCCATTCTATGCATGATCCAGGACATACTC<br>ATTCACCCTCCCCATAGACAGCTCCAAACCACTACCAAGTCACCTAACTATGAATGGTTGCAGGACATAAATCTTACTCTCATGCTCTTCCCCCAACAAGTCACCTAAC<br>TATGAATGGTTGCAGGACATACATTTAACTACC--<br>ATGTTCTAACCCATTTGGTTATGCTCGCCGTATCAGATGGATTTATTGATCGTCCACCTCACGAGAGATCAGCAACCCCTGCCTGTAATGTACTTCATGACCAGTCTCA<br>GGCCCATTCTTTCCCCCTACACCCCTCGCCCTACTTGCCTTCCACCGTACCTCTGGTTCCTCGGTCAGGCACATCCCATGCATAACTCCTGAACTTTCTCACTTTTCAC<br>GAAGTCATCTGTGGATTATCTTCCCCTCTTTAGTCCGTGATCGCGGCATCTTCTCTCTTCTATTGCTGTTGGTTCCTTCTCTTTTTGGGGCTTCTTCACAGGTTACCCTT<br>CACAGTGCGGGTGCGGAGTGCTATTCAAGTGAAGCCTGGACTACACCTGCGTTGCGTCCTATCCTAGTCCTCTCGTGTCCCTCGATGAGACGGTTTGCGTGTATGGGG<br>AATCATCTTGACACTGATGCACTTTGGATCGCATTTGGTTATGGTTCTTCCACCCNCCCC-<br>GGTAAATGGTGCTATTTAGTGAATGCTTGTCGGACATATTTTTATCAATTTTCACTTCCTCTATTTTCTTCACAAAACTAGGAAATTCACCACAATTTTTTCTTTGTTATT<br>TTTTAATTTTTTTTTTATTTTTTAAAAACATTTTTTAAAAAACTAAATTACATACAAACTACCGCATAAAATCCCTCAAACTATACAAACGTTTATCGTATAATATATATAC<br>ATTATTGTTTATTCTATCATTATTAGAGAAACTCCACTACCAAAACCATCATTAAAACAAAAATTTACATGCCACTTAACTCCCCTCACAAACAATCGTTATTTATATTGT<br>TAATTAGCAAACACAAAACCCGC
KY039406	DBKY039406_Philippines_Hap_D1	ATTTTATTTTTTAACCTAACTCCCCTACTAAGTGTACCCCCCCTTTCCCCCCCAGGGGGGGTATACTATGCATAATCGTGCATACATTTATATACCACATATATTATGGT<br>ACCGGTAATATATACTATATATGTACTAAACCCATTATATGTATACGGGCATTAATCTATATTCCACATTTCTCCCAATGTCCATTCTATGCATGATCCAGGACATACTC<br>ATTTACCCTCCCCATAGACAGCTCCAAACCACTACCAAGTCACCTAACTATGAATGGTTGCAGGACATAAATCTCACTCTCATGCTCTTCCCCCAACAAGTCACCTAAC<br>TATGAATGGTTACAGGACATACATTTAACTACC--<br>ATGTTCTAACCCATTTGGTTATGCTCGCCGTATCAGATGGATTTATTGATCGTCCACCTCACGAGAGATCAGCAACCCCTGCCTGTAATGTACTTCATGACCAGTCTCA<br>GGCCCATTCTTTCCCCCTACACCCCTCGCCCTACTTGCCTTCCACCGTACCTCTGGTTCCTCGGTCAGGCACATCCCATGCATAACTCCTGAACTTTCTCACTTTTCAC<br>GAAGTCATCTGTGGATTATCTTCCCCTCTTTAGTCCGTGATCGCGGCATCTTCTCTCTTCTATTGCTGTTGGTTCCTTCTCTTTTTGGGGCTTCTTCACAGGTTGCCCTT<br>CACAGTGCGGGTGCGGAGTGCTATTCAAGTGAAGCCTGGACTACACCTGCGTTGCGTCCTATCCTAGTCCTCTCGTGTCCCTCGATGAGACGGTTTGCGTGTATGGGG<br>AATCATCTTGACACTGATGCACTTTGGATCGCATTTGGTTATGGTTCTTCCACCCNCCCC-<br>GGTAAATGGTGCTATTTAGTGAATGCTTGTCGGACATATTTTTATCAATTTTCACTTCCTCTATTTTCTTCACAAAACTAGGAAATTCACCACAATTTTTTCTTTGTTATT<br>TTTTAATTTTTTTTTTATTTTTTAAAAACATTTTTTAAAAAACTAAATTACATACAAACTACCGCATAAAATCCCTCAAACTATACAAACGTTTATCGTATAATATATATAC<br>ATTATTGTTTATTCTATCATTATTAGAGAAACTCCACTACCAAAACCATCATTAAAACAAAAATTTACATGCCACTTAACTCCCCTCACAAACAATCGTTATTTATATTGT<br>TAATTAGCAAACACAAAACCCGC

KY039407	DBKY039407_Philippines_Hap_D1a	ATTTTATTTTTTAACCTAACTCCCCTACTAAGTGTACCCCCCCTTTCCCCCCCAGGGGGGGTATACTATGCATAATCGTGCATACATTTATATACCACATATATTATGGT<br>ACCGGTAATATATACTATATATGTACTAAACCCATTATATGTATACGGGCATTAATCTATATTCCACATTTCTCCCAATGTCCATTCTATGCATGATCCAGGACATACTC<br>ATTCACCCTCCCCATAGACAGCTCCAAACCACTACCAAGTCACCTAACTATGAATGGTTGCAGGACATAAATCTTACTCTCATGCTCTTCCCCCAACAAGTCACCTAAC<br>TATGAATGGTTGCAGGACATACATTTAACTACC---<br>ATGTTCTAACCCATTTGGTTATGCTCGCCGTATCAGATGGATTTATTGATCGTCCACCTCACGAGAGATCAGCAACCCCTGCCTGTAATGTACTTCATGACCAGTCTCA<br>GGCCCATTCTTTCCCCCTACACCCCTCGCCCTACTTGCCTTCCACCGTACCTCTGGTTCCTCGGTCAGGCACATCCCATGCATAACTCCTGAACTTTCTCACTTTTCAC<br>GAAGTCATCTGTGGATTATCTTCCCCTCTTTAGTCCGTGATCGCGGCATCTTCTCTCTTCTATTGCTGTTGGTTCCTTCTCTTTTTGGGGCTTCTTCACAGGTTACCCTT<br>CACAGTGCGGGTGCGGAGTGCTATTCAAGTGAAGCCTGGACTACACCTGCGTTGCGTCCTATCCTAGTCCTCTCGTGTCCCTCGATGAGACGGTTTGCGTGTATGGGG<br>AATCATCTTGACACTGATGCACTTTGGATCGCATTTGGTTATGGTTCTTCCACCCNCCCC-<br>GGTAAATGGTGCTATTTAGTGAATGCTTGTCGGACATATTTTTATCAATTTTCACTTCCTCTATTTTCTTCACAAAACTAGGAAATTCACCACAATTTTTTCTTTGTTATT<br>TTTTAATTTTTTTTTTATTTTTTAAAAACATTTTTTAAAAAACTAAATTACATACAAACTACCGCATAAAATCCCTCAAACTATACAAACGTTTATCGTATAATATATATAC<br>ATTATTGTTTATTCTATCATTATTAGAGAAACTCCACTACCAAAACCATCATTAAAACAAAAATTTACATGCCACTTAACTCCCCTCACAAACAATCGTTATTTATATTGT<br>TAATTAGCAAACACAAAACCCGC
KY039408	DBKY039408_Philippines_Hap_D1a	ATTTTATTTTTTAACCTAACTCCCCTACTAAGTGTACCCCCCCTTTCCCCCCCAGGGGGGGTATACTATGCATAATCGTGCATACATTTATATACCACATATATTATGGT<br>ACCGGTAATATATACTATATATGTACTAAACCCATTATATGTATACGGGCATTAATCTATATTCCACATTTCTCCCAATGTCCATTCTATGCATGATCCAGGACATACCC<br>ATTCACCCTCCCCATAGACAGCTCCAAACCACTACCAAGTCACCTAACTATGAATGGTTGCAGGACATAAATCTCACTCTCATGCTCTTCCCCCAACAAGTCACCTAAC<br>TATGAATGGTTGCAGGACATACATTTAACTACC---<br>ATGTTCTAACCCATTTGGTTATGCTCGCCGTATCAGATGGATTTATTGATCGTCCACCTCACGAGAGATCAGCAACCCCTGCCTGTAATGTACTTCATGACCAGTCTCA<br>GGCCCATTCTTTCCCCCTACACCCCTCGCCCTACTTGCCTTCCACCGTACCTCTGGTTCCTCGGTCAGGCACATCCCATGCATAACTCCTGAACTTTCTCACTTTTCAC<br>GAAGTCATCTGTGGATTATCTTCCCCTCTTTAGTCCGTGATCGCGGCATCTTCTCTCTTCTATTGCTGTTGGTTCCTTCTCTTTTTGGGGCTTCTTCACAGGTTGCCCTT<br>CACAGTGCGGGTGCGGAGTGCTATTCAAGTGAAGCCTGGACTACACCTGCGTTGCGTCCTATCCTAGTCCTCTCGTGTCCCTCGATGAGACGGTTTGCGTGTATGGGG<br>AATCATCTTGACACTGATGCACTTTGGATCGCATTTGGTTATGGTTCTTCCANCCCCCCC-<br>GGTAAATGGTGCTATTTAGTGAATGCTTGTCGGACATATTTTTATCAATTTTCACTTCCTCTATTTTCTTCACAAAACTAGGAAATTCACCACAATTTTTTCTTTGTTATT<br>TTTTAATTTTTTTTTTATTTTTTAAAAACATTTTTTAAAAAACTAAATTACATACAAACTACCGCATAAAATCCCTCAAACTATACAAACGTTTATCGTATAATATATATAC<br>ATTATTGTTTATTCTATCATTATTAGAGAAACTCCACTACCAAAACCATCATTAAAACAAAAATTTACATGCCACTTAACTCCCCTCACAAACAATCGTTATTTATATTGT<br>TAATTAGCAAACACAAAACCCGC
KY039409	DBKY039409_Philippines_Hap_D1	ATTTTATTTTTTAACCTAACTCCCCTACTAAGTGTACCCCCCCTTTCCCCCCCAGGGGGGGTATACTATGCATAATCGTGCATACATTTATATACCACATATATTATGGT<br>ACCGGTAATATATACTATATATGTACTAAACCCATTATATGTATACGGGCATTAATCTATATTCCACATTTCTCCCAATGTCCATTCTATGCATGATCCAGGACATACTC<br>ATTCACCCTCCCCATAGACAGCTCCAAACCACTACCAAGTCACCTAACTATGAATGGTTGCAGGACATAAATCTCACTCTTATGCTCCTCCCCCAACAAGTCACCTAAC<br>TATGAATGGTTACAGGACATACATTTAACTACC---<br>ATGTTCTAACCCATTTGGTTATGCTCGCCGTATCAGATGGATTTATTGATCGTCCACCTCACGAGAGATCAGCAACCCCTGCCTGTAATGTACTTCATGACCAGTCTCA<br>GGCCCATTCTTTCCCCCTACACCCCTCGCCCTACTTGCCTTCCACCGTACCTCTGGTTCCTCGGTCAGGCACATCCCATGCATAACTCCTGAACTTTCTCACTTTTCAC<br>GAAGTCATCTGTGGATTATCTTCCCCTCTTTAGTCCGTGATCGCGGCATCTTCTCTCTTCTATTGCTGTTGGTTCCTTCTCTTTTTGGGGCTTCTTCACAGGTTGCCCTT<br>CACAGTGCGGGTGCGGAGTGCTATTCAAGTGAAGCCTGGACTACACCTGCGTTGCGTCCTATCCTAGTCCTCTCGTGTCCCTCGATGAGACGGTTTGCGTGTATGGGG<br>AATCATCTTGACACTGATGCACTTTGGATCGCATTTGGTTATGGTTCTTCCACCCNCCCC-<br>GGTAAATGGTGCTATTTAGTGAATGCTTGTCGGACATATTTTTATCAATTTTCACTTCCTCTATTTTCTTCACAAAACTAGGAAATTCACCACAATTTTTTCTTTGTTATT<br>TTTTAATTTTTTTTTTATTTTTTAAAAACATTTTTTAAAAAACTAAATTACATACAAACTACCGCATAAAATCCCTCAAACTATACAAACGTTTATCGTATAATATATATAC<br>ATTATTGTTTATTCTATCATTATTAGAGAAACTCCACTACCAAAACCATCATTAAAACAAAAATTTACATGCCACTTAACTCCCCTCACAAACAATCGTTATTTATATTGT<br>TAATTAGCAAACACAAAACCCGC

KY039410	DBKY039410_Philippines_Hap_D1a	ATTTTATTTTTTAACCTAACTCCCCTACTAAGTGTACCCCCCCTTTCCCCCCCAGGGGGGGTATACTATGCATAATCGTGCATACATTTATATACCACATATATTATGGT<br>ACCGGTAATATATACTATATATGTACTAAACCCATTATATGTATACGGGCATTAATCTATATTCCACATTTCTCCCAATGTCCATTCTATGCATGATCCAGGACATACTC<br>ATTCACCCTCCCCATAGACAGCTCCAAACCACTACCAAGTCACCTAACTATGAATGGTTGCAGGACATAAATCTTACTCTCATGCTCTTCCCCCAACAAGTCACCTAAC<br>TATGAATGGTTGCAGGACATACATTTAACTACC--<br>ATGTTCTAACCCATTTGGTTATGCTCGCCGTATCAGATGGATTTATTGATCGTCCACCTCACGAGAGATCAGCAACCCCTGCCTGTAATGTACTTCATGACCAGTCTCA<br>GGCCCATTCTTTCCCCCTACACCCCTCGCCCTACTTGCCTTCCACCGTACCTCTGGTTCCTCGGTCAGGCACATCCCATGCATAACTCCTGAACTTTCTCACTTTTCAC<br>GAAGTCATCTGTGGATTATCTTCCCCTCTTTAGTCCGTGATCGCGGCATCTTCTCTCTTCTATTGCTGTTGGTTCCTTCTCTTTTTGGGGCTTCTTCACAGGTTACCCTT<br>CACAGTGCGGGTGCGGAGTGCTATTCAAGTGAAGCCTGGACTACACCTGCGTTGCGTCCTATCCTAGTCCTCTCGTGTCCCTCGATGAGACGGTTTGCGTGTATGGGG<br>AATCATCTTGACACTGATGCACTTTGGATCGCATTTGGTTATGGTTCTTCCANCCCCCC-<br>GGTAAATGGTGCTATTTAGTGAATGCTTGTCGGACATATTTTTATCAATTTTCACTTCCTCTATTTTCTTCACAAAACTAGGAAATTCACCACAATTTTTTCTTTGTTATT<br>TTTTAATTTTTTTTTTATTTTTTAAAAACATTTTTTAAAAAACTAAATTACATACAAACTACCGCATAAAATCCCTCAAACTATACAAACGTTTATCGTATAATATATATAC<br>ATTATTGTTTATTCTATCATTATTAGAGAAACTCCACTACCAAAACCATCATTAAAACAAAAATTTACATGCCACTTAACTCCCCTCACAAACAATCGTTATTTATATTGT<br>TAATTAGCAAACACAAAACCCGC
KY039411	DBKY039411_Philippines_Hap_D1a	ATTTTATTTTTTAACCTAACTCCCCTACTAAGTGTACCCCCCCTTTCCCCCCCAGGGGGGGTATACTATGCATAATCGTGCATACATTTATATACCACATATATTATGGT<br>ACCGGTAATATATACTATATATGTACTAAACCCATTATATGTATACGGGCATTAATCTATATTCCACATTTCTCCCAATGTCCATTCTATGCATGATCCAGGACATACTC<br>ATTCACCCTCCCCATAGACAGTTCCAAACCACTACCAAGTCACCTAACTATGAATGGTTGCAGGACATAAATCTCACTCTCATGCTCTTCCCCCAACAAGTCACCTAAC<br>TATGAATGGTTGCAGGACATACATTTAACTACC--<br>ATGTTCTAACCCATTTGGTTATGCTCGCCGTATCAGATGGATTTATTGATCGTCCACCTCACGAGAGATCAGCAACCCCTGCCTGTAATGTACTTCATGACCAGTCTCA<br>GGCCCATTCTTTCCCCCTACACCCCTCGCCCTACTTGCCTTCCACCGTACCTCTGGTTCCTCGGTCAGGCACATCCCATGCATAACTCCTGAACTTTCTCACTTTTCAC<br>GAAGTCATCTGTGGATTATCTTCCCCTCTTTAGTCCGTGATCGCGGCATCTTCTCTCTTCTATTGCTGTTGGTTCCTTCTCTTTTTGGGGCTTCTTCACAGGTTGCCCTT<br>CACAGTGCGGGTGCGGAGTGCTATTCAAGTGAAGCCTGGACTACACCTGCGTTGCGTCCTATCCTAGTCCTCTCGTGTCCCTCGATGAGACGGTTTGCGTGTATGGGG<br>AATCATCTTGACACTGATGCACTTTGGATCGCATTTGGTTATGGTTCTTCCACCCCCCCC-<br>GGTAAATGGTGCTATTTAGTGAATGCTTGTCGGACATATTTTTATCAATTTTCACTTCCTCTATTTTCTTCACAAAACTAGGAAATTCACCACAATTTTTTCTTTGTTATT<br>TTTTAATTTTTTTTTTATTTTTTAAAAACATTTTTTAAAAAACTAAATTACATACAAACTACCGCATAAAATCCCTCAAACTATACAAACGTTTATCGTATAATATATATAC<br>ATTATTGTTTATTCTATCATTATTAGAGAAACTCCACTACCAAAACCATCATTAAAACAAAAATTTACATGCCACTTAACTCCCCTCACAAACAATCGTTATTTATATTGT<br>TAATTAGCAAACACAAAACCCGC
KY039412	DBKY039412_Philippines_Hap_D1a	ATTTTATTTTTTAACCTAACTCCCCTACTAAGTGTACCCCCCCTTTCCCCCCCAGGGGGGGTATACTATGCATAATCGTGCATACATTTATATACCACATATATTATGGT<br>ACCGGTAATATATACTATATATGTACTAAACCCATTATATGTATACGGGCATTAATCTATATTCCACATTTCTCCCAATGTCCATTCTATGCATGATCCAGGACATACTC<br>ATTCACCCTCCCCATAGACAGTTCCAAACCACTACCAAGTCACCTAACTATGAATGGTTGCAGGACATAAATCTCACTCTCATGCTCTTCCCCCAACAAGTCACCTAAC<br>TATGAATGGTTGCAGGACATACATTTAACTACC--<br>ATGTTCTAACCCATTTGGTTATGCTCGCCGTATCAGATGGATTTATTGATCGTCCACCTCACGAGAGATCAGCAACCCCTGCCTGTAATGTACTTCATGACCAGTCTCA<br>GGCCCATTCTTTCCCCCTACACCCCTCGCCCTACTTGCCTTCCACCGTACCTCTGGTTCCTCGGTCAGGCACATCCCATGCATAACTCCTGAACTTTCTCACTTTTCAC<br>GAAGTCATCTGTGGATTATCTTCCCCTCTTTAGTCCGTGATCGCGGCATCTTCTCTCTTCTATTGCTGTTGGTTCCTTCTCTTTTTGGGGCTTCTTCACAGGTTGCCCTT<br>CACAGTGCGGGTGCGGAGTGCTATTCAAGTGAAGCCTGGACTACACCTGCGTTGCGTCCTATCCTAGTCCTCTCGTGTCCCTCGATGAGACGGTTTGCGTGTATGGGG<br>AATCATCTTGACACTGATGCACTTTGGATCGCATTTGGTTATGGTTCTTCCACCCCCCCC-<br>GGTAAATGGTGCTATTTAGTGAATGCTTGTCGGACATATTTTTATCAATTTTCACTTCCTCTATTTTCTTCACAAAACTAGGAAATTCACCACAATTTTTTCTTTGTTATT<br>TTTTAATTTTTTTTTTATTTTTTAAAAACATTTTTTAAAAAACTAAATTACATACAAACTACCGCATAAAATCCCTCAAACTATACAAACGTTTATCGTATAATATATATAC<br>ATTATTGTTTATTCTATCATTATTAGAGAAACTCCACTACCAAAACCATCATTAAAACAAAAATTTACATGCCACTTAACTCCCCTCACAAACAATCGTTATTTATATTGT<br>TAATTAGCAAACACAAAACCCGC

KY039413	DBKY039413_Philippines_Hap_D1a	ATTTTATTTTTTAACCTAACTCCCCTACTAAGTGTACCCCCCCTTTCCCCCCCAGGGGGGGTATACTATGCATAATCGTGCATACATTTATATACCACATATATTATGGT<br>ACCGGTAATATATACTATATATGTACTAAACCCATTATATGTATACGGGCATTAATCTATATTCCACATTTCTCCCAATGTCCATTCTATGCATGATCCAGGACATACTC<br>ATTCACCCTCCCCATAGACAGCTCTAAACCACTACCAAGTCACCTAACTATGAATGGTTGCAGGACATAAATCTTACTCTCATGCTCTTCCCCCAACAAGTCACCTAAC<br>TATGAATGGTTGCAGGACATACATTTAACTACC---<br>ATGTTCTAACCCATTTGGTTATGCTCGCCGTATCAGATGGATTTATTGATCGTCCACCTCACGAGAGATCAGCAACCCCTGCCTGTAATGTACTTCATGACCAGTCTCA<br>GGCCCATTCTTTCCCCCTACACCCCTCGCCCTACTTGCCTTCCACCGTACCTCTGGTTCCTCGGTCAGGCACATCCCATGCATAACTCCTGAACTTTCTCACTTTTCAC<br>GAAGTCATCTGTGGATTATCTTCCCCTCTTTAGTCCGTGATCGCGGCATCTTCTCTCTTCTATTGCTGTTGGTTCCTTCTCTTTTTGGGGCTTCTTCACAGGTTACCCTT<br>CACAGTGCGGGTGCGGAGTGCTATTCAAGTGAAGCCTGGACTACACCTGCGTTGCGTCCTATCCTAGTCCTCTCGTGTCCCTCGATGAGACGGTTTGCGTGTATGGGG<br>AATCATCTTGACACTGATGCACTTTGGATCGCATTTGGTTATGGTTCTTCCACCCCCCCC-<br>GGTAAATGGTGCTATTTAGTGAATGCTTGTCGGACATATTTTTATCAATTTTCACTTCCTCTATTTTCTTCACAAAACTAGGAAATTCACCACAATTTTTTCTTTGTTATT<br>TTTTAATTTTTTTTTTATTTTTTAAAAACATTTTTTAAAAAACTAAATTACATACAAACTACCGCATAAAATCCCTCAAACTATACAAACGTTTATCGTATAATATATATAC<br>ATTATTGTTTATTCTATCATTATTAGAGAAACTCCACTACCAAAACCATCATTAAAACAAAAATTTACATGCCACTTAACTCCCCTCACAAACAATCGTTATTTATATTGT<br>TAATTAGCAAACACAAAACCCGC
KY039414	DBKY039414_Philippines_Hap_D1	ATTTTATTTTTTAACCTAACTCCCCTACTAAGTGTACCCCCCCTTTCCCCCCCAGGGGGGGTATACTATGCATAATCGTGCATACATTTATATACCACATATATTATGGT<br>ACCGGTAATATATACTATATATGTACTAAACCCATTATATGTATACGGGCATTAATCTATATTCCACATTTCTCCCAATGTCCATTCTATGCATGATCCAGGACATACCC<br>ATTCACCCTCCCCATAGACAGCTCCAAACCACTACCAAGTCACCTAACTATGAATGGTTGCAGGACATAAATCTCACTCTCATGCTCTTCCCCCAACAAGTCACCTAAC<br>TATGAATGGTTACAGGACATACATTTAACTACC---<br>ATGTTCTAACCCATTTGGTTATGCTCGCCGTATCAGATGGATTTATTGATCGTCCACCTCACGAGAGATCAGCAACCCCTGCCTGTAATGTACTTCATGACCAGTCTCA<br>GGCCCATTCTTTCCCCCTACACCCCTCGCCCTACTTGCCTTCCACCGTACCTCTGGTTCCTCGGTCAGGCACATCCCATGCATAACTCCTGAACTTTCTCACTTTTCAC<br>GAAGTCATCTGTGGATTATCTTCCCCTCTTTAGTCCGTGATCGCGGCATCTTCTCTCTTCTATTGCTGTTGGTTCCTTCTCTTTTTGGGGCTTCTTCACAGGTTGCCCTT<br>CACAGTGCGGGTGCGGAGTGCTATTCAAGTGAAGCCTGGACTACACCTGCGTTGCGTCCTATCCTAGTCCTCTCGTGTCCCTCGATGAGACGGTTTGCGTGTATGGGG<br>AATCATCTTGACACTGATGCACTTTGGATCGCATTTGGTTATGGTTCTTCCANCCCCCCC-<br>GGTAAATGGTGCTATTTAGTGAATGCTTGTCGGACATATTTTTATCAATTTTCACTTCCTCTATTTTCTTCACAAAACTAGGAAATTCACCACAATTTTTTCTTTGTTATT<br>TTTTAATTTTTTTTTTATTTTTTAAAAACATTTTTTAAAAAACTAAATTACATACAAACTACCGCATAAAATCCCTCAAACTATACAAACGTTTATCGTATAATATATATAC<br>ATTATTGTTTATTCTATCATTATTAGAGAAACTCCACTACCAAAACCATCATTAAAACAAAAATTTACATGCCACTTAACTCCCCTCACAAACAATCGTTATTTATATTGT<br>TAATTAGCAAACACAAAACCCGC
KY039415	DBKY039415_Philippines_Hap_D1a	ATTTTATTTTTTAACCTAACTCCCCTACTAAGTGTACCCCCCCTTTCCCCCCCAGGGGGGGTATACTATGCATAATCGTGCATACATTTATATACCACATATATTATGGT<br>ACCGGTAATATATACTATATATGTACTAAACCCATTATATGTATACGGGCATTAATCTATATTCCACATTTCTCCCAATGTCCATTCTATGCATGATCCAGGACATACTC<br>ATTCACCCTCCCCATAGACAGCTCCAAACCACTACCAAGTCACCTAACTATGAATGGTTGCAGGACATAAATCTTACTCTCATGCTCTTCCCCCAACAAGTCACCTAAC<br>TATGAATGGTTGCAGGACATACATTTAACTACC---<br>ATGTTCTAACCCATTTGGTTATGCTCGTCGTATCAGATGGATTTATTGATCGTCCACCTCACGAGAGATCAGCAACCCCTGCCTGTAATGTACTTCATGACCAGTCTCA<br>GGCCCATTCTTTCCCCCTACACCCCTCGCCCTACTTGCCTTCCACCGTACCTCTGGTTCCTCGGTCAGGCACATCCCATGCATAACTCCTGAACTTTCTCACTTTTCAC<br>GAAGTCATCTGTGGATTATCTTCCCCTCTTTAGTCCGTGATCGCGGCATCTTCTCTCTTCTATTGCTGTTGGTTCCTTCTCTTTTTGGGGCTTCTTCACAGGTTACCCTT<br>CACAGTGCGGGTGCGGAGTGCTATTCAAGTGAAGCCTGGACTACACCTGCGTTGCGTCCTATCCTAGTCCTCTCGTGTCCCTCGATGAGACGGTTTGCGTGTATGGGG<br>AATCATCTTGACACTGATGCACTTTGGATCGCATTTGGTTATGGTTCTTCCACCCCCCCC-<br>GGTAAATGGTGCTATTTAGTGAATGCTTGTCGGACATATTTTTATCAATTTTCACTTCCTCTATTTTCTTCACAAAACTAGGAAATTCACCACAATTTTTTCTTTGTTATT<br>TTTTAATTTTTTTTTTATTTTTTAAAAACATTTTTTAAAAAACTAAATTACATACAAACTACCGCATAAAATCCCTCAAACTATACAAACGTTTATCGTATAATATATATAC<br>ATTATTGTTTATTCTATCATTATTAGAGAAACTCCACTACCAAAACCATCATTAAAACAAAAATTTACATGCCACTTAACTCCCCTCACAAACAATCGTTATTTATATTGT<br>TAATTAGCAAACACAAAACCCGC

KY039416	DBKY039416_Philippines_Hap_D1a	ATTTTATTTTTTAACCTAACTCCCCTACTAAGTGTACCCCCCCTTTCCCCCCCAGGGGGGGTATACTATGCATAATCGTGCATACATTTATATACCACATATATTATGGT<br>ACCGGTAATATATACTATATATGTACTAAACCCATTATATGTATACGGGCATTAATCTATATTCCACATTTCTCCCAATGTCCATTCTATGCATGATCCAGGACATACTC<br>ATTCACCCTCCCCATAGACAGCTCCAAACCACTACCAAGTCACCTAACTATGAATGGTTGCAGGACATAAATCTTACTCTCATGCTCTTCCCCCAACAAGTCACCTAAC<br>TATGAATGGTTGCAGGACATACATTTAACTACC---<br>ATGTTCTAACCCATTTGGTTATGCTCGCCGTATCAGATGGATTTATTGATCGTCCACCTCACGAGAGATCAGCAACCCCTGCCTGTAATGTACTTCATGACCAGTCTCA<br>GGCCCATTCTTTCCCCCTACACCCCTCGCCCTACTTGCCTTCCACCGTACCTCTGGTTCCTCGGTCAGGCACATCCCATGCATAACTCCTGAACTTTCTCACTTTTCAC<br>GAAGTCATCTGTGGATTATCTTCCCCTCTTTAGTCCGTGATCGCGGCATCTTCTCTCTTCTATTGCTGTTGGTTCCTTCTCTTTTTGGGGCTTCTTCACAGGTTACCCTT<br>CACAGTGCGGGTGCGGAGTGCTATTCAAGTGAAGCCTGGACTACACCTGCGTTGCGTCCTATCCTAGTCCTCTCGTGTCCCTCGATGAGACGGTTTGCGTGTATGGGG<br>AATCATCTTGACACTGATGCACTTTGGATCGCATTTGGTTATGGTTCTTCCACCCCCCCC-<br>GGTAAATGGTGCTATTTAGTGAATGCTTGTCGGACATATTTTTATCAATTTTCACTTCCTCTATTTTCTTCACAAAACTAGGAAATTCACCACAATTTTTTCTTTGTTATT<br>TTTTAATTTTTTTTTTTATTTTTTAAAAACATTTTTTAAAAAACTAAATTACATACAAACTACCGCATAAAATCCCTCAAACTATACAAACGTTTATCGTATAATATATATAC<br>ATTATTGTTTATTCTATCATTATTAGAGAAACTCCACTACCAAAACCATCATTAAAACAAAAATTTACATGCCACTTAACTCCCCTCACAAACAATCGTTATTTATATTGT<br>TAATTAGCAAACACAAAACCCGC
KY039417	DBKY039417_Philippines_Hap_D1a	ATTTTATTTTTTAACCTAACTCCCCTACTAAGTGTACCCCCCCTTTCCCCCCCAGGGGGGGTATACTATGCATAATCGTGCATACATTTATATACCACATATATTATGGT<br>ACCGGTAATATATACTATATATGTACTAAACCCATTATATGTATACGGGCATTAATCTATATTCCACATTTCTCCCAATGTCCATTCTATGCATGATCCAGGACATACTC<br>ATTCACCCTCCCCATAGACAGCTCCAAACCACTACCAAGTCACCTAACTATGAATGGTTGCAGGACATAAATCTCACTCTCATGCTCCTCCCCCAACAAGTCACCTAAC<br>TATGAATGGTTGCAGGACATACATTCAACTACC---<br>ATGTTCTAACCCATTTGGTTATGCTCGCCGTATCAGATGGATTTATTGATCGTCCACCTCACGAGAGATCAGCAACCCCTGCCTGTAATGTACTTCATGACCAGTCTCA<br>GGCCCATTCTTTCCCCCTACACCCCTCGCCCTACTTGCCTTCCACCGTACCTCTGGTTCCTCGGTCAGGCACATCCCATGCATAACTCCTGAACTTTCTCACTTTTCAC<br>GAAGTCATCTGTGGATTATCTTCCCCTCTTTAGTCCGTGATCGCGGCATCTTCTCTCTTCTATTGCTGTTGGTTCCTTCTCTTTTTGGGGCTTCTTCACAGGTTGCCCTT<br>CACAGTGCGGGTGCGGAGTGCTATTCAAGTGAAGCCTGGACTACACCTGCGTTGCGTCCTATCCTAGTCCTCTCGTGTCCCTCGATGAGACGGTTTGCGTGTATGGGG<br>AATCATCTTGACACTGATGCACTTTGGATCGCATTTGGTTATGGTTCTTCCACCCNCCCC-<br>GGTAAATGGTGCTATTTAGTGAATGCTTGTCGGACATATTTTTATCAATTTTCACTTCCTCTATTTTCTTCACAAAACTAGGAAATTCACCACAATTTTTTCTTTGTTATT<br>TTTTAATTTTTTTTTTTATTTTTTAAAAACATTTTTTAAAAAACTAAATTACATACAAACTACCGCATAAAATCCCTCAAACTATACAAACGTTTATCGTATAATATATATAC<br>ATTATTGTTTATTCTATCATTATTAGAGAAACTCCACTACCAAAACCATCATTAAAACAAAAATTTACATGCCACTTAACTCCCCTCACAAACAATCGTTATTTATATTGT<br>TAATTAGCAAACACAAAACCCGC
KY039418	DBKY039418_Indonesia_Hap_D1	ATTTTATTTTTTAACCTAACTCCCCTACTAAGTGTACCCCCCCTTTCCCCCCCAGGGGGGGTATACTATGCATAATCGTGCATACATTTATATACCACATATATTATGGT<br>ACCGGTAATATATACTATATATGTACTAAACCCATTATATGTATACGGGCATTAATCTATATTCCACATTTCTCCCAATGTCCATTCTATGCATGATCCAGGACATACTC<br>ATTCACCCTCCCCATAGACAGCTCCAAACCACTACCAAGTCACCTAACTATGAATGGTTGCAGGACATAAATCTCACTCTTATGCTCCTCCCCCAACAAGTCACCTAAC<br>TATGAATGGTTACAGGACATACATTTAACTACC---<br>ATGTTCTAACCCATTTGGTTATGCTCGCCGTATCAGATGGATTTATTGATCGTCCACCTCACGAGAGATCAGCAACCCCTGCCTGTAATGTACTTCATGACCAGTCTCA<br>GGCCCATTCTTTCCCCCTACACCCCTCGCCCTACTTGCCTTCCACCGTACCTCTGGTTCCTCGGTCAGGCACATCCCATGCATAACTCCTGAACTTTCTCACTTTTCAC<br>GAAGTCATCTGTGGATTATCTTCCCCTCTTTAGTCCGTGATCGCGGCATCTTCTCTCTTCTATTGCTGTTGGTTCCTTCTCTTTTTGGGGCTTCTTCACAGGTTGCCCTT<br>CACAGTGCGGGTGCGGAGTGCTATTCAAGTGAAGCCTGGACTACACCTGCGTTGCGTCCTATCCTAGTCCTCTCGTGTCCCTCGATGAGACGGTTTGCGTGTATGGGG<br>AATCATCTTGACACTGATGCACTTTGGATCGCATTTGGTTATGGTTCTTCCANCCCCCCC-<br>GGTAAATGGTGCTATTTAGTGAATGCTTGTCGGACATATTTTTATCAATTTTCACTTCCTCTATTTTCTTCACAAAACTAGGAAATTCACCACAATTTTTTCTTTGTTATT<br>TTTTAATTTTTTTTTTTATTTTTTAAAAACATTTTTTAAAAAACTAAATTACATACAAACTACCGCATAAAATCCCTCAAACTATACAAACGTTTATCGTATAATATATATAC<br>ATTATTGTTTATTCTATCATTATTAGAGAAACTCCACTACCAAAACCATCATTAAAACAAAAATTTACATGCCACTTAACTCCCCTCACAAACAATCGTTATTTATATTGT<br>TAATTAGCAAACACAAAACCCGC

KY039419	DBKY039419_Indonesia_Hap_D1	ATTTTATTTTTTAACCTAACTCCCCTACTAAGTGTACCCCCCCTTTCCCCCCCAGGGGGGGTATACTATGCATAATCGTGCATACATTTATATACCACATATATTATGGT<br>ACCGGTAATATATACTATATATGTACTAAACCCATTATATGTATACGGGCATTAATCTATATTCCACATTTCTCCCAATGTCCATTCTATGCATGATCCAGGACATACTC<br>ATTCACCCTCCCCATAGACAGCTCCAAACCACTACCAAGTCACCTAACTATGAATGGTTGCAGGACATAAATCTTACTCTCATGCTCTTCCCCCAACAAGTCACCTAAC<br>TATGAATGGTTACAGGACATACATTTAACTACC--<br>ATGTTCTAACCCATTTGGTTATGCTCGTCGTATCAGATGGATTTATTGATCGTCCACCTCACGAGAGATCAGCAACCCCTGCCTGTAATGTACTTCATGACCAGTCTCA<br>GGCCCATTCTTTCCCCCTACACCCCTCGCCCTACTTGCCTTCCACCGTACCTCTGGTTCCTCGGTCAGGCACATCCCATGCATAACTCCTGAACTTTCTCACTTTTCAC<br>GAAGTCATCTGTGGATTATCTTCCCCTCTTTAGTCCGTGATCGCGGCATCTTCTCTCTTCTATTGCTGTTGGTTCCTTCTCTTTTTGGGGCTTCTTCACAGGTTGCCCTT<br>CACAGTGCGGGTGCGGAGTGCTATTCAAGTGAAGCCTGGACTACACCTGCGTTGCGTCCTATCCTAGTCCTCTCGTGTCCCTCGATGAGACGGTTTGCGTGTATGGGG<br>AATCATCTTGACACTGATGCACTTTGGATCGCATTTGGTTATGGTTCTTCCACCCCCCCC-<br>GGTAAATGGTGCTATTTAGTGAATGCTTGTCGGACATATTTTTATCAATTTTCACTTCCTCTATTTTCTTCACAAAACTAGGAAATTCACCACAATTTTTTCTTTGTTATT<br>TTTTAATTTTTTTTTTATTTTTTAAAAACATTTTTTAAAAAACTAAATTACATACAAACTACCGCATAAAATCCCTCAAACTATACAAACGTTTATCGTATAATATATATAC<br>ATTATTGTTTATTCTATCATTATTAGAGAAACTCCACTACCAAAACCATCATTAAAACAAAAATTTACATGCCACTTAACTCCCCTCACAAACAATCGTTATTTATATTGT<br>TAATTAGCAAACACAAAACCCGC
KY039420	DBKY039420_Indonesia_Hap_D1	ATTTTATTTTTTAACCTAACTCCCCTACTAAGTGTACCCCCCCTTTCCCCCCCAGGGGGGGTATACTATGCATAATCGTGCATACATTTATATACCACATATATTATGGT<br>ACCGGTAATATATACTATATATGTACTAAACCCATTATATGTATACGGGCATTAATCTATATTCCACATTTCTCCCAATGTCCATTCTATGCATGATCCAGGACATACTC<br>ATTCACCCTCCCCATAGACAGCTCCAAACCACTACCAAGTCACCTAACCATGAATGGTTGCAGGACATAAATCTCACTCTCATGCTCCTCCCCCAACAAGTCACCTAA<br>CTATGAATGGTTACAGGACATACATTTAACTACC--<br>ATGTTCTAACCCATTTGGTTATGCTCGCCGTATCAGATGGATTTATTGATCGTCCACCTCACGAGAGATCAGCAACCCCTGCCTGTAATGTACTTCATGACCAGTCTCA<br>GGCCCATTCTTTCCCCCTACACCCCTCGCCCTACTTGCCTTCCACCGTACCTCTGGTTCCTCGGTCAGGCACATCCCATGCATAACTCCTGAACTTTCTCACTTTTCAC<br>GAAGTCATCTGTGGATTATCTTCCCCTCTTTAGTCCGTGATCGCGGCATCTTCTCTCTTCTATTGCTGTTGGTTCCTTCTCTTTTTGGGGCTTCTTCACAGGTTGCCCTT<br>CACAGTGCGGGTGCGGAGTGCTATTCAAGTGAAGCCTGGACTACACCTGCGTTGCGTCCTATCCTAGTCCTCTCGTGTCCCTCGATGAGACGGTTTGCGTGTATGGGG<br>AATCATCTTGACACTGATGCACTTTGGATCGCATTTGGTTATGGTTCTTCCACCCNCCCC-<br>GGTAAATGGTGCTATTTAGTGAATGCTTGTCGGACATATTTTTATCAATTTTCACTTCCTCTATTTTCTTCACAAAACTAGGAAATTCACCACAATTTTTTCTTTGTTATT<br>TTTTAATTTTTTTTTTATTTTTTAAAAACATTTTTTAAAAAACTAAATTACATACAAACTACCGCATAAAATCCCTCAAACTATACAAACGTTTATCGTATAATATATATAC<br>ATTATTGTTTATTCTATCATTATTAGAGAAACTCCACTACCAAAACCATCATTAAAACAAAAATTTACATGCCACTTAACTCCCCTCACAAACAATCGTTATTTATATTGT<br>TAATTAGCAAACACAAAACCCGC
KY039421	DBKY039421_Indonesia_Hap_D1	ATTTTATTTTTTAACCTAACTCCCCTACTAAGTGTACCCCCCCTTTCCCCCCCAGGGGGGGTATACTATGCATAATCGTGCATACATTTATATACCACATATATTATGGT<br>ACCGGTAATATATACTATATATGTACTAAACCCATTATATGTATACGGGCATTAATCTATATTCCACATTTCTCCCAATGTCCATTCTATGCATGATCCAGGACATACCC<br>ATTCACCCTCCCCATAGACAGCTCCAAACCACTACCAAGTCACCTAACTATGAATGGTTGCAGGACATAAATCTNACTCTCATGCTCNTCCCCCAACAAGTCACCTAAC<br>TATGAATGGTTGCAGGACATACATTTAACTACC--<br>ATGTTCTAACCCATTTGGTTATGCTCGTCGTATCAGATGGATTTATTGATCGTCCACCTCACGAGAGATCAGCAACCCCTGCCTGTAATGTACTTCATGACCAGTCTCA<br>GGCCCATTCTTTCCCCCTACACCCCTCGCCCTACTTGCCTTCCACCGTACCTCTGGTTCCTCGGTCAGGCACATCCCATGCATAACTCCTGAACTTTCTCACTTTTCAC<br>GAAGTCATCTGTGGATTATCTTCCCCTCTTTAGTCCGTGATCGCGGCATCTTCTCTCTTCTATTGCTGTTGGTTCCTTCTCTTTTTGGGGCTTCTTCACAGGTTGCCCTT<br>CACAGTGCGGGTGCGGAGTGCTATTCAAGTGAAGCCTGGACTACACCTGCGTTGCGTCCTATCCTAGTCCTCTCGTGTCCCTCGATGAGACGGTTTGCGTGTATGGGG<br>AATCATCTTGACACTGATGCACTTTGGATCGCATTTGGTTATGGTTCTTCCACCCNCCCC-<br>GGTAAATGGTGCTATTTAGTGAATGCTTGTCGGACATATTTTTATCAATTTTCACTTCCTCTATTTTCTTCACAAAACTAGGAAATTCACCACAATTTTTTCTTTGTTATT<br>TTTTAATTTTTTTTTTATTTTTTAAAAACATTTTTTAAAAAACTAAATTACATACAAACTACCGCATAAAATCCCTCAAACTATACAAACGTTTATCGTATAATATATATAC<br>ATTATTGTTTATTCTATCATTATTAGAGAAACTCCACTACCAAAACCATCATTAAAACAAAAATTTACATGCCACTTAACTCCCCTCACAAACAATCGTTATTTATATTGT<br>TAATTAGCAAACACAAAACCCGC

KY039422	DBKY039422_Indonesia_Hap_D1a	ATTTTATTTTTTAACCTAACTCCCCTACTAAGTGTACCCCCCCTTTCCCCCCCAGGGGGGGTATACTATGCATAATCGTGCATACATTTATATACCACATATATTATGGT<br>ACCGGTAATATATACTATATATGTACTAAACCCATTATATGTATACGGGCATTAATCTATATTCCACATTTCTCCCAATGTCCATTCTATGCATGATCCAGGACATACTC<br>ATTCACCCTCCCCATAGACAGCTCCAAACCACTACCAAGTCACCTAACTATGAATGGTTGCAGGACATAAATCTCACTCTCATGCTCTTCCCCCAACAAGTCACCTAAC<br>TATGAATGGTTGCAGGACATACATTTAACTACC---<br>ATGTTCTAACCCATTTGGTTATGCTCGCCGTATCAGATGGATTTATTGATCGTCCACCTCACGAGAGATCAGCAACCCCTGCCTGTAATGTACTTCATGACCAGTCTCA<br>GGCCCATTCTTTCCCCCTACACCCCTCGCCCTACTTGCCTTCCACCGTACCTCTGGTTCCTCGGTCAGGCACATCCCATGCATAACTCCTGAACTTTCTCACTTTTCAC<br>GAAGTCATCTGTGGATTATCTTCCCCTCTTTAGTCCGTGATCGCGGCATCTTCTCTCTTCTATTGCTGTTGGTTCCTTCTCTTTTTGGGGCTTCTTCACAGGTTGCCCTT<br>CACAGTGCGGGTGCGGAGTGCTATTCAAGTGAAGCCTGGACTACACCTGCGTTGCGTCCTATCCTAGTCCTCTCGTGTCCCTCGATGAGACGGTTTGCGTGTATGGGG<br>AATCATCTTGACACTGATGCACTTTGGATCGCATTTGGTTATGGTTCTTCCACCCNCCCC-<br>GGTAAATGGTGCTATTTAGTGAATGCTTGTCGGACATATTTTTATCAATTTTCACTTCCTCTATTTTCTTCACAAAACTAGGAAATTCACCACAATTTTTTCTTTGTTATT<br>TTTTAATTTTTTTTTTATTTTTTAAAAACATTTTTTAAAAAACTAAATTACATACAAACTACCGCATAAAATCCCTCAAACTATACAAACGTTTATCGTATAATATATATAC<br>ATTATTGTNTATTCTATCATTATTAGAGAAACTCCACTACCAAAACCATCATTAAAACAAAAATTTACATGCCACTTAACTCCCCTCACAAACAATCGTTATTTATATTGT<br>TAATTAGCAAACACAAAACCCGC
KY039423	DBKY039423_Indonesia_Hap_D1	ATTTTATTTTTTAACCTAACTCCCCTACTAAGTGTACCCCCCCTTTCCCCCCCAGGGGGGGTATACTATGCATAATCGTGCATACATTTATATACCACATATATTATGGT<br>ACCGGTAATATATACTATATATGTACTAAACCCATTATATGTATACGGGCATTAATCTATATTCCACATTTCTCCCAATGTCCATTCTATGCATGATCCAGGACATACTC<br>ATTCACCCTCCCCATAGACAGCTCCAAACCACTACCAAGTCACCTAACTATGAATGGTTGCAGGACATAAATCTTACTCTCATGCTCTTCCCCCAACAAGTCACCTAAC<br>TATGAATGGTTACAGGACATACATTTAACTACC---<br>ATGTTCTAACCCATTTGGTTATGCTCGTCGTATCAGATGGATTTATTGATCGTCCACCTCACGAGAGATCAGCAACCCCTGCCTGTAATGTACTTCATGACCAGTCTCA<br>GGCCCATTCTTTCCCCCTACACCCCTCGCCCTACTTGCCTTCCACCGTACCTCTGGTTCCTCGGTCAGGCACATCCCATGCATAACTCCTGAACTTTCTCACTTTTCAC<br>GAAGTCATCTGTGGATTATCTTCCCCTCTTTAGTCCGTGATCGCGGCATCTTCTCTCTTCTATTGCTGTTGGTTCCTTCTCTTTTTGGGGCTTCTTCACAGGTTGCCCTT<br>CACAGTGCGGGTGCGGAGTGCTATTCAAGTGAAGCCTGGACTACACCTGCGTTGCGTCCTATCCTAGTCCTCTCGTGTCCCTCGATGAGACGGTTTGCGTGTATGGGG<br>AATCATCTTGACACTGATGCACTTTGGATCGCATTTGGTTATGGTTCTTCCACCCCCCCC-<br>GGTAAATGGTGCTATTTAGTGAATGCTTGTCGGACATATTTTTATCAATTTTCACTTCCTCTATTTTCTTCACAAAACTAGGAAATTCACCACAATTTTTTCTTTGTTATT<br>TTTTAATTTTTTTTTTATTTTTTAAAAACATTTTTTAAAAAACTAAATTACATACAAACTACCGCATAAAATCCCTCAAACTATACAAACGTTTATCGTATAATATATATAC<br>ATTATTGTTTATTCTATCATTATTAGAGAAACTCCACTACCAAAACCATCATTAAAACAAAAATTTACATGCCACTTAACTCCCCTCACAAACAATCGTTATTTATATTGT<br>TAATTAGCAAACACAAAACCCGC
KY039424	DBKY039424_Indonesia_Hap_D1	ATTTTATTTTTTAACCTAACTCCCCTACTAAGTGTACCCCCCCTTTCCCCCCCAGGGGGGGTATACTATGCATAATCGTGCATACATTTATATACCACATATATTATGGT<br>ACCGGTAATATATACTATATATGTACTAAACCCATTATATGTATACGGGCATTAATCTATATTCCACATTTCTCCCAATGTCCATTCTATGCATGATCCAGGACATACTC<br>ATTCACCCTCCCCATAGACAGCTCCAAACCACTACCAAGTCACCTAACTATGAATGGTTGCAGGACATAAATCTCACTCTCATGCTCTTCCCCCAACAAGTCACCTAAC<br>TATGAATGGTTACAGGACATACATTTAACTACC---<br>ATGTTCTAACCCATTTGGTTATGCTCGCCGTATCAGATGGATTTATTGATCGTCCACCTCACGAGAGATCAGCAACCCCTGCCTGTAATGTACTTCATGACCAGTCTCA<br>GGCCCATTCTTTCCCCCTACACCCCTCGCCCTACTTGCCTTCCACCGTACCTCTGGTTCCTCGGTCAGGCACATCCCATGCATAACTCCTGAACTTTCTCACTTTTCAC<br>GAAGTCATCTGTGGATTATCTTCCCCTCTTTAGTCCGTGATCGCGGCATCTTCTCTCTTCTATTGCTGTTGGTTCCTTCTCTTTTTGGGGCTTCTTCACAGGTTGCCCTT<br>CACAGTGCGGGTGCGGAGTGCTATTCAAGTGAAGCCTGGACTACACCTGCGTTGCGTCCTATCCTAGTCCTCTCGTGTCCCTCGATGAGACGGTTTGCGTGTATGGGG<br>AATCATCTTGACACTGATGCACTTTGGATCGCATTTGGTTATGGTTCTTCCANCCCCCCC-<br>GGTAAATGGTGCTATTTAGTGAATGCTTGTCGGACATATTTTTATCAATTTTCACTTCCTCTATTTTCTTCACAAAACTAGGAAATTCACCACAATTTTTTCTTTGTTATT<br>TTTTAATTTTTTTTTTATTTTTTAAAAACATTTTTTAAAAAACTAAATTACATACAAACTACCGCATAAAATCCCTCAAACTATACAAACGTTTATCGTATAATATATATAC<br>ATTATTGTTTATTCTATCATTATTANAGAAACTCCACTACCAAAACCATCATTAAAACAAAAATTTACATGCCACTTAACTCCCCTCACAAACAATCGTTATTTATATTGT<br>TAATTAGCAAACACAAAACCCGC

KY039425	DBKY039425_Indonesia_Hap_D1a	ATTTTATTTTTTAACCTAACTCCCCTACTAAGTGTACCCCCCCTTTCCCCCCCAGGGGGGGTATACTATGCATAATCGTGCATACATTTATATACCACATATATTATGGT<br>ACCGGTAATATATACTATATATGTACTAAACCCATTATATGTATACGGGCATTAATCTATATTCCACATTTCTCCCAATGTCCATTCTATGCATGATCCAGGACATACTC<br>ATTCACCCTCCCCATAGACAGCTCCAAACCACTACCAAGTCACCTAACTATGAATGGTTGCAGGACATAAATCTCACTCTCATGCTCTTCCCCCAACAAGTCACCTAAC<br>TATGAATGGTTGCAGGACATACATTTAACTACC--<br>ATGTTCTAACCCATTTGGTTATGCTCGCCGTATCAGATGGATTTATTGATCGTCCACCTCACGAGAGATCAGCAACCCCTGCCTGTAATGTACTTCATGACCAGTCTCA<br>GGCCCATTCTTTCCCCCTACACCCCTCGCCCTACTTGCCTTCCACCGTACCTCTGGTTCCTCGGTCAGGCACATCCCATGCATAACTCCTGAACTTTCTCACTTTTCAC<br>GAAGTCATCTGTGGATTATCTTCCCCTCTTTAGTCCGTGATCGCGGCATCTTCTCTCTTCTATTGCTGTTGGTTCCTTCTCTTTTTGGGGCTTCTTCACAGGTTGCCCTT<br>CACAGTGCGGGTGCGGAGTGCTATTCAAGTGAAGCCTGGACTACACCTGCGTTGCGTCCTATCCTAGTCCTCTCGTGTCCCTCGATGAGACGGTTTGCGTGTATGGGG<br>AATCATCTTGACACTGATGCACTTTGGATCGCATTTGGTTATGGTTCTTCCANCCCCCCC-<br>GGTAAATGGTGCTATTTAGTGAATGCTTGTCGGACATATTTTTATCAATTTTCACTTCCTCTATTTTCTTCACAAAACTAGGAAATTCACCACAATTTTTTCTTTGTTATT<br>TTTTAATTTTTTTTTTATTTTTTAAAAACATTTTTTAAAAAACTAAATTACATACAAACTACCGCATAAAATCCCTCAAACTATACAAACGTTTATCGTATAATATATATAC<br>ATTATTGTTTATTCTATCATTATTAGAGAAACTCCACTACCAAAACCATCATTAAAACAAAAATTTACATGCCACTTAACTCCCCTCACAAACAATCGTTATTTATATTGT<br>TAATTAGCAAACACAAAACCCGC
KY039426	DBKY039426_Indonesia_Hap_D1a	ATTTTATTTTTTAACCTAACTCCCCTACTAAGTGTACCCCCCCTTTCCCCCCCAGGGGGGGTATACTATGCATAATCGTGCATACATTTATATACCACATATATTATGGT<br>ACCGGTAATATATACTATATATGTACTAAACCCATTATATGTATACGGGCATTAATCTATATTCCACATTTCTCCCAATGTCCATTCTATGCATGATCCAGGACATACTC<br>ATTCACCCTCCCCATAGACAGCTCCAAACCACTACCAAGTCACCTAACTATGAATGGTTGCAGGACATAAATCTCACTCTCATGCTCTTCCCCCAACAAGTCACCTAAC<br>TATGAATGGTTGCAGGACATACATTTAACTACC--<br>ATGTTCTAACCCATTTGGTTATGCTCGTCGTATCAGATGGATTTATTGATCGTCCACCTCACGAGAGATCAGCAACCCCTGCCTGTAATGTACTTCATGACCAGTCTCA<br>GGCCCATTCTTTCCCCCTACACCCCTCGCCCTACTTGCCTTCCACCGTACCTCTGGTTCCTCGGTCAGGCACATCCCATGCATAACTCCTGAACTTTCTCACTTTTCAC<br>GAAGTCATCTGTGGATTATCTTCCCCTCTTTAGTCCGTGATCGCGGCATCTTCTCTCTTCTATTGCTGTTGGTTCCTTCTCTTTTTGGGGCTTCTTCACAGGTTGCCCTT<br>CACAGTGCGGGTGCGGAGTGCTATTCAAGTGAAGCCTGGACTACACCTGCGTTGCGTCCTATCCTAGTCCTCTCGTGTCCCTCGATGAGACGGTTTGCGTGTATGGGG<br>AATCATCTTGACACTGATGCACTTTGGATCGCATTTGGTTATGGTTCTTCCACCCNCCCC-<br>GGTAAATGGTGCTATTTAGTGAATGCTTGTCGGACATATTTTTATCAATTTTCACTTCCTCTATTTTCTTCACAAAACTAGGAAATTCACCACAATTTTTTCTTTGTTATT<br>TTTTAATTTTTTTTTTATTTTTTAAAAACATTTTTTAAAAAACTAAATTACATACAAACTACCGCATAAAATCCCTCAAACTATACAAACGTTTATCGTATAATATATATAC<br>ATTATTGTTTATTCTATCATTATTAGAGAAACTCCACTACCAAAACCATCATTAAAACAAAAATTTACATGCCACTTAACTCCCCTCACAAACAATCGTTATTTATATTGT<br>TAATTAGCAAACACAAAACCCGC
KY039427	DBKY039427_Indonesia_Hap_D1	ATTTTATTTTTTAACCTAACTCCCCTACTAAGTGTACCCCCCCTTTCCCCCCCAGGGGGGGTATACTATGCATAATCGTGCATACATTTATATACCACATATATTATGGT<br>ACCGGTAATATATACTATATATGTACTAAACCCATTATATGTATACGGGCATTAATCTATATTCCACATTTCTCCCAATGTCCATTCTATGCATGATCCAGGACATACTC<br>ATTCACCCTCCCCATAGACAGCTCCAAACCACTACCAAGTCACCTAACTATGAATGGTTGCAGGACATAAATCTCACTCTCATGCTCTTCCTCCAACAAGTCACCTAAC<br>TATGAATGGTTACAGGACATACATTTAACTACC--<br>ATGTTCTAACCCATTTGGTTATGCTCGCCGTATCAGATGGATTTATTGATCGTCCACCTCACGAGAGATCAGCAACCCCTGCCTGTAATGTACTTCATGACCAGTCTCA<br>GGCCCATTCTTTCCCCCTACACCCCTCGCCCTACTTGCCTTCCACCGTACCTCTGGTTCCTCGGTCAGGCACATCCCATGCATAACTCCTGAACTTTCTCACTTTTCAC<br>GAAGTCATCTGTGGATTATCTTCCCCTCTTTAGTCCGTGATCGCGGCATCTTCTCTCTTCTATTGCTGTTGGTTCCTTCTCTTTTTGGGGCTTCTTCACAGGTTGCCCTT<br>CACAGTGCGGGTGCGGAGTGCTATTCAAGTGAAGCCTGGACTACACCTGCGTTGCGTCCTATCCTAGTCCTCTCGTGTCCCTCGATGAGACGGTTTGCGTGTATGGGG<br>AATCATCTTGACACTGATGCACTTTGGATCGCATTTGGTTATGGTTCTTCCACCCNCCCC-<br>GGTAAATGGTGCTATTTAGTGAATGCTTGTCGGACATATTTTTATCAATTTTCACTTCCTCTATTTTCTTCACAAAACTAGGAAATTCACCACAATTTTTTCTTTGTTATT<br>TTTTAATTTTTTTTTTATTTTTTAAAAACATTTTTTAAAAAACTAAATTACATACAAACTACCGCATAAAATCCCTCAAACTATACAAACGTTTATCGTATAATATATATAC<br>ATTATTGTTTATTCTATCATTATTAGAGAAACTCCACTACCAAAACCATCATTAAAACAAAAATTTACATGCCACTTAACTCCCCTCACAAACAATCGTTATTTATATTGT<br>TAATTAGCAAACACAAAACCCGC

KY039428	DBKY039428_Indonesia_Hap_D1	ATTTTATTTTTTAACCTAACTCCCCTACTAAGTGTACCCCCCCTTTCCCCCCCAGGGGGGGTATACTATGCATAATCGTGCATACATTTATATACCACATATATTATGGT<br>ACCGGTAATATATACTATATATGTACTAAACCCATTATATGTATACGGGCATTAATCTATATTCCACATTTCTCCCAATGTCCATTCTATGCATGATCCAGGACATACTC<br>ATTCACCCTCCCCATAGACAGCTCCAAACCACTACCAAGTCACCTAACTATGAATGGTTGCAGGACATAAATCTCACTCTCATGCTCTTCCCCCAACAAGTCACCTAAC<br>TATGAATGGTTACAGGACATACATTTAACTACC---<br>ATGTTCTAACCCATTTGGTTATGCTCGCCGTATCAGATGGATTTATTGATCGTCCACCTCACGAGAGATCAGCAACCCCTGCCTGTAATGTACTTCATGACCAGTCTCA<br>GGCCCATTCTTTCCCCCTACACCCCTCGCCCTACTTGCCTTCCACCGTACCTCTGGTTCCTCGGTCAGGCACATCCCATGCATAACTCCTGAACTTTCTCACTTTTCAC<br>GAAGTCATCTGTGGATTATCTTCCCCTCTTTAGTCCGTGATCGCGGCATCTTCTCTCTTCTATTGCTGTTGGTTCCTTCTCTTTTTGGGGCTTCTTCACAGGTTGCCCTT<br>CACAGTGCGGGTGCGGAGTGCTATTCAAGTGAAGCCTGGACTACACCTGCGTTGCGTCCTATCCTAGTCCTCTCGTGTCCCTCGATGAGACGGTTTGCGTGTATGGGG<br>AATCATCTTGACACTGATGCACTTTGGATCGCATTTGGTTATGGTTCTTCCACCCNCCCC-<br>GGTAAATGGTGCTATTTAGTGAATGCTTGTCGGACATATTTTTATCAATTTTCACTTCCTCTATTTTCTTCACAAAACTAGGAAATTCACCACAATTTTTTCTTTGTTATT<br>TTTTAATTTTTTTTTTATTTTTTAAAAACATTTTTTAAAAAACTAAATTACATACAAACTACCGCATAAAATCCCTCAAACTATACAAACGTTTATCGTATAATATATATAC<br>ATTATTGTTTATTCTATCATTATTAGAGAAACTCCACTACCAAAACCATCATTAAAACAAAAATTTACATGCCACTTAACTCCCCTCACAAACAATCGTTATTTATATTGT<br>TAATTAGCAAACACAAAACCCGC
KY039429	DBKY039429_Indonesia_Hap_D1	ATTTTATTTTTTAACCTAACTCCCCTACTAAGTGTACCCCCCCTTTCCCCCCC-<br>NGGGGGGTATACTATGCATAATCGTGCATACATTTATATACCACATATATTATGGTACCGGTAATATATACTATATATGTACTAAACCCATTATATGTATACGGGCATTA<br>ATCTATATTCCACATTTCTCCCAATGTCCATTCTATGCATGATCCAGGACATACTCATTCACCCTCCCCATAGACAGCTCCAAACCACTACCAAGTCACCTAACTATGA<br>ATGGTTGCAGGACATAAATCTCACTCTCATGCTCCTCCCCCAACAAGTCACCTAACTATGAATGGTTACAGGACATACATTTAACTACC---<br>--<br>ATGTTCTAACCCATTTGGTTATGCTCGCCGTATCAGATGGATTTATTGATCGTCCACCTCACGAGAGATCAGCAACCCCTGCCTGTAATGTACTTCATGACCAGTCTCA<br>GGCCCATTCTTTCCCCCTACACCCCTCGCCCTACTTGCCTTCCACCGTACCTCTGGTTCCTCGGTCAGGCACATCCCATGCATAACTCCTGAACTTTCTCACTTTTCAC<br>GAAGTCATCTGTGGATTATCTTCCCCTCTTTAGTCCGTGATCGCGGCATCTTCTCTCTTCTATTGCTGTTGGTTCCTTCTCTTTTTGGGGCTTCTTCACAGGTTGCCCTT<br>CACAGTGCGGGTGCGGAGTGCTATTCAAGTGAAGCCTGGACTACACCTGCGTTGCGTCCTATCCTAGTCCTCTCGTGTCCCTCGATGAGACGGTTTGCGTGTATGGGG<br>AATCATCTTGACACTGATGCACTTTGGATCGCATTTGGTTATGGTTCTTCCACCCNCCCC-<br>GGTAAATGGTGCTATTTAGTGAATGCTTGTCGGACATATTTTTATCAATTTTCACTTCCTCTATTTTCTTCACAAAACTAGGAAATTCACCACAATTTTTTCTTTGTTATT<br>TTTTAATTTTTTTTTTATTTTTTAAAAACATTTTTTAAAAAACTAAATTACATACAAACTACCGCATAAAATCCCTCAAACTATACAAACGTTTATCGTATAATATATATAC<br>ATTATTGTTTATTCTATCATTATTAGAGAAACTCCACTACCAAAACCATCATTAAAACAAAAATTTACATGCCACTTAACTCCCCTCACAAACAATCGTTATTTATATTGT<br>TAATTAGCAAACACAAAACCCGC
KY039430	DBKY039430_Vietnam_Hap_D3	ATTTTATTTTTTAACCTAACTCCCCTACTAAGTGTACCCCCCCTTTCCCCCCCAGGGGGGGTATACTATGCATAATCGTGCATACATTTATATACCACATATATTATGGT<br>ACCGGTAATATATACTATATATGTACTAAACCCATTATATGTATACGGGCATTAATCTATATTCCACATTTCTCCCAATGTCCATTCTATGCATGATCCAGGACATACTC<br>ATTCACCCTCCCCATAGACAGCTCCAAACCACTACCAAGTCACCTAACTATGAATGGTTGCAGGACATAAATCTCACTCTCATGCTCTTCCCCCAACAAGTCACCTAAC<br>TATGAATGGTTACAGGACATACATTTAACTACC---<br>ATGTTCTAACCCATTTGGTTATGCTCGACGTATCAGATGGATTTATTGATCGTCCACCTCACGAGAGATCAGCAACCCCTGCCTGTAATGTACTTCATGACCAGTCTCA<br>GGCCCATTCTTTCCCCCTACACCCCTCGCCCTACTTGCCTTCCACCGTACCTCTGGTTCCTCGGTCAGGCACATCCCATGCATAACTCCTGAACTTTCTCACTTTTCAC<br>GAAGTCATCTGTGGATTATCTTCCCCTCTTTAGTCCGTGATCGCGGCATCTTCTCTCTTCTATTGCTGTTGGTTCCTTCTCTTTTTGGGGCTTCTTCACAGGTTGCCCTT<br>CACAGTGCGGGTGCGGAGTGCTATTCAAGTGAAGCCTGGACTACACCTGCGTTGCGTCCTATCCTAGTCCTCTCGTGTCCCTCGATGAGACGGTTTGCGTGTATGGGG<br>AATCATCTTGACACTGATGCACTTTGGATCGCATTTGGTTATGGTTCTTCCACCCCCCCC-<br>GGTAAATGGTGCTATTTAGTGAATGCTTGTCGGACATATTTTTATCAATTTTCACTTCCTCTATTTTCTTCACAAAACTAGGAAATTCACCACAATTTTTTCTTTGTTATT<br>TTTTAATTTTTTTTTTATTTTTTAAAAACATTTTTTAAAAAACTAAATTACATACAAACTACCGCATAAAATCCCTCAAACTATACAAACGTTTATCGTATAATATATATAC<br>ATTATTGTTTATTCTATCATTATTAGAGAAACTCCACTACCAAAACCATCATTAAAACAAAAATTTACATGCCACTTAACTCCCCTCACAAACAATCGTTATTTATATTGT<br>TAATTAGCAAACACAAAACCCGC

KY039431	DBKY039431_New Caledonia_Hap_D1	ATTTTATTTTTTAACCTAACTCCCCTACTAAGTGTACCCCCCCTTTCCCCCCCAGGGGGGGTATACTATGCATAATCGTGCATACATTTATATACCACATATATTATGGT<br>ACCGGTAATATATACTATATATGTACTAAACCCATTATATGTATACGGGCATTAATCTATATTCCACATTTCTCCCAATGTCCATTCTATGCATGATCCAGGACATACTC<br>ATTCACCCTCCCCATAGACAGCTCCAAACCACTACCAAGTCACCTAACTATGAATGGTTGCAGGACATAAATCTTACTCTCATGCTCTTCCCCCAACAAGTCACCTAAC<br>TATGAATGGTTGCAGGACATACATTTAACTACC---<br>ATGTTCTAACCCATTTGGTTATGCTCGCCGTATCAGATGGATTTATTGATCGTCCACCTCACGAGAGATCAGCAACCCCTGCCTGTAATGTACTTCATGACCAGTCTCA<br>GGCCCATTCTTTCCCCCTACACCCCTCGCCCTACTTGCCTTCCACCGTACCTCTGGTTCCTCGGTCAGGCACATCCCATGCATAACTCCTGAACTTTCTCACTTTTCAC<br>GAAGTCATCTGTGGATTATCTTCCCCTCTTTAGTCCGTGATCGCGGCATCTTCTCTCTTCTATTGCTGTTGGTTCCTTCTCTTTTTGGGGCTTCTTCACAGGTTACCCTT<br>CACAGTGCGGGTGCGGAGTGCTATTCAAGTGAAGCCTGGACTACACCTGCGTTGCGTCCTATCCTAGTCCTCTCGTGTCCCTCGATGAGACGGTTTGCGTGTATGGGG<br>AATCATCTTGACACTGATGCACTTTGGATCGCATTTGGTTATGGTTCTTCCANCCCCCCC-<br>GGTAAATGGTGCTATTTAGTGAATGCTTGTCGGACATATTTTTATCAATTTTCACTTCCTCTATTTTCTTCACAAAACTAGGAAATTCACCACAATTTTTTCTTTGTTATT<br>TTTTAATTTTTTTTTTATTTTTTAAAAACATTTTTTAAAAAACTAAATTACATACAAACTACCGCATAAAATCCCTCAAACTATACAAACGTTTATCGTATAATATATATAC<br>ATTATTGTTTATTCTATCATTATTAGAGAAACTCCACTACCAAAACCATCATTAAAACAAAAATTTACATGCCACTTAACTCCCCTCACAAACAATCGTTATTTATATTGT<br>TAATTAGCAAACACAAAACCCGC
KY039432	DBKY039432_New Caledonia_Hap_D1	ATTTTATTTTTTAACCTAACTCCCCTACTAAGTGTACCCCCCCTTTCCCCCCCAGGGGGGGTATACTATGCATAATCGTGCATACATTTATATACCACATATATTATGGT<br>ACCGGTAATATATACTATATATGTACTAAACCCATTATATGTATACGGGCATTAATCTATATTCCACATTTCTCCCAATGTCCATTCTATGCATGATCCAGGACATACTC<br>ATTCACCCTCCCCATAGACAGCTCCAAACCACTACCAAGTCACCTAACTATGAATGGTTGCAGGACATAAATCTTACTCTCATGCTCTTCCCCCAACAAGTCACCTAAC<br>TATGAATGGTTGCAGGACATACATTTAACTACC---<br>ATGTTCTAACCCATTTGGTTATGCTCGCCGTATCAGATGGATTTATTGATCGTCCACCTCACGAGAGATCAGCAACCCCTGCCTGTAATGTACTTCATGACCAGTCTCA<br>GGCCCATTCTTTCCCCCTACACCCCTCGCCCTACTTGCCTTCCACCGTACCTCTGGTTCCTCGGTCAGGCACATCCCATGCATAACTCCTGAACTTTCTCACTTTTCAC<br>GAAGTCATCTGTGGATTATCTTCCCCTCTTTAGTCCGTGATCGCGGCATCTTCTCTCTTCTATTGCTGTTGGTTCCTTCTCTTTTTGGGGCTTCTTCACAGGTTACCCTT<br>CACAGTGCGGGTGCGGAGTGCTATTCAAGTGAAGCCTGGACTACACCTGCGTTGCGTCCTATCCTAGTCCTCTCGTGTCCCTCGATGAGACGGTTTGCGTGTATGGGG<br>AATCATCTTGACACTGATGCACTTTGGATCGCATTTGGTTATGGTTCTTCCANCCCCCCC-<br>GGTAAATGGTGCTATTTAGTGAATGCTTGTCGGACATATTTTTATCAATTTTCACTTCCTCTATTTTCTTCACAAAACTAGGAAATTCACCACAATTTTTTCTTTGTTATT<br>TTTTAATTTTTTTTTTATTTTTTAAAAACATTTTTTAAAAAACTAAATTACATACAAACTACCGCATAAAATCCCTCAAACTATACAAACGTTTATCGTATAATATATATAC<br>ATTATTGTTTATTCTATCATTATTAGAGAAACTCCACTACCAAAACCATCATTAAAACAAAAATTTACATGCCACTTAACTCCCCTCACAAACAATCGTTATTTATATTGT<br>TAATTAGCAAACACAAAACCCGC
KY039433	DBKY039433_China_Hap_Z	ATTTTATTTTTTAACCTAACTCCCCTACTAAGTGTACCCCCCCTTTCCCCCCCAGGGGGGGTATACTATGCATAATCGTGCATACATTTATATACCACATATATTATGGT<br>ACCGGTAATATATACTATATATGTACTAAACCCATTATATGTATACGGGCATTAATCTATATTCCACATTTCTCCCAATGTCCATTCTATGCATGATCCAGGACATACTC<br>ATTCACCCTCCCCATAGACAGTTCCAAACCACTACCAAGCCACCTAACTATGAATGGTTACAGGACATAAATCTCACTCTCATGCTCTTCCCCCAACAAGTCACCTAAC<br>TATGAATGGTTACAGGACATACATTTAACTACC---<br>ATGTTCTAACCCATTTGGTTATGCTCGCCGTATCAGATGGATTTATTGATCGTCCACCTCACGAGAGATCAGCAACCCCTGCCTGTAATGTACTTCATGACCAGTCTCA<br>GGCCCATTCTTTCCCCCTACACCCCTCGCCCTACTTGCCTTCCACCGTACCTCTGGTTCCTCGGTCAGGCACATCCCATGCATAACTCCTGAACTTTCTCACTTTTCAC<br>GAAGTCATCTGTGGATTATCTTCCCCTCTTTAGTCCGTGATCGCGGCATCTTCTCTCTTCTATTGCTGTTGGTTCCTTCTCTTTTTGGGGCTTCTTCACAGGTTGCCCTT<br>CACAGTGCGGGTGCGGAGTGCTATTCAAGTGAAGCCTGGACTACACCTGCGTTGCGTCCTATCCTAGTCCTCTCGTGTCCCTCGATGAGACGGTTTGCGTGTATGGGG<br>AATCATCTTGACACTGATGCACTTTGGATCGCATTTGGTTATGGTTCTTCCANCCCCCCC-<br>GGTAAATGGTGCTATTTAGTGAATGCTTGCCGGACATATTTTTATCAATTTTCACTTCCTCTATTTTCTTCACAAAACTAGGAAATTCACCACAATTTTTTCTTTGTTATT<br>TTTTAATTTTTTTTTTATTTTTTAAAAACATTTTTTAAAAAACTAAATTACATACAAACTACCGCATAAAATCCCTCAAACTATACAAACGTTTATCGTATAATATATATAC<br>ATTATTGTTTATTCTATCATTATTAGAGAAACTCCACTACCAAAACCATCATTAAAACAAAAATTTACATGCCACTTAACTCCCCTCACAAACAATCGTTATTTATATTGT<br>TAATTAGCAAACACAAAACCCGC

KY039434	DBKY039434_China_Hap_D1a	ATTTTATTTTTTAACCTAACTCCCCTACTAAGTGTACCCCCCCTTTCCCCCCCAGGGGGGGTATACTATGCATAATCGTGCATACATTTATATACCACATATATTATGGT<br>ACCGGTAATATATACTATATATGTACTAAACCCATTATATGTATACGGGCATTAATCTATATTCCACATTTCTCCCAATGTCCATTCTATGCATGATCCAGGACATACTC<br>ATTCACCCTCCCCATAGACAGCTCCAAACCACTACCAAGTCACCTAACTATGAATGGTTGCAGGACATAAATCTTACTCTCATGCTCTTCCCCCAACAAGTCACCTAAC<br>TATGAATGGTTGCAGGACATACATCTAACTACC--<br>ATGTTCTAACCCATTTGGTTATGCTCGCCGTATCAGATGGATTTATTGATCGTCCACCTCACGAGAGATCAGCAACCCCTGCCTGTAATGTACTTCATGACCAGTCTCA<br>GGCCCATTCTTTCCCCCTACACCCCTCGCCCTACTTGCCTTCCACCGTACCTCTGGTTCCTCGGTCAGGCACATCCCATGCATAACTCCTGAACTTTCTCACTTTTCAC<br>GAAGTCATCTGTGGATTATCTTCCCCTCTTTAGTCCGTGATCGCGGCATCTTCTCTCTTCTATTGCTGTTGGTTCCTTCTCTTTTTGGGGCTTCTTCACAGGTTACCCTT<br>CACAGTGCGGGTGCGGAGTGCTATTCAAGTGAAGCCTGGACTACACCTGCGTTGCGTCCTATCCTAGTCCTCTCGTGTCCCTCGATGAGACGGTTTGCGTGTATGGGG<br>AATCATCTTGACACTGATGCACTTTGGATCGCATTTGGTTATGGTTCTTCCACCCCCCCC-<br>GGTAAATGGTGCTATTTAGTGAATGCTTGTCGGACATATTTTTATCAATTTTCACTTCCTCTATTTTCTTCACAAAACTAGGAAATTCACCACAATTTTTTCTTTGTTATT<br>TTTTAATTTTTTTTTTATTTTTTAAAAACATTTTTTAAAAAACTAAATTACATACAAACTACCGCATAAAATCCCTCAAACTATACAAACGTTTATCGTATAATATATATAC<br>ATTATTGTTTATTCTATCATTATTAGAGAAACTCCACTACCAAAACCATCATTAAAACAAAAATTTACATGCCACTTAACTCCCCTCACAAACAATCGTTATTTATATTGT<br>TAATTAGCAAACACAAAACCCGC
KY039435	DBKY039435_Malacca_Hap_D1	ATTTTATTTTTTAACCTAACTCCCCTACTAAGTGTACCCCCCCTTTCCCCCCCAGGGGGGGTATACTATGCATAATCGTGCATACATTTATATACCACATATATTATGGT<br>ACCGGTAATATATACTATATATGTACTAAACCCATTATATGTATACGGGCATTAATCTATATTCCACATTTCTCCCAATGTCCATTCTATGCATGATCTAGGACATACTC<br>ATTCACCCTCCCCATAGACAGCTCCAAACCACTACCAAGTCACCTAACTATGAATGGTTGCAGGACATAAATCTCACTCTCATGCTCTTCCCCCAACAAGTCACCTAAC<br>TATGAATGGTTACAGGACATACATTTGACTACC--<br>ATGTTCTAACCCATTTGGTTATGCTCGCCGTATCAGATGGATTTATTGATCGTCCACCTCACGAGAGATCAGCAACCCCTGCCTGTAATGTACTTCATGACCAGTCTCA<br>GGCCCATTCTTTCCCCCTACACCCCTCGCCCTACTTGCCTTCCACCGTACCTCTGGTTCCTCGGTCAGGCACATCCCATGCATAACTCCTGAACTTTCTCACTTTTCAC<br>GAAGTCATCTGTGGATTATCTTCCCCTCTTTAGTCCGTGATCGCGGCATCTTCTCTCTTCTATTGCTGTTGGTTCCTTCTCTTTTTGGGGCTTCTTCACAGGTTGCCCTT<br>CACAGTGCGGGTGCGGAGTGCTATTCAAGTGAAGCCTGGACTACACCTGCGTTGCGTCCTATCCTAGTCCTCTCGTGTCCCTCGATGAGACGGTTTGCGTGTATGGGG<br>AATCATCTTGACACTGATGCACTTTGGATCGCATTTGGTTATGGTTCTTCCACCCCCCCC-<br>GGTAAATGGTGCTATTTAGTGAATGCTTGTCGGACATATTTTTATCAATTTTCACTTCCTCTATTTTCTTCACAAAACTAGGAAATTCACCACAATTTTTTCTTTGTTATT<br>TTTTAATTTTTTTTTTATTTTTTAAAAACATTTTTTAAAAAACTAAATTACATACAAACTACCGCATAAAATCCCTCAAACTATACAAACGTTTATCGTATAATATATATAC<br>ATTATTGTTTATTCTATCATTATTAGAGAAACTCCACTACCAAAACCATCATTAAAACAAAAATTTACATGCCACTTAACTCCCCTCACAAACAATCGTTATTTATATTGT<br>TAATTAGCAAACACAAAACCCGC
KY039436	DBKY039436_China_Hap_D1	ATTTTATTTTTTAACCTAACTCCCCTACTAAGTGTACCCCCCCTTTCCCCCCCAGGGGGGGTATACTATGCATAATCGTGCATACATTTATATACCACATATATTATGGT<br>ACCGGTAATATATACTATATATGTACTAAACCCATTATATGTATACGGGCATTAATCTATATTCCACATTTCTCCCAATGTCCATTCTATGCATGATCCAGGACATACTC<br>ATTCACCCTCCCCATAGACAGCTCCAAACCACTACCAAGTCACCTAACTATGAATGGTTGCAGGACATAAATCTTACTCTCATGCTCTTCCCCCAACAAGTCACCTAAC<br>TATGAATGGTTACAGGACATACATTTAACTACC--<br>ATGTTCTAACCCATTTGGTTATGCTCGCCGTATCAGATGGATTTATTGATCGTCCACCTCACGAGAGATCAGCAACCCCTGCCTGTAATGTACTTCATGACCAGTCTCA<br>GGCCCATTCTTTCCCCCTACACCCCTCGCCCTACTTGCCTTCCACCGTACCTCTGGTTCCTCGGTCAGGCACATCCCATGCATAACTCCTGAACTTTCTCACTTTTCAC<br>GAAGTCATCTGTGGATTATCTTCCCCTCTTTAGTCCGTGATCGCGGCATCTTCTCTCTTCTATTGCTGTTGGTTCCTTCTCTTTTTGGGGCTTCTTCACAGGTTACCCTT<br>CACAGTGCGGGTGCGGAGTGCTATTCAAGTGAAGCCTGGACTACACCTGCGTTGCGTCCTATCCTAGTCCTCTCGTGTCCCTCGATGAGACGGTTTGCGTGTATGGGG<br>AATCATCTTGACACTGATGCACTTTGGATCGCATTTGGTTATGGTTCTTCCACCCCCCCC-<br>GGTAAATGGTGCTATTTAGTGAATGCTTGTCGGACATATTTTTATCAATTTTCACTTCCTCTATTTTCTTCACAAAACTAGGAAATTCACCACAATTTTTTCTTTGTTATT<br>TTTTAATTTTTTTTTTATTTTTTAAAAACATTTTTTAAAAAACTAAATTACATACAAACTACCGCATAAAATCCCTCAAACTATACAAACGTTTATCGTATAATATATATAC<br>ATTATTGTTTATTCTATCATTATTAGAGAAACTCCACTACCAAAACCATCATTAAAACAAAAATTTACATGCCACTTAACTCCCCTCACAAACAATCGTTATTTATATTGT<br>TAATTAGCAAACACAAAACCCGC

KY039437	DBKY039437_Philippines_Hap_D1a	ATTTTATTTTTTAACCTAACTCCCCTACTAAGTGTACCCCCCCTTTCCCCCCCAGGGGGGGTATACTATGCATAATCGTGCATACATTTATATACCACATATATTATGGT<br>ACCGGTAATATATACTATATATGTACTAAACCCATTATATGTATACGGGCATTAATCTATATTCCACATTTCTCCCAATGTCCATTCTATGCATGATCCAGGACATACTC<br>ATTCACCCTCCCCATAGACAGCTCCAAACCACTACCAAGTCACCTAACTATGAATGGTTGCAGGACATAAATCTTACTCTCATGCTCTTCCCCCAACAAGTCACCTAAC<br>TATGAATGGTTGCAGGACATACATTTAACTACC---------------------------------<br>ATGTTCTAACCCATTTGGTTATGCTCGCCGTATCAGATGGATTTATTGATCGTCCACCTCACGAGAGATCAGCAACCCCTGCCTGTAATGTACTTCATGACCAGTCTCA<br>GGCCCATTCTTTCCCCCTACACCCCTCGCCCTACTTGCCTTCCACCGTACCTCTGGTTCCTCGGTCAGGCACATCCCATGCATAACTCCTGAACTTTCTCACTTTTCAC<br>GAAGTCATCTGTGGATTATCTTCCCCTCTTTAGTCCGTGATCGCGGCATCTTCTCTCTTCTATTGCTGTTGGTTCCTTCTCTTTTTGGGGCTTCTTCACAGGTTACCCTT<br>CACAGTGCGGGTGCGGAGTGCTATTCAAGTGAAGCCTGGACTACACCTGCGTTGCGTCCTATCCTAGTCCTCTCGTGTCCCTCGATGAGACGGTTTGCGTGTATGGGG<br>AATCATCTTGACACTGATGCACTTTGGATCGCATTTGGTTATGGTTCTTCCANCCCCCCC-<br>GGTAAATGGTGCTATTTAGTGAATGCTTGTCGGACATATTTTTATCAATTTTCACTTCCTCTATTTTCTTCACAAAACTAGGAAATTCACCACAATTTTTTCTTTGTTATT<br>TTTTAATTTTTTTTTTATTTTTTAAAAACATTTTTTAAAAAACTAAATTACATACAAACTACCGCATAAAATCCCTCAAACTATACAAACGTTTATCGTATAATATATATAC<br>ATTATTGTTTATTCTATCATTATTAGAGAAACTCCACTACCAAAACCATCATTAAAACAAAAATTTACATGCCACTTAACTCCCCTCACAAACAATCGTTATTTATATTGT<br>TAATTAGCAAACACAAAACCCGC
KY054997	DBKY054997_China_Hap_A	ATTTTATTTTTTAACCTAACTCCCCTACTAAGTGTACCCCCCCTTTCCCCCCCAGGGGGGGTATACTATGCATAATCGTGCATACATTTATATACCACATATATTATGGT<br>ACCGGTAATATATACTATATATGTACTAAACCCATTATATGTATACGGGCATTAACCTATATTCCACATTTCTCCCAATGTCCATTCTATGCATGATCCAGGACATACTC<br>ATTTACCCTCCCCATAGACAGTTCCAAACCACTATCAAGCCACCTAACTATGAATGGTTACAGGACATAAATCTCACTCTCATGTTCTCCCCCCAACAAGTCACCTAAC<br>TATGAATGGTTACAGGACATACATTTAACTACC---------------------------------<br>ATGTTCTAACCCATTTGGTTATGCTCGCCGTATCAGATGGATTTATTGATCGTCCACCTCACGAGAGATCAGCAACCCCTGCCTGTAATGTACTTCATGACCAGTCTCA<br>GGCCCATTCTTTCCCCCTACACCCCTCGCCCTACTTGCCTTCCACCGTACCTCTGGTTCCTCGGTCAGGCACATCCCATGCATAACTCCTGAACTTTCTCACTTTTCAC<br>GAAGTCATCTGTGGATTATCTTCCCCTCTTTAGTCCGTGATCGCGGCATCTTCTCTCTTCTATTGCTGTTGGTTCCTTCTCTTTTTGGGGCTTCTTCACAGGTTGCCCTT<br>CACAGTGCGGGTGCGGAGTGCTATTCAAGTGAAGCCTGGACTACACCTGCGTTGCGTCCTATCCTAGTCCTCTCGTGTCCCTCGATGAGACGGTTTGCGTGTATGGGG<br>AATCATCTTGACACTGATGCACTTTGGATCGCATTTGGTTATGGTTCTTCCACCCCCCCC--<br>GGTAAATGGTGCTATTTAGTGAATGCTTGTCGGACATATTTTTATCAATTTTCACTTCCTCTATTTTCTTCACAAAACTAGGAAATTCACCACAATTTTTTCTTTGTTATT<br>TTTTAATTTTTTTTTTATTTTTTAAAAACATTTTTTAAAAAACTAAATTACATACAAACTACCGCATAAAATCCCTCAAACTATACAAACGTTTATCGTATAATATATATAC<br>ATTATTGTTTATTCTATCATTATTAGAGAAACTCCACTACCAAAACCATCATTAAAACAAAAATTTACATGCCACTTAACTCCCCTCACAAACAATCGTTATTTATATTGT<br>TAATTAGCAAACACAAAACCCGC
MG605671	DBMG605671_Thailand_Hap_X	ATTTTATTTTTTAACCTAACTCCCCTACTAAGTGTACCCCCCCTTTCCCCCCCAGGGGGGGTATACTATGCATAATCGTGCATACATTTATATACCACATATATTATGGT<br>ACCGGTAATATATACTATATATGTACTAAACCCATTATATGTATACGGGCATTAATCTATATTCCACATTTCTCCCAATGTCCATTCTATGCATGATCCAGGACATACTC<br>ATTCACCCTCCCCATAGACAGCTCCAAACCACTACCAAGTCACCTAACTATGAATGGTTACAGGACATAAATCTCACTCTCATGTTCTTCCCCCAACAAGTCACCTAAC<br>TATGAATGGTTACAGGACATACATTTAACTACT---------------------------------<br>ATGTTCTAACCCATTTGGTTATGCTCGCCGTATCAGATGGATTTATTGATCGTCCACCTCACGAGAGATCAGCAACCCCTGCTCGTAATGTACTTCATGACCAGTCTCA<br>GGCCCATTCTTTCCCCCTACACCCCTCGCCCTACTTGCCTTCCACCGTACCTCTGGTTCCTCGGTCAGGCACATCCCATGCATAACTCCTGAACTTTCTCACTTTTCAC<br>GAAGTCATCTGTGGATTATCTTCCCCTCTTTAGTCCGTGATCGCGGCATCTTCTCTCTTCTATTGCTGTTGGTTCCTTCTCTTTTTGGGGCTTCTTCACAGGTTGCCCTT<br>CACAGTGCGGGTGCGGAGTGCTATTCAAGTGAAGCCTGGACTACACCTGCGTTGCGTCCTATCCTAGTCCTCTCGTGTCCCTCGATGAGACGGTTTGCGTGTATGGGG<br>AATCATCTTGACACTGATGCACTTTGGATCGCATTTGGTTATGGTTCTTCCACCCCCCCCC-<br>GGTAAATGGTGCTATTTAGTGAATGCTTGTCGGACATATTTTTATCAATTTTCACTTCCTCTATTTTCTTCACAAAACTAGGAAATTCACCACAATTTTTTCTTTGTTATT<br>TTTTAATTTTTTTTTTATTTTTTAAAAACATTTTTTAAAAAACTAAATTACATACAAACTACCGCATAAAATCCCTCAAACTATACAAACGTTTATCGTATAATATATATAC<br>ATTATTGTTTATTCTATCATTATTAGAGAAACTCCACTACCAAAACCATCATTAAAACAAAAATTTACATGCCACTTAACTCCCCTCACAAACAATCGTTATTTATATTGT<br>TAATTAGCAAACACAAAACCTGC

MG837547	DBMG837547_China_Hap_E1	ATTTTATTTTTTAACCTAACTCCCCTACTAAGTGTACCCCCCCTTTCCCCCCCAGGGGGGGTATACTATGCATAATCGTGCATACATTTATATACCACATATATTATGGT<br>ACCGGTAATATATACTATATATGTACTAAACCCATTATATGTATACGGGCATTAATCTATATTCCACATTTCTCCCAATGTCCATTCTATGCATGATCCAGGACACACTC<br>ATTCACCCTCCCCATAGACAGCTCCAAACCACTACCAAGTCACCTAACTATGAATGGTTACAGGACATAAATCTCACTCTCATGTTCTTCCCCCAACAAGTCACCTAAC<br>TATGAATGGTTACAGGACATACATTTAACTACC--<br>ATGTTCTAACCCATTTGGTTATGCTCGCCGTATCAGATGGATTTATTGATCGTCCACCTCACGAGAGATCAGCAACCCCTGCTTGTAATGTACTTCATGACCAGTCTCA<br>GGCCCATTCTTTCCCCCTACACCCCTCGCCCTACTTGCCTTCCACCGTACCTCTGGTTCCTCGGTCAGGCACATCCCATGCATAACTCCTGAACTTTCTCACTTTTCAC<br>GAAGTCATCTGTGGATTATCTTCCCCTCTTTAGTCCGTGATCGCGGCATCTTCTCTCTTCTATTGCTGTTGGTTCCTTCTCTTTTTGGGGCTTCTTCACAGGTTGCCCTT<br>CACAGTGCGGGTGCGGAGTGCTATTCAAGTGAAGCCTGGACTACACCTGCGTTGCGTCCTATCCTAGTCCTCTCGTGTCCCTCGATGAGACGGTTTGCGTGTATGGGG<br>AATCATCTTGACACTGATGCACTTTGGATCGCATTTGGTTATGGTTCTTCCACCCCCCC--<br>GGTAAATGGTGCTATTTAGTGAATGCTTGTCGGACATATTTTTATCAATTTTCACTTCCTCTATTTTCTTCACAAAACTAGGAAATTCACCACAATTTTTTCTTTGTTATT<br>TTTTAATTTTTTTTTTATTTTTTAAAAACATTTTTTAAAAAACTAAATTACATACAAACTACCGCATAAAATCCCTCAAACTATACAAACGTTTATCGTATAATATATATAC<br>ATTATTGTTTATTCTATCATTATTAGAGAAACTCCACTACCAAAACCATCATTAAAACAAAAATTTACATGCCACTTAACTCCCCTCACAAACAATCGTTATTTATATTGT<br>TAATTAGCAAACACAAAACCTGC
MG837548	DBMG837548_China_Hap_E1	ATTTTATTTTTTAACCTAACTCCCCTACTAAGTGTACCCCCCCTTTCCCCCCCAGGGGGGGTATACTATGCATAATCGTGCATACATTTATATACCACATATATTATGGT<br>ACCGGTAATATATACTATATATGTACTAAACCCATTATATGTATACGGGCATTAATCTATATTCCACATTTCTCCCAATGTCCATTCTATGCATGATCCAGGACACACTC<br>ATTCACCCTCCCCATAGACAGCTCCAAACCACTACCAAGTCACCTAACTATGAATGGTTACAGGACATAAATCTCACTCTCATGTTCTTCCCCCAACAAGTCACCTAAC<br>TATGAATGGTTACAGGACATACATTTAACTACC--<br>ATGTTCTAACCCATTTGGTTATGCTCGCCGTATCAGATGGATTTATTGATCGTCCACCTCACGAGAGATCAGCAACCCCTGCTTGTAATGTACTTCATGACCAGTCTCA<br>GGCCCATTCTTTCCCCCTACACCCCTCGCCCTACTTGCCTTCCACCGTACCTCTGGTTCCTCGGTCAGGCACATCCCATGCATAACTCCTGAACTTTCTCACTTTTCAC<br>GAAGTCATCTGTGGATTATCTTCCCCTCTTTAGTCCGTGATCGCGGCATCTTCTCTCTTCTATTGCTGTTGGTTCCTTCTCTTTTTGGGGCTTCTTCACAGGTTGCCCTT<br>CACAGTGCGGGTGCGGAGTGCTATTCAAGTGAAGCCTGGACTACACCTGCGTTGCGTCCTATCCTAGTCCTCTCGTGTCCCTCGATGAGACGGTTTGCGTGTATGGGG<br>AATCATCTTGACACTGATGCACTTTGGATCGCATTTGGTTATGGTTCTTCCACCCCCCCC-<br>GGTAAATGGTGCTATTTAGTGAATGCTTGTCGGACATATTTTTATCAATTTTCACTTCCTCTATTTTCTTCACAAAACTAGGAAATTCACCACAATTTTTTCTTTGTTATT<br>TTTTAATTTTTTTTTTATTTTTTAAAAACATTTTTTAAAAAACTAAATTACATACAAACTACCGCATAAAATCCCTCAAACTATACAAACGTTTATCGTATAATATATATAC<br>ATTATTGTTTATTCTATCATTATTAGAGAAACTCCACTACCAAAACCATCATTAAAACAAAAATTTACATGCCACTTAACTCCCCTCACAAACAATCGTTATTTATATTGT<br>TAATTAGCAAACACAAAACCTGC
MH732978	DBMH732978_China_Hap_B	ATTTTATTTTTTAACCTAACTCCCCTACTAAGTGTACCCCCCCTTTCCCCCCCAGGGGGGGTATACTATGCATAATCGTGCATACATTTATATACCACATATATTATGGT<br>ACCGGTAATATATACTATATATGTACTAAACCCATTATATGTATACGGGCATTAATCTATATTCCACATTTCTCCCAATGTCCATTCTATGCATGATCCAAGACATACTC<br>ATTCACCCTCCCCATAGACAGTTCTAAACCACTATCAAGCCACCTAACTATGAATGGTTACAGGACATAAATCTCACTCTCATGTTCTCCCCCTAACAAGTCACCTAAC<br>TATGAATGGTTACAGGACATACATTTAACTACC--<br>ATGTTCTAACCCATTTGGTTATGCTCGCCGTATCAGATGGATTTATTGATCGTCCACCTCACGAGAGATCAGCAACCCCTGCCTGTAATGTACTTCATGACCAGTCTCA<br>GGCCCATTCTTTCCCCCTACACCCCTCGCCCTACTTGCCTTCCACCGTACCTCTGGTTCCTCGGTCAGGCACATCCCATGCATAACTCCTGAACTTTCTCACTTTTCAC<br>GAAGTCATCTGTGGATTATCTTCCCCTCTTTAGTCCGTGATCGCGGCATCTTCTCTCTTCTATTGCTGTTGGTTCCTTCTCTTTTTGGGGCTTCTTCACAGGTTGCCCTT<br>CACAGTGCGGGTGCGGAGTGCTATTCAAGTGAAGCCTGGACTACACCTGCGTTGCGTCCTATCCTAGTCCTCTCGTGTCCCTCGATGAGACGGTTTGCGTGTATGGGG<br>AATCATCTTGACACTGATGCACTTTGGATCGCATTTGGTTATGGTTCTTCCACCCCCCCC-<br>GGTAAATGGTGCTATTTAGTGAATGCTTGTCGGACATATTTTTATCAATTTTCACTTCCTCTATTTTCTTCACAAAACTAGGAAATTCACCACAATTTTTTCTTTGTTATT<br>TTTTAATTTTTTTTTTATTTTTTAAAAACATTTTTTAAAAAACTAAATTACATACAAACTACCGCATAAAATCCCTCAAACTATACAAACGTTTATCGTATAATATATATAC<br>ATTATTGTTTATTCTATCATTATTAGAGAAACTCCACTACCAAAACCATCATTAAAACAAAAATTTACATGCCACTTAACTCCCCTCACAAACAATCGTTATTTATATTGT<br>TAATTAGCAAACACAAAACCCAC

MH879470	DBMH879470_China_Hap_B	ATTTTATTTTTTAACCTAACTCCCCTACTAAGTGTACCCCCCCTTTCCCCCCCAGGGGGGGTATACTATGCATAATCGTGCATACATTTATATACCACATATATTATGGT<br>ACCGGTAATATATACTATATATGTACTAAACCCATTATATGTATACGGGCATTAATCTATATTCCACATTTCTCCCAATGTCCATTCTATGCATGATCCAAGACATATTC<br>ATTCACCCTCCCCATAGACAGTTCTAAACCACTATCAAGCCACCTAACTATGAATGGTTACAGGACATAAATCTCACTCTCATGTTCTCCCCCTAACAAGTCACCTAAC<br>TATGAATGGTTACAGGACATACATTTAACTACC--<br>ATGTTCTAACCCATTTGGTTATGCTCGCCGTATCAGATGGATTTATTGATCGTCCACCTCACGAGAGATCAGCAACCCCTGCCTGTAATGTACTTCATGACCAGTCTCA<br>GGCCCATTCTTTCCCCCTACACCCCTCGCCCTACTTGCCTTCCACCGTACCTCTGGTTCCTCGGTCAGGCACATCCCATGCATAACTCCTGAACTTTCTCACTTTTCAC<br>GAAGTCATCTGTGGATTATCTTCCCCTCTTTAGTCCGTGATCGCGGCATCTTCTCTCTTCTATTGCTGTTGGTTCCTTCTCTTTTTGGGGCTTCTTCACAGGTTGCCCTT<br>CACAGTGCGGGTGCGGAGTGCTATTCAAGTGAAGCCTGGACTACACCTGCGTTGCGTCCTATCCTAGTCCTCTCGTGTCCCTCGATGAGACGGTTTGCGTGTATGGGG<br>AATCATCTTGACACTGATGCACTTTGGATCGCATTTGGTTATGGTTCTTCCACCCCCCC--<br>GGTAAATGGTGCTATTTAGTGAATGCTTGTCGGACATATTTTTATCAATTTTCACTTCCTCTATTTTCTTCACAAAACTAGGAAATTCACCACAATTTTTTCTTTGTTATT<br>TTTTAATTTTTTTTTTATTTTTTAAAAACATTTTTTAAAAAACTAAATTACATACAAACTACCGCATAAAATCCCTCAAACTATACAAACGTTTATCGTATAATATATATAC<br>ATTATTGTTTATTCTATCATTATTAGAGAAACTCCACTACCAAAACCATCATTAAAACAAAAATTTACATGCCACTTAACTCCCCTCACAAACAATCGTTATTTATATTGT<br>TAATTAGCAAACACAAAACCCAC
MK163559	DBMK163559_China_Hap_D3	ATTTTATTTTTTAACCTAACTCCCCTACTAAGTGTACCCCCCCTTTCCCCCCCAGGGGGGGTATACTATGCATAATCGTGCATACATTTATATACCACATATATTATGGT<br>ACCGGTAATATATACTATATATGTACTAAACCCATTATATGTATACGGGCATTAATCTATATTCCACATTTCTCCCAATGTCCATTCTATGCATGATCCAGGACATACTC<br>ATTCACCCTCCCCATAGACAGCTCCAAACCACTACCAAGCCACCTAACTATGAATGGTTGCAGGACATAAATCTCACTCTCATGCTCTTCCCCCAACAAGTCACCTAAC<br>TATGAATGGTTACAGGACATACATCTAACTACC--<br>ATGTTCTAACCCATTTGGTTATGCTCGTCGTATCAGATGGATTTATTGATCGTCCACCTCACGAGAGATCAGCAACCCCTGCCTGTAATGTACTTCATGACCAGTCTCA<br>GGCCCATTCTTTCCCCCTACACCCCTCGCCCTACTTGCCTTCCACCGTACCTCTGGTTCCTCGGTCAGGCACATCCCATGCATAACTCCTGAACTTTCTCACTTTTCAC<br>GAAGTCATCTGTGGATTATCTTCCCCTCTTTAGTCCGTGATCGCGGCATCTTCTCTCTTCTATTGCTGTTGGTTCCTTCTCTTTTTGGGGCTTCTTCACAGGTTGCCCTT<br>CACAGTGCGGGTGCGGAGTGCTATTCAAGTGAAGCCTGGACTACACCTGCGTTGCGTCCTATCCTAGTCCTCTCGTGTCCCTCGATGAGACGGTTTGCGTGTATGGGG<br>AATCATCTTGACACTGATGCACTTTGGATCGCATTTGGTTATGGTTCTTCCACCCCCCCC-<br>GGTAAATGGTGCTATTTAGTGAATGCTTGTCGGACATATTTTTATCAATTTTCACTTCCTCTATTTTCTTCACAAAACTAGGAAATTCACCACAATTTTTTCTTTGTTATT<br>TTTTAATTTTTTTTTTATTTTTTAAAAACATTTTTTAAAAAACTAAATTACATACAAACTACCGCATAAAATCCCTCAAACTATACAAACGTTTATCGTATAATATATATAC<br>ATTATTGTTTATTCTATCATTATTAGAGAAACTCCACTACCAAAACCATCATTAAAACAAAAATTTACATGCCACTTAACTCCCCTCACAAACAATCGTTATTTATATTGT<br>TAATTAGCAAACACAAAACCCGC
MK163560	DBMK163560_China_Hap_A	ATTTTATTTTTTAACCTAACTCCCCTACTAAGTGTACCCCCCCTTTCCCCCCCAGGGGGGGTATACTATGCATAATCGTGCATACATTTATATACCACATATATTATGGT<br>ACCGGTAATATATACTATATATGTACTAAACCCATTATATGTATACGGGCATTAACCTATATTCCACATTTCTCCCAATGTCCATTCTATGCATGATCCAGGACATACTC<br>ATTTACCCTCCCCATAGACAGTTCCAAACCACTATCAAGCCACCTAACTATGAATGGTTACAGGACATAAATCTCACTCTCATGTTCTCCCCCCAACAAGTCACCTAAC<br>TATGAATGGTTACAGGACATACATTTAACTACC--<br>ATGTTCTAACCCATTTGGTTATGCTCGCCGTACCAGATGGATTTATTGATCGTCCACCTCACGAGAGATCAGCAACCCCTGCCTGTAATGTACTTCATGACCAGTCTCA<br>GGCCCATTCTTTCCCCCTACACCCCTCGCCCTACTTGCCTTCCACCGTACCTCTGGTTCCTCGGTCAGGCACATCCCATGCATAACTCCTGAACTTTCTCACTTTTCAC<br>GAAGTCATCTGTGGATTATCTTCCCCTCTTTAGTCCGTGATCGCGGCATCTTCTCTCTTCTATTGCTGTTGGTTCCTTCTCTTTTTGGGGCTTCTTCACAGGTTGCCCTT<br>CACAGTGCGGGTGCGGAGTGCTATTCAAGTGAAGCCTGGACTACACCTGCGTTGCGTCCTATCCTAGTCCTCTCGTGTCCCTCGATGAGACGGTTTGCGTGTATGGGG<br>AATCATCTTGACACTGATGCACTTTGGATCGCATTTGGTTATGGTTCTTCCACCCCCCCC-<br>GGTAAATGGTGCTATTTAGTGAATGCTTGTCGGACATATTTTTATCAATTTTCACTTCCTCTATTTTCTTCACAAAACTAGGAAATTCACCACAATTTTTTCTTTGTTATT<br>TTTTAATTTTTTTTTTATTTTTTAAAAACATTTTTTAAAAAACTAAATTACATACAAACTACCGCATAAAATCCCTCAAACTATACAAACGTTTATCGTATAATATATATAC<br>ATTATTGTTTATTCTATCATTATTAGAGAAACTCCACTACCAAAACCATCATTAAAACAAAAATTTACATGCCACTTAACTCCCCTCACAAACAATCGTTATTTATATTGT<br>TAATTAGCAAACACAAAACCCGC

MK163561	DBMK163561_China_Hap_A	ATTTTATTTTTTAACCTAACTCCCCTACTAAGTGTACCCCCCCTTTCCCCCCCAGGGGGGGTATACTATGCATAATCGTGCATACATTTATATACCACATATATTATGGT<br>ACCGGTAATATATACTATATATGTACTAAACCCATTATATGTATACGGGCATTAACCTATATTCCACATTTCTCCCAATGTCCATTCTATGCATGATCCAGGACATACTC<br>ATTTACCCTCCCCATAGACAGTTCCAAACCACTATCAAGCCACCTAACTATGAATGGTTACAGGACATAAATCTCACTCTCATGTTCTCCCCCCAACAAGTCACCTAAC<br>TATGAATGGTTACAGGACATACATTTAACTACC--<br>ATGTTCTAACCCATTTGGTTATGCTCGCCGTATCAGATGGATTTATTGATCGTCCACCTCACGAGAGATCAGCAACCCCTGCCTGTAATGTACTTCATGACCAGTCTCA<br>GGCCCATTCTTTCCCCCTACACCCCTCGCCCTACTTGCCTTCCACCGTACCTCTGGTTCCTCGGTCAGGCACATCCCATGCATAACTCCTGAACTTTCTCACTTTTCAC<br>GAAGTCATCTGTGGATTATCTTCCCCTCTTTAGTCCGTGATCGCGGCATCTTCTCTCTTCTATTGCTGTTGGTTCCTTCTCTTTTTGGGGCTTCTTCACAGGTTGCCCTT<br>CACAGTGCGGGTGCGGAGTGCTATTCAAGTGAAGCCTGGACTACACCTGCGTTGCGTCCTATCCTAGTCCTCTCGTGTCCCTCGATGAGACGGTTTGCGTGTATGGGG<br>AATCATCTTGACACTGATGCACTTTGGATCGCATTTGGTTATGGTTCTTCCACCCCCCC--<br>GGTAAATGGTGCTATTTAGTGAATGCTTGTCGGACATATTTTTATCAATTTTCACTTCCTCTATTTTCTTCACAAAACTAGGAAATTCACCACAATTTTTTCTTTGTTATT<br>TTTTAATTTTTTTTTTATTTTTTAAAAACATTTTTTAAAAAACTAAATTACATACAAACTACCGCATAAAATCCCTCAAACTATACAAACGTTTATCGTATAATATATATAC<br>ATTATTGTTTATTCTATCATTATTAGAGAAACTCCACTACCAAAACCATCATTAAAACAAAAATTTACATGCCACTTAACTCCCCTCACAAACAATCGTTATTTATATTGT<br>TAATTAGCAAACACAAAACCCGC
MK163562	DBMK163562_China_Hap_D3	ATTTTATTTTTTAACCTAACTCCCCTACTAAGTGTACCCCCCCTTTCCCCCCCAGGGGGGGTATACTATGCATAATCGTGCATACATTTATATACCACATATATTATGGT<br>ACCGGTAATATATACTATATATGTACTAAACCCATTATATGTATACGGGCATTAATCTATATTCCACATTTCTCCCAATGTCCATTCTATGCATGATCCAGGACATACTC<br>ATTCACCCTCCCCATAGACAGCTCCAAACCACTACCAAGTCACCTAACTATGAATGGTTGCAGGACATAAATCTCACTCTCATGCTCTTCCCCCAACAAGTCACCTAAC<br>TATGAATGGTTACAGGACATACATCTAACTACC--<br>ATGTTCTAACCCATTTGGTTATGCTCGTCGTATCAGATGGATTTATTGATCGTCCACCTCACGAGAGATCAGCAACCCCTGCCTGTAATGTACTTCATGACCAGTCTCA<br>GGCCCATTCTTTCCCCCTACACCCCTCGCCCTACTTGCCTTCCACCGTACCTCTGGTTCCTCGGTCAGGCACATCCCATGCATAACTCCTGAACTTTCTCACTTTTCAC<br>GAAGTCATCTGTGGATTATCTTCCCCTCTTTAGTCCGTGATCGCGGCATCTTCTCTCTTCTATTGCTGTTGGTTCCTTCTCTTTTTGGGGCTTCTTCACAGGTTGCCCTT<br>CACAGTGCGGGTGCGGAGTGCTATTCAAGTGAAGCCTGGACTACACCTGCGTTGCGTCCTATCCTAGTCCTCTCGTGTCCCTCGATGAGACGGTTTGCGTGTATGGGG<br>AATCATCTTGACACTGATGCACTTTGGATCGCATTTGGTTATGGTTCTTCCACCCCCCCC-<br>GGTAAATGGTGCTATTTAGTGAATGCTTGTCGGACATATTTTTATCAATTTTCACTTCCTCTATTTTCTTCACAAAACTAGGAAATTCACCACAATTTTTTCTTTGTTATT<br>TTTTAATTTTTTTTTTATTTTTTAAAAACATTTTTTAAAAAACTAAATTACATACAAACTACCGCATAAAATCCCTCAAACTATACAAACGTTTATCGTATAATATATATAC<br>ATTATTGTTTATTCTATCATTATTAGAGAAACTCCACTACCAAAACCATCATTAAAACAAAAATTTACATGCCACTTAACTCCCCTCACAAACAATCGTTATTTATATTGT<br>TAATTAGCAAACACAAAACCCGC
MK163563	DBMK163563_China_Hap_E1	ATTTTATTTTTTAACCTAACTCCCCTACTAAGTGTACCCCCCCTTTCCCCCCCAGGGGGGGTATACTATGCATAATCGTGCATACATTTATATACCACATATATTATGGT<br>ACCGGTAATATATACTATATATGTACTAAACCCATTATATGTATACGGGCATTAATCTATATTCCACATTTCTCCCAATGTCCATTCCATGCATGATCCAGGACACACTC<br>ATTCACCCTCCCCATAGACAGCTCCAAACCACTACCAAGTCACCTAACTATGAATGGTTACAGGACATAAATCTCACTCTCATGTTCTTCCCCCAACAAGTCACCTAAC<br>TATGAATGGTTACAGGACATACATTTAACTACC--<br>ATGTTCTAACCCATTTGGTTATGCTCGCCGTATCAGATGGATTTATTGATCGTCCACCTCACGAGAGATCAGCAACCCCTGCTTGTAATGTACTTCATGACCAGTCTCA<br>GGCCCATTCTTTCCCCCTACACCCCTCGCCCTACTTGCCTTCCACCGTACCTCTGGTTCCTCGGTCAGGCACATCCCATGCATAACTCCTGAACTTTCTCACTTTTCAC<br>GAAGTCATCTGTGGATTATCTTCCCCTCTTTAGTCCGTGATCGCGGCATCTTCTCTCTTCTATTGCTGTTGGTTCCTTCTCTTTTTGGGGCTTCTTCACAGGTTGCCCTT<br>CACAGTGCGGGTGCGGAGTGCTATTCAAGTGAAGCCTGGACTACACCTGCGTTGCGTCCTATCCTAGTCCTCTCGTGTCCCTCGATGAGACGGTTTGCGTGTATGGGG<br>AATCATCTTGACACTGATGCACTTTGGATCGCATTTGGTTATGGTTCTTCCACCCCCCC--<br>GGTAAATGGTGCTATTTAGTGAATGCTTGTCGGACATATTTTTATCAATTTTCACTTCCTCTATTTTCTTCACAAAACTAGGAAATTCACCACAATTTTTTCTTTGTTATT<br>TTTTAATTTTTTTTTTATTTTTTAAAAACATTTTTTAAAAAACTAAATTACATACAAACTACCGCATAAAATCCCTCAAACTATACAAACGTTTATCGTATAATATATATAC<br>ATTATTGTTTATTCTATCATTATTAGAGAAACTCCACTACCAAAACCATCATTAAAACAAAAATTTACATGCCACTTAACTCCCCTCACAAACAATCGTTATTTATATTGT<br>TAATTAGCAAACACAAAACCTGC

MK163564	DBMK163564_China_Hap_B1a	ATTTTATTTTTTAACCTAACTCCCCTACTAAGTGTACCCCCCCTTTCCCCCCCAGGGGGGGTATACTATGCATAATCGTGCATACATTTATATACCACATATATTATGGT<br>ACCGGTAATATATACTATATATGTACTAAACCCATTATATGTATACGGGCATTAATCTATATTCCACATTTCTCCCAATGTCCATTCTATGCATGATCCAAGACATACTC<br>ATTCACCCTCCCCATAGACAGTTCTAAACCACTATCAAGCCACCTAACTATGAATGGTTACAGGACATAAATCTTACTCTCATGTTCTCCCCCTAACAAGTCACCTAAC<br>TATGAATGGTTACAGGACATACATTTAACTACC--<br>ATGTTCTAACCCATTTGGTTATGCTCGCCGTATCAGATGGATTTATTGATCGTCCACCTCACGAGAGATCAGCAACCCCTGCCTGTAATGTACTTCATGACCAGTCTCA<br>GGCCCATTCTTTCCCCCTACACCCCTCGCCCTACTTGCCTTCCACCGTACCTCTGGTTCCTCGGTCAGGCACATCCCATGCATAACTCCTGAACTTTCTCACTTTTCAC<br>GAAGTCATCTGTGGATTATCTTCCCCTCTTTAGTCCGTGATCGCGGCATCTTCTCTCTTCTATTGCTGTTGGTTCCTTCTCTTTTTGGGGCTTCTTCACAGGTTGCCCTT<br>CACAGTGCGGGTGCGGAGTGCTATTCAAGTGAAGCCTGGACTACACCTGCGTTGCGTCCTATCCTAGTCCTCTCGTGTCCCTCGATGAGACGGTTTGCGTATATGGGG<br>AATCATCTTGACACTGATGCACTTTGGATCGCATTTGGTTATGGTTCTTCCACCCCCCCC-<br>GGTAAATGGTGCTATTTAGTGAATGCTTGTCGGACATATTTTTATCAATTTTCACTTCCTCTATTTTCTTCACAAAACTAGGAAATTCACCACAATTTTTTCTTTGTTATT<br>TTTTAATTTTTTTTTTATTTTTTAAAAACATTTTTTAAAAAACTAAATTACATACAAACTACCGCATAAAATCCCTCAAACTATACAAACGTTTATCGTATAATATATATAC<br>ATTATTGTTTATTCTATCATTATTAGAGAAACTCCACTACCAAAACCATCATTAAAACAAAAATTTACATGCCACTTAACTCCCCTCACAAACAATCGTTATTTATATTGT<br>TAATTAGCAAACACAAAACCCAC
MK163565	DBMK163565_China_Hap_B	ATTTTATTTTTTAACCTAACTCCCCTACTAAGTGTACCCCCCCTTTCCCCCCCAGGGGGGGTATACTATGCATAATCGTGCATACATTTATATACCACATATATTATGGT<br>ACCGGTAATATATACTATATATGTACTAAACCCATTATATGTATACGGGCATTAATCTATATTCCACATTTCTCCCAATGTCCATTCTATGCATGATCCAAGACATACTC<br>ATTCACCCTCCCCATAGACAGTTCTAAACCACTATCAAGCCACCTAACTATGAATGGTTACAGGACATAAATCTCACTCTCATGTTCTCCCCCTAACAAGTCACCTAAC<br>TATGAATGGTTACAGGACATACATTTAACTACC--<br>ATGTTCTAACCCATTTGGTTATGCTCGCCGTATCAGATGGATTTATTGATCGTCCACCTCACGAGAGATCAGCAACCCCTGCCTGTAATGTACTTCATGACCAGTCTCA<br>GGCCCATTCTTTCCCCCTACACCCCTCGCCCTACTTGCCTTCCACCGTACCTCTGGTTCCTCGGTCAGGCACATCCCATGCATAACTCCTGAACTTTCTCACTTTTCAC<br>GAAGTCATCTGTGGATTATCTTCCCCTCTTTAGTCCGTGATCGCGGCATCTTCTCTCTTCTATTGCTGTTGGTTCCTTCTCTTTTTGGGGCTTCTTCACAGGTTGCCCTT<br>CACAGTGCGGGTGCGGAGTGCTATTCAAGTGAAGCCTGGACTACACCTGCGTTGCGTCCTATCCTAGTCCTCTCGTGTCCCTCGATGAGACGGTTTGCGTGTATGGGG<br>AATCATCTTGACACTGATGCACTTTGGATCGCATTTGGTTATGGTTCTTCCACCCCCCCC-<br>GGTAAATGGTGCTATTTAGTGAATGCTTGTCGGACATATTTTTATCAATTTTCACTTCCTCTATTTTCTTCACAAAACTAGGAAATTCACCACAATTTTTTCTTTGTTATT<br>TTTTAATTTTTTTTTTATTTTTTAAAAACATTTTTTAAAAAACTAAATTACATACAAACTACCGCATAAAATCCCTCAAACTATACAAACGTTTATCGTATAATATATATAC<br>ATTATTGTTTATTCTATCATTATTAGAGAAACTCCACTACCAAAACCATCATTAAAACAAAAATTTACATGCCACTTAACTCCCCTCACAAACAATCGTTATTTATATTGT<br>TAATTAGCAAACACAAAACCCAC
MN013407	DBMN013407_China_Hap_E1	ATTTTATTTTTTAACCTAACTCCCCTACTAAGTGTACCCCCCCTTTCCCCCCCAGGGGGGGTATACTATGCATAATCGTGCATACATTTATATACCACATATATTATGGT<br>ACCGGTAATATATACTATATATGTACTAAACCCATTATATGTATACGGGCATTAATCTATATTCCACATTTCTCCCAATGTCCATTCTATGCATGATCCAGGACACACTC<br>ATTCACCCTCCCCATAGACAGCTCCAAACCACTACCAAGTCACCTAACTATGAATGGTTACAGGACATAAATCTCACTCTCATGTTCTTCCCCCAACAAGTCACCTAAC<br>TATGAATGGTTACAGGACATACATTTAACTACC--<br>ATGTTCTAACCCATTTGGTTATGCTCGCCGTATCAGATGGATTTATTGATCGTCCACCTCACGAGAGATCAGCAACCCCTGCTTGTAATGTACTTCATGACCAGTCTCA<br>GGCCCATTCTTTCCCCCTACACCCCTCGCCCTACTTGCCTTCCACCGTACCTCTGGTTCCTCGGTCAGGCACATCCCATGCATAACTCCTGAACTTTCTCACTTTTCAC<br>GAAGTCATCTGTGGATTATCTTCCCCTCTTTAGTCCGTGATCGCGGCATCTTCTCTCTTCTATTGCTGTTGGTTCCTTCTCTTTTTGGGGCTTCTTCACAGGTTGCCCTT<br>CACAGTGCGGGTGCGGAGTGCTATTCAAGTGAAGCCTGGACTACACCTGCGTTGCGTCCTATCCTAGTCCTCTCGTGTCCCTCGATGAGACGGTTTGCGTGTATGGGG<br>AATCATCTTGACACTGATGCACTTTGGATCGCATTTGGTTATGGTTCTTCCACCCCCCC--<br>GGTAAATGGTGCTATTTAGTGAATGCTTGTCGGACATATTTTTATCAATTTTCACTTCCTCTATTTTCTTCACAAAACTAGGAAATTCACCACAATTTTTTCTTTGTTATT<br>TTTTAATTTTTTTTTTATTTTTTAAAAACATTTTTTAAAAAACTAAATTACATACAAACTACCGCATAAAATCCCTCAAACTATACAAACGTTTATCGTATAATATATATTC<br>ATTATTGTTTATTCTATCATTATTAGAGAAACTCCACTACCAAAACCATCATTAAAACAAAAATTTACATGCCACTTAACTCCCCTCACAAACAATCGTTATTTATATTGT<br>TAATTAGCAAACACAAAACCTGC

MT555046	DBMT555046_China_Hap_C3	ATTTTATTTTTTAACCTAACTCCCCTACTAAGTGTACCCCCCCTTTCCCCCCCAGGGGGGGTATACTATGCATAATCGTGCATACATTTATATACCACATATATTATGGT<br>ACCGGTAATATATACTATATATGTACTAAACCCATTATATGTATACGGGCATTAATCTATATTCCTAATTTCTCCCAATGTCCATTCTATGCATGATCCAGGACATCCTC<br>ATTCACCCTCCCCATAGACAGCTCCAAACCACTACCAAGTCACTCAACTATGAATGGTTGCAGGACATAAATCTCACTCTCATGCTCTTCCCCCAACAAGTCACCTAAC<br>TATGAATGGTTGCAGGACATACATTTAACTACT--<br>ATGTTCTAACCCATTTGGTTATGCTCGCCGTATCAGATGGATTTATTGATCGTCCACCTCACGAGAGATCAGCAACCCCTGCCTGTAATGTACTTCATGACCAGTCTCA<br>GGCCCATTCTTTCCCCCTACACCCCTCGCCCTACTTGCCTTCCACCGTACCTCTGGTTCCTCGGTCAGGCACATCCCATGCATAACTCCTGAACTTTCTCACTTTTCAC<br>GAAGTCATCTGTGGATTATCTTCCCCTCTTTAGTCCGTGATCGCGGCATCTTCTCTCTTCTATTGCTGTTGGTTCCTTCTCTTTTTGGGGCTTCTTCACAGGTTAGCCTT<br>CACAGTGCGGGTGCGGAGTGCTATTCAAGTGAAGCCTGGACTACACCTGCGTTGCGTCCTATCCTAGTCCTCTCGTGTCCCTCGATGAGACGGTTTGCGTGTATGGGG<br>AATCATCTTGACACTGATGCACTTTGGATCGCATTTGGTTATGGTTCTTCCACCCCCCC--<br>GGTAAATGGTGCTATTTAGTGAATGCTTGTCGGACATATTTTTATCAATTTTCACTTCCTCTATTTTCTTCACAAAACTAGGAAATTCACCACAATTTTTTCTTTGTTATT<br>TTTTAATTTTTTTTTTATTTTTTAAAAACATTTTTTAAAAAACTAAATTACATACAAACTACCGCATAAAATCCCTCAAACTATACAAACGTTTATCGTATAATATATATAC<br>ATTATTGTTTATTCTATCATTATTAGAGAAACTCCACTACCAAAACCATCATTAAAACAAAAATTTACATGCCACTTAACTCCCCTCACAAACAATCGTTATTTATATTGT<br>TAATTAGCAAACACAAAACCCGC
MT555047	DBMT555047_China_Hap_C3	ATTTTATTTTTTAACCTAACTCCCCTACTAAGTGTACCCCCCCTTTCCCCCCCAGGGGGGGTATACTATGCATAATCGTGCATACATTTATATACCACATATATTATGGT<br>ACCGGTAATATATACTATATATGTACTAAACCCATTATATGTATACGGGCATTAATCTATATTCCTAATTTCTCCCAATGTCCATTCTATGCATGATCCAGGACATCCTC<br>ATTCACCCTCCCCATAGACAGCTCCAAACCACTACCAAGTCACTCAACTATGAATGGTTGCAGGACATAAATCTCACTCTCATGCTCTTCCCCCAACAAGTCACCTAAC<br>TATGAATGGTTGCAGGACATACATTTAACTACT--<br>ATGTTCTAACCCATTTGGTTATGCTCGCCGTATCAGATGGATTTATTGATCGTCCACCTCACGAGAGATCAGCAACCCCTGCCTGTAATGTACTTCATGACCAGTCTCA<br>GGCCCATTCTTTCCCCCTACACCCCTCGCCCTACTTGCCTTCCACCGTACCTCTGGTTCCTCGGTCAGGCACATCCCATGCATAACTCCTGAACTTTCTCACTTTTCAC<br>GAAGTCATCTGTGGATTATCTTCCCCTCTTTAGTCCGTGATCGCGGCATCTTCTCTCTTCTATTGCTGTTGGTTCCTTCTCTTTTTGGGGCTTCTTCACAGGTTAGCCTT<br>CACAGTGCGGGTGCGGAGTGCTATTCAAGTGAAGCCTGGACTACACCTGCGTTGCGTCCTATCCTAGTCCTCTCGTGTCCCTCGATGAGACGGTTTGCGTGTATGGGG<br>AATCATCTTGACACTGATGCACTTTGGATCGCATTTGGTTATGGTTCTTCCACCCCCCC--<br>GGTAAATGGTGCTATTTAGTGAATGCTTGTCGGACATATTTTTATCAATTTTCACTTCCTCTATTTTCTTCACAAAACTAGGAAATTCACCACAATTTTTTCTTTGTTATT<br>TTTTAATTTTTTTTTTATTTTTTAAAAACATTTTTTAAAAAACTAAATTACATACAAACTACCGCATAAAATCCCTCAAACTATACAAACGTTTATCGTATAATATATATAC<br>ATTATTGTTTATTCTATCATTATTAGAGAAACTCCACTACCAAAACCATCATTAAAACAAAAATTTACATGCCACTTAACTCCCCTCACAAACAATCGTTATTTATATTGT<br>TAATTAGCAAACACAAAACCCGC
MT555048	DBMT555048_China_Hap_C3	ATTTTATTTTTTAACCTAACTCCCCTACTAAGTGTACCCCCCCTTTCCCCCCCAGGGGGGGTATACTATGCATAATCGTGCATACATTTATATACCACATATATTATGGT<br>ACCGGTAATATATACTATATATGTACTAAACCCATTATATGTATACGGGCATTAATCTATATTCCTAATTTCTCCCAATGTCCATTCTATGCATGATCCAGGACATCCTC<br>ATTCACCCTCCCCATAGACAGCTCCAAACCACTACCAAGTCACTCAACTATGAATGGTTGCAGGACATAAATCTCACTCTCATGCTCTTCCCCCAACAAGTCACCTAAC<br>TATGAATGGTTGCAGGACATACATTTAACTACT--<br>ATGTTCTAACCCATTTGGTTATGCTCGCCGTATCAGATGGATTTATTGATCGTCCACCTCACGAGAGATCAGCAACCCCTGCCTGTAATGTACTTCATGACCAGTCTCA<br>GGCCCATTCTTTCCCCCTACACCCCTCGCCCTACTTGCCTTCCACCGTACCTCTGGTTCCTCGGTCAGGCACATCCCATGCATAACTCCTGAACTTTCTCACTTTTCAC<br>GAAGTCATCTGTGGATTATCTTCCCCTCTTTAGTCCGTGATCGCGGCATCTTCTCTCTTCTATTGCTGTTGGTTCCTTCTCTTTTTGGGGCTTCTTCACAGGTTAGCCTT<br>CACAGTGCGGGTGCGGAGTGCTATTCAAGTGAAGCCTGGACTACACCTGCGTTGCGTCCTATCCTAGTCCTCTCGTGTCCCTCGATGAGACGGTTTGCGTGTATGGGG<br>AATCATCTTGACACTGATGCACTTTGGATCGCATTTGGTTATGGTTCTTCCACCCCCCC--<br>GGTAAATGGTGCTATTTAGTGAATGCTTGTCGGACATATTTTTATCAATTTTCACTTCCTCTATTTTCTTCACAAAACTAGGAAATTCACCACAATTTTTTCTTTGTTATT<br>TTTTAATTTTTTTTTTATTTTTTAAAAACATTTTTTAAAAAACTAAATTACATACAAACTACCGCATAAAATCCCTCAAACTATACAAACGTTTATCGTATAATATATATAC<br>ATTATTGTTTATTCTATCATTATTAGAGAAACTCCACTACCAAAACCATCATTAAAACAAAAATTTACATGCCACTTAACTCCCCTCACAAACAATCGTTATTTATATTGT<br>TAATTAGCAAACACAAAACCCGC

MT555049	DBMT555049_China_Hap_C3	ATTTTATTTTTTAACCTAACTCCCCTACTAAGTGTACCCCCCCTTTCCCCCCCAGGGGGGGTATACTATGCATAATCGTGCATACATTTATATACCACATATATTATGGT<br>ACCGGTAATATATACTATATATGTACTAAACCCATTATATGTATACGGGCATTAATCTATATTCCTAATTTCTCCCAATGTCCATTCTATGCATGATCCAGGACATACTC<br>ATTCACCCTCCCCATAGACAGCTCCAAACCACTACCAAGTCACTCAACTATGAATGGTTGCAGGACATAAATCTCACTCTCATGCTCTTCCCCCAACAAGTCACCTAAC<br>TATGAATGGTTGCAGGACATACATTTAACTACT--<br>ATGTTCTAACCCATTTGGTTATGCTCGCCGTATCAGATGGATTTATTGATCGTCCACCTCACGAGAGATCAGCAACCCCTGCCTGTAATGTACTTCATGACCAGTCTCA<br>GGCCCATTCTTTCCCCCTACACCCCTCGCCCTACTTGCCTTCCACCGTACCTCTGGTTCCTCGGTCAGGCACATCCCATGCATAACTCCTGAACTTTCTCACTTTTCAC<br>GAAGTCATCTGTGGATTATCTTCCCCTCTTTAGTCCGTGATCGCGGCATCTTCTCTCTTCTATTGCTGTTGGTTCCTTCTCTTTTTGGGGCTTCTTCACAGGTTACCCTT<br>CACAGTGCGGGTGCGGAGTGCTATTCAAGTGAAGCCTGGACTACACCTGCGTTGCGTCCTATCCTAGTCCTCTCGTGTCCCTCGATGAGACGGTTTGCGTGTATGGGG<br>AATCATCTTGACACTGATGCACTTTGGATCGCATTTGGTTATGGTTCTTCCACCCCCCC--<br>GGTAAATGGTGCTATTTAGTGAATGCTTGTCGGACATATTTTTATCAATTTTCACTTCCTCTATTTTCTTCACAAAACTAGGAAATTCACCACAATTTTTTCTTTGTTATT<br>TTTTAATTTTTTTTTTATTTTTTAAAAACATTTTTTAAAAAACTAAATTACATACAAACTACCGCATAAAATCCCTCAAACTATACAAACGTTTATCGTATAATATATATAC<br>ATTATTGTTTATTCTATCATTATTAGAGAAACTCCACTACCAAAACCATCATTAAAACAAAAATTTACATGCCACTTAACTCCCCTCACAAACAATCGTTATTTATATTGT<br>TAATTAGCAAACACAAAACCCGC
NC040902	DBNC040902_Myanmar_Hap_C3	ATTTTATTTTTTAACCTAACTCCCCTACTAAGTGTACCCCCCCTTTCCCCCCCAGGGGGGGTATACTATGCATAATCGTGCATACATTTATATACCACATATATTATGGT<br>ACCGGTAATATATACTATATATGTACTAAACCCATTATATGTATACGGGCATTAATCTATATTCCTCATTTCTCCCAATGTCCATTCTATGCATGATCCAGGACATACTC<br>ATTCACCCTCCCCATAGACAGCTCCAAACCACTACCAAGTCACTTAACTATGAATGGTTGCAGGACATAAATCTCACTCTCATGCTCTTCCCCCAACAAGTCACCTAAC<br>TATGAATGGTTGCAGGACATACATTTAACTACT--<br>ATGTTCTAACCCATTTGGTTATGCTCGCCGTATCAGATGGATTTATTGATCGTCCACCTCACGAGAGATCAGCAACCCCTGCCTGTAATGTACTTCATGACCAGTCTCA<br>GGCCCATTCTTTCCCCCTACACCCCTCGCCCTACTTGCCTTCCACCGTACCTCTGGTTCCTCGGTCAGGCACATCCCATGCATAACTCCTGAACTTTCTCACTTTTCAC<br>GAAGTCATCTGTGGATTATCTTCCCCTCTTTAGTCCGTGATCGCGGCATCTTCTCTCTTCTATTGCTGTTGGTTCCTTCTCTTTTTGGGGCTTCTTCACAGGTTACCCTT<br>CACAGTGCGGGTGCGGAGTGCTATTCAAGTGAAGCCTGGACTACACCTGCGTTGCGTCCTATCCTAGTCCTCTCGTGTCCCTCGATGAGACGGTTTGCGTGTATGGGG<br>AATCATCTTGACACTGATGCACTTTGGATCGCATTTGGTTATGGTTCTTCCACCCCCCC--<br>GGTAAATGGTGCTATTTAGTGAATGCTTGTCGGACATATTTTTATCAATTTTCACTTCCTCTATTTTCTTCACAAAACTAGGAAATTCACCACAATTTTTTCTTTGTTATT<br>TTTTAATTTTTTTTTTATTTTTTAAAAACATTTTTTAAAAAACTAAATTACATACAAACTACCGCATAAAATCCCTCAAACTATACAAACGTTTATCGTATAATATATATAC<br>ATTATTGTTTATTCTATCATTATTAGAGAAACTCCACTACCAAAACCATCATTAAAACAAAAATTTACATGCCACTTAACTCCCCTCACAAACAATCGTTATTTATATTGT<br>TAATTAGCAAACACAAAACCCGC
NC040970	DBNC040970_China_Hap_E1	ATTTTATTTTTTAACCTAACTCCCCTACTAAGTGTACCCCCCCTTTCCCCCCCAGGGGGGGTATACTATGCATAATCGTGCATACATTTATATACCACATATATTATGGT<br>ACCGGTAATATATACTATATATGTACTAAACCCATTATATGTATACGGGCATTAATCTATATTCCACATTTCTCCCAATGTCCATTCTATGCATGATCCAGGACACACTC<br>ATTCACCCTCCCCATAGACAGCTCCAAACCACTACCAAGTCACCTAACTATGAATGGTTACAGGACATAAATCTCACTCTCATGTTCTTCCCCCAACAAGTCACCTAAC<br>TATGAATGGTTACAGGACATACATTTAACTACC--<br>ATGTTCTAACCCATTTGGTTATGCTCGCCGTATCAGATGGATTTATTGATCGTCCACCTCACGAGAGATCAGCAACCCCTGCTTGTAATGTACTTCATGACCAGTCTCA<br>GGCCCATTCTTTCCCCCTACACCCCTCGCCCTACTTGCCTTCCACCGTACCTCTGGTTCCTCGGTCAGGCACATCCCATGCATAACTCCTGAACTTTCTCACTTTTCAC<br>GAAGTCATCTGTGGATTATCTTCCCCTCTTTAGTCCGTGATCGCGGCATCTTCTCTCTTCTATTGCTGTTGGTTCCTTCTCTTTTTGGGGCTTCTTCACAGGTTGCCCTT<br>CACAGTGCGGGTGCGGAGTGCTATTCAAGTGAAGCCTGGACTACACCTGCGTTGCGTCCTATCCTAGTCCTCTCGTGTCCCTCGATGAGACGGTTTGCGTGTATGGGG<br>AATCATCTTGACACTGATGCACTTTGGATCGCATTTGGTTATGGTTCTTCCACCCCCCC--<br>GGTAAATGGTGCTATTTAGTGAATGCTTGTCGGACATATTTTTATCAATTTTCACTTCCTCTATTTTCTTCACAAAACTAGGAAATTCACCACAATTTTTTCTTTGTTATT<br>TTTTAATTTTTTTTTTATTTTTTAAAAACATTTTTTAAAAAACTAAATTACATACAAACTACCGCATAAAATCCCTCAAACTATACAAACGTTTATCGTATAATATATATAC<br>ATTATTGTTTATTCTATCATTATTAGAGAAACTCCACTACCAAAACCATCATTAAAACAAAAATTTACATGCCACTTAACTCCCCTCACAAACAATCGTTATTTATATTGT<br>TAATTAGCAAACACAAAACCTGC

NC007239	DBNC007239 (G_lafayetii)	ATTTTATTTTTTAACCTAACTCCCCTACTAAGTGTACCCCCCCTTTCCCCCCCAGGGGGGGTATACTATGCATAATCGTGCATACATTTATATACCACATATATTATGGT<br>ACCGGTAATATATACTATATATGTACTAAACCCATTATATGTATACGGGCATTAATCTACATTCCCCATTTCTCCCAATGTCCATCTCATGAATGGTCCAGGTCAATCCT<br>ATCTATCCTACCCATACATAA–<br>TCTTTACCACTAACAAGTCACCTAACTATGAATGGTTACAGGACATACATCTAACCTTAATGCTCTTCCTCTAACAAGCCACCTAACTATGAATGGTTACAGGACATAC<br>ATCTAACCTTAATGCTCTTCCCCTGACAGGTCACCTAACTATGAATGGTTACAGGACATACCCTCCAATCCTTATGTTCTAACCCATTTGGTTATGCTCGTCGTATCAG<br>ATGGATTTATTGATCGTTCACCTCACGAGAGATCAGCAACCCCTGCCTGTAATGTACTTCATGACCAGTCTCAGGCCCATTCTTTCCCCCTACACCCCTCGCCCTACTT<br>GCCTTCCACCGTGCCTCTGGTTCCTCGGTCAGGCACATCCCATGCATAACTCCTGAACTTTCTCACTTTTCACGAAGTCATCTGTGGATTATCTTCCCCTTTTTAGTCC<br>GTGATCGCGGCATCTTCTCTCTTCTATTGCTGTTGGTTCCTTTTCTCTTTGGGGCTTCTTCACAGGTTGCCCTTCACAGTGCGGGTGCGGAGTGCTATTCAAGTGAAGC<br>CTGGACTACACCTGCGTTGCGTCCTATCCTAGTCCTCTCGTGTCCCTCGATGAGACGGTTTGCGTGTATGGGGAATCATCTTGACACTGATGCACTTTGGATCGCATTT<br>GGTTATGGTTCTTCCACCCCCCCC––<br>GGTAAATGGTGCTATTTAGTGAATGCTTGTCGGACATATTTTTATCAATTTTCACTTCCTCTATTTTCTTCACAAAACTAGGAAATTCACCACAATTTTTTCTTTGGTTTT<br>TTTTAATTTTTATTTCATTTTTTAAAAACATTTTTTAAAAAACTAAATTATAAATAAACTACCGCATAAAATCCCTCAAACTATACAAACGTTTATCGTATAGTATATATAC<br>ATTATTGTTTGTTTCATCATTATTAGAGAAACTCCACTACCAAAACCATCATTAAAACAAAAATTTACACACCACTTAACTTCCCCTACAAACAATCGTTATTTATATTGT<br>TAATTAGCAAACACAAAACCTGC

Supplementary Table S4. List of mitochondrial DNA D-loop halotypes found in Cambodian indigenous chickens and commercial chicken breeds

Types of chickens	Province or municipality	Population No.	Village / Commune / District	Collection date	ID	Sex[1)]	Haplotype	Haplogroup	Accession No.
Indigeous chicken	Oddar Meanchey	Pop 1	Doun Kaen village, Sangkat Samraong commune, Krong Samraong district	13-Mar-20	RT650	F	CamHap_07	D	LC629779
					RT651	F	CamHap_02	D	LC629780
					RT652	F	CamHap_05	A	LC629781
					RT653	F	CamHap_06	J	LC629782
					RT654	F	CamHap_09	B	LC629783
					RT655	F	CamHap_10	A	LC629784
					RT656	F	CamHap_08	B	LC629785
					RT657	F	CamHap_11	A	LC629786
					RT658	F	CamHap_09	B	LC629787
					RT659	F	CamHap_04	D	LC629788
					RT660	F	CamHap_01	B	LC629789
					RT661	F	CamHap_02	D	LC629790
					RT662	F	CamHap_03	B	LC629791
					RT663	F	CamHap_03	B	LC629792
					RT664	F	CamHap_04	D	LC629793
					RT665	F	CamHap_05	A	LC629794
					RT666	F	CamHap_05	A	LC629795
					RT667	F	CamHap_05	A	LC629796
					RT668	F	CamHap_07	D	LC629797
					RT669	F	CamHap_06	J	LC629798
					RT670	F	CamHap_02	D	LC629799
					RT671	F	CamHap_08	B	LC629800
					RT672	F	CamHap_07	D	LC629801
					RT673	F	CamHap_02	D	LC629802
					RT674	F	CamHap_02	D	LC629803
	Banteay Meanchey	Pop 2	Klengpor, Sla Kram, Svay Chek	19-Nov-19	RT366	F	CamHap_12	B	LC629804
					RT367	F	CamHap_02	D	LC629805
					RT368	F	CamHap_08	B	LC629806
					RT370	F	CamHap_08	B	LC629807
					RT371	F	CamHap_08	B	LC629808
					RT372	F	CamHap_13	D	LC629809
					RT373	M	CamHap_08	B	LC629810
					RT374	F	CamHap_08	B	LC629811
					RT375	F	CamHap_14	B	LC629812
					RT376	F	CamHap_15	B	LC629813
					RT377	F	CamHap_02	D	LC629814
					RT378	F	CamHap_08	B	LC629815
					RT379	M	CamHap_08	B	LC629816
					RT380	F	CamHap_08	B	LC629817
					RT381	F	CamHap_08	B	LC629818

				RT382	F	CamHap_14	B	LC629819
				RT383	F	CamHap_08	B	LC629820
				RT384	F	CamHap_08	B	LC629821
				RT385	F	CamHap_02	D	LC629822
				RT386	F	CamHap_08	B	LC629823
				RT387	F	CamHap_02	D	LC629824
				RT388	F	CamHap_16	A	LC629825
Siem Reap	Pop 3	Preshdak, Preah Dak, Banteay Srei	21-Nov-19	RT389	M	CamHap_17	J	LC629826
				RT390	F	CamHap_08	B	LC629827
				RT391	M	CamHap_08	B	LC629828
				RT392	M	CamHap_16	A	LC629829
				RT393	F	CamHap_18	D	LC629830
				RT394	F	CamHap_18	D	LC629831
				RT395	F	CamHap_08	B	LC629832
				RT396	F	CamHap_19	J	LC629833
				RT397	F	CamHap_16	A	LC629834
				RT398	F	CamHap_18	D	LC629835
				RT399	F	CamHap_18	D	LC629836
				RT400	F	CamHap_18	D	LC629837
				RT401	F	CamHap_20	B	LC629838
				RT402	F	CamHap_16	A	LC629839
				RT403	F	CamHap_16	A	LC629840
				RT404	F	CamHap_18	D	LC629841
				RT405	M	CamHap_19	J	LC629842
				RT406	M	CamHap_19	J	LC629843
				RT407	F	CamHap_16	A	LC629844
				RT408	F	CamHap_18	D	LC629845
				RT409	F	CamHap_16	A	LC629846
				RT410	F	CamHap_21	D	LC629847
				RT411	F	CamHap_21	D	LC629848
Preah Vihear	Pop 4-1	Kandal, Sangkat Kampong Pranak, Krong Preah Vihear	14-Mar-20	RT676	F	CamHap_17	J	LC629849
				RT677	F	CamHap_02	D	LC629850
				RT678	F	CamHap_17	J	LC629851
				RT679	M	CamHap_08	B	LC629852
				RT680	F	CamHap_02	D	LC629853
				RT681	M	CamHap_18	D	LC629854
				RT682	M	CamHap_18	D	LC629855
				RT684	M	CamHap_22	D	LC629856
				RT685	M	CamHap_23	A	LC629857
				RT686	M	CamHap_18	D	LC629858
				RT687	F	CamHap_18	D	LC629859
	Pop 4-2	Stapo, Sangkat Pal Hal, Krong Preah Vihear	14-Mar-20	RT688	M	CamHap_24	D	LC629860

				RT689	F	CamHap_23	A	LC629861
				RT690	F	CamHap_08	B	LC629862
				RT692	F	CamHap_08	B	LC629863
				RT693	F	CamHap_18	D	LC629864
				RT694	F	CamHap_23	A	LC629865
				RT695	F	CamHap_19	J	LC629866
				RT696	F	CamHap_02	D	LC629867
				RT697	F	CamHap_25	D	LC629868
				RT698	F	CamHap_18	D	LC629869
				RT699	F	CamHap_19	J	LC629870
Pailin	Pop 5	Toulslorlaov, Sangkat Toul Lvea, Krong Pailin	22-Sep-20	RT758	F	CamHap_18	D	LC629871
				RT759	F	CamHap_18	D	LC629872
				RT760	F	CamHap_18	D	LC629873
				RT761	F	CamHap_18	D	LC629874
				RT762	F	CamHap_26	D	LC629875
				RT763	F	CamHap_18	D	LC629876
				RT764	F	CamHap_18	D	LC629877
				RT765	F	CamHap_18	D	LC629878
				RT766	F	CamHap_18	D	LC629879
				RT767	F	CamHap_19	J	LC629880
				RT768	M	CamHap_02	D	LC629881
				RT769	F	CamHap_02	D	LC629882
				RT770	M	CamHap_27	A	LC629883
				RT771	M	CamHap_28	A	LC629884
				RT774	F	CamHap_18	D	LC629885
				RT775	F	CamHap_29	D	LC629886
				RT777	F	CamHap_30	B	LC629887
				RT778	F	CamHap_31	D	LC629888
				RT779	M	CamHap_32	A	LC629889
				RT780	M	CamHap_31	D	LC629890
				RT781	F	CamHap_06	J	LC629891
				RT782	F	CamHap_24	D	LC629892
Battambang	Pop 6	Toul Ta Aek, Sangkat Toul Ta Aek, Krong Battambang	23-Sep-20	RT783	F	CamHap_30	B	LC629893
				RT784	F	CamHap_30	B	LC629894
				RT785	F	CamHap_32	A	LC629895
				RT786	F	CamHap_26	D	LC629896
				RT787	F	CamHap_33	E	LC629897
				RT788	F	CamHap_34	B	LC629898
				RT789	F	CamHap_34	B	LC629899
				RT790	F	CamHap_30	B	LC629900
				RT791	F	CamHap_30	B	LC629901
				RT792	F	CamHap_34	B	LC629902

				RT793	F	CamHap_26	D	LC629903
				RT794	F	CamHap_30	B	LC629904
				RT795	F	CamHap_33	E	LC629905
				RT796	F	CamHap_35	B	LC629906
				RT797	F	CamHap_35	B	LC629907
				RT798	F	CamHap_35	B	LC629908
				RT799	F	CamHap_30	B	LC629909
				RT800	F	CamHap_30	B	LC629910
				RT801	F	CamHap_11	A	LC629911
				RT802	F	CamHap_11	A	LC629912
				RT803	F	CamHap_33	E	LC629913
				RT804	F	CamHap_30	B	LC629914
				RT805	F	CamHap_30	B	LC629915
				RT806	F	CamHap_36	E	LC629916
				RT807	F	CamHap_36	E	LC629917
Pursat	Pop 7-1	Chamkar Ou, Trapeang Chorng, Bakan	7-Jun-19	RT168	F	CamHap_06	J	LC629918
				RT169	F	CamHap_18	D	LC629919
				RT170	F	CamHap_06	J	LC629920
				RT171	M	CamHap_06	J	LC629921
				RT172	F	CamHap_37	A	LC629922
				RT173	F	CamHap_06	J	LC629923
				RT174	F	CamHap_37	A	LC629924
				RT176	M	CamHap_18	D	LC629925
				RT177	F	CamHap_06	J	LC629926
				RT178	F	CamHap_37	A	LC629927
				RT179	F	CamHap_18	D	LC629928
				RT180	F	CamHap_06	J	LC629929
				RT181	M	CamHap_06	J	LC629930
				RT182	F	CamHap_18	D	LC629931
				RT183	F	CamHap_06	J	LC629932
				RT184	M	CamHap_06	J	LC629933
				RT185	M	CamHap_37	A	LC629934
				RT186	M	CamHap_37	A	LC629935
	Pop 7-2	Krobaochrum, Bak Chenhchien, Phnum Kravanh	30-Dec-20	RT808	F	CamHap_30	B	LC629936
				RT810	F	CamHap_30	B	LC629937
				RT811	F	CamHap_18	D	LC629938
				RT812	F	CamHap_18	D	LC629939
				RT813	F	CamHap_38	D	LC629940
				RT814	F	CamHap_06	J	LC629941
				RT815	F	CamHap_05	A	LC629942
				RT816	F	CamHap_38	D	LC629943
				RT819	F	CamHap_18	D	LC629944
				RT821	F	CamHap_18	D	LC629945

				RT822	F	CamHap_39	B	LC629946
				RT824	F	CamHap_40	D	LC629947
				RT826	F	CamHap_06	J	LC629948
				RT827	F	CamHap_30	B	LC629949
				RT828	F	CamHap_06	J	LC629950
				RT829	M	CamHap_40	D	LC629951
				RT830	F	CamHap_06	J	LC629952
				RT831	F	CamHap_06	J	LC629953
				RT832	F	CamHap_18	D	LC629954
	Pop 7-3	Pramaoy, Pramaoy, Veal Veaeng	31-Dec-20	RT835	F	CamHap_33	E	LC629955
				RT836	F	CamHap_18	D	LC629956
				RT837	F	CamHap_11	A	LC629957
				RT838	F	CamHap_11	A	LC629958
				RT839	F	CamHap_38	D	LC629959
				RT840	F	CamHap_11	A	LC629960
				RT841	F	CamHap_41	B	LC629961
				RT842	F	CamHap_11	A	LC629962
				RT844	F	CamHap_06	J	LC629963
				RT845	F	CamHap_33	E	LC629964
				RT846	F	CamHap_33	E	LC629965
				RT847	F	CamHap_42	D	LC629966
				RT848	F	CamHap_18	D	LC629967
				RT849	F	CamHap_11	A	LC629968
				RT850	F	CamHap_06	J	LC629969
				RT851	F	CamHap_38	D	LC629970
				RT852	F	CamHap_18	D	LC629971
				RT853	F	CamHap_33	E	LC629972
				RT854	F	CamHap_43	D	LC629973
				RT855	F	CamHap_41	B	LC629974
Kampong Thom	Pop 8	Preybanlek, Sangkat Achar Leak, Krong Steungsen	28-Aug-19	RT197	F	CamHap_44	A	LC629975
				RT198	F	CamHap_18	D	LC629976
				RT199	M	CamHap_09	B	LC629977
				RT200	M	CamHap_05	A	LC629978
				RT201	F	CamHap_44	A	LC629979
				RT202	F	CamHap_18	D	LC629980
				RT203	F	CamHap_08	B	LC629981
				RT205	F	CamHap_44	A	LC629982
				RT206	F	CamHap_44	A	LC629983
				RT208	F	CamHap_33	E	LC629984
				RT209	F	CamHap_33	E	LC629985
				RT210	F	CamHap_45	D	LC629986
				RT211	M	CamHap_18	D	LC629987
				RT212	M	CamHap_18	D	LC629988

				RT214	M	CamHap_46	A	LC629989
				RT217	F	CamHap_33	E	LC629990
				RT218	F	CamHap_18	D	LC629991
				RT219	F	CamHap_09	B	LC629992
				RT220	F	CamHap_44	A	LC629993
				RT221	M	CamHap_06	J	LC629994
Kampong Chhnang	Pop 9	Troping Sbaov, Srae Thmei, Rolea B'ier	10-Oct-19	RT332	F	CamHap_19	J	LC629995
				RT334	F	CamHap_18	D	LC629996
				RT335	F	CamHap_47	D	LC629997
				RT336	F	CamHap_19	J	LC629998
				RT338	F	CamHap_18	D	LC629999
				RT339	M	CamHap_11	A	LC630000
				RT340	M	CamHap_48	D	LC630001
				RT341	F	CamHap_49	C	LC630002
				RT342	F	CamHap_18	D	LC630003
				RT343	M	CamHap_02	D	LC630004
				RT344	F	CamHap_19	J	LC630005
				RT345	F	CamHap_18	D	LC630006
				RT346	F	CamHap_50	A	LC630007
				RT347	M	CamHap_08	B	LC630008
				RT348	F	CamHap_02	D	LC630009
				RT349	M	CamHap_51	E	LC630010
				RT350	M	CamHap_02	D	LC630011
				RT351	F	CamHap_02	D	LC630012
				RT353	F	CamHap_52	D	LC630013
				RT354	F	CamHap_26	D	LC630014
				RT355	F	CamHap_53	A	LC630015
				RT356	F	CamHap_47	D	LC630016
Kampong Cham	Pop 10	Kaohdach, Kaoh Mitt, Kampong Siem	20-Jan-20	RT546	F	CamHap_06	J	LC630017
				RT547	F	CamHap_54	D	LC630018
				RT548	F	CamHap_06	J	LC630019
				RT549	M	CamHap_55	A	LC630020
				RT550	F	CamHap_01	B	LC630021
				RT551	F	CamHap_06	J	LC630022
				RT552	F	CamHap_06	J	LC630023
				RT554	M	CamHap_56	D	LC630024
				RT555	F	CamHap_11	A	LC630025
				RT556	F	CamHap_39	B	LC630026
				RT557	F	CamHap_24	D	LC630027
				RT558	F	CamHap_01	B	LC630028
				RT559	M	CamHap_05	A	LC630029
				RT560	M	CamHap_57	D	LC630030
				RT561	M	CamHap_05	A	LC630031

				RT562	F	CamHap_18	D	LC630032
				RT563	F	CamHap_39	B	LC630033
				RT564	F	CamHap_58	D	LC630034
				RT565	M	CamHap_05	A	LC630035
				RT566	F	CamHap_18	D	LC630036
				RT567	F	CamHap_05	A	LC630037
				RT568	F	CamHap_05	A	LC630038
				RT569	F	CamHap_06	J	LC630039
				RT570	F	CamHap_59	A	LC630040
Tboung Khmum	Pop 11	Chamkar Kor, Chak, Ou Reang Ov	19-Jan-20	RT521	F	CamHap_60	D	LC630041
				RT522	F	CamHap_60	D	LC630042
				RT523	F	CamHap_60	D	LC630043
				RT524	F	CamHap_60	D	LC630044
				RT525	F	CamHap_60	D	LC630045
				RT526	F	CamHap_60	D	LC630046
				RT527	F	CamHap_11	A	LC630047
				RT528	F	CamHap_56	D	LC630048
				RT529	F	CamHap_18	D	LC630049
				RT530	F	CamHap_01	B	LC630050
				RT531	F	CamHap_18	D	LC630051
				RT532	F	CamHap_18	D	LC630052
				RT533	F	CamHap_18	D	LC630053
				RT534	F	CamHap_18	D	LC630054
				RT535	M	CamHap_24	D	LC630055
				RT536	F	CamHap_61	D	LC630056
				RT537	F	CamHap_11	A	LC630057
				RT538	F	CamHap_24	D	LC630058
				RT539	M	CamHap_11	A	LC630059
				RT540	F	CamHap_11	A	LC630060
				RT541	F	CamHap_11	A	LC630061
				RT542	F	CamHap_11	A	LC630062
				RT543	F	CamHap_11	A	LC630063
				RT544	F	CamHap_11	A	LC630064
				RT545	F	CamHap_11	A	LC630065
Koh Kong	Pop 12	Toul Kokir Leu, Toul Kokir, Mondol Seima	14-Jan-20	RT471	F	CamHap_62	D	LC630066
				RT472	F	CamHap_18	D	LC630067
				RT473	F	CamHap_63	D	LC630068
				RT474	F	CamHap_06	J	LC630069
				RT475	F	CamHap_62	D	LC630070
				RT476	F	CamHap_06	J	LC630071
				RT477	F	CamHap_06	J	LC630072
				RT478	F	CamHap_06	J	LC630073
				RT479	F	CamHap_06	J	LC630074

				RT481	F	CamHap_62	D	LC630075
				RT482	F	CamHap_62	D	LC630076
				RT483	F	CamHap_62	D	LC630077
				RT484	F	CamHap_06	J	LC630078
				RT485	F	CamHap_64	C	LC630079
				RT486	F	CamHap_64	C	LC630080
				RT489	F	CamHap_62	D	LC630081
				RT490	F	CamHap_64	C	LC630082
				RT491	F	CamHap_64	C	LC630083
				RT492	F	CamHap_11	A	LC630084
				RT493	F	CamHap_06	J	LC630085
				RT494	F	CamHap_18	D	LC630086
Kampong Speu	Pop 13-1	Chamkadoung, Sangkat Chbar Mon, Krong Chbar Mon	25-Sep-19	RT232	M	CamHap_24	D	LC630087
				RT233	F	CamHap_65	D	LC630088
				RT234	F	CamHap_65	D	LC630089
				RT235	M	CamHap_18	D	LC630090
				RT236	M	CamHap_18	D	LC630091
				RT237	F	CamHap_26	D	LC630092
				RT238	F	CamHap_11	A	LC630093
				RT239	M	CamHap_26	D	LC630094
				RT240	F	CamHap_65	D	LC630095
				RT241	M	CamHap_51	E	LC630096
				RT242	F	CamHap_65	D	LC630097
	Pop 13-2	Pungro, Sangkat Kandaol Dom, Krong Chbar Mon	25-Sep-19	RT243	F	CamHap_66	F	LC630098
				RT244	F	CamHap_66	F	LC630099
				RT245	F	CamHap_19	J	LC630100
				RT246	F	CamHap_50	A	LC630101
				RT247	F	CamHap_06	J	LC630102
				RT248	M	CamHap_66	F	LC630103
				RT249	M	CamHap_11	A	LC630104
				RT250	F	CamHap_66	F	LC630105
				RT251	M	CamHap_18	D	LC630106
				RT252	M	CamHap_18	D	LC630107
				RT253	F	CamHap_11	A	LC630108
				RT254	F	CamHap_66	F	LC630109
				RT255	F	CamHap_18	D	LC630110
Kandal	Pop 14	Ta Kdol, Sangkat Ta Kdol, Krong Ta Khmau	22-Jan-20	RT571	F	CamHap_18	D	LC630111
				RT572	M	CamHap_05	A	LC630112
				RT573	F	CamHap_18	D	LC630113
				RT574	F	CamHap_11	A	LC630114
				RT575	F	CamHap_67	D	LC630115

				RT576	F	CamHap_24	D	LC630116
				RT577	F	CamHap_24	D	LC630117
				RT578	F	CamHap_18	D	LC630118
				RT579	F	CamHap_53	A	LC630119
				RT580	M	CamHap_06	J	LC630120
				RT581	F	CamHap_08	B	LC630121
				RT582	F	CamHap_68	A	LC630122
				RT583	F	CamHap_02	D	LC630123
				RT584	F	CamHap_26	D	LC630124
				RT585	F	CamHap_69	D	LC630125
				RT586	F	CamHap_70	J	LC630126
				RT587	F	CamHap_69	D	LC630127
				RT588	F	CamHap_02	D	LC630128
				RT589	F	CamHap_11	A	LC630129
				RT590	F	CamHap_18	D	LC630130
				RT591	M	CamHap_11	A	LC630131
				RT592	F	CamHap_71	A	LC630132
				RT593	F	CamHap_24	D	LC630133
				RT594	F	CamHap_72	D	LC630134
				RT595	F	CamHap_11	A	LC630135
Prey Veng	Pop 15	Taket, Preah Sdach, Preah Sdach	22-Feb-20	RT625	F	CamHap_18	D	LC630136
				RT626	F	CamHap_53	A	LC630137
				RT627	F	CamHap_53	A	LC630138
				RT628	F	CamHap_73	D	LC630139
				RT629	M	CamHap_73	D	LC630140
				RT630	F	CamHap_18	D	LC630141
				RT632	F	CamHap_73	D	LC630142
				RT633	F	CamHap_53	A	LC630143
				RT635	F	CamHap_18	D	LC630144
				RT636	F	CamHap_18	D	LC630145
				RT637	F	CamHap_73	D	LC630146
				RT638	F	CamHap_18	D	LC630147
				RT639	M	CamHap_53	A	LC630148
				RT640	F	CamHap_53	A	LC630149
				RT641	F	CamHap_53	A	LC630150
				RT642	F	CamHap_53	A	LC630151
				RT643	F	CamHap_05	A	LC630152
				RT644	F	CamHap_24	D	LC630153
				RT646	F	CamHap_33	E	LC630154
				RT647	F	CamHap_06	J	LC630155
				RT648	F	CamHap_11	A	LC630156
				RT649	F	CamHap_24	D	LC630157
Svay Rieng	Pop 16	Traok, Kampong Chamlang, Svay Chrum	21-Feb-20	RT600	F	CamHap_02	D	LC630158

				RT601	F	CamHap_02	D	LC630159
				RT602	F	CamHap_02	D	LC630160
				RT603	F	CamHap_02	D	LC630161
				RT604	M	CamHap_18	D	LC630162
				RT605	F	CamHap_02	D	LC630163
				RT606	F	CamHap_06	J	LC630164
				RT607	F	CamHap_06	J	LC630165
				RT608	F	CamHap_74	J	LC630166
				RT609	F	CamHap_02	D	LC630167
				RT610	F	CamHap_02	D	LC630168
				RT611	F	CamHap_02	D	LC630169
				RT612	F	CamHap_02	D	LC630170
				RT613	F	CamHap_06	J	LC630171
				RT614	F	CamHap_06	J	LC630172
				RT615	F	CamHap_02	D	LC630173
				RT616	F	CamHap_22	D	LC630174
				RT617	F	CamHap_11	A	LC630175
				RT618	F	CamHap_75	D	LC630176
				RT619	M	CamHap_24	D	LC630177
				RT620	F	CamHap_06	J	LC630178
				RT621	F	CamHap_06	J	LC630179
				RT622	F	CamHap_06	J	LC630180
				RT623	F	CamHap_06	J	LC630181
				RT624	F	CamHap_06	J	LC630182
Preah Sihanouk	Pop 17	Troping Sruy, Andoung Thma, Prey	15-Jan-20	RT497	M	CamHap_76	A	LC630183
				RT498	F	CamHap_22	D	LC630184
				RT499	F	CamHap_76	A	LC630185
				RT500	F	CamHap_76	A	LC630186
				RT501	F	CamHap_76	A	LC630187
				RT502	F	CamHap_22	D	LC630188
				RT504	F	CamHap_68	A	LC630189
				RT505	F	CamHap_76	A	LC630190
				RT506	F	CamHap_76	A	LC630191
				RT507	M	CamHap_77	J	LC630192
				RT508	F	CamHap_09	B	LC630193
				RT509	F	CamHap_18	D	LC630194
				RT510	F	CamHap_18	D	LC630195
				RT511	F	CamHap_76	A	LC630196
				RT512	F	CamHap_68	A	LC630197
				RT513	F	CamHap_76	A	LC630198
				RT514	F	CamHap_78	D	LC630199
				RT515	F	CamHap_76	A	LC630200
				RT516	F	CamHap_76	A	LC630201
				RT517	F	CamHap_06	J	LC630202

				RT518	F	CamHap_10	A	LC630203
				RT519	F	CamHap_06	J	LC630204
				RT520	F	CamHap_79	J	LC630205
Kampot	Pop 18	Breal, Tani, Angkor Chey	2-Oct-19	RT307	F	CamHap_18	D	LC630206
				RT308	F	CamHap_53	A	LC630207
				RT309	F	CamHap_18	D	LC630208
				RT310	F	CamHap_18	D	LC630209
				RT311	F	CamHap_05	A	LC630210
				RT312	F	CamHap_24	D	LC630211
				RT313	F	CamHap_05	A	LC630212
				RT314	F	CamHap_05	A	LC630213
				RT315	F	CamHap_05	A	LC630214
				RT316	F	CamHap_80	B	LC630215
				RT317	F	CamHap_05	A	LC630216
				RT318	F	CamHap_81	A	LC630217
				RT319	F	CamHap_05	A	LC630218
				RT320	F	CamHap_05	A	LC630219
				RT322	F	CamHap_62	D	LC630220
				RT323	F	CamHap_05	A	LC630221
				RT324	F	CamHap_05	A	LC630222
				RT326	F	CamHap_05	A	LC630223
				RT327	F	CamHap_05	A	LC630224
				RT328	F	CamHap_24	D	LC630225
				RT329	F	CamHap_24	D	LC630226
				RT330	F	CamHap_05	A	LC630227
				RT331	F	CamHap_62	D	LC630228
Kep	Pop 19	Chamkachek, Pong Tuek, Damnak Chang'aeur	1-Oct-19	RT282	F	CamHap_06	J	LC630229
				RT283	F	CamHap_36	E	LC630230
				RT284	F	CamHap_04	D	LC630231
				RT285	F	CamHap_82	B	LC630232
				RT286	F	CamHap_03	B	LC630233
				RT287	F	CamHap_03	B	LC630234
				RT288	F	CamHap_04	D	LC630235
				RT289	F	CamHap_24	D	LC630236
				RT290	F	CamHap_06	J	LC630237
				RT291	F	CamHap_04	D	LC630238
				RT292	F	CamHap_03	B	LC630239
				RT293	F	CamHap_06	J	LC630240
				RT294	F	CamHap_04	D	LC630241
				RT295	F	CamHap_06	J	LC630242
				RT296	F	CamHap_04	D	LC630243
				RT297	F	CamHap_03	B	LC630244
				RT298	F	CamHap_04	D	LC630245

				RT299	F	CamHap_48	D	LC630246
				RT300	F	CamHap_03	B	LC630247
				RT301	F	CamHap_18	D	LC630248
				RT303	F	CamHap_73	D	LC630249
				RT304	F	CamHap_73	D	LC630250
				RT305	F	CamHap_18	D	LC630251
				RT306	F	CamHap_53	A	LC630252
Takeo	Pop 20	Troping Thom, Roneam, Trang	1-Oct-19	RT257	F	CamHap_18	D	LC630253
				RT258	F	CamHap_18	D	LC630254
				RT259	M	CamHap_11	A	LC630255
				RT260	F	CamHap_18	D	LC630256
				RT261	F	CamHap_18	D	LC630257
				RT262	F	CamHap_18	D	LC630258
				RT263	F	CamHap_53	A	LC630259
				RT264	F	CamHap_18	D	LC630260
				RT265	F	CamHap_18	D	LC630261
				RT266	F	CamHap_06	J	LC630262
				RT267	M	CamHap_18	D	LC630263
				RT268	F	CamHap_06	J	LC630264
				RT269	F	CamHap_06	J	LC630265
				RT270	F	CamHap_08	B	LC630266
				RT271	F	CamHap_83	D	LC630267
				RT272	F	CamHap_08	B	LC630268
				RT273	F	CamHap_02	D	LC630269
				RT274	M	CamHap_24	D	LC630270
				RT275	F	CamHap_11	A	LC630271
				RT276	M	CamHap_24	D	LC630272
				RT277	F	CamHap_06	J	LC630273
				RT278	F	CamHap_06	J	LC630274
				RT279	F	CamHap_06	J	LC630275
				RT280	F	CamHap_06	J	LC630276
				RT281	F	CamHap_24	D	LC630277
Stung Treng	Pop 21	Reacheanukhul, Sangkat Stung Treng, Stung Treng Municipality	3-Dec-19	RT441	F	CamHap_49	C	LC630278
				RT443	M	CamHap_51	E	LC630279
				RT444	F	CamHap_49	C	LC630280
				RT445	F	CamHap_84	B	LC630281
				RT446	F	CamHap_51	E	LC630282
				RT447	F	CamHap_48	D	LC630283
				RT448	F	CamHap_33	E	LC630284
				RT449	M	CamHap_33	E	LC630285
				RT450	F	CamHap_48	D	LC630286
				RT452	F	CamHap_51	E	LC630287
				RT453	M	CamHap_18	D	LC630288

				RT454	F	CamHap_09	B	LC630289
				RT455	M	CamHap_33	E	LC630290
				RT456	M	CamHap_18	D	LC630291
				RT457	F	CamHap_18	D	LC630292
				RT458	F	CamHap_18	D	LC630293
				RT459	F	CamHap_18	D	LC630294
				RT460	F	CamHap_06	J	LC630295
				RT461	F	CamHap_49	C	LC630296
				RT462	M	CamHap_36	E	LC630297
				RT463	F	CamHap_35	B	LC630298
				RT464	F	CamHap_09	B	LC630299
				RT465	M	CamHap_33	E	LC630300
Ratanakiri	Pop 22	Pruok, Ba Tang, Lumphat	24-Jul-20	RT733	F	CamHap_18	D	LC630301
				RT735	M	CamHap_18	D	LC630302
				RT736	M	CamHap_06	J	LC630303
				RT737	M	CamHap_85	J	LC630304
				RT738	M	CamHap_06	J	LC630305
				RT739	M	CamHap_06	J	LC630306
				RT741	F	CamHap_06	J	LC630307
				RT742	F	CamHap_11	A	LC630308
				RT743	F	CamHap_06	J	LC630309
				RT744	F	CamHap_06	J	LC630310
				RT745	M	CamHap_28	A	LC630311
				RT746	M	CamHap_06	J	LC630312
				RT747	F	CamHap_06	J	LC630313
				RT748	M	CamHap_11	A	LC630314
				RT749	M	CamHap_11	A	LC630315
				RT750	M	CamHap_11	A	LC630316
				RT751	F	CamHap_06	J	LC630317
Kratie	Pop 23	Dar, Dar, Chetr Borei	2-Dec-19	RT417	F	CamHap_18	D	LC630318
				RT418	F	CamHap_18	D	LC630319
				RT419	F	CamHap_51	E	LC630320
				RT421	F	CamHap_18	D	LC630321
				RT422	F	CamHap_18	D	LC630322
				RT423	F	CamHap_18	D	LC630323
				RT424	F	CamHap_56	D	LC630324
				RT425	F	CamHap_18	D	LC630325
				RT426	F	CamHap_51	E	LC630326
				RT427	F	CamHap_18	D	LC630327
				RT428	F	CamHap_18	D	LC630328
				RT429	F	CamHap_18	D	LC630329
				RT430	F	CamHap_18	D	LC630330
				RT431	F	CamHap_05	A	LC630331
				RT432	F	CamHap_86	B	LC630332

					RT433	F	CamHap_26	D	LC630333
					RT434	F	CamHap_51	E	LC630334
					RT435	F	CamHap_18	D	LC630335
					RT436	F	CamHap_18	D	LC630336
					RT437	F	CamHap_18	D	LC630337
					RT438	F	CamHap_18	D	LC630338
					RT439	F	CamHap_18	D	LC630339
					RT440	F	CamHap_18	D	LC630340
	Mondul Kiri	Pop 24	Polung, Sangkat Romonea, Senmonorom Municipality	23-Jul-20	RT709	F	CamHap_67	D	LC630341
					RT710	F	CamHap_87	D	LC630342
					RT712	F	CamHap_67	D	LC630343
					RT713	F	CamHap_67	D	LC630344
					RT714	F	CamHap_67	D	LC630345
					RT715	F	CamHap_53	A	LC630346
					RT716	F	CamHap_67	D	LC630347
					RT717	F	CamHap_24	D	LC630348
					RT718	F	CamHap_67	D	LC630349
					RT719	F	CamHap_53	A	LC630350
					RT720	M	CamHap_88	D	LC630351
					RT721	F	CamHap_89	D	LC630352
					RT722	F	CamHap_18	D	LC630353
					RT723	F	CamHap_18	D	LC630354
					RT724	F	CamHap_67	D	LC630355
					RT725	F	CamHap_18	D	LC630356
					RT726	F	CamHap_67	D	LC630357
					RT727	F	CamHap_53	A	LC630358
					RT728	F	CamHap_67	D	LC630359
					RT729	F	CamHap_24	D	LC630360
					RT730	F	CamHap_24	D	LC630361
					RT731	F	CamHap_24	D	LC630362
					RT732	F	CamHap_67	D	LC630363
Commercial chicken Three-way hybrid	Phnom Penh	Pop 25		14-Aug-19	RT187	F	CamHap_33	E	LC630364
					RT188	F	CamHap_33	E	LC630365
					RT189	F	CamHap_33	E	LC630366
				21-Aug-19	RT191	F	CamHap_01	B	LC630367
					RT192	F	CamHap_08	B	LC630368
					RT193	F	CamHap_11	A	LC630369
					RT194	F	CamHap_11	A	LC630370
					RT195	F	CamHap_26	D	LC630371
					RT196	F	CamHap_03	B	LC630372
Isa Brown	Phnom Penh	Pop 26		4-Sep-19	RT222	F	CamHap_90	E	LC630373
					RT224	F	CamHap_90	E	LC630374

					RT225	F	CamHap_91	E	LC630375
					RT226	F	CamHap_90	E	LC630376
					RT227	F	CamHap_90	E	LC630377
					RT228	F	CamHap_91	E	LC630378
					RT229	F	CamHap_90	E	LC630379
				19-Sep-19	RT230	F	CamHap_91	E	LC630380
					RT231	F	CamHap_91	E	LC630381
				30-Oct-19	RT357	F	CamHap_91	E	LC630382
					RT358	F	CamHap_90	E	LC630383
					RT359	F	CamHap_90	E	LC630384
					RT360	F	CamHap_90	E	LC630385
					RT361	F	CamHap_90	E	LC630386
					RT362	F	CamHap_90	E	LC630387
				22-Nov-19	RT413	F	CamHap_90	E	LC630388
					RT414	F	CamHap_91	E	LC630389
					RT415	F	CamHap_92	E	LC630390
				22-Jan-20	RT596	F	CamHap_33	E	LC630391
					RT597	F	CamHap_33	E	LC630392
					RT598	F	CamHap_36	E	LC630393
					RT599	F	CamHap_33	E	LC630394
				30-Apr-20	RT700	F	CamHap_91	E	LC630395
					RT701	F	CamHap_90	E	LC630396
					RT702	F	CamHap_51	E	LC630397
Rhode Island White	Kandal	Pop 27	Thum, Koki, Kien Svay	4-Nov-18	RT156	F	CamHap_51	E	LC630398
					RT157	F	CamHap_33	E	LC630399
					RT158	F	CamHap_33	E	LC630400
					RT159	F	CamHap_51	E	LC630401
					RT160	F	CamHap_33	E	LC630402
					RT161	F	CamHap_33	E	LC630403

[1)] F, female; M, male

Supplementary Table S5. Nucleotide sequences of mitochondrial DNA D-loop halotypes found in Cambodian indigenous chickens and commercial chicken breeds

Haplotype	Nucleotide sequence
CamHap_1	ATTTTATTTTTTAACCTAACTCCCCTACTAAGTGTACCCCCCCTTTCCCCCCCAGGGGGGGTATACTATGCATAATCGTGCATACATTTATATACCACATATATTATGGTACCGGTAATATATACTATATATGTACTAAACCCATTAT<br>ATGTATACGGGCATTAATCTATATTCCACATTTCTCCCAATGTCCATTCTATGCATGATCCAAGACATACTCATTCACCCTCCCCATAGACAGTTCTAAACCACTATCAAGCCACCTAACTATGAATGGTTACAGGACATAAATCTC<br>ACTCTCATGTTCTCCCCCTAACAAGTCACCTAACTATGAATGGTTACAGGACATACATTTAACTACC––<br>ATGTTCTAACCCATTTGGTTATGCTCGCCGTATCAGATGGATTTATTGATCGTCCACCTCACGAGAGATCAGCAACCCCTGCCTGTAATGTACTTCATGACCAGTCTCAGGCCCATTCTTTCCCCCTACACCCCTCGCCCTACTTG<br>CCTTCCACCGTACCTCTGGTTCCTCGGTCAGGCACATCCCATGCATAACTCCTGAACTTTCTCACTTTTCACGAAGTCATCTGTGGATTATCTTCCCCTCTTTAGTCCGTGATCGCGGCATCTTCTCTCTTCTATTGCTGTTGGTTC<br>CTTCTCTTTTTGGGGCTTCTTCACAGGTTGCCCTTCACAGTGCGGGTGCGGAGTGCTATTCAAGTGAAGCCTGGACTACACCTGCGTTGCGTCCTATCCTAGTCCTCTCGTGTCCCTCGATGAGACGGTTTGCGTATATGGGGAAT<br>CATCTTGACACTGATGCACTTTGGATCGCATTTGGTTATGGTTCTTCCACCCCCCCC–<br>GGTAAATGGTGCTATTTAGTGAATGCTTGTCGGACATATTTTTATCAATTTTCACTTCCTCTATTTTCTTCACAAAACTAGGAAATTCACCACAATTTTTTCTTTGTTATTTTTTAATTTTTTTTTTATTTTTTAAAAACATTTTTTAAA<br>AAACTAAATTACATACAAACTACCGCATAAAATCCCTCAAACTATACAAACGTTTATCGTATAATATATATACATTATTGTTTATTCTATCATTATTAGAGAAACTCCACTACCAAAACCATCATTAAAACAAAAATTTACATGCCAC<br>TTAACTCCCCTCACAAACAATCGTTATTTATATTGTTAATTAGCAAACACAAAACCCAC
CamHap_2	ATTTTATTTTTTAACCTAACTCCCCTACTAAGTGTACCCCCCCTTTCCCCCCCAGGGGGGGTATACTATGCATAATCGTGCATACATTTATATACCACATATATTATGGTACCGGTAATATATACTATATATGTACTAAACCCATTAT<br>ATGTATACGGGCATTAATCTATATTCCACATTTCTCCCAATGTCCATTCTATGCATGATCCAGGACATACTCATTCACCCTCCCCATAGACAGCTCCAAACCACTACCAAGTCACCTAACTATGAATGGTTGCAGGACATAAATCTC<br>ACTCTCATGCTCTTCCCCCAACAAGTCACCTAACTATGAATGGTTACAGGACATACATTTAACTACC––<br>ATGTTCTAACCCATTTGGTTATGCTCGCCGTATCAGATGGATTTATTGATCGTCCACCTCACGAGAGATCAGCAACCCCTGCCTGTAATGTACTTCATGACCAGTCTCAGGCCCATTCTTTCCCCCTACACCCCTCGCCCTACTTG<br>CCTTCCACCGTACCTCTGGTTCCTCGGTCAGGCACATCCCATGCATAACTCCTGAACTTTCTCACTTTTCACGAAGTCATCTGTGGATTATCTTCCCCTCTTTAGTCCGTGATCGCGGCATCTTCTCTCTTCTATTGCTGTTGGTTC<br>CTTCTCTTTTTGGGGCTTCTTCACAGGTTGCCCTTCACAGTGCGGGTGCGGAGTGCTATTCAAGTGAAGCCTGGACTACACCTGCGTTGCGTCCTATCCTAGTCCTCTCGTGTCCCTCGATGAGACGGTTTGCGTGTATGGGGAAT<br>CATCTTGACACTGATGCACTTTGGATCGCATTTGGTTATGGTTCTTCCACCCCCCCC–<br>GGTAAATGGTGCTATTTAGTGAATGCTTGTCGGACATATTTTTATCAATTTTCACTTCCTCTATTTTCTTCACAAAACTAGGAAATTCACCACAATTTTTTCTTTGTTATTTTTTAATTTTTTTTTTATTTTTTAAAAACATTTTTTAAA<br>AAACTAAATTACATACAAACTACCGCATAAAATCCCTCAAACTATACAAACGTTTATCGTATAATATATATACATTATTGTTTATTCTATCATTATTAGAGAAACTCCACTACCAAAACCATCATTAAAACAAAAATTTACATGCCAC<br>TTAACTCCCCTCACAAACAATCGTTATTTATATTGTTAATTAGCAAACACAAAACCCGC
CamHap_3	ATTTTATTTTTTAACCTAACTCCCCTACTAAGTGTACCCCCCCTTTCCCCCCCAGGGGGGGTATACTATGCATAATCGTGCATACATTTATATACCACATATATTATGGTACCGGTAATATATACTATATATGTACTAAACCCATTAT<br>ATGTATACGGGCATTAATCTATATTCCACATTTCTCCCAATGTCCATTCTATGCATGATCCAAGACATACTCATTCACCCTCCCCATAGACAGTTCTAAACCACTATCAAGTCACCTAACTATGAATGGTTACAGGACATAAATCTT<br>ACTCTCATGTTCTCCCCCTAACAAGTCACCTAACTATGAATGGTTACAGGACATACATTTAACTACC––<br>ATGTTCTAACCCATTTGGTTATGCTCGCCGTATCAGATGGATTTATTGATCGTCCACCTCACGAGAGATCAGCAACCCCTGCCTGTAATGTACTTCATGACCAGTCTCAGGCCCATTCTTTCCCCCTACACCCCTCGCCCTACTTG<br>CCTTCCACCGTACCTCTGGTTCCTCGGTCAGGCACATCCCATGCATAACTCCTGAACTTTCTCACTTTTCACGAAGTCATCTGTGGATTATCTTCCCCTCTTTAGTCCGTGATCGCGGCATCTTCTCTCTTCTATTGCTGTTGGTTC<br>CTTCTCTTTTTGGGGCTTCTTCACAGGTTGCCCTTCACAGTGCGGGTGCGGAGTGCTATTCAAGTGAAGCCTGGACTACACCTGCGTTGCGTCCTATCCTAGTCCTCTCGTGTCCCTCGATGAGACGGTTTGCGTATATGGGGAAT<br>CATCTTGACACTGATGCACTTTGGATCGCATTTGGTTATGGTTCTTCCACCCCCCCC–<br>GGTAAATGGTGCTATTTAGTGAATGCTTGTCGGACATATTTTTATCAATTTTCACTTCCTCTATTTTCTTCACAAAACTAGGAAATTCACCACAATTTTTTCTTTGTTATTTTTTAATTTTTTTTTTATTTTTTAAAAACATTTTTTAAA<br>AAACTAAATTACATACAAACTACCGCATAAAATCCCTCAAACTATACAAACGTTTATCGTATAATATATATACATTATTGTTTATTCTATCATTATTAGAGAAACTCCACTACCAAAACCATCATTAAAACAAAAATTTACATGCCAC<br>TTAACTCCCCTCACAAACAATCGTTATTTATATTGTTAATTAGCAAACACAAAACCCAC
CamHap_4	ATTTTATTTTTTAACCTAACTCCCCTACTAAGTGTACCCCCCCTTTCCCCCCCAGGGGGGGTATACTATGCATAATCGTGCATACATTTATATACCACATATATTATGGTACCGGTAATATATACTATATATGTACTAAACCCATTAT<br>ATGTATACGGGCATTAATCTATATTCCACATTTCTCCCAATGTCCATTCTATGCATGATCCAGGACATACTCATTCACCCTCCCCATAGACAGCTTCAAACCACTACCAAGTCACCTAACTATGAATGGTTGCAGGACATAAATCTC<br>ACTCTCATGCTCTCCCCCCAACAAGTCACCTAACTATGAATGGTTACAGGACATACATTTAACTACC––<br>ATGTTCTAACCCATTTGGTTATGCTCGCCGTATCAGATGGATTTATTGATCGTCCACCTCACGAGAGATCAGCAACCCCTGCCTGTAATGTACTTCATGACCAGTCTCAGGCCCATTCTTTCCCCCTACACCCCTCGCCCTACTTG<br>CCTTCCACCGTACCTCTGGTTCCTCGGTCAGGCACATCCCATGCATAACTCCTGAACTTTCTCACTTTTCACGAAGTCATCTGTGGATTATCTTCCCCTCTTTAGTCCGTGATCGCGGCATCTTCTCTCTTCTATTGCTGTTGGTTC<br>CTTCTCTTTTTGGGGCTTCTTCACAGGTTGCCCTTCACAGTGCGGGTGCGGAGTGCTATTCAAGTGAAGCCTGGACTACACCTGCGTTGCGTCCTATCCTAGTCCTCTCGTGTCCCTCGATGAGACGGTTTGCGTGTATGGGGAAT<br>CATCTTGACACTGATGCACTTTGGATCGCATTTGGTTATGGTTCTTCCACCCCCCC––<br>GGTAAATGGTGCTATTTAGTGAATGCTTGTCGGACATATTTTTATCAATTTTCACTTCCTCTATTTTCTTCACAAAACTAGGAAATTCACCACAATTTTTTCTTTGTTATTTTTTAATTTTTTTTTTATTTTTTAAAAACATTTTTTAAA<br>AAACTAAATTACATACAAACTACCGCATAAAATCCCTCAAACTATACAAACGTTTATCGTATAATATATATACATTATTGTTTATTCTATCATTATTAGAGAAACTCCACTACCAAAACCATCATTAAAACAAAAATTTACATGCCAC<br>TTAACTCCCCTCACAAACAATCGTTATTTATATTGTTAATTAGCAAACACAAAACCCGC

CamHap_5	ATTTTATTTTTTAACCTAACTCCCCTACTAAGTGTACCCCCCCTTTCCCCCCCAGGGGGGGTATACTATGCATAATCGTGCATACATTTATATACCACATATATTATGGTACCGGTAATATATACTATATATGTACTAAACCCATTAT<br>ATGTATACGGGCATTAACCTATATTCCACATTTCTCCCAATGTCCATTCTATGCATGATCCAGGACATACTCATTTACCCTCCCCATAGACAGTTCCAAACCACTATCAAGCCACCTAACTATGAATGGTTACAGGACATAAATCTC<br>ACTCTCATGTTCTCCCCCCAACAAGTCACCTAACTATGAATGGTTACAGGACATACATTTAACTACC---<br>ATGTTCTAACCCATTTGGTTATGCTCGCCGTATCAGATGGATTTATTGATCGTCCACCTCACGAGAGATCAGCAACCCCTGCCTGTAATGTACTTCATGACCAGTCTCAGGCCCATTCTTTCCCCCTACACCCCTCGCCCTACTTG<br>CCTTCCACCGTACCTCTGGTTCCTCGGTCAGGCACATCCCATGCATAACTCCTGAACTTTCTCACTTTTCACGAAGTCATCTGTGGATTATCTTCCCCTCTTTAGTCCGTGATCGCGGCATCTTCTCTCTTCTATTGCTGTTGGTTC<br>CTTCTCTTTTTGGGGCTTCTTCACAGGTTACCCTTCACAGTGCGGGTGCGGAGTGCTATTCAAGTGAAGCCTGGACTACACCTGCGTTGCGTCCTATCCTAGTCCTCTCGTGTCCCTCGATGAGACGGTTTGCGTGTATGGGGAAT<br>CATCTTGACACTGATGCACTTTGGATCGCATTTGGTTATGGTTCTTCCACCCCCCC--<br>GGTAAATGGTGCTATTTAGTGAATGCTTGTCGGACATATTTTTATCAATTTTCACTTCCTCTATTTTCTTCACAAAACTAGGAAATTCACCACAATTTTTTCTTTGTTATTTTTTAATTTTTTTTTTATTTTTTAAAAACATTTTTTAAA<br>AAACTAAATTACATACAAACTACCGCATAAAATCCCTCAAACTATACAAACGTTTATCGTATAATATATATACATTATTGTTTATTCTATCATTATTAGAGAAACTCCACTACCAAAACCATCATTAAAACAAAAATTTACATGCCAC<br>TTAACTCCCCTCACAAACAATCGTTATTTATATTGTTAATTAGCAAACACAAAACCCGC
CamHap_6	ATTTTATTTTTTAACCTAACTCCCCTACTAAGTGTACCCCCCCTTTCCCCCCCAGGGGGGGTATACTATGCATAATCGTGCATACATTTATATACCACATATATTATGGTACCGGTAATATATACTATATATGTACTAAACCCATTAT<br>ATGTATACGGGCATTAATCTATATTCCACATTTCTCCCAATGTCCATTCTATGCATGATCCAAGACATACTCATTCACTCTCCCCATGGACAGCTCTAAACCACTATCAAGCCACCTAACTATGAATGGTTGCAGGACATAAATCTC<br>ACTCTCATGTTCTTCCCCCAACAAGTCACCTAACTATGAATGGTTACAGGACATACATCTAACTACT---<br>ATGTTCTAACCCATTTGGTTATGCTCGTCGTATCAGATGGATTTATTGATCGTCCACCTCACGAGAGATCAGCAACCCCTGCCTGTAATGTACTTCATGACCAGTCTCAGGCCCATTCTTTCCCCCTACACCCCTCGCCCTACTTG<br>CCTTCCACCGTACCTCTGGTTCCTCGGTCAGGCACATCCCATGCATAACTCCTGAACTTTCTCACTTTTCACGAAGTCATCTGTGGATTATCTTCCCCTCTTTAGTCCGTGATCGCGGCATCTTCTCTCTTCTATTGCTGTTGGTTC<br>CTTCTCTTTTTGGGGCTTCTTCACAGGTTGCCCTTCACAGTGCGGGTGCGGAGTGCTATTCAAGTGAAGCCTGGACTACACCTGCGTTGCGTCCTATCCTAGTCCTCTCGTGTCCCTCGATGAGACGGTTTGCGTGTATGGGGAAT<br>CATCTTGACACTGATGCACTTTGGATCGCATTTGGTTATGGTTCTTCCACCCCCCC--<br>GGTAAATGGTGCTATTTAGTGAATGCTTGTCGGACATATTTTTATCAATTTTCACTTCCTCTATTTTCTTCACAAAACTAGGAAATTCACCACAATTTTTTCTTTGTTATTTTTTAATTTTTTTTTTATTTTTTAAAAACATTTTTTAAA<br>AAACTAAATTACATACAAACTACCGCATAAAATCCCTCAAACTATACAAACGTTTATCGTATAATATATATACATTATTGTTTATTCTATCATTATTAGAGAAACTCCACTACCAAAACCATCATTAAAACAAAAATTTACATGCCAC<br>TTAACTCCCCTCACAAACAATCGTTATTTATATTGTTAATTAGCAAACACAAAACCCGC
CamHap_7	ATTTTATTTTTTAACCTAACTCCCCTACTAAGTGTACCCCCCCTTTCCCCCCCAGGGGGGGTATACTATGCATAATCGTGCATACATTTATATACCACATATATTATGGTACCGGTAATATATACTATATATGTACTAAACCCATTAT<br>ATGTATACGGGCATTAATCTATATTCCACATTTCTCCCAATGTCCATTCTATGCATGATCCAGGACATACTCATTCACCCTCCCCATAGACAGCTCCAAACCACTACCAAGTCACCTAACTATGAATGGTTGCAGGACATAAATCTC<br>ACTCTCATGCTCCTCCCCCAACAAGTCACCTAACTATGAATGGTTACAGGACATACATTTAACTACC---<br>ATGTTCTAACCCATTTGGTTATGCTCGCCGTATCAGATGGATTTATTGATCGTCCACCTCACGAGAGATCAGCAACCCCTGCCTGTAATGTACTTCATGACCAGTCTCAGGCCCATTCTTTCCCCCTACACCCCTCGCCCTACTTG<br>CCTTCCACCGTACCTCTGGTTCCTCGGTCAGGCACATCCCATGCATAACTCCTGAACTTTCTCACTTTTCACGAAGTCATCTGTGGATTATCTTCCCCTCTTTAGTCCGTGATCGCGGCATCTTCTCTCTTCTATTGCTGTTGGTTC<br>CTTCTCTTTTTGGGGCTTCTTCACAGGTTGCCCTTCACAGTGCGGGTGCGGAGTGCTATTCAAGTGAAGCCTGGACTACACCTGCGTTGCGTCCTATCCTAGTCCTCTCGTGTCCCTCGATGAGACGGTTTGCGTGTATGGGGAAT<br>CATCTTGACACTGATGCACTTTGGATCGCATTTGGTTATGGTTCTTCCACCCCCCCC-<br>GGTAAATGGTGCTATTTAGTGAATGCTTGTCGGACATATTTTTATCAATTTTCACTTCCTCTATTTTCTTCACAAAACTAGGAAATTCACCACAATTTTTTCTTTGTTATTTTTTAATTTTTTTTTTATTTTTTAAAAACATTTTTTAAA<br>AAACTAAATTACATACAAACTACCGCATAAAATCCCTCAAACTATACAAACGTTTATCGTATAATATATATACATTATTGTTTATTCTATCATTATTAGAGAAACTCCACTACCAAAACCATCATTAAAACAAAAATTTACATGCCAC<br>TTAACTCCCCTCACAAACAATCGTTATTTATATTGTTAATTAGCAAACACAAAACCCGC
CamHap_8	ATTTTATTTTTTAACCTAACTCCCCTACTAAGTGTACCCCCCCTTTCCCCCCCAGGGGGGGTATACTATGCATAATCGTGCATACATTTATATACCACATATATTATGGTACCGGTAATATATACTATATATGTACTAAACCCATTAT<br>ATGTATACGGGCATTAATCTATATTCCACATTTCTCCCAATGTCCATTCTATGCATGATCCAAGACATACTCATTCACCCTCCCCATAGACAGTTCTAAACCACTATCAAGCCACCTAACTATGAATGGTTACAGGACATAAATCTT<br>ACTCTCATGTTCTCCCCCTAACAAGTCACCTAACTATGAATGGTTACAGGACATACATTTAACTACC---<br>ATGTTCTAACCCATTTGGTTATGCTCGCCGTATCAGATGGATTTATTGATCGTCCACCTCACGAGAGATCAGCAACCCCTGCCTGTAATGTACTTCATGACCAGTCTCAGGCCCATTCTTTCCCCCTACACCCCTCGCCCTACTTG<br>CCTTCCACCGTACCTCTGGTTCCTCGGTCAGGCACATCCCATGCATAACTCCTGAACTTTCTCACTTTTCACGAAGTCATCTGTGGATTATCTTCCCCTCTTTAGTCCGTGATCGCGGCATCTTCTCTCTTCTATTGCTGTTGGTTC<br>CTTCTCTTTTTGGGGCTTCTTCACAGGTTGCCCTTCACAGTGCGGGTGCGGAGTGCTATTCAAGTGAAGCCTGGACTACACCTGCGTTGCGTCCTATCCTAGTCCTCTCGTGTCCCTCGATGAGACGGTTTGCGTATATGGGGAAT<br>CATCTTGACACTGATGCACTTTGGATCGCATTTGGTTATGGTTCTTCCACCCCCCCC-<br>GGTAAATGGTGCTATTTAGTGAATGCTTGTCGGACATATTTTTATCAATTTTCACTTCCTCTATTTTCTTCACAAAACTAGGAAATTCACCACAATTTTTTCTTTGTTATTTTTTAATTTTTTTTTTATTTTTTAAAAACATTTTTTAAA<br>AAACTAAATTACATACAAACTACCGCATAAAATCCCTCAAACTATACAAACGTTTATCGTATAATATATATACATTATTGTTTATTCTATCATTATTAGAGAAACTCCACTACCAAAACCATCATTAAAACAAAAATTTACATGCCAC<br>TTAACTCCCCTCACAAACAATCGTTATTTATATTGTTAATTAGCAAACACAAAACCCAC

CamHap_9	ATTTTATTTTTTAACCTAACTCCCCTACTAAGTGTACCCCCCCTTTCCCCCCCAGGGGGGGTATACTATGCATAATCGTGCATACATTTATATACCACATATATTATGGTACCGGTAATATATACTATATATGTACTAAACCCATTAT<br>ATGTATACGGGCATTAATCTATATTCCACATTTCTCCCAATGTCCATTCTATGCATGATCCAAGACATACTCATTCACCCTCCCCATAGACAGTTCTAAACCACTATCAAGCCACCTAACTATGAATGGTTACAGGACATAAATCTC<br>ACTCTCATGTTCTCCCCCTAACAAGTCACCTAACTATGAATGGTTACAGGACATACATTTAACTACC--<br>ATGTTCTAACCCATTTGGTTATGCTCGCCGTATCAGATGGATTTATTGATCGTCCACCTCACGAGAGATCAGCAACCCCTGCCTGTAATGTACTTCATGACCAGTCTCAGGCCCATTCTTTCCCCCTACACCCCTCGCCCTACTTG<br>CCTTCCACCGTACCTCTGGTTCCTCGGTCAGGCACATCCCATGCATAACTCCTGAACTTTCTCACTTTTCACGAAGTCATCTGTGGATTATCTTCCCCTCTTTAGTCCGTGATCGCGGCATCTTCTCTCTTCTATTGCTGTTGGTTC<br>CTTCTCTTTTTGGGGCTTCTTCACAGGTTGCCCTTCACAGTGCGGGTGCGGAGTGCTATTCAAGTGAAGCCTGGACTACACCTGCGTTGCGTCCTATCCTAGTCCTCTCGTGTCCCTCGATGAGACGGTTTGCGTATATGGGGAAT<br>CATCTTGACACTGATGCACTTTGGATCGCATTTGGTTATGGTTCTTCCACCCCCCC--<br>GGTAAATGGTGCTATTTAGTGAATGCTTGTCGGACATATTTTTATCAATTTTCACTTCCTCTATTTTCTTCACAAAACTAGGAAATTCACCACAATTTTTTCTTTGTTATTTTTTAATTTTTTTTTTATTTTTTAAAAACATTTTTTAAA<br>AAACTAAATTACATACAAACTACCGCATAAAATCCCTCAAACTATACAAACGTTTATCGTATAATATATATACATTATTGTTTATTCTATCATTATTAGAGAAACTCCACTACCAAAACCATCATTAAAACAAAAATTTACATGCCAC<br>TTAACTCCCCTCACAAACAATCGTTATTTATATTGTTAATTAGCAAACACAAAACCCAC
CamHap_10	ATTTTATTTTTTAACCTAACTCCCCTACTAAGTGTACCCCCCCTTTCCCCCCCAGGGGGGGTATACTATGCATAATCGTGCATACATTTATATACCACATATATTATGGTACCGGTAATATATACTATATATGTACTAAACCCATTAT<br>ATGTATACGGGCATTAACCTATATTCCACATTTCTCCCAATGTCCATTCTATGCATGATCCAGGACATACTCATTTACCCTCCCCATAGACAGTTCCAAACCACTATCAAGCCACCTAACTATGAATGGTTACAGGACATAAATCTC<br>ACTCTCATGTTCTCCCCCCAACAAGTCACCTAACTATGAATGGTTACAGGACATACATTTAACTACC--<br>ATGTTCTAACCCATTTGGTTATGCTCGTCGTATCAGATGGATTTATTGATCGTCCACCTCACGAGAGATCAGCAACCCCTGCCTGTAATGTACTTCATGACCAGTCTCAGGCCCATTCTTTCCCCCTACACCCCTCGCCCTACTTG<br>CCTTCCACCGTACCTCTGGTTCCTCGGTCAGGCACATCCCATGCATAACTCCTGAACTTTCTCACTTTTCACGAAGTCATCTGTGGATTATCTTCCCCTCTTTAGTCCGTGATCGCGGCATCTTCTCTCTTCTATTGCTGTTGGTTC<br>CTTCTCTTTTTGGGGCTTCTTCACAGGTTGCCCTTCACAGTGCGGGTGCGGAGTGCTATTCAAGTGAAGCCTGGACTACACCTGCGTTGCGTCCTATCCTAGTCCTCTCGTGTCCCTCGATGAGACGGTTTGCGTGTATGGGGAAT<br>CATCTTGACACTGATGCACTTTGGATCGCATTTGGTTATGGTTCTTCCACCCCCCC--<br>GGTAAATGGTGCTATTTAGTGAATGCTTGTCGGACATATTTTTATCAATTTTCACTTCCTCTATTTTCTTCACAAAACTAGGAAATTCACCACAATTTTTTCTTTGTTATTTTTTAATTTTTTTTTTATTTTTTAAAAACATTTTTTAAA<br>AAACTAAATTACATACAAACTACCGCATAAAATCCCTCAAACTATACAAACGTTTATCGTATAATATATATACATTATTGTTTATTCTATCATTATTAGAGAAACTCCACTACCAAAACCATCATTAAAACAAAAATTTACATGCCAC<br>TTAACTCCCCTCACAAACAATCGTTATTTATATTGTTAATTAGCAAACACAAAACCCGC
CamHap_11	ATTTTATTTTTTAACCTAACTCCCCTACTAAGTGTACCCCCCCTTTCCCCCCCAGGGGGGGTATACTATGCATAATCGTGCATACATTTATATACCACATATATTATGGTACCGGTAATATATACTATATATGTACTAAACCCATTAT<br>ATGTATACGGGCATTAACCTATATTCCACATTTCTCCCAATGTCCATTCTATGCATGATCCAGGACATACTCATTTACCCTCCCCATAGACAGTTCCAAACCACTATCAAGCCACCTAACTATGAATGGTTACAGGACATAAATCTC<br>ACTCTCATGTTCTCCCCCCAACAAGTCACCTAACTATGAATGGTTACAGGACATACATTTAACTACC--<br>ATGTTCTAACCCATTTGGTTATGCTCGCCGTATCAGATGGATTTATTGATCGTCCACCTCACGAGAGATCAGCAACCCCTGCCTGTAATGTACTTCATGACCAGTCTCAGGCCCATTCTTTCCCCCTACACCCCTCGCCCTACTTG<br>CCTTCCACCGTACCTCTGGTTCCTCGGTCAGGCACATCCCATGCATAACTCCTGAACTTTCTCACTTTTCACGAAGTCATCTGTGGATTATCTTCCCCTCTTTAGTCCGTGATCGCGGCATCTTCTCTCTTCTATTGCTGTTGGTTC<br>CTTCTCTTTTTGGGGCTTCTTCACAGGTTGCCCTTCACAGTGCGGGTGCGGAGTGCTATTCAAGTGAAGCCTGGACTACACCTGCGTTGCGTCCTATCCTAGTCCTCTCGTGTCCCTCGATGAGACGGTTTGCGTGTATGGGGAAT<br>CATCTTGACACTGATGCACTTTGGATCGCATTTGGTTATGGTTCTTCCACCCCCCC--<br>GGTAAATGGTGCTATTTAGTGAATGCTTGTCGGACATATTTTTATCAATTTTCACTTCCTCTATTTTCTTCACAAAACTAGGAAATTCACCACAATTTTTTCTTTGTTATTTTTTAATTTTTTTTTTATTTTTTAAAAACATTTTTTAAA<br>AAACTAAATTACATACAAACTACCGCATAAAATCCCTCAAACTATACAAACGTTTATCGTATAATATATATACATTATTGTTTATTCTATCATTATTAGAGAAACTCCACTACCAAAACCATCATTAAAACAAAAATTTACATGCCAC<br>TTAACTCCCCTCACAAACAATCGTTATTTATATTGTTAATTAGCAAACACAAAACCCGC
CamHap_12	ATTTTATTTTTTAACCTAACTCCCCTACTAAGTGTACCCCCCCTTTCCCCCCCAGGGGGGGTATACTATGCATAATCGTGCATACATTTATATACCACATATATTATGGTACCGGTAATATATACTATATATGTACTAAACCCATTAT<br>ATGTATACGGGCATTAATCTATATTCCACATTTCTCCCAATGTCCATTCTATGCATGATCCAAGACATACTCATTCACCCTCCCCATAGACAGTTCTAAACCACTATCAAGCCACCTAACTATGAATGGTTACAGGACATAAATCTT<br>ACTCTCATGTTCTCCCCCTAACAAGTCACCTAACTATGAATGGTTACAGGACATACATCTAACTACC--<br>ATGTTCTAACCCATTTGGTTATGCTCGCCGTATCAGATGGATTTATTGATCGTCCACCTCACGAGAGATCAGCAACCCCTGCCTGTAATGTACTTCATGACCAGTCTCAGGCCCATTCTTTCCCCCTACACCCCTCGCCCTACTTG<br>CCTTCCACCGTACCTCTGGTTCCTCGGTCAGGCACATCCCATGCATAACTCCTGAACTTTCTCACTTTTCACGAAGTCATCTGTGGATTATCTTCCCCTCTTTAGTCCGTGATCGCGGCATCTTCTCTCTTCTATTGCTGTTGGTTC<br>CTTCTCTTTTTGGGGCTTCTTCACAGGTTGCCCTTCACAGTGCGGGTGCGGAGTGCTATTCAAGTGAAGCCTGGACTACACCTGCGTTGCGTCCTATCCTAGTCCTCTCGTGTCCCTCGATGAGACGGTTTGCGTATATGGGGAAT<br>CATCTTGACACTGATGCACTTTGGATCGCATTTGGTTATGGTTCTTCCACCCCCCC--<br>GGTAAATGGTGCTATTTAGTGAATGCTTGTCGGACATATTTTTATCAATTTTCACTTCCTCTATTTTCTTCACAAAACTAGGAAATTCACCACAATTTTTTCTTTGTTATTTTTTAATTTTTTTTTTATTTTTTAAAAACATTTTTTAAA<br>AAACTAAATTACATACAAACTACCGCATAAAATCCCTCAAACTATACAAACGTTTATCGTATAATATATATACATTATTGTTTATTCTATCATTATTAGAGAAACTCCACTACCAAAACCATCATTAAAACAAAAATTTACATGCCAC<br>TTAACTCCCCTCACAAACAATCGTTATTTATATTGTTAATTAGCAAACACAAAACCCAC

CamHap_13	ATTTTATTTTTTAACCTAACTCCCCTACTAAGTGTACCCCCCCTTTCCCCCCCAGGGGGGGTATACTATGCATAATCGTGCATACATTTATATACCACATATATTATGGTACCGGTAATATATACTATATATGTACTAAACCCATTAT<br>ATGTATACGGGCATTAATCTATATTCCACATTTCTCCCAATGTCCATTCTATGCATGATCCAGGACATACTCATTCACCCTCCCCATAGACAGCTCCAAACCACTACCAAGTCACCTAACTATGAATGGTTGCAGGACATAAATCTC<br>ACTCTCATGCTCTTCCCCCAACAAGTCACCTAACTATGAATGGTTGCAGGACATACATTTAACTACC---<br>ATGTTCTAACCCATTTGGTTATGCTCGCCGTATCAGATGGATTTATTGATCGTCCACCTCACGAGAGATCAGCAACCCCTGCCTGTAATGTACTTCATGACCAGTCTCAGGCCCATTCTTTCCCCCTACACCCCTCGCCCTACTTG<br>CCTTCCACCGTACCTCTGGTTCCTCGGTCAGGCACATCCCATGCATAACTCCTGAACTTTCTCACTTTTCACGAAGTCATCTGTGGATTATCTTCCCCTCTTTAGTCCGTGATCGCGGCATCTTCTCTCTTCTATTGCTGTTGGTTC<br>CTTCTCTTTTTGGGGCTTCTTCACAGGTTGCCCTTCACAGTGCGGGTGCGGAGTGCTATTCAAGTGAAGCCTGGACTACACCTGCGTTGCGTCCTATCCTAGTCCTCTCGTGTCCCTCGATGAGACGGTTTGCGTGTATGGGGAAT<br>CATCTTGACACTGATGCACTTTGGATCGCATTTGGTTATGGCTCTTCCACCCCCCCC-<br>GGTAAATGGTGCTATTTAGTGAATGCTTGTCGGACATATTTTTATCAATTTTCACTTCCTCTATTTTCTTCACAAAACTAGGAAATTCACCACAATTTTTTCTTTGTTATTTTTTAATTTTTTTTTTATTTTTTAAAAACATTTTTTAAA<br>AAACTAAATTACATACAAACTACCGCATAAAATCCCTCAAACTATACAAACGTTTATCGTATAATATATATACATTATTGTTTATTCTATCATTATTAGAGAAACTCCACTACCAAAACCATCATTAAAACAAAAATTTACATGCCAC<br>TTAACTCCCCTCACAAACAATCGTTATTTATATTGTTAATTAGCAAACACAAAACCCGC
CamHap_14	ATTTTATTTTTTAACCTAACTCCCCTACTAAGTGTACCCCCCCTTTCCCCCCCAGGGGGGGTATACTATGCATAATCGTGCATACATTTATATACCACATATATTATGGTACCGGTAATATATACTATATATGTACTAAACCCATTAT<br>ATGTATACGGGCATTAATCTATATTCCACATTTCTCCCAATGTCCATTCTATGCATGATCCAAGACATACTCATTCACCCTCCCCATAGACAGTTCTAAACCACTATCAAGCCACCTAACTATGAATGGTTACAGGACATAAATCTT<br>ACTCTCATGTTCTCCCCCTAACAAGTCACCTAACTATGAATGGTTACAGGACATACATCTAACTACC---<br>ATGTTCTAACCCATTTGGTTATGCTCGCCGTATCAGATGGATTTATTGATCGTCCACCTCACGAGAGATCAGCAACCCCTGCCTGTAATGTACTTCATGACCAGTCTCAGGCCCATTCTTTCCCCCTACACCCCTCGCCCTACTTG<br>CCTTCCACCGTACCTCTGGTTCCTCGGTCAGGCACATCCCATGCATAACTCCTGAACTTTCTCACTTTTCACGAAGTCATCTGTGGATTATCTTCCCCTCTTTAGTCCGTGATCGCGGCATCTTCTCTCTTCTATTGCTGTTGGTTC<br>CTTCTCTTTTTGGGGCTTCTTCACAGGTTGCCCTTCACAGTGCGGGTGCGGAGTGCTATTCAAGTGAAGCCTGGACTACACCTGCGTTGCGTCCTATCCTAGTCCTCTCGTGTCCCTCGATGAGACGGTTTGCGTATATGGGGAAT<br>CATCTTGACACTGATGCACTTTGGATCGCATTTGGTTATGGTTCTTCCACCCCCCCC-<br>GGTAAATGGTGCTATTTAGTGAATGCTTGTCGGACATATTTTTATCAATTTTCACTTCCTCTATTTTCTTCACAAAACTAGGAAATTCACCACAATTTTTTCTTTGTTATTTTTTAATTTTTTTTTTATTTTTTAAAAACATTTTTTAAA<br>AAACTAAATTACATACAAACTACCGCATAAAATCCCTCAAACTATACAAACGTTTATCGTATAATATATATACATTATTGTTTATTCTATCATTATTAGAGAAACTCCACTACCAAAACCATCATTAAAACAAAAATTTACATGCCAC<br>TTAACTCCCCTCACAAACAATCGTTATTTATATTGTTAATTAGCAAACACAAAACCCAC
CamHap_15	ATTTTATTTTTTAACCTAACTCCCCTACTAAGTGTACCCCCCCTTTCCCCCCCAGGGGGGGTATACTATGCATAATCGTGCATACATTTATATACCACATATATTATGGTACCGGTAATATATACTATATATGTACTAAACCCATTAT<br>ATGTATACGGGCATTAATCTATATTCCACATTTCTCCCAATGTCCATTCTATGCATGATCCAAGACATACTCATTCACCCTCCCCATAGACAGTTCTAAACCACTATCAAGCCACCTAACTATGAATGGTTACAGGACATAAATCTT<br>ACTCTCATGTTCTCCCCCTAACAAGTCACCTAACTATGAATGGTTACAGGACATACATTTAACTACC---<br>ATGTTCTAACCCATTTGGTTATGCTCGCCGTATCAGATGGATTTATTGATCGTCCACCTCACGAGAGATCAGCAACCCCTGCCTGTAATGTACTTCATGACCAGTCTCAGGCCCATTCTTTCCCCCTACACCCCTCGCCCTACTTG<br>CCTTCCACCGTACCTCTGGTTCCTCGGTCAGGCACATCCCATGCATAACTCCTGAACTTTCTCACTTTTCACGAAGTCATCTGTGGATTATCTTCCCCTCTTTAGTCCGTGATCGCGGCATCTTCTCTCTTCTATTGCTGTTGGTTC<br>CTTCTCTTTTTGGGGCTTCTTCACAGGTTGCCCTTCACAGTGCGGGTGCGGAGTGCTATTCAAGTGAAGCCTGGACTACACCTGCGTTGCGTCCTATCCTAGTCCTCTCGTGTCCCTCGATGAGACGGTTTGCGTGTATGGGGAAT<br>CATCTTGACACTGATGCACTTTGGATCGCATTTGGTTATGGTTCTTCCACCCCCCCC-<br>GGTAAATGGTGCTATTTAGTGAATGCTTGTCGGACATATTTTTATCAATTTTCACTTCCTCTATTTTCTTCACAAAACTAGGAAATTCACCACAATTTTTTCTTTGTTATTTTTTAATTTTTTTTTTATTTTTTAAAAACATTTTTTAAA<br>AAACTAAATTACATACAAACTACCGCATAAAATCCCTCAAACTATACAAACGTTTATCGTATAATATATATACATTATTGTTTATTCTATCATTATTAGAGAAACTCCACTACCAAAACCATCATTAAAACAAAAATTTACATGCCAC<br>TTAACTCCCCTCACAAACAATCGTTATTTATATTGTTAATTAGCAAACACAAAACCCGC
CamHap_16	ATTTTATTTTTTAACCTAACTCCCCTACTAAGTGTACCCCCCCTTTCCCCCCCAGGGGGGGTATACTATGCATAATCGTGCATACATTTATATACCACATATATTATGGTACCGGTAATATATACTATATATGTACTAAACCCATTAT<br>ATGTATACGGGCATTAACCTATATTCCACATTTCTCCCAATGTCCATTCTATGCATGATCCAGGACATACTCATTTACCCTCCCCATAGACAGTTCCAAACCACTATCAAGCCACCTAACTATGAATGGTTACAGGACATAAATCTC<br>ACTCTCATGTTCTCCCCCCAACAAGTCACCTAACTATGAATGGTTACAGGACATACATTTAACTACC---<br>ATGTTCTAACCCATTTGGTTATGCTCGCCGTATCAGATGGATTTATTGATCGTCCACCTCACGAGAGATCAGCAACCCCTGCCTGTAATGTACTTCATGACCAGTCTCAGGCCCATTCTTTCCCCCTACACCCCTCGCCCTACTTG<br>CCTTCCACCGTACCTCTGGTTCCTCGGTCAGGCACATCCCATGCATAACTCCTGAACTTTCTCACTTTTCACGAAGTCATCTGTGGATTATCTTCCCCTCTTTAGTCCGTGATCGCGGCATCTTCTCTCTTCTATTGCTGTTGGTTC<br>CTTCTCTTTTTGGGGCTTCTTCACAGGTTACCCTTCACAGTGCGGGTGCGGAGTGCTATTCAAGTGAAGCCTGGACTACACCTGCGTTGCGTCCTATCCTAGTCCTCTCGTGTCCCTCGATGAGACGGTTTGCGTGTATGGGGAAT<br>CATCTTGACACTGATGCACTTTGGATCGCATTTGGTTATGGTTCTTCCACCCCCCCC-<br>GGTAAATGGTGCTATTTAGTGAATGCTTGTCGGACATATTTTTATCAATTTTCACTTCCTCTATTTTCTTCACAAAACTAGGAAATTCACCACAATTTTTTCTTTGTTATTTTTTAATTTTTTTTTTATTTTTTAAAAACATTTTTTAAA<br>AAACTAAATTACATACAAACTACCGCATAAAATCCCTCAAACTATACAAACGTTTATCGTATAATATATATACATTATTGTTTATTCTATCATTATTAGAGAAACTCCACTACCAAAACCATCATTAAAACAAAAATTTACATGCCAC<br>TTAACTCCCCTCACAAACAATCGTTATTTATATTGTTAATTAGCAAACACAAAACCCGC

CamHap_17	ATTTTATTTTTTAACCTAACTCCCCTACTAAGTGTACCCCCCCCTTTCCCCCCCAGGGGGGGTATACTATGCATAATCGTGCATACATTTATATACCACATATATTATGGTACCGGTAATATATACTATATATGTACTAAACCCATTAT<br>ATGTATACGGGCATTAATCTATATTCCACATTTCTCCCAATGTCCATTCTATGCATGATCCAAGACATACTCATTCACTCTCCCCATGGACAGCTCTAAACCACTACCAAGCCACCTAACTATGAATGGTTGCAGGACATAAATCTC<br>ACTCTCATGTTCTTCCCCCAACAAGTCACCTAACTATGAATGGTTACAGGACATACATCTAACTACT--<br>ATGTTCTAACCCATTTGGTTATGCTCGTCGTATCAGATGGATTTATTGATCGTCCACCTCACGAGAGATCAGCAACCCCTGCCTGTAATGTACTTCATGACCAGTCTCAGGCCCATTCTTTCCCCCTACACCCCTCGCCCTACTTG<br>CCTTCCACCGTACCTCTGGTTCCTCGGTCAGGCACATCCCATGCATAACTCCTGAACTTTCTCACTTTTCACGAAGTCATCTGTGGATTATCTTCCCCTCTTTAGTCCGTGATCGCGGCATCTTCTCTCTTCTATTGCTGTTGGTTC<br>CTTCTCTTTTTGGGGCTTCTTCACAGGTTGCCCTTCACAGTGCGGGTGCGGAGTGCTATTCAAGTGAAGCCTGGACTACACCTGCGTTGCGTCCTATCCTAGTCCTCTCGTGTCCCTCGATGAGACGGTTTGCGTGTATGGGGAAT<br>CATCTTGACACTGATGCACTTTGGATCGCATTTGGTTATGGTTCTTCCACCCCCCCC-<br>GGTAAATGGTGCTATTTAGTGAATGCTTGTCGGACATATTTTTATCAATTTTCACTTCCTCTATTTTCTTCACAAAACTAGGAAATTCACCACAATTTTTTCTTTGTTATTTTTTAATTTTTTTTTTATTTTTTAAAAACATTTTTTAAA<br>AAACTAAATTACATACAAACTACCGCATAAAATCCCTCAAACTATACAAACGTTTATCGTATAATATATATACATTATTGTTTATTCTATCATTATTAGAGAAACTCCACTACCAAAACCATCATTAAAACAAAAATTTACATGCCAC<br>TTAACTCCCCTCACAAACAATCGTTATTTATATTGTTAATTAGCAAACACAAAACCCGC
CamHap_18	ATTTTATTTTTTAACCTAACTCCCCTACTAAGTGTACCCCCCCCTTTCCCCCCCAGGGGGGGTATACTATGCATAATCGTGCATACATTTATATACCACATATATTATGGTACCGGTAATATATACTATATATGTACTAAACCCATTAT<br>ATGTATACGGGCATTAATCTATATTCCACATTTCTCCCAATGTCCATTCTATGCATGATCCAGGACATACTCATTCACCCTCCCCATAGACAGCTCCAAACCACTACCAAGTCACCTAACTATGAATGGTTGCAGGACATAAATCTC<br>ACTCTCATGCTCTTCCCCCAACAAGTCACCTAACTATGAATGGTTACAGGACATACATTTAACTACC--<br>ATGTTCTAACCCATTTGGTTATGCTCGCCGTATCAGATGGATTTATTGATCGTCCACCTCACGAGAGATCAGCAACCCCTGCCTGTAATGTACTTCATGACCAGTCTCAGGCCCATTCTTTCCCCCTACACCCCTCGCCCTACTTG<br>CCTTCCACCGTACCTCTGGTTCCTCGGTCAGGCACATCCCATGCATAACTCCTGAACTTTCTCACTTTTCACGAAGTCATCTGTGGATTATCTTCCCCTCTTTAGTCCGTGATCGCGGCATCTTCTCTCTTCTATTGCTGTTGGTTC<br>CTTCTCTTTTTGGGGCTTCTTCACAGGTTGCCCTTCACAGTGCGGGTGCGGAGTGCTATTCAAGTGAAGCCTGGACTACACCTGCGTTGCGTCCTATCCTAGTCCTCTCGTGTCCCTCGATGAGACGGTTTGCGTGTATGGGGAAT<br>CATCTTGACACTGATGCACTTTGGATCGCATTTGGTTATGGTTCTTCCACCCCCCC--<br>GGTAAATGGTGCTATTTAGTGAATGCTTGTCGGACATATTTTTATCAATTTTCACTTCCTCTATTTTCTTCACAAAACTAGGAAATTCACCACAATTTTTTCTTTGTTATTTTTTAATTTTTTTTTTATTTTTTAAAAACATTTTTTAAA<br>AAACTAAATTACATACAAACTACCGCATAAAATCCCTCAAACTATACAAACGTTTATCGTATAATATATATACATTATTGTTTATTCTATCATTATTAGAGAAACTCCACTACCAAAACCATCATTAAAACAAAAATTTACATGCCAC<br>TTAACTCCCCTCACAAACAATCGTTATTTATATTGTTAATTAGCAAACACAAAACCCGC
CamHap_19	ATTTTATTTTTTAACCTAACTCCCCTACTAAGTGTACCCCCCCCTTTCCCCCCCAGGGGGGGTATACTATGCATAATCGTGCATACATTTATATACCACATATATTATGGTACCGGTAATATATACTATATATGTACTAAACCCATTAT<br>ATGTATACGGGCATTAATCTATATTCCACATTTCTCCCAATGTCCATTCTATGCATGATCCAAGACATACTCATTCACTCTCCCCATGGACAGCTCTAAACCACTACCAAGCCACCTAACTATGAATGGTTGCAGGACATAAATCTC<br>ACTCTCATGTTCTTCCCCCAACAAGTCACCTAACTATGAATGGTTACAGGACATACATCTAACTACT--<br>ATGTTCTAACCCATTTGGTTATGCTCGTCGTATCAGATGGATTTATTGATCGTCCACCTCACGAGAGATCAGCAACCCCTGCCTGTAATGTACTTCATGACCAGTCTCAGGCCCATTCTTTCCCCCTACACCCCTCGCCCTACTTG<br>CCTTCCACCGTACCTCTGGTTCCTCGGTCAGGCACATCCCATGCATAACTCCTGAACTTTCTCACTTTTCACGAAGTCATCTGTGGATTATCTTCCCCTCTTTAGTCCGTGATCGCGGCATCTTCTCTCTTCTATTGCTGTTGGTTC<br>CTTCTCTTTTTGGGGCTTCTTCACAGGTTGCCCTTCACAGTGCGGGTGCGGAGTGCTATTCAAGTGAAGCCTGGACTACACCTGCGTTGCGTCCTATCCTAGTCCTCTCGTGTCCCTCGATGAGACGGTTTGCGTGTATGGGGAAT<br>CATCTTGACACTGATGCACTTTGGATCGCATTTGGTTATGGTTCTTCCACCCCCCC--<br>GGTAAATGGTGCTATTTAGTGAATGCTTGTCGGACATATTTTTATCAATTTTCACTTCCTCTATTTTCTTCACAAAACTAGGAAATTCACCACAATTTTTTCTTTGTTATTTTTTAATTTTTTTTTTATTTTTTAAAAACATTTTTTAAA<br>AAACTAAATTACATACAAACTACCGCATAAAATCCCTCAAACTATACAAACGTTTATCGTATAATATATATACATTATTGTTTATTCTATCATTATTAGAGAAACTCCACTACCAAAACCATCATTAAAACAAAAATTTACATGCCAC<br>TTAACTCCCCTCACAAACAATCGTTATTTATATTGTTAATTAGCAAACACAAAACCCGC
CamHap_20	ATTTTATTTTTTAACCTAACTCCCCTACTAAGTGTACCCCCCCCTTTCCCCCCCAGGGGGGGTATACTATGCATAATCGTGCATACATTTATATACCACATATATTATGGTACCGGTAATATATACTATATATGTACTAAACCCATTAT<br>ATGTATACGGGCATTAATCTATATTCCACATTTCTCCCAATGTCCATTCTATGCATGATCCAAGACATACTCATTCACCCTCCCCATAGACAGTTCTAAACCACTATCAAGCCACCTAACTATGAATGGTTACAGGACATAAATCTT<br>ACTCTCATGTTCTCCCCCTAACAAGTCACCTAACTATGAATGGTTACAGGACATACATTCAACTACC--<br>ATGTTCTAACCCATTTGGTTATGCTCGCCGTATCAGATGGATTTATTGATCGTCCACCTCACGAGAGATCAGCAACCCCTGCCTGTAATGTACTTCATGACCAGTCTCAGGCCCATTCTTTCCCCCTACACCCCTCGCCCTACTTG<br>CCTTCCACCGTACCTCTGGTTCCTCGGTCAGGCACATCCCATGCATAACTCCTGAACTTTCTCACTTTTCACGAAGTCATCTGTGGATTATCTTCCCCTCTTTAGTCCGTGATCGCGGCATCTTCTCTCTTCTATTGCTGTTGGTTC<br>CTTCTCTTTTTGGGGCTTCTTCACAGGTTGCCCTTCACAGTGCGGGTGCGGAGTGCTATTCAAGTGAAGCCTGGACTACACCTGCGTTGCGTCCTATCCTAGTCCTCTCGTGTCCCTCGATGAGACGGTTTGCGTATATGGGGAAT<br>CATCTTGACACTGATGCACTTTGGATCGCATTTGGTTATGGTTCTTCCACCCCCCC--<br>GGTAAATGGTGCTATTTAGTGAATGCTTGTCGGACATATTTTTATCAATTTTCACTTCCTCTATTTTCTTCACAAAACTAGGAAATTCACCACAATTTTTTCTTTGTTATTTTTTAATTTTTTTTTTATTTTTTAAAAACATTTTTTAAA<br>AAACTAAATTACATACAAACTACCGCATAAAATCCCTCAAACTATACAAACGTTTATCGTATAATATATATACATTATTGTTTATTCTATCATTATTAGAGAAACTCCACTACCAAAACCATCATTAAAACAAAAATTTACATGCCAC<br>TTAACTCCCCTCACAAACAATCGTTATTTATATTGTTAATTAGCAAACACAAAACCCAC

CamHap_21	ATTTTATTTTTTAACCTAACTCCCCTACTAAGTGTACCCCCCCTTTCCCCCCCAGGGGGGGTATACTATGCATAATCGTGCATACATTTATATACCACATATATTATGGTACCGGTAATATATACTATATATGTACTAAACCCATTAT<br>ATGTATACGGGCATTAATCTATATTCCACATTTCTCCCAATGTCCATTCTATGCATGATCCAGGACATACTCATTCACCCTCCCCATAGACAGCTCCAAACCACTACCAAGTCACCTAACTATGAATGGTTGCGGACATAAATCT<br>CACTCTCATGCTCTTCCCCCAACAAGTCACCTAACTATGAATGGTTACAGGACATACATTTAACTACC---<br>ATGTTCTAACCCATTTGGTTATGCTCGCCGTATCAGATGGATTTATTGATCGTCCACCTCACGAGAGATCAGCAACCCCTGCCTGTAATGTACTTCATGACCAGTCTCAGGCCCATTCTTTCCCCCTACACCCCTCGCCCTACTTG<br>CCTTCCACCGTACCTCTGGTTCCTCGGTCAGGCACATCCCATGCATAACTCCTGAACTTTCTCACTTTTCACGAAGTCATCTGTGGATTATCTTCCCCTCTTTAGTCCGTGATCGCGGCATCTTCTCTCTTCTATTGCTGTTGGTTC<br>CTTCTCTTTTTGGGGCTTCTTCACAGGTTGCCCTTCACAGTGCGGGTGCGGAGTGCTATTCAAGTGAAGCCTGGACTACACCTGCGTTGCGTCCTATCCTAGTCCTCTCGTGTCCCTCGATGAGACGGTTTGCGTGTATGGGGAAT<br>CATCTTGACACTGATGCACTTTGGATCGCATTTGGTTATGGTTCTTCCACCCCCCC--<br>GGTAAATGGTGCTATTTAGTGAATGCTTGTCGGACATATTTTTATCAATTTTCACTTCCTCTATTTTCTTCACAAAACTAGGAAATTCACCACAATTTTTTCTTTGTTATTTTTTAATTTTTTTTTTATTTTTTAAAAACATTTTTTAAA<br>AAACTAAATTACATACAAACTACCGCATAAAATCCCTCAAACTATACAAACGTTTATCGTATAATATATATACATTATTGTTTATTCTATCATTATTAGAGAAACTCCACTACCAAAACCATCATTAAAACAAAAATTTACATGCCAC<br>TTAACTCCCCTCACAAACAATCGTTATTTATATTGTTAATTAGCAAACACAAAACCCGC
CamHap_22	ATTTTATTTTTTAACCTAACTCCCCTACTAAGTGTACCCCCCCTTTCCCCCCCAGGGGGGGTATACTATGCATAATCGTGCATACATTTATATACCACATATATTATGGTACCGGTAATATATACTATATATGTACTAAACCCATTAT<br>ATGTATACGGGCATTAATCTATATTCCACATTTCTCCCAATGTCCATTCTATGCATGATCCAGGACATACTCATTCACCCTCCCCATAGACAGCTCCAAACCACTACCAAGTCACCTAACTATGAATGGTTGCAGGACATAAATCTC<br>ACTCTCATGCTCTTCCCCCAACAAGTCACCTAACTATGAATGGTTGCAGGACATACATCTAACTACC---<br>ATGTTCTAACCCATTTGGTTATGCTCGCCGTATCAGATGGATTTATTGATCGTCCACCTCACGAGAGATCAGCAACCCCTGCCTGTAATGTACTTCATGACCAGTCTCAGGCCCATTCTTTCCCCCTACACCCCTCGCCCTACTTG<br>CCTTCCACCGTACCTCTGGTTCCTCGGTCAGGCACATCCCATGCATAACTCCTGAACTTTCTCACTTTTCACGAAGTCATCTGTGGATTATCTTCCCCTCTTTAGTCCGTGATCGCGGCATCTTCTCTCTTCTATTGCTGTTGGTTC<br>CTTCTCTTTTTGGGGCTTCTTCACAGGTTGCCCTTCACAGTGCGGGTGCGGAGTGCTATTCAAGTGAAGCCTGGACTACACCTGCGTTGCGTCCTATCCTAGTCCTCTCGTGTCCCTCGATGAGACGGTTTGCGTGTATGGGGAAT<br>CATCTTGACACTGATGCACTTTGGATCGCATTTGGTTATGGTTCTTCCACCCCCCCC-<br>GGTAAATGGTGCTATTTAGTGAATGCTTGTCGGACATATTTTTATCAATTTTCACTTCCTCTATTTTCTTCACAAAACTAGGAAATTCACCACAATTTTTTCTTTGTTATTTTTTAATTTTTTTTTTATTTTTTAAAAACATTTTTTAAA<br>AAACTAAATTACATACAAACTACCGCATAAAATCCCTCAAACTATACAAACGTTTATCGTATAATATATATACATTATTGTTTATTCTATCATTATTAGAGAAACTCCACTACCAAAACCATCATTAAAACAAAAATTTACATGCCAC<br>TTAACTCCCCTCACAAACAATCGTTATTTATATTGTTAATTAGCAAACACAAAACCCGC
CamHap_23	ATTTTATTTTTTAACCTAACTCCCCTACTAAGTGTACCCCCCCTTTCCCCCCCAGGGGGGGTATACTATGCATAATCGTGCATACATTTATATACCACATATATTATGGTACCGGTAATATATACTATATATGTACTAAACCCATTAT<br>ATGTATACGGGCATTAACCTATATTCCACATTTCTCCCAACGTCCATTCTATGCATGATCCAGGACATACTCATTTACCCTCCCCATAGACAGTTCCAAACCACTATCAAGCCACCTAACTATGAATGGTTACAGGACATAAATCTC<br>ACTCTCATGTTCTCCCCCCAACAAGTCACCTAACTATGAATGGTTACAGGACATACATTTAACTACC--<br>ATGTTCTAACCCATTTGGTTATGCTCGCCGTATCAGATGGATTTATTGATCGTCCACCTCACGAGAGATCAGCAACCCCTGCCTGTAATGTACTTCATGACCAGTCTCAGGCCCATTCTTTCCCCCTACACCCCTCGCCCTACTTG<br>CCTTCCACCGTACCTCTGGTTCCTCGGTCAGGCACATCCCATGCATAACTCCTGAACTTTCTCACTTTTCACGAAGTCATCTGTGGATTATCTTCCCCTCTTTAGTCCGTGATCGCGGCATCTTCTCTCTTCTATTGCTGTTGGTTC<br>CTTCTCTTTTTGGGGCTTCTTCACAGGTTGCCCTTCACAGTGCGGGTGCGGAGTGCTATTCAAGTGAAGCCTGGACTACACCTGCGTTGCGTCCTATCCTAGTCCTCTCGTGTCCCTCGATGAGACGGTTTGCGTGTATGGGGAAT<br>CATCTTGACACTGATGCACTTTGGATCGCATTTGGTTATGGTTCTTCCACCCCCCC--<br>GGTAAATGGTGCTATTTAGTGAATGCTTGTCGGACATATTTTTATCAATTTTCACTTCCTCTATTTTCTTCACAAAACTAGGAAATTCACCACAATTTTTTCTTTGTTATTTTTTAATTTTTTTTTTATTTTTTAAAAACATTTTTTAAA<br>AAACTAAATTACATACAAACTACCGCATAAAATCCCTCAAACTATACAAACGTTTATCGTATAATATATATACATTATTGTTTATTCTATCATTATTAGAGAAACTCCACTACCAAAACCATCATTAAAACAAAAATTTACATGCCAC<br>TTAACTCCCCTCACAAACAATCGTTATTTATATTGTTAATTAGCAAACACAAAACCCGC
CamHap_24	ATTTTATTTTTTAACCTAACTCCCCTACTAAGTGTACCCCCCCTTTCCCCCCCAGGGGGGGTATACTATGCATAATCGTGCATACATTTATATACCACATATATTATGGTACCGGTAATATATACTATATATGTACTAAACCCATTAT<br>ATGTATACGGGCATTAATCTATATTCCACATTTCTCCCAATGTCCATTCTATGCATGATCCAGGACATACTCATTCACCCTCCCCATAGACAGCCCCAAACCACTACCAAGTCACCTAACTATGAATGGTTGCAGGACATAAATCT<br>CACTCTCATGCTCTTCCCCCAACAAGTCACCTAACTATGAATGGTTACAGGACATACATTTAACTACC---<br>ATGTTCTAACCCATTTGGTTATGCTCGCCGTATCAGATGGATTTATTGATCGTCCACCTCACGAGAGATCAGCAACCCCTGCCTGTAATGTACTTCATGACCAGTCTCAGGCCCATTCTTTCCCCCTACACCCCTCGCCCTACTTG<br>CCTTCCACCGTACCTCTGGTTCCTCGGTCAGGCACATCCCATGCATAACTCCTGAACTTTCTCACTTTTCACGAAGTCATCTGTGGATTATCTTCCCCTCTTTAGTCCGTGATCGCGGCATCTTCTCTCTTCTATTGCTGTTGGTTC<br>CTTCTCTTTTTGGGGCTTCTTCACAGGTTGCCCTTCACAGTGCGGGTGCGGAGTGCTATTCAAGTGAAGCCTGGACTACACCTGCGTTGCGTCCTATCCTAGTCCTCTCGTGTCCCTCGATGAGACGGTTTGCGTGTATGGGGAAT<br>CATCTTGACACTGATGCACTTTGGATCGCATTTGGTTATGGTTCTTCCACCCCCCC--<br>GGTAAATGGTGCTATTTAGTGAATGCTTGTCGGACATATTTTTATCAATTTTCACTTCCTCTATTTTCTTCACAAAACTAGGAAATTCACCACAATTTTTTCTTTGTTATTTTTTAATTTTTTTTTTATTTTTTAAAAACATTTTTTAAA<br>AAACTAAATTACATACAAACTACCGCATAAAATCCCTCAAACTATACAAACGTTTATCGTATAATATATATACATTATTGTTTATTCTATCATTATTAGAGAAACTCCACTACCAAAACCATCATTAAAACAAAAATTTACATGCCAC<br>TTAACTCCCCTCACAAACAATCGTTATTTATATTGTTAATTAACAAACACAAAACCCGC

CamHap_25	ATTTTATTTTTTAACCTAACTCCCCTACTAAGTGTACCCCCCCTTTCCCCCCCAGGGGGGGTATACTATGCATAATCGTGCATACATTTATATACCACATATATTATGGTACCGGTAATATATACTATATATGTACTAAACCCATTAT<br>ATGTATACGGGCATTAATCTATATTCCACATTTCTCCCAATGTCCATTCTATGCATGATCCAGGACATACTCATTCACCCTCCCCATAGACAGCTCCAAACCACTACCAAGTCACCTAACTATGAATGGTTGCAGGACATAAATCTC<br>ACTCTCATGCTCTTCCCCCAACAAGTCACCTAACTATGAATGGTTGCAGGACATACATTTAACTACC--<br>ATGTTCTAACCCATTTGGTTATGCTCGCCGTATCAGATGGATTTATTGATCGTCCACCTCACGAGAGATCAGCAACCCCTGCCTGTAATGTACTTCATGACCAGTCTCAGGCCCATTCTTTCCCCCTACACCCCTCGCCCTACTTG<br>CCTTCCACCGTACCTCTGGTTCCTCGGTCAGGCACATCCCATGCATAACTCCTGAACTTTCTCACTTTTCACGAAGTCATCTGTGGATTATCTTCCCCTCTTTAGTCCGTGATCGCGGCATCTTCTCTCTTCTATTGCTGTTGGTTC<br>CTTCTCTTTTTGGGGCTTCTTCACAGGTTGCCCTTCACAGTGCGGGTGCGGAGTGCTATTCAAGTGAAGCCTGGACTACACCTGCGTTGCGTCCTATCCTAGTCCTCTCGTGTCCCTCGATGAGACGGTTTGCGTGTATGGGGAAT<br>CATCTTGACACTGATGCACTTTGGATCGCATTTGGTTATGGCTCTTCCACCCCCCC--<br>GGTAAATGGTGCTATTTAGTGAATGCTTGTCGGACATATTTTTATCAATTTTCACTTCCTCTATTTTCTTCACAAAACTAGGAAATTCACCACAATTTTTTCTTTGTTATTTTTTAATTTTTTTTTTATTTTTTAAAAACATTTTTTAAA<br>AAACTAAATTACATACAAACTACCGCATAAAATCCCTCAAACTATACAAACGTTTATCGTATAATATATATACATTATTGTTTATTCTATCATTATTAGAGAAACTCCACTACCAAAACCATCATTAAAACAAAAATTTACATGCCAC<br>TTAACTCCCCTCACAAACAATCGTTATTTATATTGTTAATTAGCAAACACAAAACCCGC
CamHap_26	ATTTTATTTTTTAACCTAACTCCCCTACTAAGTGTACCCCCCCTTTCCCCCCCAGGGGGGGTATACTATGCATAATCGTGCATACATTTATATACCACATATATTATGGTACCGGTAATATATACTATATATGTACTAAACCCATTAT<br>ATGTATACGGGCATTAATCTATATTCCACATTTCTCCCAATGTCCATTCTATGCATGATCCAGGACATACTCATTCACCCTCCCCATAGACAGCTCCAAACCACTACCAAGTCACCTAACTATGAATGGTTGCAGGACATAAATCTC<br>ACTCTCATGCTCTTCCCCCAACAAGTCACCTAACTATGAATGGTTGCAGGACATACATTTAACTACC--<br>ATGTTCTAACCCATTTGGTTATGCTCGCCGTATCAGATGGATTTATTGATCGTCCACCTCACGAGAGATCAGCAACCCCTGCCTGTAATGTACTTCATGACCAGTCTCAGGCCCATTCTTTCCCCCTACACCCCTCGCCCTACTTG<br>CCTTCCACCGTACCTCTGGTTCCTCGGTCAGGCACATCCCATGCATAACTCCTGAACTTTCTCACTTTTCACGAAGTCATCTGTGGATTATCTTCCCCTCTTTAGTCCGTGATCGCGGCATCTTCTCTCTTCTATTGCTGTTGGTTC<br>CTTCTCTTTTTGGGGCTTCTTCACAGGTTGCCCTTCACAGTGCGGGTGCGGAGTGCTATTCAAGTGAAGCCTGGACTACACCTGCGTTGCGTCCTATCCTAGTCCTCTCGTGTCCCTCGATGAGACGGTTTGCGTGTATGGGGAAT<br>CATCTTGACACTGATGCACTTTGGATCGCATTTGGTTATGGTTCTTCCACCCCCCC--<br>GGTAAATGGTGCTATTTAGTGAATGCTTGTCGGACATATTTTTATCAATTTTCACTTCCTCTATTTTCTTCACAAAACTAGGAAATTCACCACAATTTTTTCTTTGTTATTTTTTAATTTTTTTTTTATTTTTTAAAAACATTTTTTAAA<br>AAACTAAATTACATACAAACTACCGCATAAAATCCCTCAAACTATACAAACGTTTATCGTATAATATATATACATTATTGTTTATTCTATCATTATTAGAGAAACTCCACTACCAAAACCATCATTAAAACAAAAATTTACATGCCAC<br>TTAACTCCCCTCACAAACAATCGTTATTTATATTGTTAATTAGCAAACACAAAACCCGC
CamHap_27	ATTTTATTTTTTAACCTAACTCCCCTACTAAGTGTACCCCCCCTTTCCCCCCCAGGGGGGGTATACTATGCATAATCGTGCATACATTTATATACCACATATATTATGGTACCGGTAATATATACTATATATGTACTAAACCCATTAT<br>ATGTATACGGGCATTAATCTATATTCCACATTTCTCCCAATGTCCATTCTATGCATGATCCAGGACATACTCATTTACCCTCCCCATAGACAGTTCCAAACCACTATCAAGCCACCTAACTATGAATGGTTACAGGACATAAATCTC<br>ACTCTCATGTTCTCCCCCCAACAAGTCACCTAACTATGAATGGTTACAGGACATACATTTAACTACC--<br>ATGTTCTAACCCATTTGGTTATGCTCGCCGTATCAGATGGATTTATTGATCGTCCACCTCACGAGAGATCAGCAACCCCTGCCTGTAATGTACTTCATGACCAGTCTCAGGCCCATTCTTTCCCCCTACACCCCTCGCCCTACTTG<br>CCTTCCACCGTACCTCTGGTTCCTCGGTCAGGCACATCCCATGCATAACTCCTGAACTTTCTCACTTTTCACGAAGTCATCTGTGGATTATCTTCCCCTCTTTAGTCCGTGATCGCGGCATCTTCTCTCTTCTATTGCTGTTGGTTC<br>CTTCTCTTTTTGGGGCTTCTTCACAGGTTGCCCTTCACAGTGCGGGTGCGGAGTGCTATTCAAGTGAAGCCTGGACTACACCTGCGTTGCGTCCTATCCTAGTCCTCTCGTGTCCCTCGATGAGACGGTTTGCGTGTATGGGGAAT<br>CATCTTGACACTGATGCACTTTGGATCGCATTTGGTTATGGTTCTTCCACCCCCCC--<br>GGTAAATGGTGCTATTTAGTGAATGCTTGTCGGACATATTTTTATCAATTTTCACTTCCTCTATTTTCTTCACAAAACTAGGAAATTCACCACAATTTTTTCTTTGTTATTTTTTAATTTTTTTTTTATTTTTTAAAAACATTTTTTAAA<br>AAACTAAATTACATACAAACTACCGCATAAAATCCCTCAAACTATACAAACGTTTATCGTATAATATATATACATTATTGTTTATTCTATCATTATTAGAGAAACTCCACTACCAAAACCATCATTAAAACAAAAATTTACATGCCAC<br>TTAACTCCCCTCACAAACAATCGTTATTTATATTGTTAATTAGCAAACACAAAACCCGC
CamHap_28	ATTTTATTTTTTAACCTAACTCCCCTACTAAGTGTACCCCCCCTTTCCCCCCCAGGGGGGGTATACTATGCATAATCGTGCATACATTTATATACCACATATATTATGGTACCGGTAATATATACTATATATGTACTAAACCCATTAT<br>ATGTATACGGGCATTAACCTATATTCCACATTTCTCCCAATGTCCATTCTATGCATGATCCAGGACATACTCATTTACCCTCCCCATAGACAGTTCCAAACCACTATCAAGCCACCTAACTATGAATGGTTACAGGACATAAATCTC<br>ACTCTCATGTTCTTCCCCCAACAAGTCACCTAACTATGAATGGTTACAGGACATACATTTAACTACC--<br>ATGTTCTAACCCATTTGGTTATGCTCGCCGTATCAGATGGATTTATTGATCGTCCACCTCACGAGAGATCAGCAACCCCTGCCTGTAATGTACTTCATGACCAGTCTCAGGCCCATTCTTTCCCCCTACACCCCTCGCCCTACTTG<br>CCTTCCACCGTACCTCTGGTTCCTCGGTCAGGCACATCCCATGCATAACTCCTGAACTTTCTCACTTTTCACGAAGTCATCTGTGGATTATCTTCCCCTCTTTAGTCCGTGATCGCGGCATCTTCTCTCTTCTATTGCTGTTGGTTC<br>CTTCTCTTTTTGGGGCTTCTTCACAGGTTGCCCTTCACAGTGCGGGTGCGGAGTGCTATTCAAGTGAAGCCTGGACTACACCTGCGTTGCGTCCTATCCTAGTCCTCTCGTGTCCCTCGATGAGACGGTTTGCGTGTATGGGGAAT<br>CATCTTGACACTGATGCACTTTGGATCGCATTTGGTTATGGTTCTTCCACCCCCCC--<br>GGTAAATGGTGCTATTTAGTGAATGCTTGTCGGACATATTTTTATCAATTTTCACTTCCTCTATTTTCTTCACAAAACTAGGAAATTCACCACAATTTTTTCTTTGTTATTTTTTAATTTTTTTTTTATTTTTTAAAAACATTTTTTAAA<br>AAACTAAATTACATACAAACTACCGCATAAAATCCCTCAAACTATACAAACGTTTATCGTATAATATATATACATTATTGTTTATTCTATCATTATTAGAGAAACTCCACTACCAAAACCATCATTAAAACAAAAATTTACATGCCAC<br>TTAACTCCCCTCACAAACAATCGTTATTTATATTGTTAATTAGCAAACACAAAACCCGC

CamHap_29	ATTTTATTTTTTAACCTAACTCCCCTACTAAGTGTACCCCCCCTTTCCCCCCCAGGGGGGGTATACTATGCATAATCGTGCATACATTTATATACCACATATATTATGGTACCGGTAATATATACTATATATGTACTAAACCCATTAT<br>ATGTATACGGGCATTAATCTATATTCCACATTTCTCCCAATGTCCATTCTATGCATGATCCAGGACATACTCATTCACCCTCCCCATAGACAGCTCCAAACCACTACCAAGTCACCTAACTATGAATGGTTGCAGGACATAAATCTC<br>ACTCTCATGCTCTTCCCCCAACAAGTCACCTAACTATGAATGGTTACAGGGCATACATTTAACTACC--<br>ATGTTCTAACCCATTTGGTTATGCTCGCCGTATCAGATGGATTTATTGATCGTCCACCTCACGAGAGATCAGCAACCCCTGCCTGTAATGTACTTCATGACCAGTCTCAGGCCCATTCTTTCCCCCTACACCCCTCGCCCTACTTG<br>CCTTCCACCGTACCTCTGGTTCCTCGGTCAGGCACATCCCATGCATAACTCCTGAACTTTCTCACTTTTCACGAAGTCATCTGTGGATTATCTTCCCCTCTTTAGTCCGTGATCGCGGCATCTTCTCTCTTCTATTGCTGTTGGTTC<br>CTTCTCTTTTTGGGGCTTCTTCACAGGTTGCCCTTCACAGTGCGGGTGCGGAGTGCTATTCAAGTGAAGCCTGGACTACACCTGCGTTGCGTCCTATCCTAGTCCTCTCGTGTCCCTCGATGAGACGGTTTGCGTGTATGGGGAAT<br>CATCTTGACACTGATGCACTTTGGATCGCATTTGGTTATGGTTCTTCCACCCCCCCC-<br>GGTAAATGGTGCTATTTAGTGAATGCTTGTCGGACATATTTTTATCAATTTTCACTTCCTCTATTTTCTTCACAAAACTAGGAAATTCACCACAATTTTTTCTTTGTTATTTTTTAATTTTTTTTTTATTTTTTAAAAACATTTTTTAAA<br>AAACTAAATTACATACAAACTACCGCATAAAATCCCTCAAACTATACAAACGTTTATCGTATAATATATATACATTATTGTTTATTCTATCATTATTAGAGAAACTCCACTACCAAAACCATCATTAAAACAAAAATTTACATGCCAC<br>TTAACTCCCCTCACAAACAATCGTTATTTATATTGTTAATTAGCAAACACAAAACCCGC
CamHap_30	ATTTTATTTTTTAACCTAACTCCCCTACTAAGTGTACCCCCCCTTTCCCCCCCAGGGGGGGTATACTATGCATAATCGTGCATACATTTATATACCACATATATTATGGTACCGGTAATATATACTATATATGTACTAAACCCATTAT<br>ATGTATACGGGCATTAATCTATATTCCACATTTCTCCCAATGTCCATTCTATGCATGATCCAAGACATACTCATTCACCCTCCCCATAGACAGTTCTAAACCACTATCAAGCCACCTAACTATGAATGGTTACAGGACATAAATCTC<br>ACTCTCATGTTCTCCCCCTAACAAGTCACCTAACTATGAATGGTTACAGGACATACATTTAACTACC--<br>ATGTTCTAACCCATTTGGTTATGCTCGCCGTATCAGATGGATTTATTGATCGTCCACCTCACGAGAGATCAGCAACCCCTGCCTGTAATGTACTTCATGACCAGTCTCAGGCCCATTCTTTCCCCCTACACCCCTCGCCCTACTTG<br>CCTTCCACCGTACCTCTGGTTCCTCGGTCAGGCACATCCCATGCATAACTCCTGAACTTTCTCACTTTTCACGAAGTCATCTGTGGATTATCTTCCCCTCTTTAGTCCGTGATCGCGGCATCTTCTCTCTTCTATTGCTGTTGGTTC<br>CTTCTCTTTTTGGGGCTTCTTCACAGGTTGCCCTTCACAGTGCGGGTGCGGAGTGCTATTCAAGTGAAGCCTGGACTACACCTGCGTTGCGTCCTATCCTAGTCCTCTCGTGTCCCTCGATGAGACGGTTTGCGTGTATGGGGAA<br>TCATCTTGACACTGATGCACTTTGGATCGCATTTGGTTATGGTTCTTCCACCCCCCC--<br>GGTAAATGGTGCTATTTAGTGAATGCTTGTCGGACATATTTTTATCAATTTTCACTTCCTCTATTTTCTTCACAAAACTAGGAAATTCACCACAATTTTTTCTTTGTTATTTTTTAATTTTTTTTTTATTTTTTAAAAACATTTTTTAAA<br>AAACTAAATTACATACAAACTACCGCATAAAATCCCTCAAACTATACAAACGTTTATCGTATAATATATATACATTATTGTTTATTCTATCATTATTAGAGAAACTCCACTACCAAAACCATCATTAAAACAAAAATTTACATGCCAC<br>TTAACTCCCCTCACAAACAATCGTTATTTATATTGTTAATTAGCAAACACAAAACCCAC
CamHap_31	ATTTTATTTTTTAACCTAACTCCCCTACTAAGTGTACCCCCCCTTTCCCCCCCAGGGGGGGTATACTATGCATAATCGTGCATACATTTATATACCACATATATTATGGTACCGGTAATATATACTATATACGTACTAAACCCATTAT<br>ATGTATACGGGCATTAATCTATATTCCACATTTCTCCCAATGTCCATTCTATGCATGATCCAGGACATACTCATTCACCCTCCCCATAGACAGCTCCAAACCACTACCAAGTCACCTAACTATGAATGGTTGCAGGACATAAATCTC<br>ACTCTCATGCTCTTCCCCCAACAAGTCACCTAACTATGAATGGTTACAGGACATACATTTAACTACC--<br>ATGTTCTAACCCATTTGGTTATGCTCGCCGTATCAGATGGATTTATTGATCGTCCACCTCACGAGAGATCAGCAACCCCTGCCTGTAATGTACTTCATGACCAGTCTCAGGCCCATTCTTTCCCCCTACACCCCTCGCCCTACTTG<br>CCTTCCACCGTACCTCTGGTTCCTCGGTCAGGCACATCCCATGCATAACTCCTGAACTTTCTCACTTTTCACGAAGTCATCTGTGGATTATCTTCCCCTCTTTAGTCCGTGATCGCGGCATCTTCTCTCTTCTATTGCTGTTGGTTC<br>CTTCTCTTTTTGGGGCTTCTTCACAGGTTGCCCTTCACAGTGCGGGTGCGGAGTGCTATTCAAGTGAAGCCTGGACTACACCTGCGTTGCGTCCTATCCTAGTCCTCTCGTGTCCCTCGATGAGACGGTTTGCGTGTATGGGGAAT<br>CATCTTGACACTGATGCACTTTGGATCGCATTTGGTTATGGTTCTTCCACCCCCCC--<br>GGTAAATGGTGCTATTTAGTGAATGCTTGTCGGACATATTTTTATCAATTTTCACTTCCTCTATTTTCTTCACAAAACTAGGAAATTCACCACAATTTTTTCTTTGTTATTTTTTAATTTTTTTTTTATTTTTTAAAAACATTTTTTAAA<br>AAACTAAATTACATACAAACTACCGCATAAAATCCCTCAAACTATACAAACGTTTATCGTATAATATATATACATTATTGTTTATTCTATCATTATTAGAGAAACTCCACTACCAAAACCATCATTAAAACAAAAATTTACATGCCAC<br>TTAACTCCCCTCACAAACAATCGTTATTTATATTGTTAATTAGCAAACACAAAACCCGC
CamHap_32	ATTTTATTTTTTAACCTAACTCCCCTACTAAGTGTACCCCCCCTTTCCCCCCCAGGGGGGGTATACTATGCATAATCGTGCATACATTTATATACCACATATATTATGGTACCGGTAATATATACTATATATGTACTAAACCCATTAT<br>ATGTATACGGGCATTAACCTATATTCCACATTTCTCCCAATGTCCATTCTATGCATGATCCAGGACATACTCATTTACCCTCCCCATAGGCAGTTCCAAACCACTATCAAGCCACCTAACTATGAATGGTTACAGGACATAAATCTC<br>ACTCTCATGTTCTCCCCCCAACAAGTCACCTAACTATGAATGGTTACAGGACATACATTTAACTACC--<br>ATGTTCTAACCCATTTGGTTATGCTCGCCGTATCAGATGGATTTATTGATCGTCCACCTCACGAGAGATCAGCAACCCCTGCCTGTAATGTACTTCATGACCAGTCTCAGGCCCATTCTTTCCCCCTACACCCCTCGCCCTACTTG<br>CCTTCCACCGTACCTCTGGTTCCTCGGTCAGGCACATCCCATGCATAACTCCTGAACTTTCTCACTTTTCACGAAGTCATCTGTGGATTATCTTCCCCTCTTTAGTCCGTGATCGCGGCATCTTCTCTCTTCTATTGCTGTTGGTTC<br>CTTCTCTTTTTGGGGCTTCTTCACAGGTTGCCCTTCACAGTGCGGGTGCGGAGTGCTATTCAAGTGAAGCCTGGACTACACCTGCGTTGCGTCCTATCCTAGTCCTCTCGTGTCCCTCGATGAGACGGTTTGCGTGTATGGGGAA<br>TCATCTTGACACTGATGCACTTTGGATCGCATTTGGTTATGGTTCTTCCACCCCCCC--<br>GGTAAATGGTGCTATTTAGTGAATGCTTGTCGGACATATTTTTATCAATTTTCACTTCCTCTATTTTCTTCACAAAACTAGGAAATTCACCACAATTTTTTCTTTGTTATTTTTTAATTTTTTTTTTATTTTTTAAAAACATTTTTTAAA<br>AAACTAAATTACATACAAACTACCGCATAAAATCCCTCAAACTATACAAACGTTTATCGTATAATATATATACATTATTGTTTATTCTATCATTATTAGAGAAACTCCACTACCAAAACCATCATTAAAACAAAAATTTACATGCCAC<br>TTAACTCCCCTCACAAACAATCGTTATTTATATTGTTAATTAGCAAACACAAAACCCGC

CamHap_33	ATTTTATTTTTTAACCTAACTCCCCTACTAAGTGTACCCCCCCTTTCCCCCCCAGGGGGGGTATACTATGCATAATCGTGCATACATTTATATACCACATATATTATGGTACCGGTAATATATACTATATATGTACTAAACCCATTAT<br>ATGTATACGGGCATTAATCTATATTCCACATTTCTCCCAATGTCCATTCTATGCATGATCCAGGACACACTCATTCACCCTCCCCATAGACAGCTCCAAACCACTACCAAGTCACCTAACTATGAATGGTTACAGGACATAAATCT<br>CACTCTCATGTTCTTCCCCCAACAAGTCACCTAACTATGAATGGTTACAGGACATACATTTAACTACC--<br>ATGTTCTAACCCATTTGGTTATGCTCGCCGTATCAGATGGATTTATTGATCGTCCACCTCACGAGAGATCAGCAACCCCTGCTTGTAATGTACTTCATGACCAGTCTCAGGCCCATTCTTTCCCCCTACACCCCTCGCCCTACTTG<br>CCTTCCACCGTACCTCTGGTTCCTCGGTCAGGCACATCCCATGCATAACTCCTGAACTTTCTCACTTTTCACGAAGTCATCTGTGGATTATCTTCCCCTCTTTAGTCCGTGATCGCGGCATCTTCTCTCTTCTATTGCTGTTGGTTC<br>CTTCTCTTTTTGGGGCTTCTTCACAGGTTGCCCTTCACAGTGCGGGTGCGGAGTGCTATTCAAGTGAAGCCTGGACTACACCTGCGTTGCGTCCTATCCTAGTCCTCTCGTGTCCCTCGATGAGACGGTTTGCGTGTATGGGGAAT<br>CATCTTGACACTGATGCACTTTGGATCGCATTTGGTTATGGTTCTTCCACCCCCCC--<br>GGTAAATGGTGCTATTTAGTGAATGCTTGTCGGACATATTTTTATCAATTTTCACTTCCTCTATTTTCTTCACAAAACTAGGAAATTCACCACAATTTTTTCTTTGTTATTTTTTAATTTTTTTTTTATTTTTTAAAAACATTTTTTAAA<br>AAACTAAATTACATACAAACTACCGCATAAAATCCCTCAAACTATACAAACGTTTATCGTATAATATATATACATTATTGTTTATTCTATCATTATTAGAGAAACTCCACTACCAAAACCATCATTAAAACAAAAATTTACATGCCAC<br>TTAACTCCCCTCACAAACAATCGTTATTTATATTGTTAATTAGCAAACACAAAACCCGC
CamHap_34	ATTTTATTTTTTAACCTAACTCCCCTACTAAGTGTACCCCCCCTTTCCCCCCCAGGGGGGGTATACTATGCATAATCGTGCATACATTTATATACCACATATATTATGGTACCGGTAATATATACTATATATGTACTAAACCCATTAT<br>ATGTATACGGGCATTAATCTATATTCCACATTTCTCCCAATGTCCATTCTATGCATGATCCAGGACATACTCATTCACCCTCCCTACAGACAGCTCCAAACCACCACCAAGTCACCTAACTATGAATGGTTACAGGACATAAATCT<br>CACTCTCATGTTCTTCCCCTACCAAGTCACCTAACTATGAATGGTTACAGGACATACATTTAACTACC--<br>ATGTTCTAACCCATTTGGTTATGCTCGCCGTACCAGATGGATTTATTGATCGTCCACCTCACGAGAGATCAGCAACCCCTGCTTGTAATGTACTTCATGACCAGTCTCAGGCCCATTCTTTCCCCCTACACCCCTCGCCCTACTTG<br>CCTTCCACCGTACCTCTGGTTCCTCGGTCAGGCACATCCCATGCATAACTCCTGAACTTTCTCACTTTTCACGAAGTCATCTGTGGATTATCTTCCCCTCTTTAGTCCGTGATCGCGGCATCTTCTCTCTTCTATTGCTGTTGGTTC<br>CTTCTCTTTTTGGGGCTTCTTCACAGGTTGCCCTTCACAGTGCGGGTGCGGAGTGCTATTCAAGTGAAGCCTGGACTACACCTGCGTTGCGTCCTATCCTAGTCCTCTCGTGTCCCTCGATGAGACGGTTTGCGTGTATGGGGAAT<br>CATCTTGACACTGATGCACTTTGGATCGCATTTGGTTATGGTTCTTCCACCCCCCC--<br>GGTAAATGGTGCTATTTAGTGAATGCTTGTCGGACATATTTTTACCAATTTTCACTTCCTCTATTTTCTTCACAAAACTAGGAAATTCACCACAATTTTTTCTTTGTTATTTTTTAATTTTTTTTTTATTTTTTAAAAACATTTTTTAAA<br>AAACTAAATTACATACAAACTACCGCATAAAATCCCTCAAACTATACAAACGTTTATCGTATAATATATATACATTATTGTTTATTCTATCATTATTAGAGAAACTCCACTACCAAAACCATCATTAAAACAAAAATTTACATGCCAC<br>TTAACTCCCCTCACAAACAATCGTTATTTATATTGTTAATTAGCAAACACAAAACCTGC
CamHap_35	ATTTTATTTTTTAACCTAACTCCCCTACTAAGTGTACCCCCCCTTTCCCCCCCAGGGGGGGTATACTATGCATAATCGTGCATACATTTATATACCACATATATTATGGTACCGGTAATATATACTATATATGTACTAAACCCATTAT<br>ATGTATACGGGCATTAATCTATATTCCACATTTCTCCCAATGTCCATTCTATGCATGATCCAGGACATACTCATTCACCCTCCCTACAGACAGCTCCAAACCACCACCAAGTCACCTAACTATGAATGGTTACAGGACATAAATCT<br>CACTCTCATGTTCTTCCCCTACCAAGTCACCTAACTATGAATGGTTACAGGACATACATTTAACTACC--<br>ATGTTCTAACCCATTTGGTTATGCTCGCCGTACCAGATGGATTTATTGATCGTCCACCTCACGAGAGATCAGCAACCCCTGCTTGTAATGTACTTCATGACCAGTCTCAGGCCCATTCTTTCCCCCTACACCCCTCGCCCTACTTG<br>CCTTCCACCGTACCTCTGGTTCCTCGGTCAGGCACATCCCATGCATAACTCCTGAACTTTCTCACTTTTCACGAAGTCATCTGTGGATTATCTTCCCCTCTTTAGTCCGTGATCGCGGCATCTTCTCTCTTCTATTGCTGTTGGTTC<br>CTTCTCTTTTTGGGGCTTCTTCACAGGTTGCCCTTCACAGTGCGGGTGCGGAGTGCTATTCAAGTGAAGCCTGGACTACACCTGCGTTGCGTCCTATCCTAGTCCTCTCGTGTCCCTCGATGAGACGGTTTGCGTGTATGGGGAAT<br>CATCTTGACACTGATGCACTTTGGATCGCATTTGGTTATGGTTCTTCCACCCCCCC--<br>GGTAAATGGTGCTATTTAGTGAATGCTTGTCGGACATATTTTTACCAATTTTCACTTCCTCTATTTTCTTCACAAAACTAGGAAATTCACCACAATTTTTTCTTTGTTATTTTTTAATTTTTTTTTTATTTTTTAAAAACATTTTTTAAA<br>AAACTAAATTACATACAAACTACCGCATAAAATCCCTCAAACTATACAAACGTTTATCGTATAATATATATACATTATTGTTTATTCTATCATTATTAGAGAAACTCCACTACCAAAACCATCATTAAAACAAAAATTTACATGCCAC<br>TTAACTCCCCTCACAAACAATCGTTATTTATATTGTTAATTAGCAAACACAAAACCCGC
CamHap_36	ATTTTATTTTTTAACCTAACTCCCCTACTAAGTGTACCCCCCCTTTCCCCCCCAGGGGGGGTATACTATGCATAATCGTGCATACATTTATATACCACATATATTATGGTACCGGTAATATATACTATATATGTACTAAACCCATTAT<br>ATGTATACGGGCATTAACCTATATTCCACATTTCTCCCAATGTCCATTCTATGCATGATCCAGGACATACTCATTTACCCTCCCCATAGACAGTTCCAAACCACTATCAAGCCACCTAACTATGAATGGTTACAGGACATAAATCTC<br>ACTCTCATGTTCTCCCCCCAACAAGTCACCTAACTATGAATGGTTACAGGACATACATTTAACTACC--<br>ATGTTCTAACCCATTTGGTTATGCTCGCCGTACCAGATGGATTTATTGATCGTCCACCTCACGAGAGATCAGCAACCCCTGCCTGTAATGTACTTCATGACCAGTCTCAGGCCCATTCTTTCCCCCTACACCCCTCGCCCTACTTG<br>CCTTCCACCGTACCTCTGGTTCCTCGGTCAGGCACATCCCATGCATAACTCCTGAACTTTCTCACTTTTCACGAAGTCATCTGTGGATTATCTTCCCCTCTTTAGTCCGTGATCGCGGCATCTTCTCTCTTCTATTGCTGTTGGTTC<br>CTTCTCTTTTTGGGGCTTCTTCACAGGTTGCCCTTCACAGTGCGGGTGCGGAGTGCTATTCAAGTGAAGCCTGGACTACACCTGCGTTGCGTCCTATCCTAGTCCTCTCGTGTCCCTCGATGAGACGGTTTGCGTGTATGGGGAAT<br>CATCTTGACACTGATGCACTTTGGATCGCATTTGGTTATGGTTCTTCCACCCCCCC--<br>GGTAAATGGTGCTATTTAGTGAATGCTTGTCGGACATATTTTTATCAATTTTCACTTCCTCTATTTTCTTCACAAAACTAGGAAATTCACCACAATTTTTTCTTTGTTATTTTTTAATTTTTTTTTTATTTTTTAAAAACATTTTTTAAA<br>AAACTAAATTACATACAAACTACCGCATAAAATCCCTCAAACTATACAAACGTTTATCGTATAATATATATACATTATTGTTTATTCTATCATTATTAGAGAAACTCCACTACCAAAACCATCATTAAAACAAAAATTTACATGCCAC<br>TTAACTCCCCTCACAAACAATCGTTATTTATATTGTTAATTAGCAAACACAAAACCCGC

CamHap_37	ATTTTATTTTTTAACCTAACTCCCCTACTAAGTGTACCCCCCCTTTCCCCCCCAGGGGGGGTATACTATGCATAATCGTGCATACATTTATATACCACATATATTATGGTACCGGTAATATATACTATATATGTACTAAACCCATTAT<br>ATGTATACGGGCATTAACCTATATTCCACATTTCTCCCAATGTCCATTCTATGCATGATCCAGGACATACTCATTTACCCTCCCCATAGACAGTTCCAAACCACTATCAAGCCACCTAACTATGAATGGTTACAGGACATAAATCTC<br>ACTCTCATGTTCTCCCCCCAACAAGTCACCTAACTATGAATGGTTACAGGACATACATTTAACTACC---<br>ATGTTCTAGCCCATTTGGTTATGCTCGCCGTATCAGATGGATTTATTGATCGTCCACCTCACGAGAGATCAGCAACCCCTGCCTGTAATGTACTTCATGACCAGTCTCAGGCCCATTCTTTCCCCCTACACCCCTCGCCCTACTTG<br>CCTTCCACCGTACCTCTGGTTCCTCGGTCAGGCACATCCCATGCATAACTCCTGAACTTTCTCACTTTTCACGAAGTCATCTGTGGATTATCTTCCCCTCTTTAGTCCGTGATCGCGGCATCTTCTCTCTTCTATTGCTGTTGGTTC<br>CTTCTCTTTTTGGGGCTTCTTCACAGGTTGCCCTTCACAGTGCGGGTGCGGAGTGCTATTCAAGTGAAGCCTGGACTACACCTGCGTTGCGTCCTATCCTAGTCCTCTCGTGTCCCTCGATGAGACGGTTTGCGTGTATGGGGAA<br>TCATCTTGACACTGATGCACTTTGGATCGCATTTGGTTATGGTTCTTCCACCCCCCC--<br>GGTAAATGGTGCTATTTAGTGAATGCTTGTCGGACATATTTTTATCAATTTTCACTTCCTCTATTTTCTTCACAAAACTAGGAAATTCACCACAATTTTTTCTTTGTTATTTTTTAATTTTTTTTTTATTTTTTAAAAACATTTTTTAAA<br>AAACTAAATTACATACAAACTACCGCATAAAATCCCTCAAACTATACAAACGTTTATCGTATAATATATATACATTATTGTTTATTCTATCATTATTAGAGAAACTCCACTACCAAAACCATCATTAAAACAAAAATTTACATGCCAC<br>TTAACTCCCCTCACAAACAATCGTTATTTATATTGTTAATTAGCAAACACAAAACCCGC
CamHap_38	ATTTTATTTTTTAACCTAACTCCCCTACTAAGTGTACCCCCCCTTTCCCCCCCAGGGGGGGTATACTATGCATAATCGTGCATACATTTATATACCACATATATTATGGTACCGGTAATATATACTATATATGTACTAAACCCATTAT<br>ATGTATACGGGCATTAATCTATATTCCACATTTCTCCCAATGTCCATTCTATGCATGATCCAGGACATACCCATTCACCCTCCCCATAGACAGCTCCAAACCACTACCAAGTCACCTAACTATGAATGGTTGCAGGACATAAATCT<br>CACTCTCATGCTCTTCCCCCAACAAGTCACCTAACTATGAATGGTTACGGGACATACATTTAACTACC---<br>ATGTTCTAACCCATTTGGTTATGCTCGCCGTATCAGATGGATTTATTGATCGTCCACCTCACGAGAGATCAGCAACCCCTGCCTGTAATGTACTTCATGACCAGTCTCAGGCCCATTCTTTCCCCCTACACCCCTCGCCCTACTTG<br>CCTTCCACCGTACCTCTGGTTCCTCGGTCAGGCACATCCCATGCATAACTCCTGAACTTTCTCACTTTTCACGAAGTCATCTGTGGATTATCTTCCCCTCTTTAGTCCGTGATCGCGGCATCTTCTCTCTTCTATTGCTGTTGGTTC<br>CTTCTCTTTTTGGGGCTTCTTCACAGGTTGCCCTTCACAGTGCGGGTGCGGAGTGCTATTCAAGTGAAGCCTGGACTACACCTGCGTTGCGTCCTATCCTAGTCCTCTCGTGTCCCTCGATGAGACGGTTTGCGTGTATGGGGAAT<br>CATCTTGACACTGATGCACTTTGGATCGCATTTGGTTATGGTTCTTCCACCCCCCC--<br>GGTAAATGGTGCTATTTAGTGAATGCTTGTCGGACATATTTTTATCAATTTTCACTTCCTCTATTTTCTTCACAAAACTAGGAAATTCACCACAATTTTTTCTTTGTTATTTTTTAATTTTTTTTTTATTTTTTAAAAACATTTTTTAAA<br>AAACTAAATTACATACAAACTACCGCATAAAATCCCTCAAACTATACAAACGTTTATCGTATAATATATATACATTATTGTTTATTCTATCATTATTAGAGAAACTCCACTACCAAAACCATCATTAAAACAAAAATTTACATGCCAC<br>TTAACTCCCCTCACAAACAATCGTTATTTATATTGTTAATTAGCAAACACAAAACCCGC
CamHap_39	ATTTTATTTTTTAACCTAACTCCCCTACTAAGTGTACCCCCCCTTTCCCCCCCAGGGGGGGTATACTATGCATAATCGTGCATACATTTATATACCACATATATTATGGTACCGGTAATATATACTATATATGTACTAAACCCATTAT<br>ATGTATACGGGCATTAACCTATATTCCACATTTCTCCCAATGTCCATTCTATGCATGATCCGGGACATACTCATTTACCCTCCCCATAGACAGTTCCAAACCACTATCAAGCCACCTAACTATGAATGGTTACAGGACATAAATCTC<br>ACTCTCATGCTCTCCCCCCAACAAGTCACCTAACTATGAATGGTTACAGGACATACATTTAACTACC---<br>ATGTTCTAACCCATTTGGTTATGCTCGCCGTATCAGATGGATTTATTGATCGTCCACCTCACGAGAGATCAGCAACCCCTGCCTGTAATGTACTTCATGACCAGTCTCAGGCCCATTCTTTCCCCCTACACCCCTCGCCCTACTTG<br>CCTTCCACCGTACCTCTGGTTCCTCGGTCAGGCACATCCCATGCATAACTCCTGAACTTTCTCACTTTTCACGAAGTCATCTGTGGATTATCTTCCCCTCTTTAGTCCGTGATCGCGGCATCTTCTCTCTTCTATTGCTGTTGGTTC<br>CTTCTCTTTTTGGGGCTTCTTCACAGGTTGCCCTTCACAGTGCGGGTGCGGAGTGCTATTCAAGTGAAGCCTGGACTACACCTGCGTTGCGTCCTATCCTAGTCCTCTCGTGTCCCTCGATGAGACGGTTTGCGTGTATGGGGAAT<br>CATCTTGACACTGATGCACTTTGGATCGCATTTGGTTATGGTTCTTCCACCCCCCC--<br>GGTAAATGGTGCTATTTAGTGAATGCTTGTCGGACATATTTTTATCAATTTTCACTTCCTCTATTTTCTTCACAAAACTAGGAAATTCACCACAATTTTTTCTTTGTTATTTTTTAATTTTTTTTTTATTTTTTAAAAACATTTTTTAAA<br>AAACTAAATTACATACAAACTACCGCATAAAATCCCTCAAACTATACAAACGTTTATCGTATAATATATATACATTATTGTTTATTCTATCATTATTAGAGAAACTCCACTACCAAAACCATCATTAAAACAAAAATTTACATGCCAC<br>TTAACTCCCCTCACAAACAATCGTTATTTATATTGTTAATTAGCAAACACAAAACCCGC
CamHap_40	ATTTTATTTTTTAACCTAACTCCCCTACTAAGTGTACCCCCCCTTTCCCCCCCAGGGGGGGTATACTATGCATAATCGTGCATACATTTATATACCACATATATTATGGTACCGGTAATATATACTATATATGTACTAAACCCATTAT<br>ATGTATACGGGCATTAATCTATATTCCACATTTCTCCCAATGTCCATTCTATGCATGATCCAAGACATACTCATTCACCCTCCCCATAGACAGTTCTAAACCACTATCAAGCCACCTAACTATGAATGGTTACAGGACATAAATCTT<br>ACTCTCATGTTCTCCCCCTAACAAGTCACCTAACTATGAATGGTTACAGGACATACATTTAACTACC---<br>ATGTTCTAACCCATTTGGTTATGCTCGCCGTATCAGATGGATTTATTGATCGTCCACCTCACGAGAGATCAGCAACCCCTGCCTGTAATGTACTTCATGACCAGTCTCAGGCCCATTCTTTCCCCCTACACCCCTCGCCCTACTTG<br>CCTTCCACCGTACCTCTGGTTCCTCGGTCAGGCACATCCCATGCATAACTCCTGAACTTTCTCACTTTTCACGAAGTCATCTGTGGATTATCTTCCCCTCTTTAGTCCGTGATCGCGGCATCTTCTCTCTTCTATTGCTGTTGGTTC<br>CTTCTCTTTTTGGGGCTTCTTCACAGGTTGCCCTTCACAGTGCGGGTGCGGAGTGCTATTCAAGTGAAGCCTGGACTACACCTGCGTTGCGTCCTATCCTAGTCCTCTCGTGTCCCTCGATGAGACGGTTTGCGTATATGGGGAAT<br>CATCTTGACACTGATGCACTTTGGATCGCATTTGGTTATGGTTCTTCCACCCCCCC--<br>GGTAAATGGTGCTATTTAGTGAATGCTTGTCGGACATATTTTTATCAATTTTCACTTCCTCTATTTTCTTCACAAAACTAGGAAATTCACCACAATTTTTTCTTTGTTATTTTTTAATTTTTTTTTTATTTTTTAAAAACATTTTTTAAA<br>AAACTAAATTACATACAAACTACCGCATAAAATCCCTCAAACTATACAAACGTTTATCGTATAATATATATACATTATTGTTTATTCTATCATTATTAGAGAAACTCCACTACCAAAACCATCATTAAAACAAAAATTTACATGCCAC<br>TTAACTCCCCTCACAAACAATCGTTATTTATATTGTTAATTAGCAAACACAAAACCCAC

CamHap_41	ATTTTATTTTTTAACCTAACTCCCCTACTAAGTGTACCCCCCCTTTCCCCCCCAGGGGGGGTATACTATGCATAATCGTGCATACATTTATATACCACATATATTATGGTACCGGTAATATATACTATATATGTACTAAACCCATTAT<br>ATGTATACGGGCATTAATCTATATTCCACATTTCTCCCAATGTCCATTCCATGCATGATCCAGGACACACTCATTCACCCTCCCCATAGACAGCTCCAAACCACTACCAAGTCACCTAACTATGAATGGTTACAGGACATAAATCT<br>CACTCTCATGTTCTTCCCCCAACAAGTCACCTAACTATGAATGGTTACAGGACATACATTTAACTACC---<br>ATGTTCTAACCCATTTGGTTATGCTCGCCGTATCAGATGGATTTATTGATCGTCCACCTCACGAGAGATCAGCAACCCCTGCTTGTAATGTACTTCATGACCAGTCTCAGGCCCATTCTTTCCCCCTACACCCCTCGCCCTACTTG<br>CCTTCCACCGTACCTCTGGTTCCTCGGTCAGGCACATCCCATGCATAACTCCTGAACTTTCTCACTTTTCACGAAGTCATCTGTGGATTATCTTCCCCTCTTTAGTCCGTGATCGCGGCATCTTCTCTCTTCTATTGCTGTTGGTTC<br>CTTCTCTTTTTGGGGCTTCTTCACAGGTTGCCCTTCACAGTGCGGGTGCGGAGTGCTATTCAAGTGAAGCCTGGACTACACCTGCGTTGCGTCCTATCCTAGTCCTCTCGTGTCCCTCGATGAGACGGTTTGCGTGTATGGGGAAT<br>CATCTTGACACTGATGCACTTTGGATCGCATTTGGTTATGGTTCTTCCACCCCCCC--<br>GGTAAATGGTGCTATTTAGTGAATGCTTGTCGGACATATTTTTATCAATTTTCACTTCCTCTATTTTCTTCACAAAACTAGGAAATTCACCACAATTTTTTCTTTGTTATTTTTTAATTTTTTTTTTATTTTTTAAAAACATTTTTTAAA<br>AAACTAAATTACATACAAACTACCGCATAAAATCCCTCAAACTATACAAACGTTTATCGTATAATATATATACATTATTGTTTATTCTATCATTATTAGAGAAACTCCACTACCAAAACCATCATTAAAACAAAAATTTACATGCCAC<br>TTAACTCCCCTCACAAACAATCGTTATTTATATTGTTAATTAGCAAACACAAAACCTGC
CamHap_42	ATTTTATTTTTTAACCTAACTCCCCTACTAAGTGTACCCCCCCTTTCCCCCCCAGGGGGGGTATACTATGCATAATCGTGCATACATTTATATACCACATATATTATGGTACCGGTAATATATACTATATATGTACTAAACCCATTAT<br>ATGTATACGGGCATTAATCTATATTCCACATTTCTCCCAATGTCCATTCTATGCATGATCCAAGACATACTCATTCACCCTCCCCATAGACAGTTCTAAACCACTATCAAGCCACCTAACTATGAATGGTTACAGGACATAAATCTC<br>ACTCTCATGTTCTCCCCCTAACAAGTCACCTAACTATGAATGGTTACAGGACATACATTTAACTACC---<br>ATGTTCTAACCCATTTGGTTATGCTCGCCGTATCAGATGGATTTATTGATCGTCCACCTCACGAGAGATCAGCAACCCCTGCCTGTAATGTACTTCATGACCAGTCTCAGGCCCATTCTTTCCCCCTACACCCCTCGCCCTACTTG<br>CCTTCCACCGTACCTCTGGTTCCTCGGTCAGGCACATCCCATGCATAACTCCTGAACTTTCTCACTTTTCACGAAGTCATCTGTGGATTATCTTCCCCTCTTTAGTCCGTGATCGCGGCATCTTCTCTCTTCTATTGCTGTTGGTTC<br>CTTCTCTTTTTGGGGCTTCTTCACAGGTTACCCTTCACAGTGCGGGTGCGGAGTGCTATTCAAGTGAAGCCTGGACTACACCTGCGTTGCGTCCTATCCTAGTCCTCTCGTGTCCCTCGATGAGACGGTTTGCGTATATGGGGAAT<br>CATCTTGACACTGATGCACTTTGGATCGCATTTGGTTATGGTTCTTCCACCCCCCC--<br>GGTAAATGGTGCTATTTAGTGAATGCTTGTCGGACATATTTTTATCAATTTTCACTTCCTCTATTTTCTTCACAAAACTAGGAAATTCACCACAATTTTTTCTTTGTTATTTTTTAATTTTTTTTTTATTTTTTAAAAACATTTTTTAAA<br>AAACTAAATTACATACAAACTACCGCATAAAATCCCTCAAACTATACAAACGTTTATCGTATAATGTATATACATTATTGTTTATTCTATCATTATTAGAGAAACTCCACTACCAAAACCATCATTAAAACAAAAATTTACATGCCAC<br>TTAACTCCCCTCACAAACAATCGTTATTTATATTGTTAATTAGCAAACACAAAACCCAC
CamHap_43	ATTTTATTTTTTAACCTAACTCCCCTACTAAGTGTACCCCCCCTTTCCCCCCCAGGGGGGGTATACTATGCATAATCGTGCATACATTTATATACCACATATATTATGGTACCGGTAATATATACTATATATGTACTAAACCCATTAT<br>ATGTATACGGGCATTAATCTATATTCCACATTTCTCCCAATGTCCATTCTATGCATGATCCAAGACATACTCATTCACCCTCCCCATAGACAGTTCTAAACCACTATCAAGCCACCTAACTATGAATGGTTACAGGACATAAATCTC<br>ACTCTCATGTTCTCCCCCTAACAAGTCACCTAACTATGAATGGTTACAGGACATACATTTAACTACC---<br>ATGTTCTAACCCATTTGGTTATGCTCGCCGTATCAGATGGATTTATTGATCGTCCACCTCACGAGAGATCAGCAACCCCTGCCTGTAATGTACTTCATGACCAGTCTCAGGCCCATTCTTTCCCCCTACACCCCTCGCCCTACTTG<br>CCTTCCACCGTACCTCTGGTTCCTCGGTCAGGCACATCCCATGCATAACTCCTGAACTTTCTCACTTTTCACGAAGTCATCTGTGGATTATCTTCCCCTCTTTAGTCCGTGATCGCGGCATCTTCTCTCTTCTATTGCTGTTGGTTC<br>CTTCTCTTTTTGGGGCTTCTTCACAGGTTACCCTTCACAGTGCGGGTGCGGAGTGCTATTCAAGTGAAGCCTGGACTACACCTGCGTTGCGTCCTATCCTAGTCCTCTCGTGTCCCTCGATGAGACGGTTTGCGTATATGGGGAAT<br>CATCTTGACACTGATGCACTTTGGATCGCATTTGGTTATGGTTCTTCCACCCCCCC--<br>GGTAAATGGTGCTATTTAGTGAATGCTTGTCGGACATATTTTTATCAATTTTCACTTCCTCTATTTTCTTCACAAAACTAGGAAATTCACCACAATTTTTTCTTTGTTATTTTTTAATTTTTTTTTTATTTTTTAAAAACATTTTTTAAA<br>AAACTAAATTACATACAAACTACCGCATAAAATCCCTCAAACTATACAAACGTTTATCGTATAATATATATACATTATTGTTTATTCTATCATTATTAGAGAAACTCCACTACCAAAACCATCATTAAAACAAAAATTTACATGCCAC<br>TTAACTCCCCTCACAAACAATCGTTATTTATATTGTTAATTAGCAAACACAAAACCCAC
CamHap_44	ATTTTATTTTTTAACCTAACTCCCCTACTAAGTGTACCCCCCCTTTCCCCCCCAGGGGGGGTATACTATGCATAATCGTGCATACATTTATATACCACATATATTATGGTACCGGTAATATATACTATATATGTACTAAACCCATTAT<br>ATGTATACGGGCATTAATCTATATTCCACATTTCTCCCAATGTCCATTCTATGCATGATCCAGGACACACTCATTCACCCTCCCCATAGACAGCTCCAAACCACTACCAAGTCACCTAACTATGAATGGTTACAGGACATAAATCT<br>CACTCTCATGTTCTTCCCCCAACAAGTCACCTAACTATGAATGGTTACAGGACATACATTTAACTACC---<br>ATGTTCTAACCCATTTGGTTATGCTCGCCGTATCAGATGGATTTATTGATCGTCCACCTCACGAGAGATCAGCAACCCCTGCTTGTAATGTACTTCATGACCAGTCTCAGGCCCATTCTTTCCCCCTACACCCCTCGCCCTACTTG<br>CCTTCCACCGTACCTCTGGTTCCTCGGTCAGGCACATCCCATGCATAACTCCTGAACTTTCTCACTTTTCACGAAGTCATCTGTGGATTATCTTCCCCTCTTTAGTCCGTGATCGCGGCATCTTCTCTCTTCTATTGCTGTTGGTTC<br>CTTCTCTTTTTGGGGCTTCTTCACAGGTTGCCCTTCACAGTGCGGGTGCGGAGTGCTATTCAAGTGAAGCCTGGACTACACCTGCGTTGCGTCCTATCCTAGTCCTCTCGTGTCCCTCGATGAGACGGTTTGCGTGTATGGGGAAT<br>CATCTTGACACTGATGCACTTTGGATCGCATTTGGTTATGGTTCTTCCACCCCCCCC-<br>GGTAAATGGTGCTATTTAGTGAATGCTTGTCGGACATATTTTTATCAATTTTCACTTCCTCTATTTTCTTCACAAAACTAGGAAATTCACCACAATTTTTTCTTTGTTATTTTTTAATTTTTTTTTTATTTTTTAAAAACATTTTTTAAA<br>AAACTAAATTACATACAAACTACCGCATAAAATCCCTCAAACTATACAAACGTTTATCGTATAATATATATACATTATTGTTTATTCTATCATTATTAGAGAAACTCCACTACCAAAACCATCATTAAAACAAAAATTTACATGCCAC<br>TTAACTCCCCTCACAAACAATCGTTATTTATATTGTTAATTAGCAAACACAAAACCTGC

CamHap_45	ATTTTATTTTTTAACCTAACTCCCCTACTAAGTGTACCCCCCCTTTCCCCCCCAGGGGGGGTATACTATGCATAATCGTGCATACATTTATATACCACATATATTATGGTACCGGTAATATATACTATATATGTACTAAACCCATTAT<br>ATGTATACGGGCATTAATCTATATTCCACATTTCTCCCAATGTCCATTCTATGCATGATCCAAGACATACTCATTCACCCTCCCCATAGACAGCTCCAAACCACTACCAAGTCACCTAACTATGAATGGTTGCAGGACATAAATCTC<br>ACTCTCATGCTCTTCCCCCAACAAGTCACCTAACTATGAATGGTTACAGGACATACATTTAACTACC---<br>ATGTTCTAACCCATTTGGTTATGCTCGCCGTATCAGATGGATTTATTGATCGTCCACCTCACGAGAGATCAGCAACCCCTGCCTGTAATGTACTTCATGACCAGTCTCAGGCCCATTCTTTCCCCCTACACCCCTCGCCCTACTTG<br>CCTTCCACCGTACCTCTGGTTCCTCGGTCAGGCACATCCCATGCATAACTCCTGAACTTTCTCACTTTTCACGAAGTCATCTGTGGATTATCTTCCCCTCTTTAGTCCGTGATCGCGGCATCTTCTCTCTTCTATTGCTGTTGGTTC<br>CTTCTCTTTTTGGGGCTTCTTCACAGGTTGCCCTTCACAGTGCGGGTGCGGAGTGCTATTCAAGTGAAGCCTGGACTACACCTGCGTTGCGTCCTATCCTAGTCCTCTCGTGTCCCTCGATGAGACGGTTTGCGTGTATGGGGAAT<br>CATCTTGACACTGATGCACTTTGGATCGCATTTGGTTATGGTTCTTCCACCCCCCC--<br>GGTAAATGGTGCTATTTAGTGAATGCTTGTCGGACATATTTTTATCAATTTTCACTTCCTCTATTTTCTTCACAAAACTAGGAAATTCACCACAATTTTTTCTTTGTTATTTTTTAATTTTTTTTTTATTTTTTAAAAACATTTTTTAAA<br>AAACTAAATTACATACAAACTACCGCATAAAATCCCTCAAACTATACAAACGTTTATCGTATAATATATATACATTATTGTTTATTCTATCATTATTAGAGAAACTCCACTACCAAAACCATCATTAAAACAAAAATTTACATGCCAC<br>TTAACTCCCCTCACAAACAATCGTTATTTATATTGTTAATTAGCAAACACAAAACCCGC
CamHap_46	ATTTTATTTTTTAACCTAACTCCCCTACTAAGTGTACCCCCCCTTTCCCCCCCAGGGGGGGTATACTATGCATAATCGTGCATACATTTATATACCACATATATTATGGTACCGGTAATATATACTATATATGTACTAAACCCATTAT<br>ATGTATACGGGCATTAACCTATATTCCACATTTCTCCCAATGTCCATTCTATGCATGATCCAGGACATACTCATTTACCCTCCCCATAGACAGTTCCAAACCACTATCAAGCCACCTAACTATGAATGGTTACAGGACATAAATCTC<br>ACTCTCATGTTCTCCCCCCAACAAGTCACCTAACTATGAATGGTTACAGGACATACATTTAACTACC---<br>ATGTTCTAACCCATTTGGTTATGCTCGCCGTATCAGATGGATTTATTGATCGTCCACCTCACGAGAGATCAGCAACCCCTGCCTGTAATGTACTTCATGACCAGTCTCAGGCCCATTCTTTCCCCCTACACCCCTCGCCCTACTTG<br>CCTTCCACCGTACCTCTGGTTCCTCGGTCAGGCACATCCCATGCATAACTCCTGAACTTTCTCACTTTTCACGAAGTCATCTGTGGATTATCTTCCCCTCTTTAGTCCGTGATCGCGGCATCTTCTCTCTTCTATTGCTGTTGGTTC<br>CTTCTCTTTTTGGGGCTTCTTCACAGGTTGCCCTTCACAGTGCGGGTGCGGAGTGCTATTCAAGTGAAGCCTGGACTACACCTGCGTTGCGTCCTATCCTAGTCCTCTCGTGTCCCTCGATGAGACGGTTTGCGTATATGGGGAAT<br>CATCTTGACACTGATGCACTTTGGATCGCATTTGGTTATGGTTCTTCCACCCCCCC--<br>GGTAAATGGTGCTATTTAGTGAATGCTTGTCGGACATATTTTTATCAATTTTCACTTCCTCTATTTTCTTCACAAAACTAGGAAATTCACCACAATTTTTTCTTTGTTATTTTTTAATTTTTTTTTTATTTTTTAAAAACATTTTTTAAA<br>AAACTAAATTACATACAAACTACCGCATAAAATCCCTCAAACTATACAAACGTTTATCGTATAATATATATACATTATTGTTTATTCTATCATTATTAGAGAAACTCCACTACCAAAACCATCATTAAAACAAAAATTTACATGCCAC<br>TTAACTCCCCTCACAAACAATCGTTATTTATATTGTTAATTAGCAAACACAAAACCCGC
CamHap_47	ATTTTATTTTTTAACCTAACTCCCCTACTAAGTGTACCCCCCCTTTCCCCCCCAGGGGGGGTATACTATGCATAATCGTGCATACATTTATATACCACATATATTATGGTACCGGTAATATATACTATATATGTACTAAACCCATTAT<br>ATGTATACGGGCATTAACCTATATTCCACATTTCTCCCAATGTCCATTCTATGCATGATCCAGGACATACTCATTTACCCTCCCCATAGACAGTTCTAAACCACTATCAAGCCACCTAACTATGAATGGTTACAGGACATAAATCTC<br>ACTCTCATGTTCTCCCCCCAACAAGTCACCTAACTATGAATGGTTACAGGACATACACTTAACTACC---<br>ATGTTCTAACCCATTTGGTTATGCTCGCCGTATCAGATGGATTTATTGATCGTCCACCTCACGAGAGATCAGCAACCCCTGCCTGTAATGTACTTCATGACCAGTCTCAGGCCCATTCTTTCCCCCTACACCCCTCGCCCTACTTG<br>CCTTCCACCGTACCTCTGGTTCCTCGGTCAGGCACATCCCATGCATAACTCCTGAACTTTCTCACTTTTCACGAAGTCATCTGTGGATTATCTTCCCCTCTTTAGTCCGTGATCGCGGCATCTTCTCTCTTCTATTGCTGTTGGTTC<br>CTTCTCTTTTTGGGGCTTCTTCACAGGTTGCCCTTCACAGTGCGGGTGCGGAGTGCTATTCAAGTGAAGCCTGGACTACACCTGCGTTGCGTCCTATCCTAGTCCTCTCGTGTCCCTCGATGAGACGGTTTGCGTGTATGGGGAAT<br>CATCTTGACACTGATGCACTTTGGATCGCATTTGGTTATGGTTCTTCCACCCCCCC--<br>GGTAAATGGTGCTATTTAGTGAATGCTTGTCGGACATATTTTTATCAATTTTCACTTCCTCTATTTTCTTCACAAAACTAGGAAATTCACCACAATTTTTTCTTTGTTATTTTTTAATTTTTTTTTTATTTTTTAAAAACATTTTTTAAA<br>AAACTAAATTACATACAAACTACCGCATAAAATCCCTCAAACTATACAAACGTTTATCGTATAATATATATACATTATTGTTTATTCTATCATTATTAGAGAAACTCCACTACCAAAACCATCATTAAAACAAAAATTTACATGCCAC<br>TTAACTCCCCTCACAAACAATCGTTATTTATATTGTTAATTAGCAAACACAAAACCCGC
CamHap_48	ATTTTATTTTTTAACCTAACTCCCCTACTAAGTGTACCCCCCCTTTCCCCCCCAGGGGGGGTATACTATGCATAATCGTGCATACATTTATATACCACATATATTATGGTACCGGTAATATATACTATATATGTACTAAACCCATTAT<br>ATGTATACGGGCATTAATCTATATTCCACATTTCTCCCAATGTCCATTCTATGCATGATCCAAGACATACTCATTCACCCTCCCCATAGACAGTTCTAAACCACTATCAAGCCACCTAACTATGAATGGTTACAGGACATAAATCTT<br>ACTCTCATGTTCTCCCCCTAACAAGTCACCTAACTATGAATGGTTACAGGACATACATTTAACTACC---<br>ATGTTCTAACCCATTTGGTTATGCTCGCCGTATCAGATGGATTTATTGATCGTCCACCTCACGAGAGATCAGCAACCCCTGCCTGTAATGTACTTCATGACCAGTCTCAGGCCCATTCTTTCCCCCTACACCCCTCGCCCTACTTG<br>CCTTCCACCGTACCTCTGGTTCCTCGGTCAGGCACATCCCATGCATAACTCCTGAACTTTCTCACTTTTCACGAAGTCATCTGTGGATTATCTTCCCCTCTTTAGTCCGTGATCGCGGCATCTTCTCTCTTCTATTGCTGTTGGTTC<br>CTTCTCTTTTTGGGGCTTCTTCACAGGTTGCCCTTCACAGTGCGGGTGCGGAGTGCTATTCAAGTGAAGCCTGGACTACACCTGCGTTGCGTCCTATCCTAGTCCTCTCGTGTCCCTCGATGAGACGGTTTGCGTGTATGGGGAAT<br>CATCTTGACACTGATGCACTTTGGATCGCATTTGGTTATGGTTCTTCCACCCCCCC--<br>GGTAAATGGTGCTATTTAGTGAATGCTTGTCGGACATATTTTTATCAATTTTCACTTCCTCTATTTTCTTCACAAAACTAGGAAATTCACCACAATTTTTTCTTTGTTATTTTTTAATTTTTTTTTTATTTTTTAAAAACATTTTTTAAA<br>AAACTAAATTACATACAAACTACCGCATAAAATCCCTCAAACTATACAAACGTTTATCGTATAATATATATACATTATTGTTTATTCTATCATTATTAGAGAAACTCCACTACCAAAACCATCATTAAAACAAAAATTTACATGCCAC<br>TTAACTCCCCTCACAAACAATCGTTATTTATATTGTTAATTAGCAAACACAAAACCCAC

CamHap_49	ATTTTATTTTTTAACCTAACTCCCCTACTAAGTGTACCCCCCCTTTCCCCCCCAGGGGGGGTATACTATGCATAATCGTGCATACATTTATATACCACATATATTATGGTACCGGTAATATATACTATATATGTACTAAACCCATTAT<br>ATGTATACGGGCATTAATCTATATTCCACATTTCTCCCAATGTCCATTCTATGCATGATCCAGGACATACTCATTCACCCTCCCCATAGACAACTCCAAACCACTACCAAGCCACCTAACTATGAATGGTTACAGGACATAAATCTC<br>ACTCTCATGTTCTTCCCCCAACAAGTCACCTAACTATGAATGGTTGCAGGACATACATTTAACTACT--<br>ATGCTCTAACCCATTTGGTTATGCTCGTCGTATCAGATGGATTTATTGATCGTCCACCTCACGAGAGATCAGCAACCCCTGCCTGTAATGTACTTCATGACCAGTCTCAGGCCCATTCTTTCCCCCTACACCCCTCGCCCTACTTG<br>CCTTCCACCGTACCTCTGGTTCCTCGGTCAGGCACATCCCATGCATAACTCCTGAACTTTCTCACTTTTCACGAAGTCATCTGTGGATTATCTTCCCCTCTTTAGTCCGTGATCGCGGCATCTTCTCTCTTCTATTGCTGTTGGTTC<br>CTTCTCTTTTTGGGGCTTCTTCACAGGTTGCCCTTCACAGTGCGGGTGCGGAGTGCTATTCAAGTGAAGCCTGGACTACACCTGCGTTGCGTCCTATCCTAGTCCTCTCGTGTCCCTCGATGAGACGGTTTGCGTGTATGGGGAAT<br>CATCTTGACACTGATGCACTTTGGATCGCATTTGGTTATGGTTCTTCCACCCCCCCC-<br>GGTAAATGGTGCTATTTAGTGAATGCTTGTCGGACATATTTTTATCAATTTTCACTTCCTCTATTTTCTTCACAAAACTAGGAAATTCACCACAATTTTTTCTTTGTTATTTTTTAATTTTTTTTTTATTTTTTAAAAACATTTTTTAAA<br>AAACTAAATTACATACAAACTACCGCATAAAATCCCTCAAACTATACAAACGTTTATCGTATAATATATATACATTATTGTTTATTCTATCATTATTAGAGAAACTCCACTACCAAAACCATCATTAAAACAAAAATTTACATGCCAC<br>TTAACTCCCCTCACAAACAATCGTTATTTATATTGTTAATTAGCAAACACAAAACCCGC
CamHap_50	ATTTTATTTTTTAACCTAACTCCCCTACTAAGTGTACCCCCCCTTTCCCCCCCAGGGGGGGTATACTATGCATAATCGTGCATACATTTATATACCACATATATTATGGTACCGGTAATATATACTATATATGTACTAAACCCATTAT<br>ATGTATACGGGCATTAATCTATATTCCACATTTCTCCCAATGTCCATTCTATGCATGATCCAGGACATACTCATTCACCCTCCCCATAGACAGCTCCAAACCACTACCAAGTCACCTAACTATGAATGGTTGCAGGACATAAATCTC<br>ACTCTCATGCTCTTCCCCCAACAAGTCACCTAACTATGAATGGTTACAGGACATACATTTAACTACC--<br>ATGTTCTAACCCATTTGGTTATGCTCGCCGTATCAGATGGATTTATTGATCGTCCACCTCACGAGAGATCAGCAACCCCTGCCTGTAATGTACTTCATGACCAGTCTCAGGCCCATTCTTTCCCCCTACACCCCTCGCCCTACTTG<br>CCTTCCACCGTACCTCTGGTTCCTCGGTCAGGCACATCCCATGCATAACTCCTGAACTTTCTCACTTTTCACGAAGTCATCTGTGGATTATCTTCCCCTCTTTAGTCCGTGATCGCGGCATCTTCTCTCTTCTATTGCTGTTGGTTC<br>CTTCTCTTTTTGGGGCTTCTTCACAGGTTGCCCTTCACAGTGCGGGTGCGGAGTGCTATTCAAGTGAAGCCTGGACTACACCTGCGTTGCGTCCTATCCTAGTCCTCTCGTGTCCCTCGATGAGACGGTTTGCGTGTATGGGGAAT<br>CATCTTGACACTGATGCACTTTGGATCGCATTTGGTTATGGTTCTTCCACCCCCCC--<br>GGTAAATGGTGCTATTTAGTGAATGCTTGTCGGACATATTTTTATCAATTTTCACTTCCTCTATTTTCTTCACAAAACTAGGAAATTCACCACAATTTTTTCTTTGTTATTTTTTAATTTTTTTTTTATTTTTTAAAAACATTTTTTAAA<br>AAACTAAATTACATACAAACTACCGCATAAAATCCCTCAAACTATACAAACGTTTATCGTATAATATATATACATTATTGTTTATTCTATCATTATTAGAGAAACTCCACTACCAAAACCATCATTAAAACAAAAATTTACATGCCAC<br>TTAACTCCCCTCACAAACAATCGTTATTTATATTGTTAATTAACAAACACAAAACCCGC
CamHap_51	ATTTTATTTTTTAACCTAACTCCCCTACTAAGTGTACCCCCCCTTTCCCCCCCAGGGGGGGTATACTATGCATAATCGTGCATACATTTATATACCACATATATTATGGTACCGGTAATATATACTATATATGTACTAAACCCATTAT<br>ATGTATACGGGCATTAACCTATATTCCACATTTCTCCCAATGTCCATTCTATGCATGATCCAGGACATACTCATTTACCCTCCCCATAGACAGTTCCAAACCACTATCAAGCCACCTAACTATGAATGGTTACAGGACATAAATCTC<br>ACTCTCATGTTCTCCCCCCAACAAGTCACCTAACTATGAATGGTTACAGGACATACATTTAACTACC--<br>ATGTTCTAACCCATTTGGTTATGCTCGCCGTATCAGATGGATTTATTGATCGTCCACCTCACGAGAGATCAGCAACCCCTGCCCGTAATGTACTTCATGACCAGTCTCAGGCCCATTCTTTCCCCCTACACCCCTCGCCCTACTTG<br>CCTTCCACCGTACCTCTGGTTCCTCGGTCAGGCACATCCCATGCATAACTCCTGAACTTTCTCACTTTTCACGAAGTCATCTGTGGATTATCTTCCCCTCTTTAGTCCGTGATCGCGGCATCTTCTCTCTTCTATTGCTGTTGGTTC<br>CTTCTCTTTTTGGGGCTTCTTCACAGGTTGCCCTTCACAGTGCGGGTGCGGAGTGCTATTCAAGTGAAGCCTGGACTACACCTGCGTTGCGTCCTATCCTAGTCCTCTCGTGTCCCTCGATGAGACGGTTTGCGTGTATGGGGAAT<br>CATCTTGACACTGATGCACTTTGGATCGCATTTGGTTATGGTTCTTCCACCCCCCC--<br>GGTAAATGGTGCTATTTAGTGAATGCTTGTCGGACATATTTTTATCAATTTTCACTTCCTCTATTTTCTTCACAAAACTAGGAAATTCACCACAATTTTTTCTTTGTTATTTTTTAATTTTTTTTTTATTTTTTAAAAACATTTTTTAAA<br>AAACTAAATTACATACAAACTACCGCATAAAATCCCTCAAACTATACAAACGTTTATCGTATAATATATATACATTATTGTTTATTCTATCATTATTAGAGAAACTCCACTACCAAAACCATCATTAAAACAAAAATTTACATGCCAC<br>TTAACTCCCCTCACAAACAATCGTTATTTATATTGTTAATTAGCAAACACAAAACCCGC
CamHap_52	ATTTTATTTTTTAACCTAACTCCCCTACTAAGTGTACCCCCCCTTTCCCCCCCAGGGGGGGTATACTATGCATAATCGTGCATACATTTATATACCACATATATTATGGTACCGGTAATATATACTATATATGTACTAAACCCATTAT<br>ATGTATACGGGCATTAATCTATATTCCACATTTCTCCCAATGTCCATTCTATGCATGATCCAGGACATACTCATTCACCCTCCCTACAGACAGCTCCAAACCACCACCAAGTCACCTAACTATGAATGGTTACAGGACATAAATCT<br>CACTCTCATGTTCTTCCCCTACCAAGTCACCTAACTATGAATGGTTACAGGACATACATTTAACTACC--<br>ATGTTCTAACCCATTTGGTTATGCTCGCCGTATCAGATGGATTTATTGATCGTCCACCTCACGAGAGATCAGCAACCCCTGCTTGTAATGTACTTCATGACCAGTCTCAGGCCCATTCTTTCCCCCTACACCCCTCGCCCTACTTG<br>CCTTCCACCGTACCTCTGGTTCCTCGGTCAGGCACATCCCATGCATAACTCCTGAACTTTCTCACTTTTCACGAAGTCATCTGTGGATTATCTTCCCCTCTTTAGTCCGTGATCGCGGCATCTTCTCTCTTCTATTGCTGTTGGTTC<br>CTTCTCTTTTTGGGGCTTCTTCACAGGTTGCCCTTCACAGTGCGGGTGCGGAGTGCTATTCAAGTGAAGCCTGGACTACACCTGCGTTGCGTCCTATCCTAGTCCTCTCGTGTCCCTCGATGAGACGGTTTGCGTGTATGGGGAAT<br>CATCTTGACACTGATGCACTTTGGATCGCATTTGGTTATGGTTCTTCCACCCCCCC--<br>GGTAAATGGTGCTATTTAGTGAATGCTTGTCGGACATATTTTTACCAATTTTCACTTCCTCTATTTTCTTCACAAAACTAGGAAATTCACCACAATTTTTTCTTTGTTATTTTTTAATTTTTTTTTTATTTTTTAAAAACATTTTTTAAA<br>AAACTAAATTACATACAAACTACCGCATAAAATCCCTCAAACTATACAAACGTTTATCGTATAATATATATACATTATTGTTTATTCTATCATTATTAGAGAAACTCCACTACCAAAACCATCATTAAAACAAAAATTTACATGCCAC<br>TTAACTCCCCTCACAAACAATCGTTATTTATATTGTTAATTAGCAAACACAAAACCTGC

CamHap_53	ATTTTATTTTTTAACCTAACTCCCCTACTAAGTGTACCCCCCCTTTCCCCCCCAGGGGGGGTATACTATGCATAATCGTGCATACATTTATATACCACATATATTATGGTACCGGTAATATATACTATATATGTACTAAACCCATTAT<br>ATGTATACGGGCATTAACCTATATTCCACATTTCTCCCAATGTCCATTCTATGCATGATCCAGGACATACTCATTTACCCTCCCCATAGACAGTTCCAAACCACTATCAAGCCACCTAACTATGAATGGTTACAGGACATAAATCTC<br>ACTCTCATGTTCTCCCCCCAACAAGTCACCTAACTATGAATGGTTACAGGACATACATTTAACTACC--<br>ATGTTCTAACCCATTTGGTTATGCTCGCCGTATCAGATGGATTTATTGATCGTCCACCTCACGAGAGATCAGCAACCCCTGCCTGTAATGTACTTCATGACCAGTCTCAGGCCCATTCTTTCCCCCTACACCCCTCGCCCTACTTG<br>CCTTCCACCGTACCTCTGGTTCCTCGGTCAGGCACATCCCATGCATAACTCCTGAACTTTCTCACTTTTCACGAAGTCATCTGTGGATTATCTTCCCCTCTTTAGTCCGTGATCGCGGCATCTTCTCTCTTCTATTGCTGTTGGTTC<br>CTTCTCTTTTTGGGGCTTCTTCACAGGTTGCCCTTCACAGTGCGGGTGCGGAGTGCTATTCAAGTGAAGCCTGGACTACACCTGCGTTGCGTCCTATCCTAGTCCTCTCGTGTCCCTCGATGAGACGGTTTGCGTGTATGGGGAAT<br>CATCTTGACACTGATGCACTTTGGATCGCATTTGGTTATGGTTCTTCCACCCCCCCC-<br>GGTAAATGGTGCTATTTAGTGAATGCTTGTCGGACATATTTTTATCAATTTTCACTTCCTCTATTTTCTTCACAAAACTAGGAAATTCACCACAATTTTTTCTTTGTTATTTTTTAATTTTTTTTTTATTTTTTAAAAACATTTTTTAAA<br>AAACTAAATTACATACAAACTACCGCATAAAATCCCTCAAACTATACAAACGTTTATCGTATAATATATATACATTATTGTTTATTCTATCATTATTAGAGAAACTCCACTACCAAAACCATCATTAAAACAAAAATTTACATGCCAC<br>TTAACTCCCCTCACAAACAATCGTTATTTATATTGTTAATTAGCAAACACAAAACCCGC
CamHap_54	ATTTTATTTTTTAACCTAACTCCCCTACTAAGTGTACCCCCCCTTTCCCCCCCAGGGGGGGTATACTATGCATAATCGTGCATACATTTATATACCACATATATTATGGTACCGGTAATATATACTATATATGTACTAAACCCATTAT<br>ATGTATACGGGCATTAATCTACATTCCACATTTCTCCCAATGTCCATTCTATGCATGATCCAAGACATACTCATTCACCCTCCCCATAGACAGCTCCAAACCACTACCAAGTCACCTAACTATGAATGGTTACAGGACATAAATCTT<br>ACTCTCATGTTCTTCCCCCAACAAGACACCTAACTATGAATGGTTACAGGACATATACTTAACTACT--<br>ATGTTCTAACCCATTTGGTTATGCTCGTCGTATCAGATGGATTTATTGATCGTTCACCTCACGAGAGATCAGCAACCCCTGCTTGTAATGTACTTCATGACCAGTCTCAGGCCCATTCTTTCCCCCTACACCCCTCGCCCTACTTG<br>CCTTCCACCGTACCTCTGGTTCCTCGGTCAGGCACATCCCATGCATAACTCCTGAACTTTCTCACTTTTCACGAAGTCATCTGTGGATTATCTTCCCCTCTTTAGTCCGTGATCGCGGCATCTTCTCTCTTCTATTGCTGTTGGTTC<br>CTTCTCTTTTTGGGGCTTCTTCACAGGTTACCCTTCACAGTGCGGGTGCGGAGTGCTATTCAAGTGAAGCCTGGACTACACCTGCGTTGCGTCCTATCCTAGTCCTCTCGTGTCCCTCGATGAGACGGTTTGCGTGTATGGGGAAT<br>CATCTTGACACTGATGCACTTTGGATCGCATTTGGTTATGGTTCTTCCACCCCCCCC-<br>GGTAAATGGTGCTATTTAGTGAATGCTTGTCGGACATATTTTTATCAATTTTCACTTCCTCTATTTTCTTCACAAAACTAGGAAATTCACCACAATTTTTTCTTTGTTATTTTTTAATTTTTTTTTTATTTTTTAAAAACATTTTTTAAA<br>AAACTAAATTACATACAAACTACCGCATAAAATCCCTCAAACTATACAAACGTTTATCGTATAATATATATACATTATTGTTTATTCTATCATTATTAGAGAAACTCCACTACCAAAACCATCATTAAAACAAAAATTTACATGCCAC<br>TTAACTCCCCTCACAAACAATCGTTATTTATATTGTTAATTAGCAAACACAAAACCCGC
CamHap_55	ATTTTATTTTTTAACCTAACTCCCCTACTAAGTGTACCCCCCCTTTCCCCCCCAGGGGGGGTATACTATGCATAATCGTGCATACATTTATATACCACATATATTATGGTACCGGTAATATATACTATATATGTACTAAACCCATTAT<br>ATGTATACGGGCATTAATCTATATTCCACATTTCTCCCAATGTCCATTCTATGCATGATCCAGGACATACTCATTCACCCTCCCTACAGACAGCTCCAAACCACCACCAAGTCACCTAACTATGAATGGTTACGGGACATAAATCT<br>CACTCTCATGTTCTTCCCCTACCAAGTCACCTAACTATGAATGGTTACAGGACATACATTTAACTACC--<br>ATGTTCTAACCCATTTGGTTATGCTCGCCGTATCAGATGGATTTATTGATCGTCCACCTCACGAGAGATCAGCAACCCCTGCTTGTAATGTACTTCATGACCAGTCTCAGGCCCATTCTTTCCCCCTACACCCCTCGCCCTACTTG<br>CCTTCCACCGTACCTCTGGTTCCTCGGTCAGGCACATCCCATGCATAACTCCTGAACTTTCTCACTTTTCACGAAGTCATCTGTGGATTATCTTCCCCTCTTTAGTCCGTGATCGCGGCATCTTCTCTCTTCTATTGCTGTTGGTTC<br>CTTCTCTTTTTGGGGCTTCTTCACAGGTTGCCCTTCACAGTGCGGGTGCGGAGTGCTATTCAAGTGAAGCCTGGACTACACCTGCGTTGCGTCCTATCCTAGTCCTCTCGTGTCCCTCGATGAGACGGTTTGCGTGTATGGGGAAT<br>CATCTTGACACTGATGCACTTTGGATCGCATTTGGTTATGGTTCTTCCACCCCCCC--<br>GGTAAATGGTGCTATTTAGTGAATGCTTGTCGGACATATTTTTACCAATTTTCACTTCCTCTATTTTCTTCACAAAACTAGGAAATTCACCACAATTTTTTCTTTGTTATTTTTTAATTTTTTTTTTATTTTTTAAAAACATTTTTTAAA<br>AAACTAAATTACATACAAACTACCGCATAAAATCCCTCAAACTATACAAACGTTTATCGTATAATATATATACATTATTGTTTATTCTATCATTATTAGAGAAACTCCACTACCAAAACCATCATTAAAACAAAAATTTACATGCCAC<br>TTAACTCCCCTCACAAACAATCGTTATTTATATTGTTAATTAGCAAACACAAAACCTGC
CamHap_56	ATTTTATTTTTTAACCTAACTCCCCTACTAAGTGTACCCCCCCTTTCCCCCCCAGGGGGGGTATACTATGCATAATCGTGCATACATTTATATACCACATATATTATGGTACCGGTAATATATACTATATATGTACTAAACCCATTAT<br>ATGTATACGGGCATTAATCTATATCCCACATTTCTCCCAATGTCCATTCTATGCATGATCCAAGACATACTCATTCACCCTCCCCATAGACAGTTCTAAACCACTATCAAGCCACCTAACTATGAATGGTTACAGGACATAAATCTC<br>ACTCTCATGTTCTCCCCCTAACAAGTCACCTAACTATGAATGGTTACAGGACATACATTTAACTACC--<br>ATGTTCTAACCCATTTGGTTATGCTCGCCGTATCAGATGGATTTATTGATCGTCCACCTCACGAGAGATCAGCAACCCCTGCCTGTAATGTACTTCATGACCAGTCTCAGGCCCATTCTTTCCCCCTACACCCCTCGCCCTACTTG<br>CCTTCCACCGTACCTCTGGTTCCTCGGTCAGGCACATCCCATGCATAACTCCTGAACTTTCTCACTTTTCACGAAGTCATCTGTGGATTATCTTCCCCTCTTTAGTCCGTGATCGCGGCATCTTCTCTCTTCTATTGCTGTTGGTTC<br>CTTCTCTTTTTGGGGCTTCTTCACAGGTTGCCCTTCACAGTGCGGGTGCGGAGTGCTATTCAAGTGAAGCCTGGACTACACCTGCGTTGCGTCCTATCCTAGTCCTCTCGTGTCCCTCGATGAGACGGTTTGCGTGTATGGGGAA<br>TCATCTTGACACTGATGCACTTTGGATCGCATTTGGTTATGGTTCTTCCACCCCCCCC-<br>GGTAAATGGTGCTATTTAGTGAATGCTTGTCGGACATATTTTTATCAATTTTCACTTCCTCTATTTTCTTCACAAAACTAGGAAATTCACCACAATTTTTTCTTTGTTATTTTTTAATTTTTTTTTTATTTTTTAAAAACATTTTTTAAA<br>AAACTAAATTACATACAAACTACCGCATAAAATCCCTCAAACTATACAAACGTTTATCGTATAATATATATACATTATTGTTTATTCTATCATTATTAGAGAAACTCCACTACCAAAACCATCATTAAAACAAAAATTTACATGCCAC<br>TTAACTCCCCTCACAAACAATCGTTATTTATATTGTTAATTAGCAAACACAAAACCCAC

CamHap_57	ATTTTATTTTTTAACCTAACTCCCCTACTAAGTGTACCCCCCCTTTCCCCCCCAGGGGGGGTATACTATGCATAATCGTGCATACATTTATATACCACATATATTATGGTACCGGTAATATATACTATATATGTACTAAACCCATTAT<br>ATGTATACGGGCATTAATCTATATTCCACATTTCTCCCAATGTCCATTCTATGCATGATCCAAGACATACTCATTCACCCTCCCCATAGACAGTTCTAAACCACTATCAAGCCACCTAACTATGAATGGTTACAGGACATAAATCTC<br>ACTCTCATGTTCTCCCCCTAACAAGTCACCTAACTATGAATGGTTACAGGACATACATTTAACTACC-----------------------------------<br>ATGTTCTAACCCATTTGGTTATGCTCGCCGTATCAGATGGATTTATTGATCGTCCACCTCACGAGAGATCAGCAACCCCTGCCTGTAATGTACTTCATGACCAGTCTCAGGCCCATTCTTTCCCCCTACACCCCTCGCCCTACTTG<br>CCTTCCACCGTACCTCTGGTTCCTCGGTCAGGCACATCCCATGCATAACTCCTGAACTTTCTCACTTTTCACGAAGTCATCTGTGGATTATCTTCCCCTCTTTAGTCCGTGATCGCGGCATCTTCTCTCTTCTATTGCTGTTGGTTC<br>CTTCTCTTTTTGGGGCTTCTTCACAGGTTGCCCTTCACAGTGCGGGTGCGGAGTGCTATTCAAGTGAAGCCTGGACTACACCTGCGTTGCGTCCTATCCTAGTCCTCTCGTGTCCCTCGATGAGACGGTTTGCGTGTATGGGGAA<br>TCATCTTGACACTGATGCACTTTGGATCGCATTTGGTTATGGTTCTTCCACCCCCCCC-<br>GGTAAATGGTGCTATTTAGTGAATGCTTGTCGGACATATTTTTATCAATTTTCACTTCCTCTATTTTCTTCACAAAACTAGGAAATTCACCACAATTTTTTCTTTGTTATTTTTTAATTTTTTTTTTATTTTTTAAAAACATTTTTTAAA<br>AAACTAAATTACATACAAACTACCGCATAAAATCCCTCAAACTATACAAACGTTTATCGTATAATATATATACATTATTGTTTATTCTATCATTATTAGAGAAACTCCACTACCAAAACCATCATTAAAACAAAAATTTACATGCCAC<br>TTAACTCCCCTCACAAACAATCGTTATTTATATTGTTAATTAGCAAACACAAAACCCAC
CamHap_58	ATTTTATTTTTTAACCTAACTCCCCTACTAAGTGTACCCCCCCTTTCCCCCCCAGGGGGGGTATACTATGCATAATCGTGCATACATTTATATACCACATATATTATGGTACCGGTAATATATACTATATATGTACTAAACCCATTAT<br>ATGTATACGGGCATTAATCTATATTCCACATTTCTCCCAATGTCCATTCTATGCATGATCCAGGACATACCCATTCACCCTCCCCATAGACAGCTCCAAACCACTACCAAGTCACCTAACTATGAATGGTTGCAGGACATAAATCT<br>CACTCTCATGCTCTTCCCCCAACAAGTCACCTAACTATGAATGGTTACGGGACATACATTTAACTACC-----------------------------------<br>ATGTTCTAACCCATTTGGTTATGCTCGCCGTATCAGATGGATTTATTGATCGTCCACCTCACGAGAGATCAGCAACCCCTGCCTGTAATGTACTTCATGACCAGTCTCAGGCCCATTCTTTCCCCCTACACCCCTCGCCCTACTTG<br>CCTTCCACCGTACCTCTGGTTCCTCGGTCAGGCACATCCCATGCATAACTCCTGAACTTTCTCACTTTTCACGAAGTCATCTGTGGATTATCTTCCCCTCTTTAGTCCGTGATCGCGGCATCTTCTCTCTTCTATTGCTGTTGGTTC<br>CTTCTCTTTTTGGGGCTTCTTCACAGGTTGCCCTTCACAGTGCGGGTGCGGAGTGCTATTCAAGTGAAGCCTGGACTACACCTGCGTTGCGTCCTATCCTAGTCCTCTCGTGTCCCTCGATGAGACGGTTTGCGTGTATGGGGAAT<br>CATCTTGACACTGATGCACTTTGGATCGCATTTGGTTATGGTTCTTCCACCCCCCCC-<br>GGTAAATGGTGCTATTTAGTGAATGCTTGTCGGACATATTTTTATCAATTTTCACTTCCTCTATTTTCTTCACAAAACTAGGAAATTCACCACAATTTTTTCTTTGTTATTTTTTAATTTTTTTTTTATTTTTTAAAAACATTTTTTAAA<br>AAACTAAATTACATACAAACTACCGCATAAAATCCCTCAAACTATACAAACGTTTATCGTATAATATATATACATTATTGTTTATTCTATCATTATTAGAGAAACTCCACTACCAAAACCATCATTAAAACAAAAATTTACATGCCAC<br>TTAACTCCCCTCACAAACAATCGTTATTTATATTGTTAATTAGCAAACACAAAACCCGC
CamHap_59	ATTTTATTTTTTAACCTAACTCCCCTACTAAGTGTACCCCCCCTTTCCCCCCCAGGGGGGGTATACTATGCATAATCGTGCATACATTTATATACCACATATATTATGGTACCGGTAATATATACTATATATGTACTAAACCCATTAT<br>ATGTATACGGGCATTAACCTATATTCCACATTTCTCCCAATGTCCATTCTATGCATGATCTAGGACATACTCATTTACCCTCCCCATAGACAGTTCCAAACCACTATCAAGCCACCTAACTATGAATGGTTACAGGACATAAATCTC<br>ACTCTCATGTTCTCCCCCCAACAAGTCACCTAACTATGAATGGTTACAGGACATACATTTAACTACC-----------------------------------<br>ATGTTCTAACCCATTTGGTTATGCTCGCCGTATCAGATGGATTTATTGATCGTCCACCTCACGAGAGATCAGCAACCCCTGCCTGTAATGTACTTCATGACCAGTCTCAGGCCCATTCTTTCCCCCTACACCCCTCGCCCTACTTG<br>CCTTCCACCGTACCTCTGGTTCCTCGGTCAGGCACATCCCATGCATAACTCCTGAACTTTCTCACTTTTCACGAAGTCATCTGTGGATTATCTTCCCCTCTTTAGTCCGTGATCGCGGCATCTTCTCTCTTCTATTGCTGTTGGTTC<br>CTTCTCTTTTTGGGGCTTCTTCACAGGTTGCCCTTCACAGTGCGGGTGCGGAGTGCTATTCAAGTGAAGCCTGGACTACACCTGCGTTGCGTCCTATCCTAGTCCTCTCGTGTCCCTCGATGAGACGGTTTGCGTGTATGGGGAAT<br>CATCTTGACACTGATGCACTTTGGATCGCATTTGGTTATGGTTCTTCCACCCCCCC--<br>GGTAAATGGTGCTATTTAGTGAATGCTTGTCGGACATATTTTTATCAATTTTCACTTCCTCTATTTTCTTCACAAAACTAGGAAATTCACCACAATTTTTTCTTTGTTATTTTTTAATTTTTTTTTTATTTTTTAAAAACATTTTTTAAA<br>AAACTAAATTACATACAAACTACCGCATAAAATCCCTCAAACTATACAAACGTTTATCGTATAATATATATACATTATTGTTTATTCTATCATTATTAGAGAAACTCCACTACCAAAACCATCATTAAAACAAAAATTTACATGCCAC<br>TTAACTCCCCTCACAAACAATCGTTATTTATATTGTTAATTAGCAAACACAAAACCCGC
CamHap_60	ATTTTATTTTTTAACCTAACTCCCCTACTAAGTGTACCCCCCCTTTCCCCCCCAGGGGGGGTATACTATGCATAATCGTGCATACATTTATATACCACATATATTATGGTACCGGTAATATATACTATATATGTACTAAACCCATTAT<br>ATGTATACGGGCATTAACCTATATTCCACATTTCTCCCAATGTCCATTCTATGCATGATCCAGGACATACTCATTTACCCTCCCCATAGACAGTTCTAAACCACTATCAAGCCACCTAACTATGAATGGTTACAGGACATAAATCTC<br>ACTCTCATGTTCTCCCCCCAACAAGTCACCTAACTATGAATGGTTACAGGACATACATTTAACTACC-----------------------------------<br>ATGTTCTAACCCATTTGGTTATGCTCGCCGTATCAGATGGATTTATTGATCGTCCACCTCACGAGAGATCAGCAACCCCTGCCTGTAATGTACTTCATGACCAGTCTCAGGCCCATTCTTTCCCCCTACACCCCTCGCCCTACTTG<br>CCTTCCACCGTACCTCTGGTTCCTCGGTCAGGCACATCCCATGCATAACTCCTGAACTTTCTCACTTTTCACGAAGTCATCTGTGGATTATCTTCCCCTCTTTAGTCCGTGATCGCGGCATCTTCTCTCTTCTATTGCTGTTGGTTC<br>CTTCTCTTTTTGGGGCTTCTTCACAGGTTGCCCTTCACAGTGCGGGTGCGGAGTGCTATTCAAGTGAAGCCTGGACTACACCTGCGTTGCGTCCTATCCTAGTCCTCTCGTGTCCCTCGATGAGACGGTTTGCGTGTATGGGGAAT<br>CATCTTGACACTGATGCACTTTGGATCGCATTTGGTTATGGTTCTTCCACCCCCCC--<br>GGTAAATGGTGCTATTTAGTGAATGCTTGTCGGACATATTTTTATCAATTTTCACTTCCTCTATTTTCTTCACAAAACTAGGAAATTCACCACAATTTTTTCTTTGTTATTTTTTAATTTTTTTTTTATTTTTTAAAAACATTTTTTAAA<br>AAACTAAATTACATACAAACTACCGCATAAAATCCCTCAAACTATACAAACGTTTATCGTATAATATATATACATTATTGTTTATTCTATCATTATTAGAGAAACTCCACTACCAAAACCATCATTAAAACAAAAATTTACATGCCAC<br>TTAACTCCCCTCACAAACAATCGTTATTTATATTGTTAATTAGCAAACACAAAACCCGC

CamHap_61	ATTTTATTTTTTAACCTAACTCCCCTACTAAGTGTACCCCCCCTTTCCCCCCCAGGGGGGGTATACTATGCATAATCGTGCATACATTTATATACCACATATATTATGGTACCGGTAATATATACTATATATGTACTAAACCCATTAT<br>ATGTATACGGGCATTAATCTATATTCCACATTTCTCCCAATGTCCATTCTATGCATGATCCAGGACATACTCATTCACCCTCCCCATAGACAGCCCCAAACCACTACCAAGTCACCTAACTATGAATGGTTGCAGGACATAAATCT<br>CACTCTCATGCTCTTCCCCCAACAAGTCACCTAACTATGAATGGTTACAGGACATACATTTAACTACC--<br>ATGTTCTAACCCATTTGGTTATGCTCGCCGTATCAGATGGATTTATTGATCGTCCACCTCACGAGAGATCAGCAACCCCTGCCTGTAATGTACTTCATGACCAGTCTCAGGCCCATTCTTTCCCCCTACACCCCTCGCCCTACTTG<br>CCTTCCACCGTACCTCTGGTTCCTCGGTCAGGCACATCCCATGCATAACTCCTGAACTTTCTCACTTTTCACGAAGTCATCTGTGGATTATCTTCCCCTCTTTAGTCCGTGATCGCGGCATCTTCTCTCTTCTATTGCTGTTGGTTC<br>CTTCTCTTTTTGGGGCTTCTTCACAGGTTGCCCTTCACAGTGCGGGTGCGGAGTGCTATTCAAGTGAAGCCTGGACTACACCTGCGTTGCGTCCTATCCTAGTCCTCTCGTGTCCCTCGATGAGACGGTTTGCGTGTATGGGGAAT<br>CATCTTGACACTGATGCACTTTGGATCGCATTTGGTTATGGTTCTTCCACCCCCCC--<br>GGTAAATGGTGCTATTTAGTGAATGCTTGTCGGACATATTTTTATCAATTTTCACTTCCTCTATTTTCTTCACAAAACTAGGAAATTCACCACAATTTTTTCTTTGTTATTTTTTAATTTTTTTTTTATTTTTTAAAAACATTTTTTAAA<br>AAACTAAATTACATACAAACTACCGCATAAAATCCCTCAAACTATACAAACGTTTATCGTATAATATATATACATTATTGTTTATTCTATCATTATTAGAGAAACTCCACTACCAAAACCATCATTAAAACAAAAATTTACATGCCAC<br>TTAACTCCCCTCACAAACAATCGTTATTTATATTGTTAATTAGCAAACACAAAACCCGC
CamHap_62	ATTTTATTTTTTAACCTAACTCCCCTACTAAGTGTACCCCCCCTTTCCCCCCCAGGGGGGGTATACTATGCATAATCGTGCATACATTTATATACCACATATATTATGGTACCGGTAATATATACTATATACATACTAAACCCATTAT<br>ATGTATACGGGCATTAATCTATATTCCACATTTCTCCCAATGTCCATTCTATGCATGATCCAGGACATACTCATTCACCCTCCCCATAGACAGCTCCAAACCACTACCAAGTCACCTAACTATGAATGGTTGCAGGACATAAATCTC<br>ACTCTCATGCTCTTCCCCCAACAAGTCACCTAACTATGAATGGTTACAGGACATACATTTAACTACC--<br>ATGTTCTAACCCATTTGGTTATGCTCGCCGTATCAGATGGATTTATTGATCGTCCACCTCACGAGAGATCAGCAACCCCTGCCTGTAATGTACTTCATGACCAGTCTCAGGCCCATTCTTTCCCCCTACACCCCTCGCCCTACTTG<br>CCTTCCACCGTACCTCTGGTTCCTCGGTCAGGCACATCCCATGCATAACTCCTGAACTTTCTCACTTTTCACGAAGTCATCTGTGGATTATCTTCCCCTCTTTAGTCCGTGATCGCGGCATCTTCTCTCTTCTATTGCTGTTGGTTC<br>CTTCTCTTTTTGGGGCTTCTTCACAGGTTGCCCTTCACAGTGCGGGTGCGGAGTGCTATTCAAGTGAAGCCTGGACTACACCTGCGTTGCGTCCTATCCTAGTCCTCTCGTGTCCCTCGATGAGACGGTTTGCGTGTATGGGGAAT<br>CATCTTGACACTGATGCACTTTGGATCGCATTTGGTTATGGTTCTTCCACCCCCCC--<br>GGTAAATGGTGCTATTTAGTGAATGCTTGTCGGACATATTTTTATCAATTTTCACTTCCTCTATTTTCTTCACAAAACTAGGAAATTCACCACAATTTTTTCTTTGTTATTTTTTAATTTTTTTTTTATTTTTTAAAAACATTTTTTAAA<br>AAACTAAATTACATACAAACTACCGCATAAAATCCCTCAAACTATACAAACGTTTATCGTATAATATATATACATTATTGTTTATTCTATCATTATTAGAGAAACTCCACTACCAAAACCATCATTAAAACAAAAATTTACATGCCAC<br>TTAACTCCCCTCACAAACAATCGTTATTTATATTGTTAATTAGCAAACACAAAACCCGC
CamHap_63	ATTTTATTTTTTAACCTAACTCCCCTACTAAGTGTACCCCCCCTTTCCCCCCCAGGGGGGGTATACTATGCATAATCGTGCATACATTTATATACCACATATATTATGGTACCGGTAATATATACTATATATGTACTAAACCCATTAT<br>ATGTATACGGGCATTAATCTATATTCCACATTTCTCCCAATGTCCATTCTATGCATGATCCAAGACATACTCATTCACCCTCCCCATAGACAGTTCTAAACCACTATCAAGCCACCTAACTATGAATGGTTACAGGACATAAGTCTT<br>ACTCTCATGTTCTCCCCCTAACAAGTCACCTAACTATGAATGGTTACAGGACATACATTTAACTACC--<br>ATGTTCTAACCCATTTGGTTATGCTCGCCGTATCAGATGGATTTATTGATCGTCCACCTCACGAGAGATCAGCAACCCCTGCCTGTAATGTACTTCATGACCAGTCTCAGGCCCATTCTTTCCCCCTACACCCCTCGCCCTACTTG<br>CCTTCCACCGTACCTCTGGTTCCTCGGTCAGGCACATCCCATGCATAACTCCTGAACTTTCTCACTTTTCACGAAGTCATCTGTGGATTATCTTCCCCTCTTTAGTCCGTGATCGCGGCATCTTCTCTCTTCTATTGCTGTTGGTTC<br>CTTCTCTTTTTGGGGCTTCTTCACAGGTTGCCCTTCACAGTGCGGGTGCGGAGTGCTATTCAAGTGAAGCCTGGACTACACCTGCGTTGCGTCCTATCCTAGTCCTCTCGTGTCCCTCGATGAGACGGTTTGCGTATATGGGGAAT<br>CATCTTGACACTGATGCACTTTGGATCGCATTTGGTTATGGTTCTTCCACCCCCCC--<br>GGTAAATGGTGCTATTTAGTGAATGCTTGTCGGACATATTTTTATCAATTTTCACTTCCTCTATTTTCTTCACAAAACTAGGAAATTCACCACAATTTTTTCTTTGTTATTTTTTAATTTTTTTTTTATTTTTTAAAAACATTTTTTAAA<br>AAACTAAATTACATACAAACTACCGCATAAAATCCCTCAAACTATACAAACGTTTATCGTATAATATATATACATTATTGTTTATTCTATCATTATTAGAGAAACTCCACTACCAAAACCATCATTAAAACAAAAATTTACATGCCAC<br>TTAACTCCCCTCACAAACAATCGTTATTTATATTGTTAATTAGCAAACACAAAACCCAC
CamHap_64	ATTTTATTTTTTAACCTAACTCCCCTACTAAGTGTACCCCCCCTTTCCCCCCCAGGGGGGGTATACTATGCATAATCGTGCATACATTTATATACCACATATATTATGGTACCGGTAATATATACTATATATGTACTAAACCCATTAT<br>ATGTATACGGGCATTAATCTATATTCCACATTTCTCCCAATGTCCATTCTATGCATGATCCAGGACATACTCATTCACCCTCCCCATAGACAGCTCCAAACCACTACCAAGTCACCTAACTATGAATGGTTGCAGGACATAAATCTC<br>ACTCTCATGCTCTTCCCCCAACAAGTCACCTAACTATGAATGGTTGCAGGACATACATCTAACTACC--<br>ATGTTCTAACCCATTTGGTTATGCTCGCCGTATCAGATGGATTTATTGATCGTCCACCTCACGAGAGATCAGCAACCCCTGCCTGTAATGTACTTCATGACCAGTCTCAGGCCCATTCTTTCCCCCTACACCCCTCGCCCTACTTG<br>CCTTCCACCGTACCTCTGGTTCCTCGGTCAGGCACATCCCATGCATAACTCCTGAACTTTCTCACTTTTCACGAAGTCATCTGTGGATTATCTTCCCCTCTTTAGTCCGTGATCGCGGCATCTTCTCTCTTCTATTGCTGTTGGTTC<br>CTTCTCTTTTTGGGGCTTCTTCACAGGTTGCCCTTCACAGTGCGGGTGCGGAGTGCTATTCAAGTGAAGCCTGGACTACACCTGCGTTGCGTCCTATCCTAGTCCTCTCGTGTCCCTCGATGAGACGGTTTGCGTGTATGGGGAAT<br>CATCTTGACACTGATGCACTTTGGATCGCATTTGGTTATGGTTCTTCCACCCCCCC--<br>GGTAAATGGTGCTATTTAGTGAATGCTTGTCGGACATATTTTTATCAATTTTCACTTCCTCTATTTTCTTCACAAAACTAGGAAATTCACCACAATTTTTTCTTTGTTATTTTTTAATTTTTTTTTTATTTTTTAAAAACATTTTTTAAA<br>AAACTAAATTACATACAAACTACCGCATAAAATCCCTCAAACTATACAAACGTTTATCGTATAATATATATACATTATTGTTTATTCTATCATTATTAGAGAAACTCCACTACCAAAACCATCATTAAAACAAAAATTTACATGCCAC<br>TTAACTCCCCTCACAAACAATCGTTATTTATATTGTTAATTAGCAAACACAAAACCCGC

CamHap_65	ATTTTATTTTTTAACCTAACTCCCCTACTAAGTGTACCCCCCCTTTCCCCCCCAGGGGGGGTATACTATGCATAATCGTGCATACATTTATATACCACATATATTATGGTACCGGTAATATATACTATATATGTACTAAACCCATTAT<br>ATGTATACGGACATTAATCTATATTCCACATTTCTCCCAATGTCCATTCTATGCATGATCCAGGACATACTCATTCACCCTCCCCATAGACAGCCCCAAACCACTACCAAGTCACCTAACTATGAATGGTTGCAGGACATAAATCT<br>CACTCTCATGCTCTTCCCCCAACAAGTCACCTAACTATGAATGGTTACAGGACATACATTTAACTACC--<br>ATGTTCTAACCCATTTGGTTATGCTCGCCGTATCAGATGGATTTATTGATCGTCCACCTCACGAGAGATCAGCAACCCCTGCCTGTAATGTACTTCATGACCAGTCTCAGGCCCATTCTTTCCCCCTACACCCCTCGCCCTACTTG<br>CCTTCCACCGTACCTCTGGTTCCTCGGTCAGGCACATCCCATGCATAACTCCTGAACTTTCTCACTTTTCACGAAGTCATCTGTGGATTATCTTCCCCTCTTTAGTCCGTGATCGCGGCATCTTCTCTCTTCTATTGCTGTTGGTTC<br>CTTCTCTTTTTGGGGCTTCTTCACAGGTTGCCCTTCACAGTGCGGGTGCGGAGTGCTATTCAAGTGAAGCCTGGACTACACCTGCGTTGCGTCCTATCCTAGTCCTCTCGTGTCCCTCGATGAGACGGTTTGCGTGTATGGGGAAT<br>CATCTTGACACTGATGCACTTTGGATCGCATTTGGTTATGGTTCTTCCACCCCCCC--<br>GGTAAATGGTGCTATTTAGTGAATGCTTGTCGGACATATTTTTATCAATTTTCACTTCCTCTATTTTCTTCACAAAACTAGGAAATTCACCACAATTTTTTCTTTGTTATTTTTTAATTTTTTTTTTATTTTTTAAAAACATTTTTTAAA<br>AAACTAAATTACATACAAACTACCGCATAAAATCCCTCAAACTATACAAACGTTTATCGTATAATATATATACATTATTGTTTATTCTATCATTATTAGAGAAACTCCACTACCAAAACCATCATTAAAACAAAAATTTACATGCCAC<br>TTAACTCCCCTCACAAACAATCGTTATTTATATTGTTAATTAGCAAACACAAAACCCGC
CamHap_66	ATTTTATTTTTTAACCTAACTCCCCTACTAAGTGTACCCCCCCTTTCCCCCCCAGGGGGGGTATACTATGCATAATCGTGCATACATTTATATACCACATATATTATGGTACCGGTAATATATACTATATATGTACTAAACCCATTAT<br>ATGTATACGGGCATTAACCTATATTCCACATTTCTCCCAATGTCCATTCTATGCATGATCCAGGACATACTCATTTACCCTCCCCATAGACAGTTCCAAACCACTATCAAGCCACCTAACTATGAATGGTTACAGGACATAAATCTC<br>ACTCTCATGTTCTCCCCCCAACAAGTCACCTAACTATGAATGGTTACAGGACATACATTTAACTACC--<br>ATGTTCTAACCCATTTGGTTATGCTCGCCGTATCAGATGGATTTATTGATCGTCCACCTCACGAGAGATCAGCAACCCCTGCCTGTAATGTACTTCATGACCAGTCTCAGGCCCATTCTTTCCCCCTACACCCCTCGCCCTACTTG<br>CCTTCCACCGTACCTCTGGTTCCTCGGTCAGGCACATCCCATGCATAACTCCTGAACTTTCTCACTTTTCACGAAGTCATCTGTGGATTATCTTCCCCTCTTTAGTCCGTGATCGCGGCATCTTCTCTCTTCTATTGCTGTTGGTTC<br>CTTCTCTTTTTGGGGCTTCTTCACAGGTTGCCCTTCACAGTGCGGGTGCGGAGTGCTATTCAAGTGAAGCCTGGACTACACCTGCGTTGCGTCCTATCCTAGTCCTCTCGTGTCCCTCGATGAGACGGTTTGCGTGTATGGGGAAT<br>CATCTTGACACTGATGCACTTTGGATCGCATTTGGTTATGGTTCTTCCACCCCCCC--<br>GGTAAATGGTGCTATTTAGTGAATGCTTGTCGGACATATTTTTATCAATTTTCACTTCCTCTATTTTCTTCACAAAACTAGGAAATTCACCACAATTTTTTCTTTGTTATTTTTTAATTTTTTTTTTATTTTTTAAAAACATTTTTTAAA<br>AAACTAAATTACATACAAACTACCGCATAAAATCCCTCAAACTATACAAACGTTTATCGTATAATATATATACATTATTGTTTATTCTATCATTATTAGAGAAACTCCACTACCAAAACCATCATTAAAACAAAAATTTACATGCCAC<br>TTAACTCCCCTCACAAACAACCGTTATTTATATTGTTAATTAGCAAACACAAAACCCGC
CamHap_67	ATTTTATTTTTTAACCTAACTCCCCTACTAAGTGTACCCCCCCTTTCCCCCCCAGGGGGGGTATACTATGCATAATCGTGCATACATTTATATACCACATATATTATGGTACCGGTAATATATACTATATATGTACTAAACCCATTAT<br>ATGTATACGGGCATTAATCTATATTCCACATTTCTCCCAATGTCCATTCTATGCATGATCCAGGACATACTCATTCACCCTCCCCATAGACAGCTCCAAACCACTACCAAGTCACCTAACTATGAATGGTTGCAGGACATAAATCTC<br>ACTCTCATGCTCTTCCCCCAACAAGTCACCTAACTATGAATGGTTACAGGACATACATTTAACTACC--<br>ATGTTCTAACCCATTTGGTTATGCTCGCCGTATCAGATGGATTTATTGATCGTCCACCTCACGAGAGATCAGCAACCCCTGCCTGTAATGTACTTCATGACCAGTCTCAGGCCCATTCTTTCCCCCTACACCCCTCGCCCTACTTG<br>CCTTCCACCGTACCTCTGGTTCCTCGGTCAGGCACATCCCATGCATAACTCCTGAACTTTCTCACTTTTCACGAAGTCATCTGTGGATTATCTTCCCCTCTTTAGTCCGTGATCGCGGCATCTTCTCTCTTCTATTGCTGTTGGTTC<br>CTTCTCTTTTTGGGGCTTCTTCACAGGTTACCCTTCACAGTGCGGGTGCGGAGTGCTATTCAAGTGAAGCCTGGACTACACCTGCGTTGCGTCCTATCCTAGTCCTCTCGTGTCCCTCGATGAGACGGTTTGCGTGTATGGGGAAT<br>CATCTTGACACTGATGCACTTTGGATCGCATTTGGTTATGGTTCTTCCACCCCCCC--<br>GGTAAATGGTGCTATTTAGTGAATGCTTGTCGGACATATTTTTATCAATTTTCACTTCCTCTATTTTCTTCACAAAACTAGGAAATTCACCACAATTTTTTCTTTGTTATTTTTTAATTTTTTTTTTATTTTTTAAAAACATTTTTTAAA<br>AAACTAAATTACATACAAACTACCGCATAAAATCCCTCAAACTATACAAACGTTTATCGTATAATATATATACATTATTGTTTATTCTATCATTATTAGAGAAACTCCACTACCAAAACCATCATTAAAACAAAAATTTACATGCCAC<br>TTAACTCCCCTCACAAACAATCGTTATTTATATTGTTAATTAGCAAACACAAAACCCGC
CamHap_68	ATTTTATTTTTTAACCTAACTCCCCTACTAAGTGTACCCCCCCTTTCCCCCCCAGGGGGGGTATACTATGCATAATCGTGCATACATTTATATACCACATATATTATGGTACCGGTAATATATACTATATATGTACTAAACCCATTAT<br>ATGTATACGGGCATTAACCTATATTCCACATTTCTCCCAATGTCCATTCTATGCATGATCCAGGACATACTCATTTACCCTCCCCATAGACAGTTCCAAACCACTATCAAGCCACCTAACTATGAATGGTTACAGGACATAAATCTC<br>ACTCTCATGTTCTCCCCCCAACAAGTCACCTAACTATGAATGGTTACAGGACATACATTTAACTACC--<br>ATGTTCTAACCCATTTGGTTATGCTCGCCGTATCAGATGGATTTATTGATCGTCCACCTCACGAGAGATCAGCAACCCCTGCCTGTAATGTACTTCATGACCAGTCTCAGGCCCATTCTTTCCCCCTACACCCCTCGCCCTACTTG<br>CCTTCCACCGTACCTCTGGTTCCTCGGTCAGGCACATCCCATGCATAACTCCTGAACTTTCTCACTTTTCACGAAGTCATCTGTGGATTATCTTCCCCTCTTTAGTCCGTGATCGCGGCATCTTCTCTCTTCTATTGCTGTTGGTTC<br>CTTCTCTTTTTGGGGCTTCTTCACAGGTTACCCTTCACAGTGCGGGTGCGGAGTGCTATTCAAGTGAAGCCTGGACTACACCTGCGTTGCGTCCTATCCTAGTCCTCTCGTGTCCCTCGATGAGACGGTTTGCGTGTATGGGGAAT<br>CATCTTGACACTGATGCACTTTGGATCGCATTTGGTTATGGTTCTTCCACCCCCCC--<br>GGTAAATGGTGCTATTTAGTGAATGCTTGTCGGACATATTTTTATCAATTTTCACTTCCTCTATTTTCTTCACAAAACTAGGAAATTCACCACAATTTTTTCTTTGTTATTTTTTAATTTTTTTTTTATTTTTTAAAAACATTTTTTAAA<br>AAACTAAATTACATACAAACTACCGCATAAAATCCCTCAAACTATACAAACGTTTATCGTATAATATATATACATTATTGTTTATTCTATCATTATTAGAGAAACTCCACTACCAAAACCATCATTAAAACAAAAATTTACATGCCAC<br>TTAACTCCCCTCACAAACAACCGTTATTTATATTGTTAATTAGCAAACACAAAACCCGC

CamHap_69	ATTTTATTTTTTAACCTAACTCCCCTACTAAGTGTACCCCCCCTTTCCCCCCCAGGGGGGGTATACTATGCATAATCGTGCATACATTTATATACCACATATATTATGGTACCGGTAATATATACTATATATGTACTAAACCCATTAT<br>ATGTATACGGGCATTAATCTATATTCCACATTTCTCCCAATGTCCATTCTATGCATGATCCAGGACATACTTATTCACCCTCCCCATAGACAGCTCCAAACCACTACCAAGTCACCTAACTATGAATGGTTGCAGGACATAAATCTC<br>ACTCTCATGCTCTTCCCCCAACAAGTCACCTAACTATGAATGGTTACAGGACATACATTTAACTACC--<br>ATGTTCTAACCCATTTGGTTATGCTCGCCGTATCAGATGGATTTATTGATCGTCCACCTCACGAGAGATCAGCAACCCCTGCCTGTAATGTACTTCATGACCAGTCTCAGGCCCATTCTTTCCCCCTACACCCCTCGCCCTACTTG<br>CCTTCCACCGTACCTCTGGTTCCTCGGTCAGGCACATCCCATGCATAACTCCTGAACTTTCTCACTTTTCACGAAGTCATCTGTGGATTATCTTCCCCTCTTTAGTCCGTGATCGCGGCATCTTCTCTCTTCTATTGCTGTTGGTTC<br>CTTCTCTTTTTGGGGCTTCTTCACAGGTTGCCCTTCACAGTGCGGGTGCGGAGTGCTATTCAAGTGAAGCCTGGACTACACCTGCGTTGCGTCCTATCCTAGTCCTCTCGTGTCCCTCGATGAGACGGTTTGCGTGTATGGGGAA<br>TCATCTTGACACTGATGCACTTTGGATCGCATTTGGTTATGGTTCTTCCACCCCCCC--<br>GGTAAATGGTGCTATTTAGTGAATGCTTGTCGGACATATTTTTATCAATTTTCACTTCCTCTATTTTCTTCACAAAACTAGGAAATTCACCACAATTTTTTCTTTGTTATTTTTTAATTTTTTTTTTATTTTTTAAAAACATTTTTTAAA<br>AAACTAAATTACATACAAACTACCGCATAAAATCCCTCAAACTATACAAACGTTTATCGTATAATATATATACATTATTGTTTATTCTATCATTATTAGAGAAACTCCACTACCAAAACCATCATTAAAACAAAAATTTACATGCCAC<br>TTAACTCCCCTCACAAACAATCGTTATTTATATTGTTAATTAGCAAACACAAAACCCGC
CamHap_70	ATTTTATTTTTTAACCTAACTCCCCTACTAAGTGTACCCCCCCTTTCCCCCCCAGGGGGGGTATACTATGCATAATCGTGCATACATTTATATACCACATATATTATGGTACCGGTAATATATACTATATATGTACTAAACCCATTAT<br>ATGTATACGGGCATTAATCTATATTCCACATTTCTCCCAATGTCCATTCTATGCATGATCCAGGACATACTCATTCACCCTCCCCATAGACAACTCCAAACCACTACCAAGCCACCTAACTATGAATGGTTGCAGGACATAAATCT<br>CACTCTCATGTTCTTCCCCCAACAAGTCACCTAACTATGAATGGTTGCAGGACATACATTTAACTACT--<br>ATGCTCTAACCCATTTGGTTATGCTCGCCGTATCAGATGGATTTATTGATCGTCCACCTCACGAGAGATCAGCAACCCCTGCCTGTAATGTACTTCATGACCAGTCTCAGGCCCATTCTTTCCCCCTACACCCCTCGCCCTACTTG<br>CCTTCCACCGTACCTCTGGTTCCTCGGTCAGGCACATCCCATGCATAACTCCTGAACTTTCTCACTTTTCACGAAGTCATCTGTGGATTATCTTCCCCTCTTTAGTCCGTGATCGCGGCATCTTCTCTCTTCTATTGCTGTTGGTTC<br>CTTCTCTTTTTGGGGCTTCTTCACAGGTTGCCCTTCACAGTGCGGGTGCGGAGTGCTATTCAAGTGAAGCCTGGACTACACCTGCGTTGCGTCCTATCCTAGTCCTCTCGTGTCCCTCGATGAGACGGTTTGCGTGTATGGGGAAT<br>CATCTTGACACTGATGCACTTTGGATCGCATTTGGTTATGGTTCTTCCACCCCCCC-<br>GGTAAATGGTGCTATTTAGTGAATGCTTGTCGGACATATTTTTATCAATTTTCACTTCCTCTATTTTCTTCACAAAACTAGGAAATTCACCACAATTTTTTCTTTGTTATTTTTTAATTTTTTTTTTATTTTTTAAAAACATTTTTTAAA<br>AAACTAAATTACATACAAACTACCGCATAAAATCCCTCAAACTATACAAACGTTTATCGTATAATATATATACATTATTGTTTATTCTATCATTATTAGAGAAACTCCACTACCAAAACCATCATTAAAACAAAAATTTACATGCCAC<br>TTAACTCCCCTCACAAACAATCGTTATTTATATTGTTAATTAGCAAACACAAAACCCGC
CamHap_71	ATTTTATTTTTTAACCTAACTCCCCTACTAAGTGTACCCCCCCTTTCCCCCCCAGGGGGGGTATACTATGCATAATCGTGCATACATTTATATACCACATATATTATGGTACCGGTAATATATACTATATATGTACTAAACCCATTAT<br>ATGTATACGGGCATTAACCTATATTCCACATTTCTCCCAATGTCCATTCCATGCATGATCCAGGACATACTCATTTACCCTCCCCATAGACAGTTCTAAACCACTATCAAGCCACCTAACTATGAATGGTTACAGGACATAAATCTC<br>ACTCTCATGTTCTCCCCCCAACAAGTCACCTAACTATGAATGGTTACAGGACATACATTTAACTACC--<br>ATGTTCTAACCCATTTGGTTATGCTCGCCGTATCAGATGGATTTATTGATCGTCCACCTCACGAGAGATCAGCAACCCCTGCCTGTAATGTACTTCATGACCAGTCTCAGGCCCATTCTTTCCCCCTACACCCCTCGCCCTACTTG<br>CCTTCCACCGTACCTCTGGTTCCTCGGTCAGGCACATCCCATGCATAACTCCTGAACTTTCTCACTTTTCACGAAGTCATCTGTGGATTATCTTCCCCTCTTTAGTCCGTGATCGCGGCATCTTCTCTCTTCTATTGCTGTTGGTTC<br>CTTCTCTTTTTGGGGCTTCTTCACAGGTTGCCCTTCACAGTGCGGGTGCGGAGTGCTATTCAAGTGAAGCCTGGACTACACCTGCGTTGCGTCCTATCCTAGTCCTCTCGTGTCCCTCGATGAGACGGTTTGCGTGTATGGGGAAT<br>CATCTTGACACTGATGCACTTTGGATCGCATTTGGTTATGGTTCTTCCACCCCCCC--<br>GGTAAATGGTGCTATTTAGTGAATGCTTGTCGGACATATTTTTATCAATTTTCACTTCCTCTATTTTCTTCACAAAACTAGGAAATTCACCACAATTTTTTCTTTGTTATTTTTTAATTTTTTTTTTATTTTTTAAAAACATTTTTTAAA<br>AAACTAAATTACATACAAACTACCGCATAAAATCCCTCAAACTATACAAACGTTTATCGTATAATATATATACATTATTGTTTATTCTATCATTATTAGAGAAACTCCACTACCAAAACCATCATTAAAACAAAAATTTACATGCCAC<br>TTAACTCCCCTCACAAACAATCGTTATTTATATTGTTAATTAGCAAACACAAAACCCGC
CamHap_72	ATTTTATTTTTTAACCTAACTCCCCTACTAAGTGTACCCCCCCTTTCCCCCCCAGGGGGGGTATACTATGCATAATCGTGCATACATTTATATACCACATATATTATGGTACCGGTAATATATACTATATATGTACTAAACCCATTAT<br>ATGTATACGGGCATTAATCTATATTCCACATTTCTCCCAATGTCCATTCTATGCATGATCCAGGACACACTCATTCACCCTCCCCATAGACAGCTCCAAACCACTACCAAGTCACCTAACTATGAATGGTTACAGGACATAAATCT<br>CACTCTCATGTTCTTCCCCCAACAAGTCACCTAACTATGAATGGTTACAGGACATACATTTAACTACC--<br>ATGTTCTAACCCATTTGGTTATGCTCGCCGTATCAGATGGATTTATTGATCGTCCACCTCACGAGAGATCAGCAACCCCTGCTTGTAATGTACTTCATGACCAGTCTCAGGCCCATTCTTTCCCCCTACACCCCTCGCCCTACTTG<br>CCTTCCACCGTACCTCTGGTTCCTCGGTCAGGCACATCCCATGCATAACTCCTGAACTTTCTCACTTTTCACGAAGTCATCTGTGGATTATCTTCCCCTCTTTAGTCCGTGATCGCGGCATCTTCTCTCTTCTATTGCTGTTGGTTC<br>CTTCTCTTTTTGGGGCTTCTTCACAGGTTGCCCTTCACAGTGCGGGTGCGGAGTGCTATTCAAGTGAAGCCTGGACTACACCTGCGTTGCGTCCTATCCTAGTCCTCTCGTGTCCCTCGATGAGACGGTTTGCGTGTATGGGGAAT<br>CATCTTGACACTGATGCACTTTGGATCGCATTTGGTTATGGTTCTTCCACCCCCCC--<br>GGTAAATGGTGCTATTTAGTGAATGCTTGTCGGACATATTTTTATCAATTTTCACTTCCTCTATTTTCTTCACAAAACTAGGAAATTCACCACAATTTTTTCTTTGTTATTTTTTAATTTTTTTTTTATTTTTTAAAAACATTTTTTAAA<br>AAACTAAATTACATACAAACTACCGCATAAAATCCCTCAAACTATACAAACGTTTATCGTATAATATATATACATTATTGTTTATTCTATCATTATTAGAGAAACTCCACTACCAAAACCATCATTAAAACAAAAATTTACATGCCAC<br>TTAACTCCCCTCACAAACAATCGTTATTTATATTGTTAATTAGCAAACACAAAACCTGC

CamHap_73	ATTTTATTTTTTAACCTAACTCCCCTACTAAGTGTACCCCCCCTTTCCCCCCCAGGGGGGGTATACTATGCATAATCGTGCATACATTTATATACCACATATATTATGGTACCGGTAATATATACTATATATGTACTAAACCCATTAT<br>ATGTATACGGGCATTAATCTATATTCCACATTTCTCCCAATGTCCATTCTATGCATGATCCAGGACATACTCATTCACCCTCCCCATAGACAGCTCCAAACCACTACCAAGCCACCTAACTATGAATGGTTGCAGGACATAAATCT<br>CACTCTCATGCTCTTCCCCCAACAAGTCACCTAACTATGAATGGTTACAGGACATACATTTAACTACC--<br>ATGTTCTAACCCATTTGGTTATGCTCGTCGTATCAGATGGATTTATTGATCGTCCACCTCACGAGAGATCAGCAACCCCTGCCTGTAATGTACTTCATGACCAGTCTCAGGCCCATTCTTTCCCCCTACACCCCTCGCCCTACTTG<br>CCTTCCACCGTACCTCTGGTTCCTCGGTCAGGCACATCCCATGCATAACTCCTGAACTTTCTCACTTTTCACGAAGTCATCTGTGGATTATCTTCCCCTCTTTAGTCCGTGATCGCGGCATCTTCTCTCTTCTATTGCTGTTGGTTC<br>CTTCTCTTTTTGGGGCTTCTTCACAGGTTGCCCTTCACAGTGCGGGTGCGGAGTGCTATTCAAGTGAAGCCTGGACTACACCTGCGTTGCGTCCTATCCTAGTCCTCTCGTGTCCCTCGATGAGACGGTTTGCGTGTATGGGGAAT<br>CATCTTGACACTGATGCACTTTGGATCGCATTTGGTTATGGTTCTTCCACCCCCCC--<br>GGTAAATGGTGCTATTTAGTGAATGCTTGTCGGACATATTTTTATCAATTTTCACTTCCTCTATTTTCTTCACAAAACTAGGAAATTCACCACAATTTTTTCTTTGTTATTTTTTAATTTTTTTTTTATTTTTTAAAAACATTTTTTAAA<br>AAACTAAATTACATACAAACTACCGCATAAAATCCCTCAAACTATACAAACGTTTATCGTATAATATATATACATTATTGTTTATTCTATCATTATTAGAGAAACTCCACTACCAAAACCATCATTAAAACAAAAATTTACATGCCAC<br>TTAACTCCCCTCACAAACAATCGTTATTTATATTGTTAATTAGCAAACACAAAACCCGC
CamHap_74	ATTTTATTTTTTAACCTAACTCCCCTACTAAGTGTACCCCCCCTTTCCCCCCCAGGGGGGGTATACTATGCATAATCGTGCATACATTTATATACCACATATATTATGGTACCGGTAATATATACTATATATGTACTAAACCCATTAT<br>ATGTATACGGGCATTAACCTATATTCCACATTTCTCCCAATGTCCATTCCATGCATGATCCAGGACATACTCATTTACCCTCCCCATAGACAGTTCCAAACCACTATCAAGCCACCTAACTATGAATGGTTACAGGACATAAATCTC<br>ACTCTCATGTTCTCCCCCCAACAAGTCACCTAACTATGAATGGTTACAGGACATACATTTAACTACC--<br>ATGTTCTAACCCATTTGGTTATGCTCGCCGTATCAGATGGATTTATTGATCGTCCACCTCACGAGAGATCAGCAACCCCTGCCTGTAATGTACTTCATGACCAGTCTCAGGCCCATTCTTTCCCCCTACACCCCTCGCCCTACTTG<br>CCTTCCACCGTACCTCTGGTTCCTCGGTCAGGCACATCCCATGCATAACTCCTGAACTTTCTCACTTTTCACGAAGTCATCTGTGGATTATCTTCCCCTCTTTAGTCCGTGATCGCGGCATCTTCTCTCTTCTATTGCTGTTGGTTC<br>CTTCTCTTTTTGGGGCTTCTTCACAGGTTGCCCTTCACAGTGCGGGTGCGGAGTGCTATTCAAGTGAAGCCTGGACTACACCTGCGTTGCGTCCTATCCTAGTCCTCTCGTGTCCCTCGATGAGACGGTTTGCGTGTATGGGGAAT<br>CATCTTGACACTGATGCACTTTGGATCGCATTTGGTTATGGTTCTTCCACCCCCCC--<br>GGTAAATGGTGCTATTTAGTGAATGCTTGTCGGACATATTTTTATCAATTTTCACTTCCTCTATTTTCTTCACAAAACTAGGAAATTCACCACAATTTTTTCTTTGTTATTTTTTAATTTTTTTTTTATTTTTTAAAAACATTTTTTAAA<br>AAACTAAATTACATACAAACTACCGCATAAAATCCCTCAAACTATACAAACGTTTATCGTATAATATATATACATTATTGTTTATTCTATCATTATTAGAGAAACTCCACTACCAAAACCATCATTAAAACAAAAATTTACATGCCAC<br>TTAACTCCCCTCACAAACAATCGTTATTTATATTGTTAATTAGCAAACACAAAACCCGC
CamHap_75	ATTTTATTTTTTAACCTAACTCCCCTACTAAGTGTACCCCCCCTTTCCCCCCCAGGGGGGGTATACTATGCATAATCGTGCATACATTTATATACCACATATATTATGGTACCGGTAATATATACTATATATGTACTAAACCCATTAT<br>ATGTATACGGGCATTAATCTATATTCCACATTTCTCCCAATGTCCATTCTATGCATGATCCAGGACATACTCATTCACCCTCCCCATAGACAGCTCCAAACCACTACCAAGTCACCTAACTATGAATGGTTGCAGGACATAAATCTC<br>ACTCTCATGCTCTTCCCCCAACAAGTCACCTAACTATGAATGGTTACAGGACATACATTTAACTACC--<br>ATGTTCTAACCCATTTGGTTATGCTCGCCGTATCAGATGGATTTATTGATCGTCCACCTCACGAGAGATCAGCAACCCCTGCCTGTAATGTACTTCATGACCAGTCTCAGGCCCATTCTTTCCCCCTACACCCCTCGCCCTACTTG<br>CCTTCCACCGTACCTCTGGTTCCTCGGTCAGGCACATCCCATGCATAACTCCTGAACTTTCTCACTTTTCACGAAGTCATCTGTGGATTATCTTCCCCTCTTTAGTCCGTGATCGCGGCATCTTCTCTCTTCTATTGCTGTTGGTTC<br>CTTCTCTTTTTGGGGCTTCTTCACAGGTTGCCCTTCACAGTGCGGGTGCGGAGTGCTATTCAAGTGAAGCCTGGACTACACCTGCGTTGCGTCCTATCCTAGTCCTCTCGTGTCCCTCGATGAGACGGTTTGCGTGTATGGGGAAT<br>CATCTTGACACTGATGCACTTTGGATCGCATTTGGTTATGGTTCTTCCACCCCCCC--<br>GGTAAATGGTGCTATTTAGTGAATGCTTGTCGGACATATTTTTATCAATTTTCACTTCCTCTATTTTCTTCACAAAACTAGGAAATTCACCACAATTTTTTCTTTGTTATTTTTTAATTTTTTTTTTATTTTTTAAAAACATTTTTTAAA<br>AAACTAAATTACATACAAACTACCGCATAAAATCCCTCAAACTATACAAACGTTTATCGTATAATATATATACATTATTGTTTATTCTATCATTATTAGAGAAACTCCACTACCAAAACCATCATTAAAACAAAAATTTACATGCCAC<br>TTAACTCCCCTCACAAACAATCGTTATTTATATTGTTAATTAGCAAACACAAAACCCAC
CamHap_76	ATTTTATTTTTTAACCTAACTCCCCTACTAAGTGTACCCCCCCTTTCCCCCCCAGGGGGGGTATACTATGCATAATCGTGCATACATTTATATACCACATATATTATGGTACCGGTAATATATACTATATATGTACTAAACCCATTAT<br>ATGTATACGGGCATTAACCTATATTCCACATTTCTCCCAATGTCCATTCTATGCATGATCCAGGACATACTCATTTACCCTCCCCATAGACAGTTCCAAACCACTATCAAGCCACCTAACTATGAATGGTTACAGGACATAAATCTC<br>ACTCTCATGTTCTCCCCCCAACAAGTCACCTAACTATGAATGGTTACAGGACATACATTTAACTACC--<br>ATGTTCTAACCCATTTGGTTATGCTCGCCGTATCAGATGGATTTATTGATCGTCCACCTCACGAGAGATCAGCAACCCCTGCCTGTAATGTACTTCATGACCAGTCTCAGGCCCATTCTTTCCCCCTACACCCCTCGCCCTACTTG<br>CCTTCCACCGTACCTCTGGTTCCTCGGTCAGGCACATCCCATGCATAACTCCTGAACTTTCTCACTTTTCACGAAGTCATCTGTGGATTATCTTCCCCTCTTTAGTCCGTGATCGCGGCATCTTCTCTCTTCTATTGCTGTTGGTTC<br>CTTCTCTTTTTGGGGCTTCTTCACAGGTTGCCCTTCACAGTGCGGGTGCGGAGTGCTATTCAAGTGAAGCCTGGACTACACCTGCGTTGCGTCCTATCCTAGTCCTCTCGTGTCCCTCGATGAGACGGTTTGCGTGTATGGGGAAT<br>CATCTTGACACTGATGCACTTTGGATCGCATTTGGTTATGGTTCTTCCACCCCCCCC-<br>GGTAAATGGTGCTATTTAGTGAATGCTTGTCGGACATATTTTTATCAATTTTCACTTCCTCTATTTTCTTCACAAAACTAGGAAATTCACCACAATTTTTTCTTTGTTATTTTTTAATTTTTTTTTTATTTTTTAAAAACATTTTTTAAA<br>AAACTAAATTACATACAAACTACCGCATAAAATCCCTCAAACTATACAAACGTTTATCGTATAATATATATACATTATTGTTTATTCTATCATTATTAGAGAAACTCCACTACCAAAACCATCATTAAAACAAAAATTTACATGCCAC<br>TTAACTCCCCTCACAAACAATCGTTATTTATATTGTAAATTAGCAAACACAAAACCCGC

CamHap_77	ATTTTATTTTTTAACCTAACTCCCCTACTAAGTGTACCCCCCCTTTCCCCCCCAGGGGGGGTATACTATGCATAATCGTGCATACATTTATATACCACATATATTATGGTACCGGTAATATATACTATATATGTACTAAACCCATTAT<br>ATGTATACGGGCATTAATCTATATTCCACATTTCTCCCAATGTCCATTCTATGCATGATCCAGGACATACTCATTCACCCTCCCCATAGACAGCTCCAAACCACTACCAAGTCACCTAACTATGAATGGTTGCAGGACATAAATCTC<br>ACTCTCATGCTCTTCCCCCAACAAGTCACCTAACTATGAATGGTTACAGGACATACATTTAACTACC---<br>ATGTTCTAACCCATTTGGTTATGCTCGCCGTATCAGATGGATTTATTGATCGTCCACCTCACGAGAGATCAGCAACCCCTGCCTGTAATGTACTTCATGACCAGTCTCAGGCCCATTCTTTCCCCCTACACCCCTCGCCCTACTTG<br>CCTTCCACCGTACCTCTGGTTCCTCGGTCAGGCACATCCCATGCATAACTCCTGAACTTTCTCACTTTTCACGAAGTCATCTGTGGATTATCTTCCCCTCTTTAGTCCGTGATCGCGGCATCTTCTCTCTTCTATTGCTGTTGGTTC<br>CTTCTCTTTTTGGGGCTTCTTCACAGGTTGCCCTTCACAGTGCGGGTGCGGAGTGCTATTCAAGTGAAGCCTGGACTACACCTGCGTTGCGTCCTATCCTAGTCCTCTCGTGTCCCTCGATGAGACGGTTTGCGTGTATGGGGAAT<br>CATCTTGACACTGATGCACTTTGGATCGCATTTGGTTATGGTTCTTCCACCCCCCC--<br>GGTAAATGGTGCTATTTAGTGAATGCTTGTCGGACATATTTTTATCAATTTTCACTTCCTCTATTTTCTTCACAAAACTAGGAAATTCACCACAATTTTTTCTTTGTTATTTTTTAATTTTTTTTTTATTTTTTAAAAACATTTTTTAAA<br>AAACTAAATTACATACAAACTACCGCATAAAATCCCTCAAACTATACAAACGTTTATCGTATAATATATATACATTATTGTTTATTCTATCATTATTAGAGAAACTCCACTACCAAAACCATCATTAAAACAAAAATTTACATGCCAC<br>TTAACTCCCCTCACAAACAATCGTTATTTATATTGTAAATTAGCAAACACAAAACCCGC
CamHap_78	ATTTTATTTTTTAACCTAACTCCCCTACTAAGTGTACCCCCCCTTTCCCCCCCAGGGGGGGTATACTATGCATAATCGTGCATACATTTATATACCACATATATTATGGTACCGGTAATATATACTATATATGTACTAAACCCATTAT<br>ATGTATACGGGCATTAATCTATATTCCACATTTCTCCCAATGTCCATTCTATGCATGATCCAGGACATACTCATTCACCCTCCCCATAGACAGCTCCAAACCACTACCAAGTCACCTAACTATGAATGGTTGCAGGACATAAATCTC<br>ACTCTCATGCTCTTCCCCCAACAAGTCACCTAACTATGAATGGTTGCAGGACATACATTTAACTACC---<br>ATGTTCTAACCCATTTGGTTATGCTCGCCGTATCAGATGGATTTATTGATCGTCCACCTCACGAGAGATCAGCAACCCCTGCCTGTAATGTACTTCATGACCAGTCTCAGGCCCATTCTTTCCCCCTACACCCCTCGCCCTACTTG<br>CCTTCCACCGTACCTCTGGTTCCTCGGTCAGGCACATCCCATGCATAACTCCTGAACTTTCTCACTTTTCACGAAGTCATCTGTGGATTATCTTCCCCTCTTTAGTCCGTGATCGCGGCATCTTCTCTCTTCTATTGCTGTTGGTTC<br>CTTCTCTTTTTGGGGCTTCTTCACAGGTTGCCCTTCACAGTGCGGGTGCGGAGTGCTATTCAAGTGAAGCCTGGACTACACCTGCGTTGCGTCCTATCCTAGTCCTCTCGTGTCCCTCGATGAGACGGTTTGCGTGTATGGGGAAT<br>CATCTTGACACTGATGCACTTTGGATCGCATTTGGTTATGGTTCTTCCACCCCCCC--<br>GGTAAATGGTGCTATTTAGTGAATGCTTGTCGGACATATTTTTATCAATTTTCACTTCCTCTATTTTCTTCACAAAACTAGGAAATTCACCACAATTTTTTCTTTGTTATTTTTTAATTTTTTTTTTATTTTTTAAAAACATTTTTTAAA<br>AAACTAAATTACATACAAACTACCGCATAAAATCCCTCAAACTATACAAACGTTTATCGTATAATATATATACATTATTGTTTATTCTATCATTATTAGAGAAACTCCACTACCAAAACCATCATTAAAACAAAAATTTACATGCCAC<br>TTAACTCCCCTCACAAACAATCGTTATTTATATTGTTAATTAACAAACACAAAACCCGC
CamHap_79	ATTTTATTTTTTAACCTAACTCCCCTACTAAGTGTACCCCCCCTTTCCCCCCCAGGGGGGGTATACTATGCATAATCGTGCATACATTTATATACCACATATATTATGGTACCGGTAATATATACTATATATGTACTAAACCCATTAT<br>ATGTATACGGGCATTAATCTATATTCCACATTTCTCCCAATGTCCATTCTATGCATGATCCAGGACACACTCATTCACCCTCCCCATAGACAGCTCCAAACCACTACCAAGTCACCTAACTATGAATGGTTGCAGGACATAAATCT<br>CACTCTCATGCTCTTCCCCCAACAAGTCACCTAACTATGAATGGTTACAGGACATACATTTAACTACC---<br>ATGTTCTAACCCATTTGGTTATGCTCGCCGTATCAGATGGATTTATTGATCGTCCACCTCACGAGAGATCAGCAACCCCTGCTTGTAATGTACTTCATGACCAGTCTCAGGCCCATTCTTTCCCCCTACACCCCTCGCCCTACTTG<br>CCTTCCACCGTACCTCTGGTTCCTCGGTCAGGCACATCCCATGCATAACTCCTGAACTTTCTCACTTTTCACGAAGTCATCTGTGGATTATCTTCCCCTCTTTAGTCCGTGATCGCGGCATCTTCTCTCTTCTATTGCTGTTGGTTC<br>CTTCTCTTTTTGGGGCTTCTTCACAGGTTGCCCTTCACAGTGCGGGTGCGGAGTGCTATTCAAGTGAAGCCTGGACTACACCTGCGTTGCGTCCTATCCTAGTCCTCTCGTGTCCCTCGATGAGACGGTTTGCGTGTATGGGGAAT<br>CATCTTGACACTGATGCACTTTGGATCGCATTTGGTTATGGTTCTTCCACCCCCCC--<br>GGTAAATGGTGCTATTTAGTGAATGCTTGTCGGACATATTTTTATCAATTTTCACTTCCTCTATTTTCTTCACAAAACTAGGAAATTCACCACAATTTTTTCTTTGTTATTTTTTAATTTTTTTTTTATTTTTTAAAAACATTTTTTAAA<br>AAACTAAATTACATACAAACTACCGCATAAAATCCCTCAAACTATACAAACGTTTATCGTATAATATATATACATTATTGTTTATTCTATCATTATTAGAGAAACTCCACTACCAAAACCATCATTAAAACAAAAATTTACATGCCAC<br>TTAACTCCCCTCACAAACAATCGTTATTTATATTGTTAATTAGCAAACACAAAACCCGC
CamHap_80	ATTTTATTTTTTAACCTAACTCCCCTACTAAGTGTACCCCCCCTTTCCCCCCCAGGGGGGGTATACTATGCATAATCGTGCATACATTTATATACCACATATATTATGGTACCGGTAATATATACTATATATGTACTAAACCCATTAT<br>ATGTATACGGGCATTAACCTATATTCCACATTTCTCCCAATGTCCATTCTATGCATGATCCAGGACATACTCATTTACCCTCCCCATAGACAGTTCCAAACCACTATCAAGCCACCTAACTATGAATGGTTACAGGACATAAATCTC<br>ACTCTCATGTTCTCCCCCCAACAAGTCACCTAACTATGAATGGTTACAGGACATACATTTAACTACC---<br>ATGTTCTAACCCATTTGGTTATGCTCGCCGTATCAGATGGATTTATTGATCGTCCACCTCACGAGAGATCAGCAACCCCTGCCTGTAATGTACTTCATGACCAGTCTCAGGCCCATTCTTTCCCCCTACACCCCTCGCCCTACTTG<br>CCTTCCACCGTACCTCTGGTTCCTCGGTCAGGCACATCCCATGCATAACTCCTGAACTTTCTCACTTTTCACGAAGTCATCTGTGGATTATCTTCCCCTCTTTAGTCCGTGATCGCGGCATCTTCTCTCTTCTATTGCTGTTGGTTC<br>CTTCTCTTTTTGGGGCTTCTTCACAGGTTGCCCTTCACAGTGCGGGTGCGGAGTGCTATTCAAGTGAAGCCTGGACTACACCTGCGTTGCGTCCTATCCTAGTCCTCTCGTGTCCCTCGATGAGACGGTTTGCGTGTATGGGGAAT<br>CATCTTGACACTGATGCACTTTGGATCGCATTTGGTTATGGTTCTTCCACCCCCCC--<br>GGTAAATGGTGCTATTTAGTGAATGCTTGTCGGACATATTTTTATCAATTTTCACTTCCTCTATTTTCTTCACAAAACTAGGAAATTCACCACAATTTTTTCTTTGTTATTTTTTAATTTTTTTTTTATTTTTTAAAAACATTTTTTAAA<br>AAACTAAATTACATACAAACTACCGCATAAAATCCCTCAAACTATACAAACGTTTATCGTATAATATATATACATTATTGTTTATTCTATCATTATTAGAGAAACTCCACTACCAAAACCATCATTAAAACAAAAATTTACATGCCAC<br>TTAACTCCCCTCACAAACAATCGTTATTTATATTGTAAATTAGCAAACACAAAACCCGC

CamHap_81	ATTTTATTTTTTAACCTAACTCCCCTACTAAGTGTACCCCCCCTTTCCCCCCCAGGGGGGGTATACTATGCATAATCGTGCATACATTTATATACCACATATATTATGGTACCGGTAATATATACTATATATGTACTAAACCCATTAT<br>ATGTATACGGGCATTAATCTATATTCCACATTTCTCCCAATGTCCATTCTATGCATGATCCAGGACATACCCATTCACCCTCCCCATAGACAGCTCCAAACCACTACCAAGTCACCTAACTATGAATGGTTGCAGGACATAAATCT<br>CACTCTCATGCTCTTCCCCCAACAAGTCACCTAACTATGAATGGTTACAGGACATACATTTAACTACC--<br>ATGTTCTAACCCATTTGGTTATGCTCGCCGTATCAGATGGATTTATTGATCGTCCACCTCACGAGAGATCAGCAACCCCTGCCTGTAATGTACTTCATGACCAGTCTCAGGCCCATTCTTTCCCCCTACACCCCTCGCCCTACTTG<br>CCTTCCACCGTACCTCTGGTTCCTCGGTCAGGCACATCCCATGCATAACTCCTGAACTTTCTCACTTTTCACGAAGTCATCTGTGGATTATCTTCCCCTCTTTAGTCCGTGATCGCGGCATCTTCTCTCTTCTATTGCTGTTGGTTC<br>CTTCTCTTTTTGGGGCTTCTTCACAGGTTGCCCTTCACAGTGCGGGTGCGGAGTGCTATTCAAGTGAAGCCTGGACTACACCTGCGTTGCGTCCTATCCTAGTCCTCTCGTGTCCCTCGATGAGACGGTTTGCGTGTATGGGGAAT<br>CATCTTGACACTGATGCACTTTGGATCGCATTTGGTTATGGTTCTTCCACCCCCCC--<br>GGTAAATGGTGCTATTTAGTGAATGCTTGTCGGACATATTTTTATCAATTTTCACTTCCTCTATTTTCTTCACAAAACTAGGAAATTCACCACAATTTTTTCTTTGTTATTTTTTAATTTTTTTTTTATTTTTTAAAAACATTTTTTAAA<br>AAACTAAATTACATACAAACTACCGCATAAAATCCCTCAAACTATACAAACGTTTATCGTATAATATATATACATTATTGTTTATTCTATCATTATTAGAGAAACTCCACTACCAAAACCATCATTAAAACAAAAATTTACATGCCAC<br>TTAACTCCCCTCACAAACAATCGTTATTTATATTGTTAATTAGCAAACACAAAACCCGC
CamHap_82	ATTTTATTTTTTAACCTAACTCCCCTACTAAGTGTACCCCCCCTTTCCCCCCCAGGGGGGGTATACTATGCATAATCGTGCATACATTTATATACCACATATATTATGGTACCGGTAATATATACTATATATGTACTAAACCCATTAT<br>ATGTATACGGGCATTAATCTATATTCCACATTTCTCCCAATGTCCATTCTATGCATGATCCAGGACATACTCATTCACCCTCCCCATAGACAGCCCCAAACCACTACCAAGTCACCTAACTATGAATGGTTGCAGGACATAAATCT<br>CACTCTCATGCTCTTCCCCCAACAAGTCACCTAACTATGAATGGTTACAGGACATACATTTAACTACC--<br>ATGTTCTAACCCATTTGGTTATGCTCGCCGTATCAGATGGATTTATTGATCGTCCACCTCACGAGAGATCAGCAACCCCTGCCTGTAATGTACTTCATGACCAGTCTCAGGCCCATTCTTTCCCCCTACACCCCTCGCCCTACTTG<br>CCTTCCACCGTACCTCTGGTTCCTCGGTCAGGCACATCCCATGCATAACTCCTGAACTTTCTCACTTTTCACGAAGTCATCTGTGGATTATCTTCCCCTCTTTAGTCCGTGATCGCGGCATCTTCTCTCTTCTATTGCTGTTGGTTC<br>CTTCTCTTTTTGGGGCTTCTTCACAGGTTGCCCTTCACAGTGCGGGTGCGGAGTGCTATTCAAGTGAAGCCTGGACTACACCTGCGTTGCGTCCTATCCTAGTCCTCTCGTGTCCCTCGATGAGACGGTTTGCGTGTATGGGGAAT<br>CATCTTGACACTGATGCACTTTGGATCGCATTTGGTTATGGTTCTTCCACCCCCCC--<br>GGTAAATGGTGCTATTTAGTGAATGCTTGTCGGACATATTTTTATCAATTTTCACTTCCTCTATTTTCTTCACAAAACTAGGAAATTCACCACAATTTTTTCTTTGTTATTTTTTAATTTTTTTTTTATTTTTTAAAAACATTTTTTAAA<br>AAACTAAATTACATACAAACTACCGCATAAAATCCCTCAAACTATACAAACGTTTATCGTATAATATATATACATTATTGTTTATTCTATCATTATTAGAGAAACTCCACTACCAAAACCATCATTAAAACAAAAATTTACATGCCAC<br>TTAACTCCCCTCACAAACAATCGTTATTTATATTGTAAATTAACAAACACAAAACCCGC
CamHap_83	ATTTTATTTTTTAACCTAACTCCCCTACTAAGTGTACCCCCCCTTTCCCCCCCAGGGGGGGTATACTATGCATAATCGTGCATACATTTATATACCACATATATTATGGTACCGGTAATATATACTATATATGTACTAAACCCATTAT<br>ATGTATACGGGCATTAATCTATATTCCACATTTCTCCCAATGTCCATTCTATGCATGATCCAGGACATACTCATTCACCCTCCCCATAGACAGCCCCAAACCACTACCAAGTCACCTAACTATGAATGGTTGCAGGACATAAATCT<br>CACTCTCATGCTCTTCCCCCAACAAGTCACCTAACTATGAATGGTTACAGGACATACATTTAACTATC--<br>ATGTTCTAACCCATTTGGTTATGCTCGCCGTATCAGATGGATTTATTGATCGTCCACCTCACGAGAGATCAGCAACCCCTGCCTGTAATGTACTTCATGACCAGTCTCAGGCCCATTCTTTCCCCCTACACCCCTCGCCCTACTTG<br>CCTTCCACCGTACCTCTGGTTCCTCGGTCAGGCACATCCCATGCATAACTCCTGAACTTTCTCACTTTTCACGAAGTCATCTGTGGATTATCTTCCCCTCTTTAGTCCGTGATCGCGGCATCTTCTCTCTTCTATTGCTGTTGGTTC<br>CTTCTCTTTTTGGGGCTTCTTCACAGGTTGCCCTTCACAGTGCGGGTGCGGAGTGCTATTCAAGTGAAGCCTGGACTACACCTGCGTTGCGTCCTATCCTAGTCCTCTCGTGTCCCTCGATGAGACGGTTTGCGTGTATGGGGAAT<br>CATCTTGACACTGATGCACTTTGGATCGCATTTGGTTATGGTTCTTCCACCCCCCC--<br>GGTAAATGGTGCTATTTAGTGAATGCTTGTCGGACATATTTTTATCAATTTTCACTTCCTCTATTTTCTTCACAAAACTAGGAAATTCACCACAATTTTTTCTTTGTTATTTTTTAATTTTTTTTTTATTTTTTAAAAACATTTTTTAAA<br>AAACTAAATTACATACAAACTACCGCATAAAATCCCTCAAACTATACAAACGTTTATCGTATAATATATATACATTATTGTTTATTCTATCATTATTAGAGAAACTCCACTACCAAAACCATCATTAAAACAAAAATTTACATGCCAC<br>TTAACTCCCCTCACAAACAATCGTTATTTATATTGTTAATTAACAAACACAAAACCCGC
CamHap_84	ATTTTATTTTTTAACCTAACTCCCCTACTAAGTGTACCCCCCCTTTCCCCCCCAGGGGGGGTATACTATGCATAATCGTGCATACATTTATATACCACATATATTATGGTACCGGTAATATATACTATATATGTACTAAACCCATTAT<br>ATGTATACGGGCATTAATCTATATTCCACATTTCTCCCAATGTCCATTCTATGCATGATCCAGGACATACTCATTCACCCTCCCCATAGACAGCCCCAAACCACTACCAAGTCACCTAACTATGAATGGTTGCAGGACATAAATCT<br>CACTCTCATGCTCTTCCCCCAACAAGTCACCTAACTATGAATGGTTACAGGACATACATTTAACTATC--<br>ATGTTCTAACCCATTTGGTTATGCTCGCCGTATCAGATGGATTTATTGATCGTCCACCTCACGAGAGATCAGCAACCCCTGCCTGTAATGTACTTCATGACCAGTCTCAGGCCCATTCTTTCCCCCTACACCCCTCGCCCTACTTG<br>CCTTCCACCGTACCTCTGGTTCCTCGGTCAGGCACATCCCATGCATAACTCCTGAACTTTCTCACTTTTCACGAAGTCATCTGTGGATTATCTTCCCCTCTTTAGTCCGTGATCGCGGCATCTTCTCTCTTCTATTGCTGTTGGTTC<br>CTTCTCTTTTTGGGGCTTCTTCACAGGTTGCCCTTCACAGTGCGGGTGCGGAGTGCTATTCAAGTGAAGCCTGGACTACACCTGCGTTGCGTCCTATCCTAGTCCTCTCGTGTCCCTCGATGAGACGGTTTGCGTGTATGGGGAAT<br>CATCTTGACACTGATGCACTTTGGATCGCATTTGGTTATGGTTCTTCCACCCCCCC--<br>GGTAAATGGTGCTATTTAGTGAATGCTTGTCGGACATATTTTTATCAATTTTCACTTCCTCTATTTTCTTCACAAAACTAGGAAATTCACCACAATTTTTTCTTTGTTATTTTTTAATTTTTTTTTTATTTTTTAAAAACATTTTTTAAA<br>AAACTAAATTACATACAAACTACCGCATAAAATCCCTCAAACTATACAAACGTTTATCGTATAATATATATACATTATTGTTTATTCTATCATTATTAGAGAAACTCCACTACCAAAACCATCATTAAAACAAAAATTTACATGCCAC<br>TTAACTCCCCTCACAAACAATCGTTATTTATATTGTTAATTAGCAAACACAAAACCCGC

CamHap_85	ATTTTATTTTTTAACCTAACTCCCCTACTAAGTGTACCCCCCCTTTCCCCCCCAGGGGGGGTATACTATGCATAATCGTGCATACATTTATATACCACATATATTATGGTACCGGTAATATATACTATATATGTACTAAACCCATTAT<br>ATGTATACGGGCATTAATCTATATTCCACATTTCTCCCAATGTCCATTCTATGCATGATCCAGGACATACTCATTCACCCTCCCCATAGACAACTCCAAACCACTACCAAGCCACCTAACTATGAATGGTTGCAGGACATAAATCT<br>CACTCTCATGTTCTTCCCCCAACAAGTCACCTAACTATGAATGGTTGCAGGACATACATTTAACTACT--<br>ATGCTCTAACCCATTTGGTTATGCTCGCCGTATCAGATGGATTTATTGATCGTCCACCTCACGAGAGATCAGCAACCCCTGCCTGTAATGTACTTCATGACCAGTCTCAGGCCCATTCTTTCCCCCTACACCCCTCGCCCTACTTG<br>CCTTCCACCGTACCTCTGGTTCCTCGGTCAGGCACATCCCATGCATAACTCCTGAACTTTCTCACTTTTCACGAAGTCATCTGTGGATTATCTTCCCCTCTTTAGTCCGTGATCGCGGCATCTTCTCTCTTCTATTGCTGTTGGTTC<br>CTTCTCTTTTTGGGGCTTCTTCACAGGTTACCCTTCACAGTGCGGGTGCGGAGTGCTATTCAAGTGAAGCCTGGACTACACCTGCGTTGCGTCCTATCCTAGTCCTCTCGTGTCCCTCGATGAGACGGTTTGCGTGTATGGGGAAT<br>CATCTTGACACTGATGCACTTTGGATCGCATTTGGTTATGGTTCTTCCACCCCCCCC-<br>GGTAAATGGTGCTATTTAGTGAATGCTTGTCGGACATATTTTTATCAATTTTCACTTCCTCTATTTTCTTCACAAAACTAGGAAATTCACCACAATTTTTTCTTTGTTATTTTTTAATTTTTTTTTTATTTTTTAAAAACATTTTTTAAA<br>AAACTAAATTACATACAAACTACCGCATAAAATCCCTCAAACTATACAAACGTTTATCGTATAATATATATACATTATTGTTTATTCTATCATTATTAGAGAAACTCCACTACCAAAACCATCATTAAAACAAAAATTTACATGCCAC<br>TTAACTCCCCTCACAAACAATCGTTATTTATATTGTTAATTAGCAAACACAAAACCCGC
CamHap_86	ATTTTATTTTTTAACCTAACTCCCCTACTAAGTGTACCCCCCCTTTCCCCCCCAGGGGGGGTATACTATGCATAATCGTGCATACATTTATATACCACATATATTATGGTACCGGTAATATATACTATATATGTACTAAACCCATTAT<br>ATGTATACGGGCATTAATCTATATTCCACATTTCTCCCAATGTCCATTTCATGCATGATCCAGGACATACTCATTCACCCTCCCCATAGACAGCTCCAAACCACTACCAAGTCACCTAACTATGAATGGTTACAGGACATAAATCTC<br>ACTCTCATGCTCTTCCCCCAACAAGTCACCTAACTATGAATGGTTACAGGACATACATTTAACTACT--<br>ATGTTCTAACCCATTTGGTTATGCTCGTCGTATCAGATGGATTTATTGATCGTCCACCTCACGAGAGATCAGCAACCCCTGCTTGTAATGTACTTCATGACCAGTCTCAGGCCCATTCTTTCCCCCTACACCCCTCGCCCTACTTG<br>CCTTCCACCGTACCTCTGGTTCCTCGGTCAGGCACATCCCATGCATAACTCCTGAACTTTCTCACTTTTCACGAAGTCATCTGTGGATTATCTTCCCCTCTTTAGTCCGTGATCGCGGCATCTTCTCTCTTCTATTGCTGTTGGTTC<br>CTTCTCTTTTTGGGGCTTCTTCACAGGTTGCCCTTCACAGTGCGGGTGCGGAGTGCTATTCAAGTGAAGCCTGGACTACACCTGCGTTGCGTCCTATCCTAGTCCTCTCGTGTCCCTCGATGAGACGGTTTGCGTGTATGGGGAAT<br>CATCTTGACACTGATGCACTTTGGATCGCATTTGGTTATGGTTCTTCCACCCCCCC--<br>GGTAAATGGTGCTATTTAGTGAATGCTTGTCGGACATATTTTTATCAATTTTCACTTCCTCTATTTTCTTCACAAAACTAGGAAATTCACCACAATTTTTTCTTTGTTATTTTTTAATTTTTTTTTTATTTTTTAAAAACATTTTTTAAA<br>AAACTAAATTACATACAAACTACCGCATAAAATCCCTCAAACTATACAAACGTTTATCGTATAATATATATACATTATTGTTTATTCTATCATTATTAGAGAAACTCCACTACCAAAACCATCATTAAAACAAAAATTTACATGCCAC<br>TTAACTCCCCTCACAAACAATCGTTATTTATATTGTTAATTAGCAAACACAAAACCCGC
CamHap_87	ATTTTATTTTTTAACCTAACTCCCCTACTAAGTGTACCCCCCCTTTCCCCCCCAGGGGGGGTATACTATGCATAATCGTGCATACATTTATATACCACATATATTATGGTACCGGTAATATATACTATATATGTACTAAACCCATTAT<br>ATGTATACGGGCATTAATCTATATTCCACATTTCTCCCAATGTCCATTCTATGCATGATCCAAGACATACTCATTCACCCTCCCCATAGACAGCTCTAAACCACTATCAAGCCACCTAACTATGAATGGTTACAGGACATAAATCTC<br>ACTCTCATGTTCTCCCCCTAACAAGTCACCTAACTATGAATGGTTACAGGACATACATTTAACTACC--<br>ATGTTCTAACCCATTTGGTTATGCTCGCCGTATCAGATGGATTTATTGATCGTCCACCTCACGAGAGATCAGCAACCCCTGCCTGTAATGTACTTCATGACCAGTCTCAGGCCCATTCTTTCCCCCTACACCCCTCGCCCTACTTG<br>CCTTCCACCGTACCTCTGGTTCCTCGGTCAGGCACATCCCATGCATAACTCCTGAACTTTCTCACTTTTCACGAAGTCATCTGTGGATTATCTTCCCCTCTTTAGTCCGTGATCGCGGCATCTTCTCTCTTCTATTGCTGTTGGTTC<br>CTTCTCTTTTTGGGGCTTCTTCACAGGTTGCCCTTCACAGTGCGGGTGCGGAGTGCTATTCAAGTGAAGCCTGGACTACACCTGCGTTGCGTCCTATCCTAGTCCTCTCGTGTCCCTCGATGAGACGGTTTGCGTATATGGGGAAT<br>CATCTTGACACTGATGCACTTTGGATCGCATTTGGTTATGGTTCTTCCACCCCCCCC-<br>GGTAAATGGTGCTATTTAGTGAATGCTTGTCGGACATATTTTTATCAATTTTCACTTCCTCTATTTTCTTCACAAAACTAGGAAATTCACCACAATTTTTTCTTTGTTATTTTTTAATTTTTTTTTTATTTTTTAAAAACATTTTTTAAA<br>AAACTAAATTACATACAAACTACCGCATAAAATCCCTCAAACTATACAAACGTTTATCGTATAATATATATACATTATTGTTTATTCTATCATTATTAGAGAAACTCCACTACCAAAACCATCATTAAAACAAAAATTTACATGCCAC<br>TTAACTCCCCTCACAAACAATCGTTATTTATATTGTTAATTAGCAAACACAAAACCCAC
CamHap_88	ATTTTATTTTTTAACCTAACTCCCCTACTAAGTGTACCCCCCCTTTCCCCCCCAGGGGGGGTATACTATGCATAATCGTGCATACATTTATATACCACATATATTATGGTACCGGTAATATATACTATATATGTACTAAACCCATTAT<br>ATGTATACGGGCATTAATCTATATTCCACATTTCTCCCAATGTCCATTCTATGCATGATCCAGGACATACTCATTCACCCTCCCCATAGACAGCTCCAAACCACTACCAAGTCACCTAACTATGAATGGTTGCAGGACATAAATCTC<br>ACTCTCATGCTCTTCCCCCAACAAGTCACCTAACTATGAATGGTTGCAGGACATACATTTAACTACC--<br>ATGTTCTAACCCATTTGGTTATGCTCGCCGTATCAGATGGATTTATTGATCGTCCACCTCACGAGAGATCAGCAACCCCTGCCTGTAATGTACTTCATGACCAGTCTCAGGCCCATTCTTTCCCCCTACACCCCTCGCCCTACTTG<br>CCTTCCACCGTACCTCTGGTTCCTCGGTCAGGCACATCCCATGCATAACTCCTGAACTTTCTCACTTTTCACGAAGTCATCTGTGGATTATCTTCCCCTCTCTAGTCCGTGATCGCGGCATCTTCTCTCTTCTATTGCTGTTGGTTC<br>CTTCTCTTTTTGGGGCTTCTTCACAGGTTGCCCTTCACAGTGCGGGTGCGGAGTGCTATTCAAGTGAAGCCTGGACTACACCTGCGTTGCGTCCTATCCTAGTCCTCTCGTGTCCCTCGATGAGACGGTTTGCGTGTATGGGGAAT<br>CATCTTGACACTGATGCACTTTGGATCGCATTTGGTTATGGTTCTTCCACCCCCCCC-<br>GGTAAATGGTGCTATTTAGTGAATGCTTGTCGGACATATTTTTATCAATTTTCACTTCCTCTATTTTCTTCACAAAACTAGGAAATTCACCACAATTTTTTCTTTGTTATTTTTTAATTTTTTTTTTATTTTTTAAAAACATTTTTTAAA<br>AAACTAAATTACATACAAACTACCGCATAAAATCCCTCAAACTATACAAACGTTTATCGTATAATATATATACATTATTGTTTATTCTATCATTATTAGAGAAACTCCACTACCAAAACCATCATTAAAACAAAAATTTACATGCCAC<br>TTAACTCCCCTCACAAACAATCGTTATTTATATTGTTAATTAGCAAACACAAAACCCGC

CamHap_89	ATTTTATTTTTTAACCTAACTCCCCTACTAAGTGTACCCCCCCTTTCCCCCCCAGGGGGGGTATACTATGCATAATCGTGCATACATTTATATACCACATATATTATGGTACCGGTAATATATACTATATATGTACTAAACCCATTAT<br>ATGTATACGGGCATTAATCTATATTCCACATTTCTCCCAATGTCCATTCTATGCATGATCCAGGACATACTCATTCACCCTCCCTACAGACAGCTCCAAACCACCACCAAGTCACCTAACTATGAATGGTTACAGGACATAAATCT<br>CACTCTCATGTTCTTCCCCTACCAAGTCACCTAACTATGAATGGTTACAGGACATACATTTAACTACC---<br>ATGTTCTAACCCATTTGGTTATGCTCGCCGTATCAGATGGATTTATTGATCGTCCACCTCACGAGAGATCAGCAACCCCTGCTTGTAATGTACTTCATGACCAGTCTCAGGCCCATTCTTTCCCCCTACACCCCTCGCCCTACTTG<br>CCTTCCACCGTACCTCTGGTTCCTCGGTCAGGCACATCCCATGCATAACTCCTGAACTTTCTCACTTTTCACGAAGTCATCTGTGGATTATCTTCCCCTCTTTAGTCCGTGATCGCGGCATCTTCTCTCTTCTATTGCTGTTGGTTC<br>CTTCTCTTTTTGGGGCTTCTTCACAGGTTACCCTTCACAGTGCGGGTGCGGAGTGCTATTCAAGTGAAGCCTGGACTACACCTGCGTTGCGTCCTATCCTAGTCCTCTCGTGTCCCTCGATGAGACGGTTTGCGTGTATGGGGAAT<br>CATCTTGACACTGATGCACTTTGGATCGCATTTGGTTATGGTTCTTCCACCCCCCC--<br>GGTAAATGGTGCTATTTAGTGAATGCTTGTCGGACATATTTTTACCAATTTTCACTTCCTCTATTTTCTTCACAAAACTAGGAAATTCACCACAATTTTTTCTTTGTTATTTTTTAATTTTTTTTTTATTTTTTAAAAACATTTTTTAAA<br>AAACTAAATTACATACAAACTACCGCATAAAATCCCTCAAACTATACAAACGTTTATCGTATAATATATATACATTATTGTTTATTCTATCATTATTAGAGAAACTCCACTACCAAAACCATCATTAAAACAAAAATTTACATGCCAC<br>TTAACTCCCCTCACAAACAATCGTTATTTATATTGTTAATTAGCAAACACAAAACCTGC
CamHap_90	ATTTTATTTTTTAACCTAACTCCCCTACTAAGTGTACCCCCCCTTTCCCCCCCAGGGGGGGTATACTATGCATAATCGTGCATACATTTATATACCACATATATTATGGTACCGGTAATATATACTATATATGTACTAAACCCATTAT<br>ATGTATACGGGCATTAATCTATATTCCACATTTCTCCCAATGTCCATTCTATGCATGATCCAGGACATACTCATTCACCCTCCCCATAGACAGCTCCAAACCACTACCAAGTCACCTAACTATGAATGGTTGCAGGACATAAATCTC<br>ACTCTCATGCTCTTCCCCCAACAAGTCACCTAACTATGAATGGTTACAGGACATACATTTAACTACC---<br>ATGTTCTAACCCATTTGGTTATGCTCGCCGTATCAGATGGATTTATTGATCGTCCACCTCACGAGAGATCAGCAACCCCTGCCTGTAATGTACTTCATGACCAGTCTCAGGCCCATTCTTTCCCCCTACACCCCTCGCCCTACTTG<br>CCTTCCACCGTACCTCTGGTTCCTCGGTCAGGCACATCCCATGCATAACTCCTGAACTTTCTCACTTTTCACGAAGTCATCTGTGGATTATCTTCCCCTCTCTAGTCCGTGATCGCGGCATCTTCTCTCTTCTATTGCTGTTGGTTC<br>CTTCTCTTTTTGGGGCTTCTTCACAGGTTGCCCTTCACAGTGCGGGTGCGGAGTGCTATTCAAGTGAAGCCTGGACTACACCTGCGTTGCGTCCTATCCTAGTCCTCTCGTGTCCCTCGATGAGACGGTTTGCGTGTATGGGGAAT<br>CATCTTGACACTGATGCACTTTGGATCGCATTTGGTTATGGTTCTTCCACCCCCCC--<br>GGTAAATGGTGCTATTTAGTGAATGCTTGTCGGACATATTTTTATCAATTTTCACTTCCTCTATTTTCTTCACAAAACTAGGAAATTCACCACAATTTTTTCTTTGTTATTTTTTAATTTTTTTTTTATTTTTTAAAAACATTTTTTAAA<br>AAACTAAATTACATACAAACTACCGCATAAAATCCCTCAAACTATACAAACGTTTATCGTATAATATATATACATTATTGTTTATTCTATCATTATTAGAGAAACTCCACTACCAAAACCATCATTAAAACAAAAATTTACATGCCAC<br>TTAACTCCCCTCACAAACAATCGTTATTTATATTGTTAATTAGCAAACACAAAACCCGC
CamHap_91	ATTTTATTTTTTAACCTAACTCCCCTACTAAGTGTACCCCCCCTTTCCCCCCCAGGGGGGGTATACTATGCATAATCGTGCATACATTTATATACCACATATATTATGGTACCGGTAATATATACTATATATGTACTAAACCCATTAT<br>ATGTATACGGGCATTAACCTATATTCCACATTTCTCCCAATGTCCATTCTATGCATGATCCAGGACATACTCATTTACCCTCCCCATAGACAGTTCCAAACCACTATCAAGCCACCTAACTATGAATGGTTACAGGACATAAATCTC<br>ACTCTCATGTTCTCCCCCCAACAAGTCACCTAATTATGAATGGTTACAGGACATACATTTAACTACC---<br>ATGTTCTAACCCATTTGGTTATGCTCGCCGTATCAGATGGATTTATTGATCGTCCACCTCACGAGAGATCAGCAACCCCTGCCTGTAATGTACTTCATGACCAGTCTCAGGCCCATTCTTTCCCCCTACACCCCTCGCCCTACTTG<br>CCTTCCACCGTACCTCTGGTTCCTCGGTCAGGCACATCCCATGCATAACTCCTGAACTTTCTCACTTTTCACGAAGTCATCTGTGGATTATCTTCCCCTCTTTAGTCCGTGATCGCGGCATCTTCTCTCTTCTATTGCTGTTGGTTC<br>CTTCTCTTTTTGGGGCTTCTTCACAGGTTGCCCTTCACAGTGCGGGTGCGGAGTGCTATTCAAGTGAAGCCTGGACTACACCTGCGTTGCGTCCTATCCTAGTCCTCTCGTGTCCCTCGATGAGACGGTTTGCGTGTATGGGGAAT<br>CATCTTGACACTGATGCACTTTGGATCGCATTTGGTTATGGTTCTTCCACCCCCCC--<br>GGTAAATGGTGCTATTTAGTGAATGCTTGTCGGACATATTTTTATCAATTTTCACTTCCTCTATTTTCTTCACAAAACTAGGAAATTCACCACAATTTTTTCTTTGTTATTTTTTAATTTTTTTTTTATTTTTTAAAAACATTTTTTAAA<br>AAACTAAATTACATACAAACTACCGCATAAAATCCCTCAAACTATACAAACGTTTATCGTATAATATATATACATTATTGTTTATTCTATCATTATTAGAGAAACTCCACTACCAAAACCATCATTAAAACAAAAATTTACATGCCAC<br>TTAACTCCCCTCACAAACAATCGTTATTTATATTGTAAATTAGCAAACACAAAACCCGC
CamHap_92	ATTTTATTTTTTAACCTAACTCCCCTACTAAGTGTACCCCCCCTTTCCCCCCCAGGGGGGGTATACTATGCATAATCGTGCATACATTTATATACCACATATATTATGGTACCGGTAATATATACTATATATGTACTAAACCCATTAT<br>ATGTATACGGGCATTAATCTATATTCCACATTTCTCCCAATGTCCATTCTATGCATGATCCAGGACATACTCATTCACCCTCCCCATAGACAGCCCCAAACCACCACCAAGTCACCTAACTATGAATGGTTGCAGGACATAAATCT<br>CACTCTCATGCTCTTCCCCCAACAAGTCACCTAACTATGAATGGTTACAGGACATACATTTAACTACC---<br>ATGTTCTAACCCATTTGGTTATGCTCGCCGTATCAGATGGATTTATTGATCGTCCACCTCACGAGAGATCAGCAACCCCTGCCTGTAATGTACTTCATGACCAGTCTCAGGCCCATTCTTTCCCCCTACACCCCTCGCCCTACTTG<br>CCTTCCACCGTACCTCTGGTTCCTCGGTCAGGCACATCCCATGCATAACTCCTGAACTTTCTCACTTTTCACGAAGTCATCTGTGGATTATCTTCCCCTCTTTAGTCCGTGATCGCGGCATCTTCTCTCTTCTATTGCTGTTGGTTC<br>CTTCTCTTTTTGGGGCTTCTTCACAGGTTGCCCTTCACAGTGCGGGTGCGGAGTGCTATTCAAGTGAAGCCTGGACTACACCTGCGTTGCGTCCTATCCTAGTCCTCTCGTGTCCCTCGATGAGACGGTTTGCGTGTATGGGGAAT<br>CATCTTGACACTGATGCACTTTGGATCGCATTTGGTTATGGTTCTTCCACCCCCCC--<br>GGTAAATGGTGCTATTTAGTGAATGCTTGTCGGACATATTTTTATCAATTTTCACTTCCTCTATTTTCTTCACAAAACTAGGAAATTCACCACAATTTTTTCTTTGTTATTTTTTAATTTTTTTTTTATTTTTTAAAAACATTTTTTAAA<br>AAACTAAATTACATACAAACTACCGCATAAAATCCCTCAAACTATACAAACGTTTATCGTATAATATATATACATTATTGTTTATTCTATCATTATTAGAGAAACTCCACTACCAAAACCATCATTAAAACAAAAATTTACATGCCAC<br>TTAACTCCCCTCACAAACAATCGTTATTTATATTGTTAATTAACAAACACAAAACCCGC
